# What Individuals Experience During Visuo-Spatial Working Memory Task Performance: An Exploratory Phenomenological Study

**DOI:** 10.3389/fpsyg.2022.811712

**Published:** 2022-05-18

**Authors:** Aleš Oblak, Anka Slana Ozimič, Grega Repovš, Urban Kordeš

**Affiliations:** ^1^Laboratory for Cognitive Neuroscience and Psychopathology, University Psychiatric Hospital Ljubljana, Ljubljana, Slovenia; ^2^Mind & Brain Lab, Department of Psychology, Faculty of Arts, University of Ljubljana, Ljubljana, Slovenia; ^3^Center for Cognitive Science, Faculty of Education, University of Ljubljana, Ljubljana, Slovenia; ^4^Observatory: Laboratory for Empirical Phenomenology, Faculty of Education, University of Ljubljana, Ljubljana, Slovenia

**Keywords:** visuo-spatial working memory, empirical phenomenology, psychological task, constructivist grounded theory, background feelings

## Abstract

In experimental cognitive psychology, objects of inquiry are typically operationalized with psychological tasks. When interpreting results from such tasks, we focus primarily on behavioral measures such as reaction times and accuracy rather than experiences – i.e., phenomenology – associated with the task, and posit that the tasks elicit the desired cognitive phenomenon. Evaluating whether the tasks indeed elicit the desired phenomenon can be facilitated by understanding the experience during task performance. In this paper we explore the breadth of experiences that are elicited by and accompany task performance using in-depth phenomenological and qualitative methodology to gather subjective reports during the performance of a visuo-spatial change detection task. Thirty-one participants (18 females) were asked to remember either colors, orientations or positions of the presented stimuli and recall them after a short delay. Qualitative reports revealed rich experiential landscapes associated with the task-performance, suggesting a distinction between two broad classes of experience: phenomena at the front of consciousness and background feelings. The former includes cognitive strategies and aspects of metacognition, whereas the latter include more difficult-to-detect aspects of experience that comprise the overall sense of experience (e.g., bodily feelings, emotional atmosphere, mood). We focus primarily on the background feelings, since strategies of task-performance to a large extent map onto previously identified cognitive processes and discuss the methodological implications of our findings.

## Introduction

In experimental cognitive psychology, the objects of study are usually operationalized with psychological tasks. Many readers have probably participated in studies with such tasks, sitting in front of a computer screen with the typical light gray background, and waiting for stimuli to appear. In well-established domains of research, these tasks are standardized so that they can be easily modified to answer specific research questions. When interpreting the results of psychological tasks, we focus primarily on behavioral measures such as reaction times and performance accuracy rather than phenomenology. We assume that the psychological tasks elicit the desired cognitive phenomenon (cf. Morrison et al., 2019) and that we can infer underlying cognitive mechanisms based on behavioral performance.

For example, when participants perform a working memory task—where working memory is considered one of the most important cognitive functions, encompassing many complex cognitive operations (Baddeley and Hitch, 1974; Repovš and Baddeley, 2006; Baddeley, 2010)—it is assumed that the phenomenon that the task is intended to elicit (i.e., working memory) is the primary content of participants’ conscious awareness. Based on this assumption, behavioral data are used to make inferences about the cognitive mechanisms of working memory (for an overview of this type of reasoning, see Caramazza, 1986; De Hollander et al., 2016; Coltheart, 2017).

If we are interested in measuring the target phenomena, we need to examine the validity of the task: understood here in the broadest sense as whether the task elicits the phenomenon in question (Stone, 2019). Assessing validity and interpreting behavioral data can be facilitated by understanding the experience while performing the psychological task (cf. Jaspers, 1997). In cognitive science, phenomenology has traditionally been used to study phenomena in naturalistic settings (e.g., Hurlburt, 1990; Gallagher et al., 2015; Oblak, 2020). When subjective reports are collected in laboratory settings, it is typically done to ensure that the task is working as intended. For example, Nelson et al. (2003) used a verbal change-detection task in which they manipulated the frequency of stimuli to elicit a familiarity-related conflict (e.g., a negative probe in the current trial was positive in the preceding trial). At the end of the experiment, a debriefing interview was used to determine whether participants were aware of this manipulation. More recently, however, in-depth phenomenological interviews have also been used with experimental tasks to assess whether the task elicits the desired phenomenology or to determine what phenomenology is associated with the task in the first place (for a theoretical overview, see Weger and Wagemann, 2015; Wagemann, 2021; for empirical examples, see Valenzuela-Moguillansky et al., 2013; Hurlburt et al., 2016).

Studies that combine phenomenological methods and psychological tasks focus primarily on task-related phenomenology. One aspect of task-related phenomenology that has been explored most are cognitive strategies. Seghier and Price (2018) propose that the same psychological tasks can be solved using different cognitive strategies (for empirical examples, see Coltheart et al., 1993; Tsukiura et al., 2005; Cummine et al., 2013; Braga et al., 2017). In spatial navigation tasks, to remember space, participants may rely on unitary encoding strategies (i.e., a single object is remembered based on cardinal directions), binary encoding strategies (i.e., objects are remembered based on the spatial relationships between them), or a combination of both (König et al., 2017). Questionnaire surveys revealed four cognitive styles that determine the strategy used to store stimuli in a memory retrieval task: verbal, visual, spatial, and image-based strategies (Miller et al., 2012); and two strategies used in performing a spatial working memory task: an auditory and a visuospatial strategy (Sanfratello et al., 2014). Furthermore, eye-tracker data distinguish between categorical and detail-based strategies when performing a spatial working memory task (Starc et al., 2017). In memory tasks, subtle changes in strategy, such as focusing on maintenance or retrieval, are associated with behavioral differences in performance (Speer et al., 2003). The use of different strategies in the same psychological task may imply that the task does not elicit the desired phenomenon and that the validity of the task may be in question (for discussions of the relationship between phenomenology, behavior, and neural dynamics, see Mcintyre, 1999; Mogensen and Overgaard, 2018).

To overcome this problem in the working memory domain, researchers use a variety of approaches, including the use of control tasks. For example, studies may be specifically interested in visual working memory and therefore want to control for the specific type of representation that participants are encoding. The possibility that participants encode stimuli in auditory form (i.e., by naming them in inner speech) rather than visual form is commonly controlled using so-called distractor tasks (e.g., articulatory suppression), in which participants must—in parallel with visual working memory task performance—continuously repeat a specific verbal phrase that prevents them from encoding and maintaining the target stimuli in auditory form (Barrouillet et al., 2007).

In addition to the various strategies participants experience when performing psychological tasks, psychological tasks are typically accompanied by a variety of confounding phenomenology, such as boredom (D’Angiulli and Smith LeBeau, 2002), anxiety (Ikeda et al., 1996), mind-wandering (Morrison et al., 2019), and task-related emotions (Laybourn et al., 2022). While working memory has been studied from a phenomenological perspective (Buchsbaum, 2013), to the best of our knowledge, novel approaches in first-person research (e.g., descriptive experience sampling, Hurlburt, 2011; micro-phenomenological interview, Petitmengin, 2006; for reviews of these methodological approaches, see Froese et al., 2011a,b; Valenzuela-Moguillansky and Demšar, 2022) have not been applied to this phenomenon. The full range of experiences that can be elicited by working memory tasks is therefore unknown.

The aim of this paper is to contribute to the understanding of working memory task performance by exploring the range of experiences evoked by and accompanying task performance. We consider such mapping of the space of experiences as a first step that can then guide further, more detailed and focused investigations of how the identified experiences may affect task performance and its neural correlates. By using a qualitative methodology to explore experiences during task performance, we aim to address four challenges: (i) the unreliability of current closed-form approaches (e.g., questionnaires, semi-structured debriefing interviews); (ii) limiting the exploration of confounding phenomena to a limited an incomplete set of *a priori* categories; (iii) incomplete interpretations of quantitative inferences in the absence of supporting qualitative findings; (iv) understanding the source of noise in the data. We discuss these aspects in more detail in the following paragraphs.

First, although there are several studies that attempt to understand differences in the strategies used in psychological tasks in general and working memory tasks in particular (see above), they usually rely on indirect measures. The existence of different strategies is inferred from behavioral or physiological data (e.g., Starc et al., 2017). Moreover, it is assumed that cognitive styles and strategies are sufficiently understood, so closed-form instruments (e.g., questionnaires) are often used to collect data on them. In recent decades, phenomenology has entered cognitive science (Varela et al., 1991; Flannagan, 1992; Thompson, 2007). Notably, an in-depth and systematic look at how individuals experience the world has been associated with a wide-range reexamination of what are the objects of inquiry in the sciences of the mind (for how phenomenology reexamined diagnostic criteria in the RDoC framework in psychopathology, for example, see Cuthbert and Insel, 2010). Such reexaminations have proven to be especially problematic for the use of closed-form questionnaires.

The second reason for choosing a qualitative methodology is that the assumption that phenomena accompanying psychological tasks are known may be invalid and needs to be empirically assessed. As noted earlier, studies using psychological tasks assume that participants experience only task-related cognitions when engaged in the task. However, as one study (Morrison et al., 2019) has shown, experiences during task performance can be quite different from what researchers intended—for a broader discussion of how the phenomenology of interaction with complex systems cannot be known *a priori* and requires further investigation, see theory-experience gap in Froese et al. (2012). Of particular interest are aspects of experience that are well known in the phenomenological tradition—e.g., existential feelings (Ratcliffe, 2008), background consciousness (Colombetti, 2011), and fringe awareness (James, 1890)—, but have not yet been explored in working memory research (although for a similar approach, see Laybourn et al., 2022). In-depth interviews can identify and provide insights into these and other aspects of experience that theory-driven questionnaires may fail to capture.

The third reason for choosing a qualitative methodology to investigate the experience during visuo-spatial working memory task performance is related to the ongoing replication crisis (Ioannidis, 2005). Recently, a new interpretation of the replication crisis has been proposed: the so-called generalizability crisis. Namely, the generalizability crisis stems in part from the fact that statistical claims in research papers about phenomena are incompatible with qualitative claims. It has been argued that commonly, there is no link between statistically testable experiments (presented in section “Materials and Methods”) and the broader claims made about the phenomena of inquiry (presented in section “Introduction” and “Discussion”), and that this gap might be addressed with formal qualitative research (Yarkoni, 2022). In his seminal monograph on descriptive experience sampling, Hurlburt (2011, chapter 21) states that first-person reports should not serve merely as the initial, exploratory step in the study of a phenomenon. Rather, at each step of theory-construction and conducting experimental research, we should reevaluate whether we are still engaging with the target phenomenology or whether we have begun to engage with theoretical abstractions.

Finally, to understand and manage variability in behavioral and neural data, it is important to have a comprehensive overview of possible sources of “noise”— phenomena that accompany the cognitive process of interest (Seghier and Price, 2018). Our final aim, therefore, was to capture the breadth of experience during the performance of a working memory task in order to build a taxonomy of experience categories that can be used in future studies (cf. Lutz et al., 2002; Hurlburt et al., 2016; Fernyhough et al., 2018). Importantly, our aim was not to examine inter-subject variability along experimental dimensions, but rather to examine the breadth of different experiences that participants may have while performing the working memory task.

To date, only two studies have used modern approaches to phenomenal data collection to incorporate the study of experience into the analysis of task performance. First, it has been shown that during performance of simple visual tasks, different strategies accessible with subjective reports are associated with different electrophysiological signatures (Lutz et al., 2002). Second, elicited and spontaneously occurring inner speech are associated not only with different but also opposite patterns of neural activity recorded by fMRI (Hurlburt et al., 2016; Fernyhough et al., 2018). To investigate the experience during visuo-spatial working memory task performance, we used an in-depth phenomenological and qualitative methodology, in particular constructivist grounded theory (Charmaz, 2004). This approach aims to outline in detail the structure of a given phenomenon. Thus, it is interested in describing the widest possible range of different experiences associated with a phenomenon. To facilitate detailed descriptions, constructivist grounded theory gathers as diverse data as possible from as many different sources as possible. Thus, we collected in-depth qualitative data from a heterogeneous sample of participants with different ages, educational backgrounds, and experience in mind sciences in different visuo-spatial working memory tasks. The data collected suggested a clear distinction between two broad classes of experiences: phenomena at the forefront of consciousness and background feelings. The former includes strategies and aspects of metacognition related to task performance, whereas the latter include descriptions of the overall impression of the experience (e.g., bodily feelings, emotional atmosphere, mood). We present all experiential categories induced in this study. However, because task performance strategies largely correspond to task-related cognitive processes identified in previous research, we focus primarily on background feelings.

## Materials and Methods

A combination of a computerized behavioral task designed to investigate visuo-spatial working memory and an in-depth phenomenological interview was used to gather first-person data. Participants were asked to attend four interview sessions. During each session, participants were asked to solve multiple trials of the visuo-spatial working memory task. At a random moment during task-performance, participants were prompted to report on their experience. A phenomenological interview followed. The interview investigated both how participants’ experience evolved through time during the trial immediately before the prompt, as well as what they experienced during each moment of the trial. Additionally, behavioral data on their task-performance (i.e., performance accuracy and reaction times) were gathered but are not reported in this paper and will be presented elsewhere.

Audio recordings of the interviews were analyzed according to the principles of constructivist grounded theory. Our main analytical instrument was coding (i.e., assigning general descriptive tags to sections of raw data). Three stages of coding took place. First, codes were induced solely from the data. Second, extant categories were compared to as-of-yet uncoded data, while novel categories were induced. Third, all the categories were fitted to the data. In line with principles of qualitative research, data acquisition and analysis were conducted in parallel, one informing the other. Then, relational coding was used to establish logical relationships between individual experiential categories constructed via coding. Finally, a codebook was constructed, outlining both individual experiential categories, and the relationships between them. In a subset of participants, the resulting codebook was validated in additional interviews.

**FIGURE 1:**
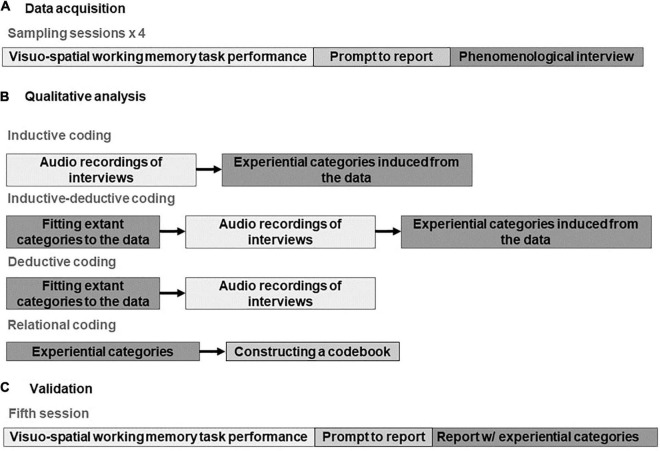
**(A–C)** Overview of the research design.

Figure 1 provides a schematic outline of the research design. Each aspect of the research design is presented in detail in subsequent subsections.

### Participants

Thirty-one participants (18 females) aged between 20 and 50 years (*M* = 27.0, *SD* = 6.21; years of education: *M* = 16.52, *SD* = 2.35) signed an informed consent to participate in four 60-min study sessions in which they performed a visuo-spatial working memory task. All participants had normal or corrected-to-normal eyesight. Participants did not self-report any neurological or psychiatric disorders. All but three participants were right-handed. The sample size was determined based on conceptual depth of the data (see section “Data Analysis”), and by the expectation that it would match or exceed the sample size in a typical working memory study (usually, between 20 and 30 participants, e.g., Bo and Seidler, 2009). 16 participants were students of cognitive science at the University of Ljubljana. Nine of them participated in the study as a part of their coursework. Since their participation was therefore not strictly speaking voluntary, they were given the option for their data to not be used in the analysis. None of the participants opted for having their data removed. To avoid the possibility of gathering data that are biased by theoretical understanding of either working memory or phenomenology, additional 15 participants who had no background in mind sciences were recruited. While quantitative studies ask for samples to be as homogenous as possible, the aims of grounded theory differ and a varied and heterogenous sample is desirable so as to account for as broad a range of dimensions associated with the phenomenon of inquiry as possible (cf. Charmaz, 2004). The nine participants who were involved in the study as a part of the course in first-person research received course credits. For non-student participants no reward was given in exchange.

### Instruments

#### Visuo-Spatial Change Detection Task

**FIGURE 2:**
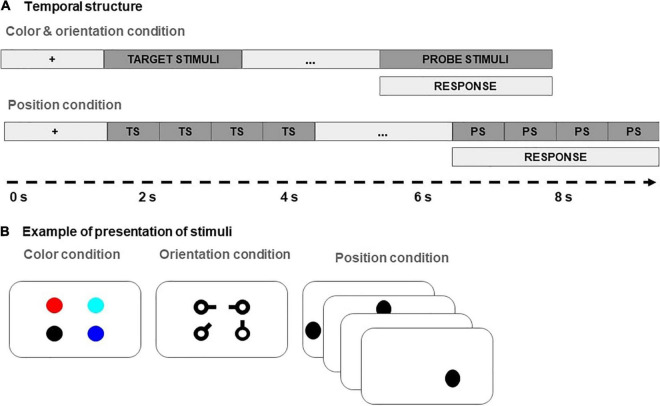
**(A)** Timing structure; **(B)** stimuli of the experiment.

The participants completed multiple trials of the visuo-spatial change detection task, in which they were asked to memorize orientations, colors or positions of the presented stimuli. The timing structure of each trial is presented in Figure 2. Briefly, in the remember color and remember orientation conditions, four target stimuli were presented simultaneously for 2.0 s. In the remember position, the target stimuli were presented sequentially, each for the duration of 0.75 s. Following a maintenance delay of 2.0 s, the probe stimuli were shown, and the participants had to indicate by a button press whether there was a change in the relevant property in any of the stimuli. Both the accuracy and reaction times of the responses were recorded. If the participant did not respond within 2.5 s, a null response was recorded, and the participant proceeded to the next trial.

The visuo-spatial change detection task was presented on an Acer Aspire 3 laptop (Intel Core i5 processor with 2.50 GHz and 3 MB RAM) running Windows 10 Pro operating system. The stimuli were presented on a 15.6-inch full LED screen with 1920 × 1080 resolution and 60 Hz refresh rate. The screen was set to maximum brightness (224 cd/m2) when the task was being performed. Participants sat approximately 75 cm away from the center of the screen.

A custom script prepared in PsychoPy (Peirce, 2007) was used to present the stimuli and collect responses. Stimuli in the orientation condition were black keys (see Figure 2), 35 px (0.55° visual angle) in length, with the main circle 19 px (0.3° visual angle) in diameter and the line 7 px (0.11° visual angle) wide. The orientation of each stimulus was randomly selected from eight possible principal directions pointing toward 0, 45, 90, 135, 180, 225, 270, and 315°. Stimuli in the color condition were colored circles, 35 px (0.55° visual angle) in diameter. The color of each stimulus was randomly selected from eight easily distinguishable color hues: red, dark blue, light blue, green, yellow, purple, and white. In the position condition, the stimuli were black circles, 20 px (0.32° visual angle) in diameter.

Stimuli in all conditions were presented on a gray background within an invisible square bounding box 520 px (8.14° visual angle) in width positioned in the center of the screen. The position of the orientation and color stimuli was fixed in the vertices of the invisible square. The exact position of the stimuli within the square varied randomly between trials, with the requirement that the minimal distance between the centers of each pair of objects be at least 4 times their bounding radius.

#### The Phenomenological Interview

The automated change-detection task was designed to allow pausing after any trial and allow for experiential sampling. Specifically, between the seventh and the fifteenth trial, the interviewer paused task execution after participants responded to the probe stimuli. The interviewer then asked the participants to stop performing the task and reflect on their experience. The interviewer guided them through the empirical phenomenological interview in which participants’ subjective reports were gathered.

The interview was designed to address the research question (i.e., how participants experience solving the visuo-spatial working memory task). It integrated interviewing approaches from qualitative research–specifically, constructivist grounded theory (Charmaz, 2004; Mills et al., 2006a,b; Charmaz and Belgrave, 2007)–and extant empirical phenomenological approaches, such as second-person in-depth phenomenological inquiry (Kordeš and Klauser, 2016), micro-phenomenological interview (Petitmengin, 2006), and descriptive experience sampling (Hurlburt, 2011).

Constructivist grounded theory and second-person in-depth phenomenological inquiry are both approaches that consider the constructive role of both the researcher and the participants in the process of knowledge-creation. The methods require participants to be highly engaged with the study. In turn, the interested position of the participants–unlike some contemporary approaches to first-person research (e.g., Petitmengin, 2006; Hurlburt, 2011)–allow the participants to go beyond an open-ended style of interviewing. Focused questions allowing for further theory-construction may be asked (cf. Charmaz, 2004).

The descriptive experience sampling technique is based on randomly sampling experiential episodes of individuals who are trained to observe and report their experience. We modified the typical descriptive experience sampling approach, which is based on sampling experience in an ecological setting, by inducing the target experience (i.e., solving a visuo-spatial working memory task) in a laboratory setting.

Initially, the interviewer asked about the overall temporal structure of the participants’ experience: what were the salient events that took place in their awareness and what was their temporal succession relative to the behavioral and functional structure of the working memory task (i.e., presentation of the target stimuli/encoding, delay/maintenance, probe stimuli/recall and response). Then, the interviewer guided the participant back to the earliest moment they reported on and gathered its detailed description. The interviewer guided the participants away from general statements, descriptions of their beliefs about their experience, folk-psychological theories, and scientific (psychological and phenomenological) concepts (Petitmengin et al., 2019; Valenzuela-Moguillansky and Vásquez-Rosati, 2019). When descriptions of all salient experiences were grounded in bodily feelings, mental gestures, specific sensory modalities, and/or attitudes, the interviewer guided the participants to the next event. When the participant reported that there is nothing more to add regarding the trial, they were invited to return to the working memory task.

Audio recordings of the interviews were gathered using an Olympus WS-852 digital voice recorder.

### Procedure

Participants were asked to take part in four study sessions, each lasting 60 min. The four sessions took place within 2 weeks^1^. Data were collected in an empty classroom with a table sectioned off for research purposes. During the task performance, the interviewer sat behind the participants.

During the first session, after signing the consent form, the participants were told how to perform the task. This included instructions for the visuo-spatial change detection task, as well as how to observe their experience. Prior to the beginning of each session, the interviewer provided a verbal description of both the working memory task and the interviewing protocol. It was emphasized that the participants should set aside their assumptions about the nature of their experience and cognition and focus only on the events and processes that occur in their awareness.

During each session, two task conditions were tested. One condition was concluded at the 30-min mark of the session. To avoid overwhelming participants with too many task parameters, the stimuli with simultaneous presentation (color and orientation) were presented at the first session. Within it, half of the participants started with the color condition and half with the orientation condition. Sequential presentation (position) was introduced in the first part of the second session. In the next session, conditions were randomized. When a new task condition was introduced, the participants were given a sample trial. The procedure was repeated until an hour elapsed.

A preliminary qualitative analysis of each session took place within 24 h after the interview. These analyses informed what follow-up questions were asked in subsequent interviews. In other words, data acquisition and initial data analysis were conducted in parallel. The process of parallel analysis allowed us to focus on the salient aspects of experience during the interview process itself and narrow our focus on the data directly addressing our research question (cf. Charmaz, 2004; Flick, 2009; Kordeš and Klauser, 2016).

### Data Analysis

A total of 124 sessions were conducted across all the participants. All in all, we gathered 501 samples of experience (on average, 16.2 per participant). On average, participants received 4.0 prompts per session. A handful of participants expressed a higher degree of interest in exploring their experience. Sessions with them consisted of fewer samples that were explored in more detail.

Based on current standards in first-person research (Hurlburt, 2011; Valenzuela-Moguillansky and Vásquez-Rosati, 2019), two criteria for determining the validity of individual samples were followed: (a) whether the participants focused their description on a single trial; and (b) whether they described their lived experience rather than theories, assumptions, or beliefs) (e.g., statements such as “I simply saved it in my working memory.” or “I stored it in my brain.”). If a given sample did not reach these criteria, we eliminated the sampled experience from the analysis. Commonly, first sessions were eliminated entirely. 376 samples (133 for the color condition, 134 for the orientation condition, and 109 for the position condition) of the experience of solving the change-detection task were considered valid and were analyzed further.

#### Analysis of the Interviews

**FIGURE 3:**
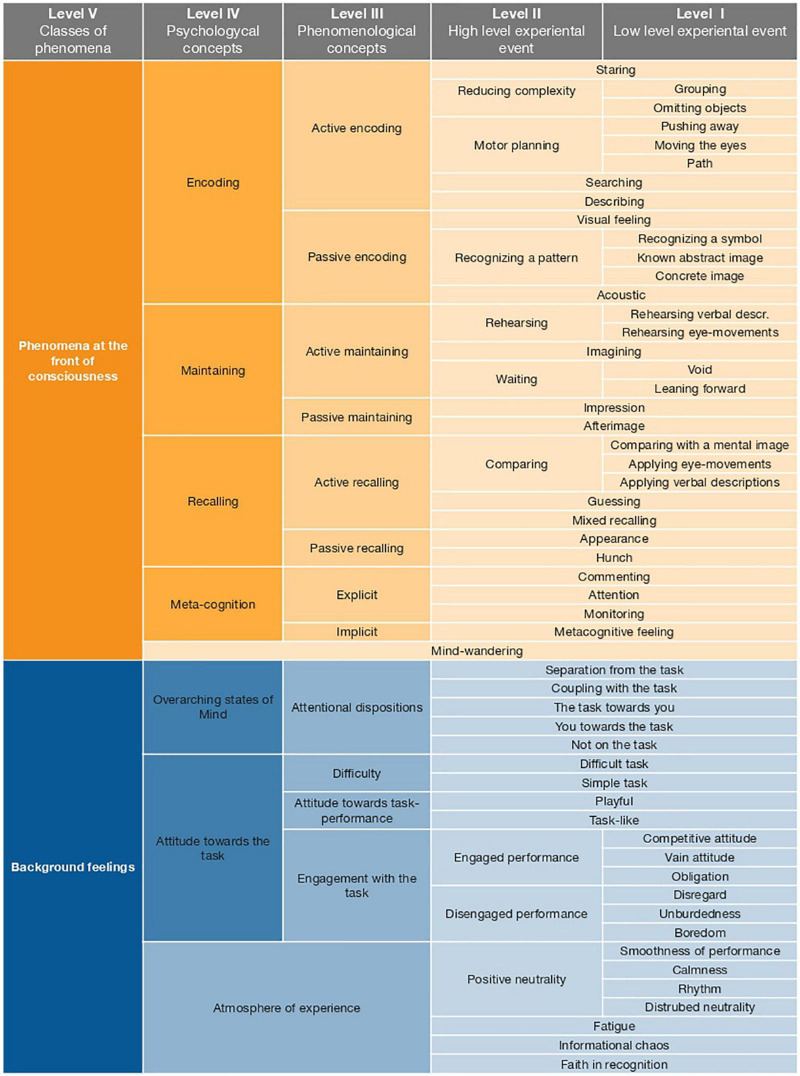
Taxonomy of experiential categories.

Interview data were analyzed according to the principles of constructivist grounded theory. The main analytical instrument in constructivist grounded theory is coding; that is, the process of assigning more general descriptive tags to sections of raw data (see section “Coding”). Codes were grouped together based on their descriptive similarities, resulting in many experiential categories. The analysis yielded a large taxonomy of experiential categories (Figure 3).

##### Coding

During the analysis, the samples from all participants and across the three tasks were grouped together. Valid samples were coded according to the principles of constructivist grounded theory (Charmaz, 2004). Our goal was to determine a system of classification that would fully describe the key aspects of experience associated with the performance of a change-detection task. Initially, our focus was on the explicit ways of how individuals solve the change detection task – what we can broadly refer to as “strategies.” However, as the data were coded inductively, other experiential categories that will be in detail presented below emerged through the process of analysis.

Following inductive coding, we employed relational coding, i.e., we constructed meaningful relationships between individual codes (Flick, 2009). Most importantly, we grouped them into higher-order categories based on descriptive similarities. Categories were constructed so that they remained stable across all participants (i.e., both the participants who received formal training in cognitive science and phenomenology, and the participants naive to those fields). Considering the large amount of phenomenal data acquired, we constructed a broad taxonomy of experiences associated with solving a change detection task (e.g., the most well-developed codes span five levels of abstraction). It is important to note here that the data we initially gathered were both broad and detailed (e.g., we gathered descriptions of how participants experienced their mental space taking shape in their consciousness). In relational coding, we narrowed our focus and constructed experiential categories that are explicitly related to engaging with a psychological task, and specifically, solving a visuo-spatial working memory task. We omitted those aspects of experience that are tangential to our research goal (e.g., experiencing the need to urinate). Finally, in relational coding, we mapped some of the categories induced from the data to extant concepts from psychological, phenomenological, and neuroscientific literature.

As mentioned in the section “Procedure,” throughout the course of the study, data acquisition and analysis were performed in parallel (Flick, 2009). Based on insights gained during the analysis, we inquired about specific questions in more detail. Further, we were able to check whether certain experiential categories we had induced in earlier interviews were valid by asking the participants about them in subsequent interviews (Charmaz, 2004). This means that in parallel to the process of data acquisition, provisional categories were constructed. The validity of these categories was then checked against subjective reports in subsequent interviews.

We observed a rich continuum of experiences associated with solving the change-detection task. As such, the relational coding yielded a complex taxonomy (Figure 3) of experiential states, spanning five levels of coding (denoted with Roman numerals). On the lowest level of coding (Level I), the categories refer to the smallest degree of abstraction from the raw transcriptions of the interview. As such, they are – for the most part – theoretically unburdened. Moving upwards through the levels of coding, the experiential categories are grouped together based on both their structural (i.e., descriptive) similarities, as well as on working memory literature. For example, we introduced differences in coding that are based on working-memory processes (i.e., target stimuli/encoding, delay/maintenance, and the presentation of the probe stimuli/recall) that a particular aspect of experience is associated with.

##### Construction of the Codebook

The taxonomy of experiential categories was organized in an annotated codebook. Constructing a codebook (sometimes referred to as the coding manual; Kalinowski et al., 2010) is a standard procedure in qualitative research, and has been productively used in empirical phenomenological studies as well (e.g., Hurlburt and Heavey, 2006; Kordeš et al., 2019; Schwartzman et al., 2020). Codebook is a text in which each experiential category is described using (a) a name; (b) a definition; (c) logical relationships with other categories (i.e., which categories are superordinate or subordinate to each other; (d) representative examples; and (e) considerations (in which specific differences between similar categories are explicated and demonstrated with examples). The codebook serves three purposes. First, it provides a way of organizing the large amount of data acquired in the study. Second, it serves as a criterion of validity (i.e., a valid taxonomy of experiential categories yields a logically consistent codebook). Finally, the codebook provides a quick way for independent researchers to familiarize themselves with our coding taxonomy. The codebook is made available in its entirety in Supplementary Material C.

##### Determining the Validity of Coding

To ensure that we have reached conceptual depth (sometimes also referred to as saturation; Saunders et al., 2018), which is the point when we have gathered enough data for constructing a theory, we used the annotated codebook approach (Nelson, 2017). This approach supplements the codebook with a saturation grid (hence annotated codebook). A saturation grid is a tabulation of interviews listed along the horizontal (in our case, we listed individual participants to make the large amount of data tractable) and the codes listed along the vertical. The occurrence of each new code is marked in the appropriate cells. When in several subsequent interviews, no new codes can be induced from the raw data, we can claim that we have reached conceptual depth. Table 1 represents a condensed saturation grid for our study. We have reached saturation after completing the interviews with Participant 14.

**TABLE 1:** Condensed saturation grid.

Code/Participant	1	2	3	4	5	6	7	8	9	10	11	12	13	14	15 – 31
New codes discovered	22	4	0	8	5	1	2	0	0	2	0	0	0	1	0

To ensure the validity of our coding process we further took two measures: intercoder verification and consensual validation. The former refers to checking whether two independent coders reached the same types of codes on the same subset of data. To this effect, the codebook was agreed on by the two principal coders, and the entirety of the gathered phenomenal data was subsequently subjected to the same codebook. Consensual validation refers to us checking with the participants themselves whether they agree that our coding process accurately reflects their experience. Consensual validation took the form of additional interviews in which the participants were given the experiential categories and asked to use them to report on their experience according to those categories. Participants were also given the opportunity to comment on whether the categories do not match their experience. No large changes were made by the participants except for remarking that the original name for the category impression should be changed from the Latin *imprimatura* to make it ‘‘less pretentious.’’^2^ In total, five sessions were conducted with participants who were perceived by the researchers as most skilled in observing and reporting on their experience.

### Epistemological Commitments

Since qualitative research, specifically phenomenology, may deviate from the positivist epistemology that is considered the standard fare in psychological research, this subsection explores the epistemological commitments that guided our study. Notably, many subjective phenomena (e.g., mind-wandering, cognitive strategies) that are commonly discussed in the literature, are phenomenologically opaque, since they are not described from the first-person perspective, but as third-person objects of inquiry (e.g., how they are operationalized in experimental research designs) (for a detailed discussion on this issue, see Varela et al., 1991; Varela, 1996; Thompson, 2007; Kordeš, 2016; Petitmengin, 2017).

Second, we adopt Hurlburt’s (2011) notion of phenomenological data being radically non-subjective. By this, it is meant that phenomenal data that are presented in this study (a) refer to phenomena that are directly present to participants’ consciousness (rather than general statements or recollections); (b) do not include participants’ opinions and generalized statements on their experience. Nonetheless, the data are not objective because the only way to access them is through interviewing the individuals who lived through a certain experience.

Where our epistemology differs from Hurlburt’s notion of radical non-subjectivity of phenomenal data is in the claim that there is an objectively correct way of describing experience. As noted above, we follow both the epistemology and methodology of constructivist grounded theory approach (Charmaz, 2004), which, broadly speaking, states that data are jointly constructed by the researchers and participants through the interviewing processes (Mills et al., 2006a,b). We attempted to increase the objectivity of the phenomenal data by: (a) conducting multiple interviewing sessions with the same participants; (b) gathering a larger amount of data than is typical for a qualitative study; and (c) engaging in the processes of consensual validation and intercoder verification.

See, however, the Section “Limitations and Future Directions” for how strategies and dispositions involved in working memory task performance might be constrained for a more objective understanding of the target phenomenology.

## Results

As mentioned in the Analysis section, we organized the qualitative data in the so-called annotated codebook. The experiential categories in the codebook span five levels of coding, with lower levels representing the smallest degree of abstraction from the raw data (i.e., amount to descriptions of experiential events as reported by the participants), whereas higher levels of coding refer to our attempts at organizing the data according to both descriptive similarities between categories, as well as insights from extant literature. The entire codebook is schematically represented in Figure 3.

At level V of coding – that is, the highest degree of abstraction from the raw data – we differentiate between phenomena at the front of consciousness and background feelings (cf. Colombetti, 2011). Phenomena at the front of consciousness refers to whatever is most present in the forefront of a participant’s experience in each moment. These aspects of experience are readily available to conscious reflection even to participants who are not trained in observing and reporting on their experience. Conversely, background feelings describe overall, integrated, and more subtle aspects of experience that were not explicitly brought into the foreground of participants’ awareness but nonetheless described how it was to be them when solving the visuo-spatial working memory task.

### Phenomena at the Front of Consciousness

Phenomena at the front of consciousness consist of five broad categories: encoding, maintaining, recalling, meta-cognition, and mind-wandering. The categories encoding, maintaining, and recalling refer to the experience of the individual stage of the working memory task. Thus, these categories could broadly be referred to as mnemonic strategies, in that, they refer to the experience of attempting to solve the visuo-spatial working memory task. Each of these three categories is further subdivided into active (i.e., an aspect of experience perceived by participants as something they do) and passive (i.e., an aspect of experience perceived by participants as something that happens to them).

#### Active Encoding

Active encoding consists of (a) staring; (b) reducing complexity; (c) motor planning; (d) searching; and (e) describing. Staring refers to the experience of simply gazing at the target stimuli, hoping that you will somehow remember them. Often, the experience of staring is accompanied by the awareness that this strategy is in vain.

Reducing complexity is an experiential category where participants find the target stimuli to be too complex for them to be able to memorize them. For participants to be able to engage with the task, they first must simplify the stimuli for themselves. This is done in two ways, grouping, and omitting. Grouping refers to participants finding a commonality in some subset of stimuli (e.g., based on shape, color, common theme), whereby they can reduce the number of stimuli that have to be remembered. Conversely, omitting is an experiential category where participants – because of the complexity of the stimuli – consciously choose to remember only a subsection of stimuli, hoping that it will be enough for them to be able to recognize whether the probe stimuli are equal to or different from the target stimuli.

Motor planning refers to an experiential category that describes instances in which participants memorize the target stimuli by memorizing how they looked over them with their gaze. This category consists of three closely related but experientially different aspects of experience. Pushing away is the experience whereby individual stimuli feel as being weighed in space and moving across them with one’s gaze is subjectively experienced as going against the apparent resistance of the stimuli. Moving the eyes is a strategy of memorizing the target stimuli whereby participants pay attention and remember the feeling in their eye muscles as they look over the stimuli. Finally, path refers to memorizing the trajectory of the center of the gaze (which may or may not be accompanied by the faint, bright line following it).

Searching is an experiential category that describes active encoding whereby participants attempt to discover a pattern in the stimuli, however, they need not actually find the pattern for this strategy to be successful. Finally, describing refers to participants tagging the target stimuli with some form of linguistic description, typically rendered in the form of inner speech.

#### Passive Encoding

Passive encoding refers to a set of experiences of the target stimuli whereby participants feel that memorization is an aspect of experience that happens to them. Passive encoding consists of three subcategories: (a) visual feeling; (b) recognizing a pattern; and (c) acoustics. Visual feeling is the experience of the overall visual atmosphere of the stimuli (e.g., blue, white, and black stimuli feeling cold).

Recognizing a pattern is an experiential category that describes the experience of some conceptual knowledge to describe the stimuli being immediately available upon perceiving them. Recognizing a pattern consists of three subcategories. First, recognizing a symbol refers to an awareness that there is some symbolic structure that can be readily related to the stimuli (e.g., white, green, and red being memorized as the colors of the Italian flag). Second, known abstract image refers to stimuli being describable through some geometrical shape (e.g., dots in the location condition outlining the shape of a star). Finally, concrete image describes instances where the stimuli elicit a mental image that incorporates some aspect of their appearance (e.g., in location condition, dots arranged in the shape of a rhombus might elicit a mental image of a kite under a blue sky).

The final category of passive encoding is acoustics. This aspect of experience refers to participants being aware of apparent sounds associated either with the appearance of an individual stage of the task or individual stimuli. Occasionally, this imaginary sound component may be sufficient for successful memorization.

#### Active Maintaining

Active maintaining describes participants willfully attempting to hold the target stimuli in their awareness during the delay period of the visuo-spatial working memory task. Active maintaining consists of three subcategories: (a) rehearsing; (b) imagining; and (c) waiting.

Rehearsing is an experiential category that refers to some aspect of experience being continuously repeated in a participant’s awareness for them not to forget the target stimuli. Rehearsing consists of two subcategories: rehearsing a verbal description and rehearsing eye-movement (in which the method of active memorizing of motor planning is rehearsed).

Imagining describes the experience of the target stimuli being recreated in one’s imagination using mental imagery (i.e., the target stimuli are maintained in the visual modality).

Finally, active maintaining consists of waiting. Interestingly, waiting was experienced as something that was actively performed by participants. It consists of two categories: void and leaning forward. The former describes an awareness of the absence of mental content. Participants are simply waiting for something to happen. Conversely, leaning forward describes the experience of performing a gesture toward the future moment (e.g., by attempting to anticipate or predict the identity of the probe stimuli).

#### Passive Maintaining

Passive maintaining is an experiential category that describes instances where the target stimuli remain in participants’ awareness seemingly of their own accord. No explicit mental acts are required for these aspects of experience. Passive maintaining consists of two subcategories: (a) impression; and (b) afterimage.

Impression is an awareness of marked space left behind by the disappearing target stimuli during the delay period. It is a spatial feeling of something having been there. Conversely, afterimage is a visual experience in the form of a rapidly deteriorating echo of the target stimuli. Afterimage is typically the opposite (i.e., contrast) color to the stimuli.

#### Active Recalling

Active recalling is the experience of participants attempting to use an explicit strategy to determine whether the probe stimuli are equal to or different from the target stimuli. Three strategies of active recalling were identified: (a) comparing; (b) guessing; and (c) mixed recalling.

Comparing is an experience in which two explicit experiences at the foreground of one’s consciousness are compared one against the other: first, there is some explicitly present memory of the target stimuli, and second, the visual presence of the probe stimuli. Three common experiences of comparing were observed: comparing with a mental image (i.e., a visually present memory of the target stimuli), applying eye-movements, and applying verbal descriptions.

Guessing refers to participants not having an explicitly present answer to the probe. Thus, they opt for picking an answer at random.

Finally, mixed recalling refers to a highly specific experiential dynamic during the probe stimuli. Namely, participants first passively become aware of whether the probe stimuli are equal to or different from the target stimuli (see below for passive recalling). In absence of an explicit strategy, participants feel unsure in their response. Thus, they use an additional explicit strategy to make sure that their initial feeling is correct.

#### Passive Recalling

Passive recalling is an experiential category that describes instances in which the knowledge about whether the probe stimuli are equal to or different from target stimuli occurs to the participants. Passive recalling contains two subcategories: (a) appearance; and (b) hunch. The two subcategories differ primarily in their location within one’s experiential field, and the level of certainty. Appearance is an experiential category that describes the answer being present immediately in the perception of the probe stimuli. Appearance is accompanied by a sense of absolute certainty that the answer is correct. Second, hunch is a bodily felt likelihood regarding the answer to the probe stimuli (cf. gut feeling). Unlike appearance, hunch is subject to doubt.

#### Meta-Cognition

During the performance of the visuo-spatial working memory task, participants commonly reflect on their performance. We coded such experiences as meta-cognition. Subcategories of meta-cognition are divided along the lines of how saliently they are present in participants’ awareness: (a) explicit; and (b) implicit. Three explicit aspects of meta-cognitive phenomenology were observed. First, commenting refers to an ongoing monolog or a dialogue, rendered in inner speech, providing feedback on what participants are doing and how well they are performing at the visuo-spatial working memory task. Second, attention refers to a sense of self-assuredness associated with the knowledge that they are paying close attention to the task. Since they are mindful of the task, the logic goes, it is unlikely that they missed something. Finally, monitoring refers to participants assuming a variety of observational perspectives of their experience of the task, both to make sure that there are no experiences that slip past them, and to be able to report on their experience.

The only implicit aspect of meta-cognitive phenomenology is meta-cognitive feeling. This experience is closely related to the experience of positive neutrality (described in detail below). Meta-cognitive feeling refers to whether participants suddenly become aware of some disturbance in their field of experience, signifying that they have made a mistake.

#### Mind-Wandering

The final phenomenon at the front of consciousness refers to mind-wandering. These are instances in which participants’ attention moves away from the task toward other objects and/or mental activities, unrelated to the visuo-spatial working memory task, that they construct for themselves.

It is important to emphasize that many phenomena at the front of consciousness occur simultaneously or overlap within a single experiential episode. Phenomena at the front of consciousness are of particular interest to the theoretical construct of working memory, specifically, what kind of cognitive strategies, as accessible to conscious reflection, participants deploy to successfully solve the task. A detailed description of the categories and a quantitative analysis of experiential categories will be presented elsewhere. In the remainder of this paper, we pay particular attention to the second Level-V experiential category: background feelings.

### Background Feelings

During data acquisition and analysis, we observed a huge variability in non-strategic experiences during the task-performance. While aspects of experience related to strategic performance of a visuo-spatial working memory task (outlined above) were - in a manner of speaking - artificial (being tied to specific stages of the working memory task), we observed an aspect of experience that provides a much more holistic description of how it feels to solve a visuo-spatial working memory task. These aspects of experience represent the second level-V category: Background feelings. Background feelings constitute aspects of experience that are not placed in the focus of participants’ awareness, but nonetheless play a major role in the description of how it is to be someone in each moment. They are represented by several subtle attentional modulations and bodily feelings.

Notably, while phenomena at the front of consciousness are easily accessible even to participants who are not trained in observing one’s experience, background feelings are not as readily apparent in conscious reflection. Background feelings include the following level IV subcategories: (a) attentional dispositions, (b) atmosphere of experience, and (c) attitude toward the task. These aspects of experience will be explored in detail in subsequent sections.

#### Attentional Dispositions

Attentional dispositions describe different attitudes we can take within our attention toward a specific object of our awareness. In turn, this attitude influences how we experience the object itself (cf. Petitmengin et al., 2009; Kordeš et al., 2019). In this study, we observed five different experiential categories that we consider to constitute attentional dispositions we may take toward a visual-spatial working memory task: separation from the task, coupling with the task, you toward the task, the task toward you, and not on the task. Separation from the task refers to an attitude of distance toward the change-detection task. Rather than being experienced as something that the participant has a causal effect over, the task is seen more as a video being passively observed. As Participant 27 reports:

“I wasn’t really with the task. I was solving it and it was important to me to solve it well, but I was also really aware of the room and you sitting behind me […] What happened was that there was this sense of whole that was left over from the previous example, and then I didn’t know whether I’m comparing it to this example or the one that came before.” (VR.WM.1.27-04-O-01)

Conversely, coupling with the task is an experiential category that describes a spatially felt connection between the participant and the task at hand. This connection can be implicit and can appear simply as a loss of awareness of the research setting, the researcher, and the environment. Conversely, it can be very apparent, experienced as a sense of an enclosed, private space between the participant and the psychological task:

“It is a kind of bubble. My attention is concentrated here at the front, at the task. This bubble is essentially a very pleasant kind of attention. I really like this focus. And my surroundings disappear. And I’m not aware of you either. There wasn’t even an awareness of where I’m sitting. All that existed was this task. And I felt warm about that. This warmth was for me and for the way I do the task. It wasn’t warm like the sun, but like a warmth in my thoughts.” (VR.WM.1.24-01-P-02)

Alternatively, participants can approach the task by performing several mental gestures upon it (you toward the task). This experience may be so strong that it appears as a sense of direction moving from the participant toward the task. Consider the following example where the performance of mental gestures is explicitly reported on:

“The task seemed more difficult than before. Before, it was enough for me to just have the impression of the image, whereas now I had to keep more of my focus on the task. I had to push away the things unrelated to the task. I performed movements with my gaze from object to object. I was jumping across them.” (VR.WM.1.10-01-C-06)

Participants may also assume the attentional disposition of the task toward you. This experiential category refers to the participants appreciating the stimuli such as they are, without performing mental gestures upon it; that is, the category constitutes a receptive attitude toward the task. Consider the following report:

“I just let the dots happen […] I trusted myself that I will be able to know whether the next stimulus is correct or not. […] It was as if I shut the task down. I was looking at the [fixation cross] and it was a little bit like meditating. I didn’t have to think of the dots or anything.” (VR.WM.1.15-02-P-04)

The final attentional disposition that we have observed within this study is not on the task, which refers to situations where participants attended to something other than the visual-spatial working memory task. This attentional disposition, however, is quite rare, as it was difficult to come across cases where the task did not in any way enter participants’ awareness without the research context breaking down (i.e., without them stopping to solve the task).

#### Attitude Toward the Task

The second subcategory of background feelings are attitudes toward the task. These are background feelings within which the participants implicitly interpret the experimental paradigm in one way or another. In this context, we use the term “interpretation” to refer to what the participants make of the whole research setting. Attitudes toward the task contain three major ways of interpreting the change-detection task: (a) difficulty, (b) attitude toward task-performance, and (c) engagement with the task.

Difficulty is an attitude toward the task in which the participants interpret the task either as easy or difficult. Importantly, it is apparent that the difficulty of the task is not a property of an observer-independent, objective world, but it amounts to an attitude that participants take.

Attitude toward task-performance is an experiential category that refers to whether the visuo-spatial working memory task is appraised by participants as playful or task-like. Consider the following example, when the task appears playful:

“The task seems more dynamic somehow. It was easier and more fun. I didn’t know where the objects will appear and so there was always a little surprise when they showed up. I didn’t have an anticipatory feeling about where they will appear. It was fun.” (VR.WM.1.06-02-P-01)

Conversely, consider the following example when the experimental setup appears task-like:

“I began thinking about how I don’t like the exercise. The tails of the objects bothered me. They caused these bad feelings inside of me. These were more mental than bodily. It wasn’t as if I was in pain or under stress. It was more of a preference. As if I didn’t have these tails on the objects.” (VR.WM.1.25-03-O-02)

Engagement with the task is an experiential category that describes the level of enthusiasm and zeal with which the participants approach it. In other words, it is a question of whether the participants approach the task as if it is important and something they must do well, or whether they approach it as something irrelevant, something that is simply there. Engagement with the task can be subdivided into engaged performance (where it is important to participants that they do well on the task), and disengaged performance (where the task is no longer at the center of their awareness). The former contains the following subcategories: (a) competitive attitude; (b) vain attitude; and (c) obligation. Disengaged performance contains the following subcategories: (a) disregard; (b) unburdedness; and (c) boredom.

#### The Atmosphere of Experience

The final subcategory of background feelings is the atmosphere of experience. It is a background feeling that describes the frame of the participant’s experience. It refers to those aspects of experience that do not carry with them a specific content, but rather color whatever is at the center of the participant’s experience. It contains four subcategories: (a) Positive neutrality, (b) fatigue, (c) informational chaos, and (d) faith in recognition. Fatigue is an experiential category that is to be understood in the trivial sense of the word – the experience of being tired.

Faith in recognition refers to an experience where despite not having an explicit mnemonic strategy, the participants feel that they will be able to successfully solve the change-detection task:

“I know I can do it. It’s just that I don’t know how exactly I know. When I look at the memory stimulus, it just seems like I will be able to remember it.” (VR.WM.1.07-01-C-02)

Informational chaos is a particular atmosphere of experience where the visual-spatial working memory task seems so complex as to be impossible to solve. It seems impossible to memorize the memory stimuli on account of how complex they seem. Participants commonly experience this complexity as the absence of any stable point at which to direct their attention to start memorizing the stimulus. It is an exceedingly unpleasant experience, and it is accompanied by feelings of anxiety and fear (cf. Kordeš, 2019). Over the course of the validation interview, Participant 22 reported that whenever she was solving the orientation condition, she was constantly anxious:

“The memory stimulus appeared on the screen, and I immediately realized that I do not have the time to encompass the whole image with my eyes, let alone memorize it. I was moving my eyes around. I was panicking. I was trying to construct a memory, a pattern that would be sensible enough to be memorable. When the test stimulus actually happened, I had no explicit memory representation. There was no sense of how familiar the second stimulus was. So, I guessed the answer.” (VR.WM.1.22-01-O-01)

Consider the further report from Participant 01 about the negative feelings incurred by the task:

“It was terrible! I felt really unpleasant because I was fixated on having to get all of them right. I can feel the tension in my body, and I feel stressed. I have a feeling that this is how tasks are supposed to be performed. This is not research. It’s an exam. It’s something I have to get right. I know I have to get it right. This was constantly in the front of my awareness. And it took a lot of attention for me to be able to solve the task. I couldn’t find any reference point that would help me remember it. I just waited for [the stimulus] to happen and try to remember its impression somehow. I was never sure about the answer. I always doubted the answer.” (VR.WM.1.01-04-P-01)

The transcript of the interview does not do justice to the participant’s distress. In research memos, we noted that her face became flushed, she was visibly uncomfortable, and bordering on tears. Participant 27 similarly reports:

“It seemed to me like it should be fun, but it isn’t. Now, it’s stressful. I am using only about 20% to pay attention to it and I kind of don’t care about it. I started to think about the task and now it’s no longer as fun. And everything began going by so quickly. And so, I started thinking, why should I even bother if everything is going by so quickly. And then I ask myself, what’s wrong with me? Am I dumb? And so, I just refuse to answer.” (VR.WM.1.27-04-O-02)

One of the key aspects of informational chaos is the experience of the lack of stability, that is, the sense that there is no part of the visual experience that the participants could anchor their attention to in order to start remembering. Participant 25 reports:

“I ran out of focus, but not because I was wrong with the previous one. It was simply no longer clear what I have to do. I don’t understand what is happening on the screen. I don’t know what to do. It was really, really uncomfortable. This panic was much worse than if I was just wrong. I almost started crying. I felt totally powerless. I felt this tension that was rising from my chest to my clavicles. I felt that I have an increased heart rate. I didn’t know what to do. There was a confusion in my mind. I didn’t know what to focus on. There was a lot of pondering, but actually, I didn’t really have any thoughts. It was all mostly bodily experience. I couldn’t stop it. I couldn’t make a decision about what to do. This panic was the only thing I was really experiencing. I wasn’t solving the task. I was just randomly clicking. Because I knew I had to. And there was this idea of a record of everything that I don’t click on.” (VR.WM.1.25-01-O-02)

She goes on:

“I began thinking about how I don’t like the exercise. The tails of the objects bothered me. They caused these bad feelings inside of me. These were more mental than bodily. It wasn’t as if I was in pain or under stress. It was more of a preference. As if I didn’t have these tails on the objects.” (VR.WM.1.25-03-O-02)

Importantly, informational chaos is associated with a particular attitude toward the task, namely the sense of obligation. As Participant 22 reports:

“I had a feeling of a total lack of control. I knew that I could not answer these questions. I randomly clicked. And when I finally gave into it, I had no thoughts about it, even though it encompassed the better part of my experience.” (VR.WM.1.22-01-0-01)

The performative dimension of informational chaos is apparent: the participants are no longer solving the task, but are rather clicking at random, because of an experience of obligation. Rather than asking the researcher for the study to end – which they are told is a possibility at the beginning of the study – the participants continue to pretend to perform the working memory task.

Positive neutrality is an atmosphere of experience in which the participants are aware of the absence of any kind of disturbance. It is the sense of the smooth running of events. This smoothness; however, is not experienced positively in and of itself: it is a noticeably neutral aspect of experience. Because the participants are aware of nothing being wrong, this neutrality is experienced as something good, as something mildly positive. Positive neutrality may be brought into one’s awareness four interrelated and connected ways: (a) Smoothness of performance; (b) calmness; and (c) rhythm.

Smoothness of performance is an atmosphere of experience in which the participants are aware of positive neutrality at their own task performance. The task – and its performance – run smoothly, without any interruptions. This smoothness in and of itself is not pleasant. The absence of disturbances is the aspect of experience that is perceived as positive. Consider the following:

“Everything was related to the rhythm. It created this flow. This rhythm was silent, discrete, a staccato. I moved my eyes according to where I was expecting the dot to appear. When it didn’t appear, I got a sense of dissonance. It went outside of the rhythm. It’s a very mental experience, yes, but it is not reasoning. It wasn’t as if I said ‘Aha, it went out of rhythm, therefore, it does not fit.’ It was a more subtle feeling. Like an aha moment. The dot was not here, so it must be there. When the dot was in an unexpected place, the rhythm broke down. And so did any image that I had in my mind.” (VR.WM.1.19-02-P-02)

The experience of rhythm is, by and large, tied to the appearance of stimuli themselves. This experiential category refers to an embodied (and sometimes even auditory, when accompanied by the experience of acoustics) element of experience. Participants report that rhythm structures their time and that task-performance in consequence becomes more relaxed and predictable. Rhythm can be embodied and is commonly tied to the feeling of heart rate. In a similar manner to the smoothness of performance, rhythm can break down in the case of a mistake or a disturbance in an experiential field. This leads to the tempo stopping. Insofar as the disturbance does not occur, the rhythm encompasses the final key press as well:

“A rhythm of performing the task established itself. At the end, I answer completely automatically. If there’s a mismatch, this way of solving the task stops.” (VR.WM.1.17-04-O-02)

Smoothness of performance is an experience that is structurally like rhythm from the point of view that once it is present, it appears as the default state of the experiential landscape. Both aspects of experience may be cut short by a disturbance. The difference is that smoothness of performance is, by and large, unconscious. Rhythm, on the other hand, clearly constitutes a presence. Consider the following example:

“I said to myself, ‘one, two, three, four.’ This happened in the same rhythm as the dots appeared in. these verbalizations were sounds that happened in my own voice. The rhythm helped me concentrate. It gave structure to my sense of time. It wasn’t intended to be a dancing rhythm but it reminded me a lot about dance where you have to count […] I’m the author of these words. I willfully produce them. When they first appeared, I didn’t willfully create them. It wasn’t my strategy at first. But then I noticed that I’m doing it and that it feels good. What felt good was the structure. I latched on to that and started actively doing it.” (VR.WM.1.04-02-P-01)

Finally, calmness is an atmosphere of experience whereby the participants feel pleasant in the absence of disturbances. Calmness itself has no object. It describes the general way of existing in a particular moment.

## Discussion

In the present study, we investigated the experience during solving a visuo-spatial working memory task. We used the change-detection task, a standard instrument for studying working memory, and gathered first-person data with a combination of experience sampling and an in-depth phenomenological interview. This resulted in a large array of data depicting the experience of participants solving the visuo-spatial working memory task. During the study, we observed that the experiential landscapes associated with visuo-spatial working memory task-performance are extremely rich, both in terms of mental acts that participants use to attempt to “solve” the task, as well as in terms of depth of experience; that is, overall experiential states accompanying (and often probably determining) the task-performance.

Our qualitative analysis separated the gathered phenomenal data into two major classes of experience. First, there is an experiential dimension, coded as phenomena at the front of consciousness. This category describes aspects of experience that occupy the center of participants’ awareness and are readily accessible to consciousness reflection even in absence of training in how to observe and report on experience. Phenomena at the front of consciousness consist of various strategies of solving the visuo-spatial working memory task, together with meta-cognitive experiences, and mind-wandering.

Second, during the analysis, an experiential dimension that is harder to access in conscious reflection as it comprises the overall sense of experience (e.g., bodily feelings, emotional atmosphere, mood) was consistently detected. We coded this experiential dimension as background feelings. Most of the present article, including the discussion, is dedicated to background feelings. The study of background feelings poses significant methodological challenges, but – we suspect – could play an important role in understanding and interpreting the results of visuo-spatial working memory task-performance, as well as working memory more broadly.

### Comparison to Existing Ideas

In the present subsection, we tie the experiential categories observed in this study with extant ideas from mind sciences. The identified categories that belong to phenomena at the front of consciousness map well onto extant constructs from the domains of cognitive strategies, metacognition, and perception. The broad distinction between active and passive experiential categories fits the discussion on agency in philosophy of mind (Gallagher, 2012). A number of categories were observed that might be summarized as representational modalities (imagining, motor planning, describing) that correspond with the basic types of experiences identified via descriptive experience sampling (inner seeing, inner speech, feeling) (Heavey and Hurlburt, 2006). Reducing complexity can be linked to chunking (Miller, 1956). The complex phenomenological dynamic seen during the delay period (i.e., the present moment can be experienced as absence of experience or an anticipation of the future) can be linked to experimental results that suggest that encoding and recall are more akin to different styles of solving the task than separate memory mechanisms (Speer et al., 2003). Finally, the embodied dimension of hunch suggests that it may be integrated into the somatic marker hypothesis, a theory that suggests intuition is the statistical awareness of the probability of some event, reflected in the personal level as a specific array of bodily feelings (Damasio, 1996). A more detailed examination of phenomena at the front of consciousness will be presented in a follow-up publication.

While phenomena at the front of consciousness amount to well-known constructs within the sciences of the mind, until recently, background feelings received little attention within psychological literature (although see Tsuchiya and Koch, 2016; de Haan, 2020). Yet, we believe that understanding this aspect of experience may be as important as understanding the strategies participants use to solve a visuo-spatial working memory task for disentangling working memory task performance.

By way of example, let us examine one of the categories that we identified in this study: positive neutrality, a subcategory of the background feeling atmosphere of experience. Positive neutrality refers to a subtle awareness of an absence of change. Participants report solving the visuo-spatial working memory task guided by the awareness of an absence of disturbances (e.g., wrong answers). This lack of disturbances itself is felt as neutral. However, since - in the context of visuo-spatial working memory task performance – it signifies that the participants have not detected any blunders, it is appraised as slightly positive. In such cases, positive neutrality also reflects the repeating, rhythmic pattern of trials occurring at a predictable rate. Only when something goes wrong (e.g., the pace at which they are scanning the stimuli, wrong answer, end of task, etc.) is this positive neutrality disturbed.

These experiential dynamics mirrors the notion of surprise in the so-called free energy principle theories of cognition (Friston, 2009, 2010). According to this theory, living organisms work to minimize their internal disorder (entropy). They create a barrier between themselves and the outside world, wherein inside of this boundary, they maintain their own homeostasis (Kirchhoff et al., 2018; Demekas et al., 2020). A surprising event in this framework, is an event that was not predicted by the organism (Schwartenbeck et al., 2013). When applied to cognition, free energy principle is related to predictive processing, the notion that to minimize the computational complexity of incoming sensory information, organisms predict the likeliest situation and then only process surprising stimuli (i.e., stimuli that they were unable to predict) (Seth et al., 2012; Seth, 2014; Clark, 2016).

The properties of some of the background feelings (e.g., the feeling of positive neutrality) are remarkably like phenomenological properties associated with or allowing for the experience of surprise (Bitbol, 2019). Integrating these ideas with our own, we posit that positive neutrality is the stable state of successful prediction. When positive neutrality is disturbed, the experiencing individual is prompted to acknowledge change.

Second, the phenomenon of attentional dispositions has been commonly reported in empirical phenomenological literature. Originally, it was reported in the study on how we experience attending to sound (Petitmengin et al., 2009). In that study, attentional dispositions refer to the changes in our experience of the source of sound when we attend to it with different attitudes (e.g., as a physical sensation in our ears, as a location in space, as an acoustic experience with volume, pitch, and timbre). The findings were corroborated in a study on the experience of meditation (Kordeš et al., 2019). Specifically, participants reported experiencing pain (e.g., in their legs or back when meditating). Pain could then be attended to with different attitudes, which, in turn, altered the intensity of noxious sensations.

Some attentional dispositions bear resemblance to what Oblak et al. (2021) refer to as affective resonance, a property of objects present in one’s environment that they can jointly form higher-order systems (see also Thompson, 2007; Kyselo and Tschacher, 2014). This description corresponds to the disposition of coupling with the task, where participants report feeling that the task-performance is not something that they do themselves, but they do it in concert with the computer. Conversely, the disposition separation from the task is descriptively similar to the phenomenological notion of detunement. Detunement refers to an inability of accessing socially shared space in depression (Fuchs, 2005, 2007), schizophrenia (Krueger and Aiken, 2016; Silverstein et al., 2017), and anxiety (Trigg, 2017). It may be that part of why individuals with specific psychopathologies underperform on working memory tasks (Christopher and MacDonald, 2005; Rose and Ebmeier, 2006; Forbes et al., 2009; Moran, 2016) is because phenomenologically, the task are not salient enough in their awareness for them to be able to attentionally engage with them.

In line with Hurlburt et al.’s (2016) findings, some insights, associated with background feelings, indicate that the experience of visuo-spatial working memory task-performance are quite removed from day-to-day experiences. Participant 22, for example, reports feelings (coded as informational chaos) that are familiar to her only from other times when she was solving similar psychological tasks:

“I remember solving a similar task for [one of the professors]. I was stuck in some lab, I was a little bit afraid, and on top of that, there was this bombardment of stimuli that was totally overwhelming. I really don’t recognize this feeling from situations other than stuff like this.” (VR.WM.1.22-01-O-01)

Gathered phenomenal data raise questions about the validity of studies operating under the assumption that psychological tasks elicit only the target phenomenon without checking participant’s experience of said task (cf. Hurlburt, 2011, Chapter 21). Our study has not gathered sufficient data to conclusively reach this conclusion. Additional investigations of experience underlying performance of visuo-spatial working memory tasks, as well as more ecological (e.g., ethnographic) research of day-to-day experience associated with visuo-spatial working memory are needed. For example, recent qualitative studies into the experience of working memory tasks have demonstrated the presence of various emotional states (bearing similarities to our experiential category informational chaos), primarily dealing with the social expectations and that are associated with performance accuracy (Laybourn et al., 2022). Thus, this study points to some as of yet unanswered questions in the broader field of working memory research.

### Toward a Neurophenomenology of Working Memory

Positive neutrality and attentional dispositions are not the only example of an experiential category where we observed background feelings playing an important (and perhaps essential) role in task-performance. The entire group of experiential categories we coded as the atmosphere of experience was – according to subjective estimates of participants – important when engaging with the visuo-spatial working memory task. Our results seem to coincide with some recent ideas in cognitive science indicating the importance of understanding the general, background atmospheres. One of such ideas is the theory of overarching states of mind (Herz et al., 2020). It proposes that, on the level of neural dynamics, we can observe integrated states that coordinate all other aspects of cognition, such as perception, emotions, thinking, as well as embodied behavior. It is not unreasonable to hypothesize that such overarching states of mind can be detected on the personal level of description as a specific class of background feelings.

We hope to have shown that aspects of experience that are not so easily identifiable form a significant part of an individual’s experiential landscape. Detection of such aspects of experience requires participants who, at the very least, are interested in exploring their own experience, but preferably are also trained in observing and reporting their experience.

Our results correspond to various observations by many within the field of first-person research (Petitmengin, 2006; Hurlburt, 2011; Kordeš, 2016), thereby indicating the necessity of re-examining the traditional roles of researcher and participant (cf. Orne, 1962) in the domain of researching experience. Since the participant is the only person with the access to experience under investigation, then, her process or reflection constitutes the principal instrument of inquiry. If this instrument is inadequate, then, no subsequent step (e.g., interview, analysis, etc.), no matter how refined, can improve the validity of a study. Thus, it is necessary to consider the people whose experience we are investigating not as passive subjects from whom data can be objectively extracted, but rather as equal partners in the research process – as co-researchers (cf. Kordeš and Klauser, 2016).

The acquired first-person data allow us to put forward a conjecture. It may be that the aspects of experience detected in this study (and coded as background feelings) determine our behavior to a greater extent than the readily accessible phenomena at the forefront of consciousness. It may be that a larger part of visuo-spatial working memory task-performance occurs below the threshold of conscious awareness (cf. Soto et al., 2011; Dutta et al., 2014; Soto and Silvanto, 2014). Explicit, conscious attempts at taking control over the task-performance (i.e., assuming a strategic attitude) may therefore only serve to interfere with task-performance. In other words, attempts at explicit performance stop subconscious task-performance (cf. Lyubomirsky et al., 1999).

However, when talking about a large part of task-performance taking place below the threshold of conscious awareness, we are referring to an everyday, and, for the most part, inaccurate awareness. Is it possible that a more precise insight into the content of consciousness allows this threshold to be raised? What if a more detailed reflection can be used to detect traces of cognitive processes taking part in visuo-spatial working memory task-performance? We believe that this is the case and that these traces can be observed, but only by participants, skilled enough to be able to access background feelings (cf. Petitmengin et al., 2007). Thus, it is essential for the validity of studies attempting to provide an in-depth account of first-person data to use participants who are skilled and trained in observing and reporting on their experience (cf. Miyahara et al., 2020).

In future research, we aim at replicating the study presented in this paper, however, recruiting only participants trained in observing and reporting on their experience. Additionally, we hold that using trained participants may be beneficial for future fMRI and neurophenomenological studies (cf. Lutz et al., 2002; Fernyhough et al., 2018). As demonstrated by Hurlburt et al. (2016), validity of studies assuming that the experimental paradigm constructs the phenomenon of inquiry, without verifying it on the level of experience, is questionable (Hurlburt, 2011).

## Limitations and Future Directions

Although in addition to first-person reports we also collected behavioral responses (i.e., performance accuracy and reaction times), this paper omitted quantitative analyses of the data. There are two reasons for this. First, as the aim of this study – i.e., exploring the breadth of experiences evoked by and accompanying performance of a visuo-spatial working memory task – was complex as it is, we wanted to avoid addressing additional research questions related to behavioral performance. For example, it would be interesting to explore the relationship between the identified experiential categories, behavioral measures (reaction times and performance accuracy) and task modality, however, this aspect of the results will be presented in the follow-up paper.

Second, the study followed the golden standard for estimating when enough qualitative data were gathered (see section “Determining the Validity of Coding”). However, despite gathering a large amount of data for a qualitative study, there are too few acquired samples to be able to draw any statistical conclusions.

Future studies should be designed specifically to constrain first-person data (i.e., reports on strategies and dispositions involved in solving a visuo-spatial working memory task), behavioral (performance accuracy and reaction times), and psychometric measures (e.g., personality questionnaires, clinical scales). Such a research design would necessitate a development of a custom-made framework for reporting on the phenomenology of solving a visuo-spatial working memory task in real time. A precedence for such an approach can be seen in the studies on the experience of inner speech. These began with sampling of overall experience (Heavey and Hurlburt, 2006), moving on to detailed account of inner speech (Hurlburt et al., 2013), before developing phenomenology-inspired questionnaires (Alderson-Day et al., 2018) and neuroimaging studies (Hurlburt et al., 2016).

## Conclusion

In this paper, we presented an empirical phenomenological study of what individuals experience during solving a visuo-spatial working memory task (specifically, the change-detection task). Using a combination of experience sampling and an in-depth interview we gathered a large amount of experiential data. The data were analyzed according to the principles of constructivist grounded theory. The resulting experiential data demonstrate the wealth of different experiences associated with solving a working memory task. Some of them are easy-to-detect and could be productively used to further approaches within experimental cognitive psychology. Others amount to overarching, integrated states of consciousness within a given moment. These require further neurophenomenological investigation, as well as reconsidering the social dynamics between researchers and participants. Gathered phenomenal data raise questions about the validity of studies operating under the assumption that psychological tasks elicit only the target phenomenon without checking participant’s experience of said task.

## Data Availability Statement

The original contributions presented in the study are publicly available. This data can be found here: https://osf.io/mkp8b/.

## Ethics Statement

The studies involving human participants were reviewed and approved by the Internal Review Board at the University Psychiatric Clinic Ljubljana, University of Ljubljana. The patients/participants provided their written informed consent to participate in this study.

## Author Contributions

GR, ASO, and UK: conceptualization. AO, GR, ASO, and UK: methodology, and writing – review and editing. GR and ASO: software. AO and UK: formal analysis. AO: investigation and writing – original draft and project administration. AO and ASO: visualization. UK and GR: supervision and funding acquisition. All authors: contributed to the article and approved the submitted version.

## Conflict of Interest

GR consults for and holds equity in Neumora Therapeutics. The remaining authors declare that the research was conducted in the absence of any commercial or financial relationships that could be construed as a potential conflict of interest.

## Publisher’s Note

All claims expressed in this article are solely those of the authors and do not necessarily represent those of their affiliated organizations, or those of the publisher, the editors and the reviewers. Any product that may be evaluated in this article, or claim that may be made by its manufacturer, is not guaranteed or endorsed by the publisher.

## Footnotes

1 First-person research is an evolving discipline in the context of cognitive science. Ongoing discussions regarding the methodological recommendations and guidelines are being conducted within the community. It is important to note that the present study was conducted between April and November 2018. At such time, a completely bottom–up approach was favored by the researchers in the first-person research community (for an epistemological discussion on the role of theory in first-person research, see Kordeš, 2016). Thus, the data acquired in this study were not constrained by questionnaires inquiring into, for instance, personality structure or the expression of mood disorders. Since examples of studies constraining these different types of instruments were published in the intervening years (Madeiros et al., 2021a) as well as discussions resolving the relevant epistemological issues (Madeiros et al., 2021b; Oblak, 2021b), it is now possible to put forward research design addressing these relationships.

2 It is to be noted that at the time of conducting this study, no procedures tailor-made for consensual validation of phenomenal data were available. The consensual validation presented in this study amounted to a first stage of developing a validation procedure for phenomenal data (for the development of the validation procedure, see Oblak, 2021a).
