# Supplementary material for: What Individuals Experience During Visuo-Spatial Working Memory Task Performance: An Exploratory Phenomenological Study
Source: Front Psychol. 2022 May 18;13:811712. doi: 10.3389/fpsyg.2022.811712 (PMC9159378; doi:10.3389/fpsyg.2022.811712)
Supplement: Supplementary file 1 [file Data_Sheet_1.pdf]

Phenomenology of Visuo-spatial Working Memory Task Performance

Supplementary Materials A: Excerpts from the Interviews

1 S01-02-P-01

Udeleženka se je zmedla in ni vedela, kateri je spominski in kateri je testni dražljaj;

Objekti sami so se ji zdeli dolgočasni in poskušala si jih je zapomniti prostorsko. Fiksacijski križ je uporabila za referenčno točko in potem si je poskušala glede na to zapomniti, kje so preostali objekti;

Objektom je sledila z očmi;

Zapomnila si jih je tako, da je zanje ustvarila nek prostor. Ta prostor je ustvarila iz beline ekrana. Zmislila si ga je v glavi. Ustvarila je nek občutek tega prostora in to ni bilo nekaj vizualnega. Ta občutek se ji je kazal tako, da vsakič ko se je neka pika prikazala, je imela občutek, da se nahaja v nekem prostoru, kjer se lahko to premika. Te štiri pike je doživljala kot eno piko, ki se premika. Izginjanje pike je doživljala kot nek takojšen trenutek, ni bilo nobenega bledenja;

2 S01-02-P-02

Pavza: obdobja med dražljaji sploh ni zaznavala; za pikami je opazila paslike kot bele negative, kot da za njimi ostane nekaj, kar je malce bolj svetlo. Ne opazi, ali bledijo, ker prehitro pride testni dražljaj. To je nekaj, kar se ji dogaja. Paslike se pojavljajo na ekranu;

Ta pika se premika in se pojavlja in izginja, kakor da obstaja potem pa se pogrezne in pride nekje ven, kakor riba, ki plava;

Pavza: doživljala je pričakovanje testnega dražljaja, med katerim se ni zavedala sobe. Cel čas, ko je zbrana in rešuje nalogo, se ne zaveda sobe, niti svojega telesa.

Testni dražljaj: ni ji ostala neka slika, ni mogla zares ohraniti zaporedja pik. Ni nekega momenta, ne more si dlje ogledovati neke reprezentacije, zato se mora zanašati na nek občutek.

Odgovor: Gre za občutek, za prepričanost, ki se ji kaže v nekem razpoloženju. Če ima občutek, da prav ve, dobi neko potrditev, nek notranji občutek. Če ji pa ne gre, pa zelo hitro izgubi zanimanje.

Meta-kognicija: zdi se ji, da je obstajal nek tak občutek vse dokler ni potrdila svojega odgovora. Ve, da ve in ve, da mora držati to vedenje, da mora pazit, da ji ne pade koncentracija.

Držanje vedenja: vse okrog nje se ugasne, ne zaznava več sobe, ne zvokov, ne vonja, niti svojega telesa. V ospredju njene zavesti je občutek tega dražljaja.

3 S01-04-O-01

Ful je še hitro mimo; za trenutek ji je padla koncentracija, ker je ugotovila, da

Zahteve naloge: Najprej je bila pripravljena na reševanje, ko pa je ugotovila, katera naloga je (to je bil nek spomin na podlagi pretekle izkušnja), se je spomnila, da ji ni všeč. Njena pozornost se je povečala, ko se je začela dogajati naloga. Ima močan občutek fokusa. Preden je začela reševati nalogo, je bilo vse bolj razpršeno, zdaj pa je bilo tako kot da se nekaj zlije skupaj: kakor da bi se vsa razpršenost zlila skupaj. Je kot da bi držala pozornost v mejah pred sabo in zdaj je samo ta naloga. To terja nek vložek energije. Prej vse malo plava – kar je, je in kar pride, delaš s tem – zdaj pa je naloga z navodili (na nek način ima obbčutek varnosti in nadzora nad tem, ker je že delala to in ker pozna navodila). Sobe se ne zaveda, ker je potrebna ta energija. Sicer je ta pozornost lahkotna, ker naloga ni kompleksna.

Zalotila se je, da razmišlja o tem, ali je prav in da ji je pomembno, da dobro rešuje nalogo. Te misli so bile nek občutek, obenem pa je čutila napetost v prsnem košu in mogoče je začela plitko dihat.

Meta-kognicija: imela je nek občutek, ampak bolj zaradi tega, da lahko drži pozornost. Ni ji begala. Le pri zadnjem primeru je opazila, da ji je pozornost malo ušla. Vseeno pa je reševala.

Zapomni si, da je nek občutek, pri katerem si naslika usmerjenost repkov. Zaznala je, da med pavzo, ko je samo fiksacijski križ, da gre še enkrat skozi;

Da si naslika usmerjenost: ni, da to vizualno vidi, ampak je občutek tega, kar je videla. Ni prostorski občutek. Če bi umaknila pogled, ne bi mogla skonstruirat tega. Podobno je temu, da bi se jo dotaknil in umaknil dotik, pa bi ostal tam nek občutek (*mindprint*). Počuti se, da ona to počne → če bi umaknila pozornost, tega ne bi več mogla skonstruirat;

Pavza: ni prostora za dodatne misli; je enako kot če bi umaknila pogled. Mogoče se dogajajo kake misli, vendar jih ne ozavesti;

Testni dražljaj: še enkrat gre skozi; vidi, da se pojavijo neke slike in vse počne v istem vrstnem redu;

Med testnim je njena pozornost na enem objektu naenkrat; pri vsakem pogleda repek in ugotovi, kam je obrnjen; aktivno jih primerja;

Trenutek odgovora: zelo je nezavedno in je precej neozaveščeno.

4 S01-04-O-02

Zelo se je zavedala, da sliši zvoke v ozadju (→ vdrlo je v njeno pozornost in potem so bili ves čas prisotni v njenem zavedanju) in je razmišljala »kaj kurac dela?!« (ni bila verbalizirana, ampak je bil bolj občutek → ta »kaj kurac dela« je interpretirala med intverjujem; nekaj jo je ziritiralo (→ neprijetno ji je bilo, moteče)) in ne ve, ali samo dejstvo, da je slišala zvok, ali so bili zvoki sami po sebi moteči)

Njena pozornost ni bila tako intenzivna, kakor je bila pri prejšnjem primeru. Zaradi tega so zvoki lahko vdrli v njeno doživljanje. Bila je bolj razpršena v smislu, da je vanjo vstopalo več elementov okolja. Med samo nalogo te pozornosti ne bi mogla intenzificirati, ampak bi terjalo nek dražljaj, da bi prekinila to stanje.

Trenutek odgovora: lahkotno ji je pritisnilo v levo, v desno pa terja več napora. Zdi se ji, da je več pravilnih kot napačnih. Več mišične aktivnosti je treba, da odgovori na desno tipko; bolj čuti tipko na blazinici kot sam prst;

Zdi se ji, da prvega vedno zajebe, ko začne reševati blok (→ ker drugje začne, vedno si zapomni, da gre od levo spodaj proti gornji, pri prvi pa začne kar nekje in to ji je nenaravno). Kot da bi jo nekaj presenetilo → neznan dražljaj je. Vsi dražljaji so ji sicer znani in lahko predvideva, kaj se bo zgodilo. Pri prvem pa ni konsistentno, kdaj se bo začeli pojavljati.

Pri spominskem dražljaju pogleda vsakega posebej in vedno v istem vrstnem redu in pogleda kam je obrnjen repek. Čeznje gre z očmi in to mora narediti zelo hitro; med pavzo naredi *brain print.* Med testnim pa gre po istem vrstnem redu in za vsakega preveri, če je enako usmerjen. Preprosto so se ji dajali kot isti ali drugačni.

Pogleda s pozornostjo: pogleda vsakega posebej, potem pa sledi s pozornostjo usmeritvi repka. Pozornost je voljna.

5 S01-04-P-01

Grozno je bilo! Bilo ji je neprijetno, ker je obremenjena s tem, da mora prav rešiti. Čuti telesno napetost in da je malo pod stresom. V njej je nek občutek, da se tako rešuje naloge. da to ni neko raziskovanje, ampak je to nalogo. Če imaš navodila (se jih ne zaveda aktivno) in jih razume, mora prav rešiti. Preprosto ve, da mora to naredit.

Spet je sledila *brain printu* in to ni bila učinkovita metoda → ker se je njeno prepričanje o odgovoru zmanjšalo: omahujoče pritisne na tipko;

Terjalo je več pozornosti, ker nima referenčnih točk. Samo čakala je, kdaj se bo prikazalo in si poskušala zapomnit ta vtis.

Spominski: s pozornostjo je sledila pojavnosti objektov; zraven ni bilo zvokov ali črte;

Zaznavala je zvok iz druge sobe;

Pavza: skonstruira, kje so se pojavile prek zgoraj navedenega *brain printa.*

Testni: poskuša iti skoz; s pozornostjo sledi pikam, ki se pojavljajo in če to ustreza brain printu, pritisne levo;

Večinoma ni prepričana in tako pritiska desno: čuti dvom → omahuje pri odgovoru.

Za noben odgovor si ne drzne trdit, da ga je pravilno odgovorila;

Zdi se ji, da je nek prazen prostor in da ni nekih okvirjev in ji je ful preveč abstraktno. Nima neke referenčne točke.

Frustracija, ker si ni mogla zapomnit → pod stresom: zakrčenost mišic.

6 S01-04-P-02

Ful je blo bolje, ker je uporabila novo metodo;

Za referenčno točko si je izbrala fiksacijski križ in je opazovala, kam so usmerjene. Na podlagi tega je opazovala, kje so se pojavili objekti;

Fokus: ni bila tako ostra kot prej, ampak je bila ostrejša. Ta je bila druga najbolj ostra. Tako je posamezne objekte le ošvrknila s pozornostjo;

Pavza: *brain print*; drugače kot prej, ker je bila pozornost drugje;

Testni: njena pozornost je bila na fiksacijskem križu. Objekti so se ji dajali kot pravilni ali drugačni in to ni bilo aktivni.

Manj je bila napeta in je bila bolj vesela sama v sebi → zadovoljstvo, ker je odkrila novo metodo. To je bil mentalen občutek, ker rešuje bolj prav. Telesno je le čutila, da je bolj sproščena.

Ta sprememba strategije so je kar zgodilo: preprosto začela je tako delovati, kar zgodilo se je;

Meta-kognicija: reakcijski čas se je zmanjšal in bolj je sproščena in zmanjšala se je obrmenjenost s tem, da mora reševati prav, ker ima že tako večje prepričanje, da rešuje prav.

7 S01-04-C-01

Ugotovila je, da strategija od prej ni tako zelo učinkovita. Zato si je zdaj pogledala celo sliko in ji je bilo lažje. Dejansko si je morala reč, katere barve so bile;

Še vedno si je naredila *brain print*;

Bila je prepričana, da bolje rešuje → bila je bolj sproščena, čutila je neko zadovoljstvo.

Telesno ji je bilo prijetno;

Še vedno je prvega v bloku zajebala;

Spominski: pogleda vsak objekt posebej in prejme občutek za barvo. Prišlo je v nekem miselnem toku. Prejela je srž posamezne barve;

Pavza: ni šla do vsakega kroga posebej, ampak je doživljala vtis cele slike. To ni bila vizualna izkušnja. Ne bi mogla rekonstruirat vseh barv; najbrž bi lahko rekonstruirala tri ali štiri;

Testni dražljaj: pogledala je celo sliko, ne vsake posebej. Dražljaj se ji je že dajal kot isti ali drugačen;

Strategija se je kar zgodila;

Zahteve naloge: fokus se je zožil.

8 S02-02-P-01

Uporabljala je isto strategijo, kot sicer; poziv k poročanju jo je malce zmedel;

Risala je krivuljo, oziroma smer, ki so jo naredile te pike → povezala jih je med sabo;

To je bilo vizualno: konkretna črta, ki je bila črna in bi jo lahko konkretno narisala; nahajala se je pred njo;

9 S02-02-P-02

Spominski dražljaj: ko se pike pojavljajo, jim sledi s pogledom;

Pavza: izriše si krivuljo, nekako z isto hitrostjo, kot se prikazujejo; ob tem malce premika glavo;

Enkrat si izriše, ker ima občutek, da fino sledi tem smerem in je precej samozavestna glede tega, da ve, kje se bodo pike prikazovale;

Edina referenčna točka jim je fiksacijski križ in ni nobene mreže; spraševala se je, kakšna je ločljivost tega prostora; ne ve, kako prefinjene so lahko razlike;

Spominski dražljaj: sledenje s pogledom: ni bila tako zelo pozorna na pogled, ampak na eni točki je opazila, da premika celo glavo. Ne ve, ali to vedno dela, ampak zdi se ji, da je malo lažje. To je avtomatiziran gib; je kot neka rezerva, kot neka podkrepitev risanja črta; lahko pride zraven, vendar ni nujno;

Risanje črte: kakor da bi videla animacijo, ki riše črte; črta nastaja zvezno; začne pri prvi piki, se izriše, pri drugi se malce ustavi; zelo se zaveda smeri in gibanja. Ona sama ni popolnoma statična. Bolj kot na sliko, ki nastaja, je pozorna na trenutno gibanje, ki se izrisuje; ko je slika že izrisana, vidi celo obliko; ni več tako, da bi jo vleklo v katero smer, ampak jo poskuša povezati v en gib. Začne s štirimi diskretnimi gibi (spominski dražljaj), ki jih potem poveže v enega (pavza); to naredi tako, da jih še enkrat nariše;

Krivulja je pomembnejša, kakor pike in ne ve, če se ji izrisujejo med pavzo;

Samozavest glede odgovora: ko je enkrat videla nalogo in uporabila strategijo z risanjem, se ji je zazdelo, da se ji to obnese, ker rešitev kar pade ven; ni se ji posebej treba trudit za strategijo; bolj je bilo kot bi gledala video, kakor reševala nalogo;

Kontrolna enota: danes ni bila prisotna;

10 S02-02-P-03

Ob koncu testnega dražljaja je imela občutek, da se ujema; dokler se je testni dražljaj izoblikoval, je sledila krivulji, potem pa je prepoznala ujemanje;

Bilo je, kakor če bi vzela podobo, ki jo je ohranjala med pavzo in z njo prekrila testni dražljaj. Ker sta se prekrivala, je sklepala, da se ujema;

Včasih se pike rišejo tako, da je krivulja odprta in nastane zanka. Takrat se mora zares osredotočiti na to, da je zanka in da ne ustvari nekatere bolj kompleksne oblike. V tem primeru črta rata malce bolj prozorna;

Na neki krogi se je krog pojavil čez fiksacijski križ in ob tem ji je bilo prijetno, ker je imela zelo jasno referenčno točko;

Fiksacijski križ: doživlja ga kot neko referenčno točko; tudi medtem ko si riše, se ji zdi, da za ta plus ne ve, koliko je prisoten, ker ga preslika iz zunanjega okolja v svoje dojemanje; kadar pa pika prikrije plus, je pa bolj gotova, da se nahaja na sredini in če je ena točka, ki je lokacija premika smeri, se ji zdi, da ima eno informacijo manj za ohranjat, ker sta in pika in plus na istem mestu;

Sicer pa se običajno ne zaveda plusa;

11 S04-01-C-01 (splošno)

Naloga se ji ni zdela težka, kljub temu pa je mestoma pomislila, da je mogoče naredila napako;

Udeleženka je sprva preverjala različne strategije, npr. da je grupirala objekte na zgornje dva, spodnje dva, ali pa na diagonalo. S to strategijo je bila prepričana v pravilnost svojega reševanja;

Pravilnost svojega reševanja je doživljala kot to, da je bila za ene odgovore popolnoma prepričana, za ene pa je med pavzo spremenila svojo strategijo (npr. pri enem primeru je vizualno izkustvo dražljaja spremljal vzklik »rasta!« Ko je odgovorila na ta primer, se je istočasno ovedla svoje zmote in sicer, da so bile barve iste, vendar so bile razporejene na drugih mestih;

Vzklik »rasta« si je konkretno rekla v glavi. Ni se zavestno odločila, da bo uporabila strategijo verbalizacije, ampak je do te besede prišla spontano;

Zelo hitro med samim reševanjem je ugotovila, da si ne bo mogla zapomnit vseh štirih in je začela grupirati objekte → po prvih dveh primerih je zgotovila, da ji ne ostanejo vsi objekti in jih je začela grupirati; do tega ni prišla prek nekega sklepanja ali sprotnega komentarja, ampak je preprosto spremenila svojo strategijo; Ugotovila je, da je naloga *tricky* in posledično je spremenila svoj odnos do nje;

Spoznanje, da ji naloga ne gre, se ji je kazalo tako, da je preprosto spremenila svoj način reševanja, potem pa je začutila neko sigurnost, ko se je soočala z manjšim številom objektov;

Grupiranje ji je dajalo večjo gotovost, neko prepričanost, da za dva objekta stoodstotno ve, za druga dva pa pričakuje, da se bosta nekako ohranila. Prepričana je, da če se preostala dva objekta zelo drastično spremenita, bo lahko opazila to spremembo;

Stranska objekta sta bila bolj v obrisu;

12 S04-01-C-02 (zadnji primer)

V trenutku poziva se je zavedala rožnate in svetlo modre barve;

Meni, da ji je v zadnjem primeru šlo slabše, ker je dajala več poudarka na svojo refleksijo reševanja, ne pa na samo nalogo;

Po testnem dražljaju si je v glavi izrekla imena barv, čemur je sledil vzklik »Aha, vidim, da si kdaj tudi govorim barve!« ob koncu artikulacije pa se je pojavil že naslednji dražljaj, kjer je morala svojo pozornost aktivno preusmeriti nazaj na nalogo;

Drugi tok spremljanja naloge jo je zmotil in to ni bil njen naravni tok reševanja, čeprav je prepričana, da jo je ta tok zmotil le zato, ker se ga je nenadoma ovedla in meni, da če bi nalogo reševala dlje časa, da bi se lahko privadila tega toka;

Spominski dražljaj: spodaj, gor, desno + verbalizacije;

Pavza: še enkrat si je izrekla tisti dve barvi, ki si ju je namenoma zapomnila (»modra, črna, modra, črna«)

Splošno: Med pavzo je kdaj gledala na isto mesto in si predstavljala, kaj je bilo tam, poleg tega, da je verbalizirala barve. To predstavo je dojemala kot njeno resnico. To je bilo vizualno izkustvo. Barve med spominskim dražljajem so izrazite, med pavzo pa so bile te barve prosojne, kakor da je bil čeznje poveznjen nek filter. Čuti, da je te barva ustvarjala ona, Včasih je med pavzo le gledala v ekran in čakala na testni dražljaj, včasih pa je poustvarjala te barve v svoji glavi. Ni zares točno videla teh barv, ampak si je zgolj predstavljala bolj bledo različico teh barv. Bile so dovolj različne in izrazite, da je vedela, kaj so in da jih ni izgubila.

Testni dražljaj: odgovori, da je pravilno rešena naloga; čemur je sledilo spoznanje, da si je barve artikulirala; po čemur se je morala prisiliti, da se zbere in da se ne osredotoča na to, kako gleda;

Vse te misli so bile verbalizirane, artikulirane z njenim glasom;

Ta glas doživlja kakor moteč in se zaradi njega ne more osredotočati le na vidno zaznavo naloge;

Med pavzo je bila običajno njena pozornost malo širša od točke;

Med reševanjem naloge se spraševalca ni zavedala.

13 S04-01-C-03 (zadnji primer)

Spominski dražljaj: direktno je vzpostavila fokus pogleda in je pogledala celotno sliko, ob čemer si je verbalizira »modra, črna, temno modra.«

Pavza: Še enkrat si je ponovila besede;

Testni dražljaj: vidi, da se pojavijo iste tri barve.

Prve tri barve si je zapomnila z neko sigurnostjo;

Med pavzo jo je začelo motit, ker si ni mogla fokusirano ostajat, ker so ji ostajale paslike. Torej je popolnoma fiziološka paslika zmotilo njeno predstavo barv, ker so te paslike tudi imele neko barvo;

Te paslike bi morala aktivno odmisliti, oziroma si močneje predstavljati, kar je videla prej;

Paslike je odmislila tako, da si je bolj verbalno ponavljala, kar je videla med spominskim dražljajem. Z verbalizacijami ji je bilo lažje *overridat* paslike;

To da je moteče, je doživljala tako, da so je kar naenkrat morala porabiti več mentalne energije, da je spremljala nalogo. To je terjalo več pozornosti, pozornost sama pa je bila drugačna od prejšnje. Prejšnja pozornost je bila bolj *in the zone* pozornost. Ni terjala truda in se ji je zdelo, da se ji to dogaja. Zdajšnja pozornost pa je bila bolj prisiljena in vanjo je morala vlagati večji nivo energije (prim. ko je utrujena in bere nek akademski članek);

Okolja se ni zavedala, ker se je tako močno ukvarjala z nalogo in z motečim elementom;

Verbalizacije so v njenem glasu, ki je isti kot ko govori, ampak se ne posluša;

Zapomnila si je tri barve, četrto je opazila, ampak je ni izgovorila; Ve, da je bila rumena, vendar je ni izgovorila, ker bi to dlje trajalo. Če bi se spremenila, meni, da bi verjetno opazila, predvsem, ker je tako drugačna od modrih in črne.

14 S04-01-O-01 (splošno)

Naloga ji je bila kar težka;

Ni se mogla orientirat glede neke točke;

Misli, da ji ni šlo dobro;

Če je bila zbrana in če se je odločila, da si je zapomnila le dve ali tri, oz. se je zanašala na paslike, se ji je kdaj pa kdaj zdelo preprosto, po čemer je izgubila fokus in naloga se ji je spet zazdela težka.

V tistem trenutku je v mislih verbalizirala: »To je pa *easy*.«

Če si je zapomnila tri in so bile pravilne, je bila 90% prepričana, da je bila četrta tudi; ne ker bi vedela, da je četrta pravilna, saj za razliko od barv sploh ni vedela, kakšna je četrta;

Ko se ni zanašala na paslike, je bil njen fokus truden;

Paslike pa je videla le, ko je bila v sproščeni pozornosti;

Paslike so nekaj strogo vizualnega; pojavijo se ko dražljaj izgine, potem pa njihova barva hitro bledi. Zmedlo jo je, ker so se pojavile tudi po testnem dražljaju in so ostale v spominskem dražljaju naslednjega primera; paslike se ji dogajajo in če jih ne bi izkoristila v svoje dobro, bi ji bilo moteče;

Misli, da si ni treba zapomniti le štirih stvari, ampak da si mora zapomniti tudi kam moli krogec in je veliko več stvari, ki si jih mora zapomnit;

Na začetku je ponovno preizkušala različne strategije;

Občasno je iskala tudi neke verbalizacije, sploh kadar se je preveč osredotočala na posamezen objekt; ta govor je bil kvalitativno podoben nezavednemu vzkliku »rasta!« → tega si ni želela govorit;

Na začetku ji je naloga šla prehitro in si ni uspela izgovarjat besed, le neke foneme, neke momljaje; to strategije so potem zamenjale paslike, na koncu pa se je odločila za strategijo, kjer si je zapomnila manjše število objektov, ki jih je verbalizirala (npr. »gor, levo.«

Udeleženka sicer čuti avtorstvo nad temi besedami, vendar se ji ne zdi, da si si jih je izbrala, ali sprva proizvedla z nekim namenom.

15 S04-01-O-02

Njena strategija je bila, da si je pogledala dva objekta in si ju zapomnila stoodstotno, ko pa sta enkrat izginila, jima je dodala pasliko;

Med testnim dražljajem je iskala ujemanje;

Le za dva primera, so ji malo uhajale misli, ko si je rekla. Takrat je morala potlačit zametke nekega govora;

Proti koncu spominskega dražljaja je oči usmerila proti tretjem dražljaju, kjer je ob začetku pavze zagledala pasliko;

To, da je potlačila govor je doživljala kot preusmeritev pozornosti, ne pa kot nek notranji dvogovor;

Te misli so bile v veliki meri nek meta-komentar na njeno reševanje, neko razvijanje strategije, ki pa ga je vseeno morala odpraviti, da je lahko spremljala nalogo;

Ob teh treh zapomnitvah je čutila neko samozavest, ki je podobna tisti iz prejšnje naloge z barvami; nima občutka, da nekaj dela, ampak z vsako uspešno nalogo, se ji zdi, da nalogo lažje rešuje; ta rast pozornosti je postopna, malo po malo;

Ko je bila enkrat bolj vešča naloge, se ji je občutek časa spremenil in počutila se je, da lahko v svoje doživljanje dodaja stvari, takrat pa je med pavzo začela dodajati tretji dražljaj;

Vzpostavil se je nek *flow* in manj je bilo nekega paničnega premikanja z očmi;

16 S04-01-O-03

Med spominskim dražljajem je gledala na sliko;

Pavze ni zares doživljala, ampak je le potrjevala pri sebi in takoj se je pojavil testni dražljaj;

Ko je bilo kdaj kaj drugače, si je pri sebi povedala »aha, to je bilo prej gor, zdaj je pa dol.« in spet se je morala prisiliti, da je samo reševala nalogo, ne pa se ukvarjala z nekim notranjim govorom;

Trudila se je, da je le ohranjala pozornost in si nič govorila;

Fokusirala se je na vse štiri objekte; hotela je zaobjeti celotni dražljaj, ne pa posameznih objektov. To je lahko počela le, če ni verbalizirala, saj ji je to zavzelo preveč časa in pozornosti.

17 S04-02-P-01

Zdelo se ji je enostavno; bila je sigurna (sigurnost je zelo postopna; ko se je pojavil prvi primer in je bilo očirno, da so iste, si je mislila, da je to lahko, s časom pa se je to spoznanje prelilo; preprosto se ji je zdelo, ker od nje ni terjala mentalnega truda, ali velike skoncentriratnosti; rekla si je »ni panike,« → ta govor je bil v drugem toku, kot štetje; mentalni glas, ki ni zares glas, ona je avtorica, ne sliši ga kot da bi si ga zares rekla; ni jezik, ampak je samo vsebina; ta vsebina je naredila razliko v počutju, v samozavesti; to se ji je dogajalo) v svoje odgovore, razen enkrat, ko ni bila prepričana, ali je prav pritisnila; vzklik je bil zvezan z nekim spoznanjem, ki jo je dal v bolj komot stanje; ni bil samogovor, kakršnega si govori pri športu, to je bilo bolj spoznanje, nek aha moment;

Komotnost: ni da dobi neko gotovost, ampak izgubi stvari, ki bi jo motile; ko ve, kaj dela, nima nekih dvomov, in ta *conifdence* v delanju je bil prisoten ti (ni prisotnost nečesa, ampak je pomanjkanje nesigurnosti)

Zadnji primer je doživljala zelo sproščeno; bila je skoncentrirana na nalogo; ni imela dodatnih misli;

Koncentracija: bila je lahkotna, oapzila je, da ko naloga nekaj časa traja, da ji pridejo neke misli, ampak zdaj po začetnih mislih, ni bilo dodatnih misli; padla je vanjo in ni je direktno proizvajala;

Verbalizirala is je »en, dva tri, štiri.« zgodile so se v istem ritmu, kakor se prikazujejo krogi; te verbalizacije so bile v zvoki, v njenem glasu; ritem ji je pomagal pri koncentraciji; to je strukturiralo njen čas; ni bil namenoma plesni ritem, ampak jo zelo spominja na ples, ker si moraš šteti; ona je avtorica teh besed; voljno jih proizvaja; ko so se prvič pojavile, jih ni voljno ustvarjala, češ da bo ubrala strategijo, ampak je opazila, da to počne in da ji je prijetno, ker ji daje struktira; to doživljanje je zagrabila in ga potlej počela;

Prijetnost ob ritmu: ker je predvidljivo in daje strukturo, ni lovljenja in iskanja. Iz prve da neko strukturo, ki ji je kul sledit; ni občutila želje, da bi kaj spremenila;

Že od začetka se ji je v glavi začelo štetje; ni se odločila, da bo štelo;

Štetje je vzpostavljalo nek tempo in v glavi si je zamišlja, kakšno pot so naredili objekti;

Drugačnost: večinoma je bilo ful opazno; očitnost → v spominskem delu se ji je preko štirih dražljajev zgodil zelo trden vzorec iz teh zamišljenih črt, kakor da bi se naredila pot; med tetsnim je pri vseh nepravilna šla ven iz te poti;

Pot je bila vezana na gibe z očmi; v tem ni bilo vizualne komponente, ampak si je predstavljala prostor, kjer naj bi se premikale pike;

Kadar se poveže v neko obliko (V ali metuljček, je lahko zapomnljivo)

Primerjava: ni bilo aktivne primerjave, ni bilo da je iskala, ali pike padejo v pot, niti ni bila pozorna na to, kaj bo drugače, ampak se ji je testni dražljaj dajal drugačen; zaporedno je pogledala vsak objekt in ob vsakem gibu je doživela neko potrditev;

Potrditev: vsaka pika je v sebi nosila neko potrditev, šele zadnja je bila napačna; o istosti je bila 100% prepričana (na vsaki točki ji en delež da prepričanost, ki je bolj nek *note* sam sebi, češ »ja,« na koncu pa se poveže v neko celoto; prepričanje je neko pomanjkanje dvoma; kvalitativno je podobno temu ritmu), ker se je 4x potrdila;

Pri kateri se je zmotila v tem, kako male razlike so bile mogoče med dražljaji;

Pred začetkom naloge je čutila neko neutemeljeno anksioznost; ko pa začne reševati ni panike;

Asociacija: »V« → je bila bolj čvrsta povezava tega in si je bilo lažje;

Testni dražljaj: naredila je preslikavo med spominsko; v glavi si je naredila vzorec med testnim, pa si ga je vzela samo še enkrat skozi; vzorec si lahko za kak trenutek predstavlja kaj bi narisale te črte: to je za hipen trenutek; tega ni ohranjala v glavi;

Pavza: med pavzo bi ga lahko narisala;

Spominski je naredil original sliko, drugo je bilo pa kot da bi testnega dali čez;

Zahteve naloge: že ko se je usedla za računalnik, je začutila anksioznost, češ da bi vse skupaj čim prej minilo; da bi se spravila iz tega skoraj začetka; ni pa bilo povezano s tem, kako bo reševala nalogo; ko je videla, da ji ni težko je anksioznost zbledela;

Ritem je nadomestil anskioznost;

18 S04-02-P-02

Naredila je klasičen Z;

Testni dražljaj je bil identičen;

Bil je siguren in *confident* odgovor (dva v rundi nista bila taka);

Spominski: »en, dva, tri, štiri,« + s pogledom ustvari prostorsko pot, ki je bledela skladno s premikanjem oči (miselna črta);

Ni si rekla, da gre za Z, samo prepoznala je to obliko → šele ko sem ji rekel, naj opiše obliko, jo je poimenovala s črko Z; v glavi je držala le podobo črke Z; to ni bila neka slika, ni bila predstava v zunanjem vidnem polju, niti domišljanje v notranjem vidnem polju, samo imela jo je, ker je znan simbol; videla je obliko in je ni poiemnovala, ampak je vedela, da če bi jo morala, bi jo znala (srž);

Pavza: ponovila je »en, dva, tri, štiri.«

V ritmu pride testni dražljaj in je tako, da ponovi zadevo že v tretje; 1 2 3 4 1 2 3 4 1 2 3 4;

Meta-kognicija: dva primera ni bila prperičana; ustvarila sta isto pot, ampak je bila mogoče en cm levo ali desno; to ni čisto pokvarilo ali obrnilo oblike. To je zaznalo prek pomanjkanja popolnega prepričanja, v dvomu; ta dvom jo je malo ustavil, prekinil je njen ritem; prim. ko ima prav, ko po vsakem odgovoru doživi mentalno potrdilo; vsaka nova naloga, skozi katero je šla s tem prepričanjem, ji je dala dodatno potrditev;

Zahteve naloge: na začetku je ponovno doživljala neprijetnost + spominček, ki ji je dal vedeti, naj bo bolj humble (spomin na dogodek s faksa= → v slikah: prišel je kar iz nekje; ni ga ona pozvala (podoben kot ko športa in dela nekaj kar *flaunta,* češ *I got dis,* temu pa sledi poraz); ona je avtorica; spomini se ji dogajajo (ne da ga prikliče, pride ji v glavo, potem pa ga malo opazuje, preden se njene misli oprijemo nečesa drugega); trajalo je delček sekunde;

19 S05-01-C-01

Spominski dražljaj: ni točno vedel, kaj naj bi delal; od njega je bil disociiran. Njegova pozornost je bila drugje. Ni še imel ponotranjenih navodil, da bi takoj vedel, kaj je moral narediti;

Pavza: v mislih pred sabo je poskušal imeti vizualiziran ta vzorec; projiciral ga je na zaslon;

Pike je videl v nasprotni smeri urinega kazalca, kjer so bili v obliki kroga razporejeni vtisi barv;

Bili so, kot da si jih je naslikal na zaslon. Dejansko je imel nek občutek zaslona; vedel je, da je zaslon pred njim, barve je pa odlagal na neko notranjo idejo zaslona. Bilo je kot da je bilo pred njim, obenem pa se je zavedal obeh zaslonov;

Te vtise je razporedil krožno;

Testni dražljaj: definitivno je primerjal: dražljaj z ekrana je primerjal s svojo idejo ekrana, če vsaka paše na pravo mesto;

Pri zadnji se je zmotil: občutek zmote: del njega je naredil nekaj v dobri veri, da dela prav, kljub temu da je iz vizualne podobe vedel, da dražljaja nista enaka;

Overlap pri primerjavi: vzel je spominski dražljaj in ga dal v svoje misli, kjer je položil eno sliko na drugo, s tem da je bila druga bolj podobna temu, kar je dejansko videl na ekranu, prva pa je bila bolj zgoščena.

Primerjava se mu je dogajala in v trenutku ni razmišljal o tem;

Občutek zmote: nepozornost med premikom in notranjo sliko teh dveh podob;

Za napačen odgovor je bil kriv »del njega« → to je naredil nek notranji impulz, ki se kaže kot nek pretanjen občutek, da ni prav, ki je vzniknil ob istem trenutku, ko je imel pred sabo sliko, ki je kazala, zakaj to ni prav;

Občutek, kako dobro si je zapomnil ni prisoten;

20 S05-01-C-02

Poskušal je imeti drugačno strategijo;

Gledal je križec in si poskušal zapomniti, kaj ga obdaja, kar je bila 2D slika krogcev, ampak kvadrata pa ni bilo, oblike v resnici niso bile prisotne;

Fiksacijski križ: doživljanje je bilo zgolj vizualno;

Poskušal je voljno dojeti neko celoto;

Poskušal je imeti drugačno strategijo: v njem se je pogosto dogajalo, da je imel instinkt da počne, kar je počel prej, ki ga je pa poglobila njegova ideja, da počne drugače; po tem je včasih prisoten tudi dvom v učinkovitost te strategije. Ta se je kazal kot da izvira iz mesta v glavi, ki ima neko skrb nad tem procesom, iz nekega mesta, ki ima tretjeosebni pogled nad njegovim početjem; ta del se je oglasil med novimi strategijami (tega meta-pogleda se je zavedal le pri konfliktu med strategijami);

To je bil občutek, ki je bil kot blaga anksioznost; ta tesnobnost je bolj mentalna, neka notranja, možganska panika. V nekaterih primerih je bilo to zvezano s samim reševanjem naloge in ne tako meta-pogledom;

Jaz sem v nekih primerih bil del njegovega doživljanja;

Pavza: med delom pavze se je dogajal dvom, na plan pride zavedanje o njegovi strategiji, pred očmi pa je imel podobo fiksacijskega križa in teh barv;

S prejšnjo strategijo bi ta vtis lahko narisal, s to pa zagotovo ne;

Testni dražljaj je bil onkraj njegovega zavedanja;

Strategija je bila prisiljena;

21 S05-01-C-03

Nova strategija: vidi barve, oči premakne v nasprotni smeri urinega kazalca in si ustvari sliko; fiksacijski križ je onkraj njegovega zavedanja;

Tu je zraven verbaliziral barve; to so bile voljne besede, ki niso bile izrečene kot cele besede; ustvarjal si je tako vtis barv, kakor besed;

Ta strategija je bila posledica njegovega poskušanja izboljšati svojo strategijo;

Konkretno je slišal svoj glas, ki je bil malce zamazan, artikulacija besed pa je terjala čas;

Pavza: ni si ponavljal besed, si je pa osveževal barve; te so bile podobne kot v primeru S05-01-C-01. Vizualne v krožni porazdelitvi; to osveževanje se mu je dogajalo in ni se mu bilo treba prepričevati, naj to počne;

Testni dražljaj: bolj je bila dominantna komponenta verbalizacije; s pogledom je šel čez barve, ki jih je videl in si ponovil besede;

Če ni bil prepričan, pa se je zanašal na *overlap* iz S05-01-C-01.

Imel je občutek, da je bolj uspešen, imel je neko samozavest;

Ko je pritisnil na tipko, je imel neko notranjo vero, nek tretjeosebni opazovalec mu je podal afirmacijo → spremljal ga je občutek, čustvo samozavesti, prepričanja vase, kar se je kazalo v bolj prepričanih pritiskih na tipko;

Govor je bil prisiljena strategija;

22 S05-01-C-01

Doživljanje je bilo tu najbolj podobno S05-01-C-01.

Prisoten je bil tako občutek samozavesti, kakor zavedanje, da se ne trudi več zares;

Cel proces mu je prišel pod koži; ve, kaj pričakovati; ni več vlagal znatnega napora v nalogo;

Pavza: v zadnjem primeru je v nasprotni smeri urinega kazalca poskušal osvežiti to, ampak v zadnjo barvo ni bil prepričan; ostal je zamazan spomin: od nje ni ostal noben vtis, bilo je le prazno;

Testni dražljaj: podobno kot 01 in 03, le da je imel občutek napake, vendar mu je bilo vseeno in ni iskal notranje validacije; zadnja barvo mu je izginila iz zavedanja.

S05-02-O-01

Ni nujno zaznaval krogcev in črt, ampak se je osredotočil na orientacije;

Pavza: ni počel ničesar posebnega;

Testni dražljaj: približno po nekem fotografskem spominu je primerjal dražljaje;

V ospredju njegovega zaznavanja so bile orientacije; te so bile usmerjene iz oblike; dobil je *gist* smeri, njeno bistvo;

Zmanjkovalo mu je časa: poskušal si je zapomniti celo sliko, ampak je ni vse zajel s pogledom; še vedno jo je preiskoval v nasprotni smeri urinega kazalca in si dobro poskušal zapomniti črte;

Prevladovala sta neprepričanost in občutek, da ni dobro videl vsega;

Pavza: ni počel ničesar določenega; le taval je z mislimi → kazale so se mu kot izjemno zmedena disociranost od problema;

Poskušal si je zapomniti nek fotografski spomin: med spominskim dražljajem si je zapomnil splošen vtis slike, med testnim dražljajem pa se ne spomni, kaj se je dogajalo;

Bil je skrajno neprepričan v dražljaj: ni želel pritisniti tipke;

Obenem pa se mu je neprepričanost mentalno kazala, ker ni imel ustvarjenega točnega spomina;

Imel je občutek, da ko si je ustvaril ta spomin, da je en del, eno oblik le hipoma ošvrknil s pogledom, da jo je mogoče ujel le s perifernim vidom. To se nanaša na to, da v resnici sploh ni vedel;

23 S05-02-O-02

Dejansko je imel strategijo;

Bila je neka faza reiteracije;

Ugotovil je, da si lažje zapomni, če najde neke vzorce znotraj tega, kar vidi:

Ne išče več posameznih vtisov črte, ampak gleda, če so črte razporejene po nekem vidnem pravilu;

S pogledom spet sledi nasprotni smeri urinega kazalca;

Ustvari neke skupine, mogoče dva po dva, glede na to kateri objekti skupaj ustvarijo stabilen vzorec, ali pa nek lik;

Pavza: reiteriral je te oblike;

Testni dražljaj: primerjal je, kar vidi s temi podobami in odgovor je bil na hitro jasen;

Zanj se to dogaja po pravilih. Pravilo je zanj v veliki meri intuitivno jasno. To ni bilo verbalizirano: sprva jih je sicer hotel verbalizirati;

To je bilo zelo jasno in lahko bi narisal te oblike; definitivno je bil prisoten vizualni element tega;

Orientacije so bile težje na eni strani, bile so bolj masivne (to je bilo eno od pravil po katerem si je lahko nekaj zapomnil);

Pavza: povezave med liki si je izrisal na zaslon: predstavljal si jih je tam, to je bilo združeno s pravili, ki jih je izločil iz *gista* spominskega dražljaja;

[udeleženec nariše sliko] !!!

Imel je splošen vtis o sliki;

Težave mu je delalo, da med risanjem ni vedel, kje je krogec; iz tega je izviralo nekaj negotovosti;

Pozornost: imel je širok pogled na sliko, obenem pa je bil skoncentriran na eno črto; ta pozornost je bila naporna;

Testni dražljaj: podobno kot med pavzo si je na zaslon zarisal slike iz spominskega dražljaja, potem pa je iskal sovpadanje, kar se je kazalo kot nek klik;

Preverjanje in impulz da klikne, je bil popolnoma ločen od tega, kaj je hotel storiti. V glavi je imel dva argumenta in eden izmed njiju je prevladal;

24 S05-02-O-03

Pavza: iterira vizualne povezave med objekti; malo ima občutek, da ima malo časa za pavzo in da testni dražljaj prihaja, ob čemer je čutil tesnobo, da bo pozabil, preden bo videl dražljaj;

Aktivno je doživljal čas: v nekaterih delih naloge je čutil naraščajoč pritisk, oziroma kot da naj si vzame čase in še enkrat preveri: to je bila tendenca, v vedenju pa dejansko ni bila izvršena, to so bila bolj navodila samemu sebi

Spominski dražljaj: grupiranje in povezovanje s črtami;

Pri spominskem dražljaju je imel občutek, da mora malenkost bolj pohiteti;

Samo ozrl se je proti spominskemu dražljaju in še preden si je dobro zapomnil, je že iskal novo pravilo;

25 S05-02-P-01

Vse se je vrtelo okrog pomena in nanašanja na premikanje oči, čeprav ne ve zagotovo, da jih je dejansko premikal;

Mogoče je bolj usmerjal pozornost protu pikam in si jih zapomnil kot nek vzorec, ki je bil povezan s črto;

Pri spominskem dražljaju je pogledal in dajal pozornost na fiksacijski križ;

Pavza: počne isto, videl je križ, si predstavljal kroge in med njimi vlekle črte;

Testni dražljaj: čaka je na napačnega;

Črta: ni bilo vizualno, bil je prostorski občutek, kot neka pot; ni bilo jasne usmeritve; pot je imela neko ukrivljenost; lahko se je nanašala na dva dražljaja nazaj;

Testni dražljaj: ko je videl dražljaj je primerjal pot, ne pa same pike; ko je zaznal divergenco, je prejel odgovor;

Naloga je bila lahko:; vse oblike so bile spirale, kar so bile domače oblike;

Pozornost ni bila popolnoma pasivna, niti ni terjala hudega napora;

Še vedno je moral obliki pripisovati nek pomen;

26 S05-02-P-02

Spominski dražljaj: imel je časovno komponento prikazovanja: zaznal je objekte v smislu, da je za njimi ostal odmev, ki je počasi bledel, te odmeve pa je potem povezal med sabi;

On temu ne bi rekel pravila: ni jih videl na ekranu, ampak v svoji glavi;

Razen fiksacijskega križa se ni zavedal prostora onkraj dražljajev;

Pavza: videl je fiksacijski križ, potem pa je okrog njega skladno s sekvenco v glavi ustvaril podobo;

To je bil *gist*: 1) splošen vtis oblike → vedno ima neko obliko vzorca, ki ga je videl; 2) pot, 3) občutek, da ga dobi iz spomina;

Kljub temu da med pavzo gleda v fiksacijski križ, ve, kaj se dogaja okrog njega;

Fiksacijski križ je med pavzo tisto, kar gleda; je jedro njegove pozornosti; najbolj jasno ga vidi od vseh oblik v njegovi pozornosti;

Testni dražljaj: spet išče prvega v relaciji do fiksacijskega križa in nanj preusmerja svojo pozornost;

Ni imel občutka ločljivosti spominskega dražljaja (kako malo spremembe so mogoče);

Takoj, ko je nova pot skrivila s stare, je vedel, kakšen je odgovor;

Ko ima v glavi sliko neke oblike, je spoznanje čutiti kot prekrivanje;

S05-04-O-01

Zaznava kroge, ki so bili na eni strani bolj obteženi. To je bilo zelo vizualno izkustvo. Imeli so večjo gostoto na eni strani;

Pavza: poskušal si je predstavljati to črno liso na ekranu. V resnici je bil to nek trikotnik z medlimi robovi. Predstavljal si je neko pasliko in to ni bila zares vizualna podoba;

To dejanje samo je bilo naporno in je terjalo določeno mero koncentracije;

Testni dražljaj: gledal je, kako dobro je novi dražljaj sovpadal s paslikami. Gledal je, kako dobro so se prekrivali in bolj ko so se prekrivali, bolj je bil prepričan v svoj odgovor. Doživljal je nek gradient od gotovosti k dvomu, ki je bil zvezan z stopnjo enakosti;

Ta projekcija je bila le vizualna; ni je spremljal nek prostorski občutek;

Te projekcije niso bile stalne. Izginjale so in se pojavljale., nekako so utripale skladno s stopnjo pozornosti, ki jim jo je posvečal;

Med testnim dražljajem je istočasno videl projekcije in testni dražljaj;

27 S05-04-O-02

Poskušal si je zapomniti čim več modalnosti znotraj teh objektov;

Grupiral jih je dva po dva;

Ob tem je doživljal nek občutek usmerjenosti;

Pavza: nima občutka, da je med pavzo počel karkoli;

Testni dražljaj: iskal je ta pravila in jih pripisoval temu, kar je videl na zaslonu. Ima občutek, da ni uporabil vseh pravil. Zapomnil si je na več načinov, kakor jih je uporabil pri dejanskem prepoznavanju;

Ob tem ni imel občutka, da se mu kam mudi;

Ko je apliciral dovolj pravil, je odgovoril. Prišla je točka, ko je vedel, ali dražljaj ustreza, ali ne.

Usmerjenost: to je bila zanj popolnoma vizualna izkušnja, objekte je doživljal kot puščice (udeleženec ima močan občutek, da je to doživljanje skonstruiral med intervjujem);

Ena od modalnosti je ustvarjanje nekega gradienta. To ni bilo prostorsko, ampak je v tem nek kvazi-vizualni občutek;

Vizualno je videl te objekte. Gradient je bila neka abstrakcija, ki jo je povlekel čez to. Imela je protsorsko komponento v smislu, da to spremljal občutek globine;

Ta pravila si je zapomnil z nekimi verbalizacijami; te niso bile artikulirane v njegovem glasu, ampak je bilo samo neko zvočno znamenje, ki ga ne bi mogel proizvesti v svojem dejanskem glasu;

Ustvaril je intonacijo, ki je ustrezala gradientu in v njej so bile nekako implicirane te usmeritve;

Pavza: čakal je, da se pojavijo naslednji dražljaji;

Več ko je lahko pripisal teh pravil, bolj je bil gotov v svoj odgovor;

28 S05-04-O-03

Imel je lebdečo pozornost na celotnem kvadratu, kjer so se pojavljali objekti;

Pregledal je detajle slike;

Pavza: objekte je poskušal obdržati na zaslonu;

Iskal je sovpadanje na sliki; v resnici je iskal nekaj, kar bi ga zmotilo;

Projekcija je bila ostra, nahajala pa se je pred njim, vendar ne popolnoma na zaslonu;

Projekcija se je zdela, kakor da je malo upognjena, skladno z obliko njegove pozornosti;

29 S06-01-C-01

Zadnji primer ga je zmedel, ker mu je zavibriral telefon; telefon mu je prekinil tok misli in pomnjena;

Verbalizacija barv: besede so bile dejansko slišane, glas je bil njegov, malce zadušen;

Kroge je pogledal od leve proti desni;

Gledal je fiksacijski križ, strategija pa je do njega prišla naravno in mu o njej ni bilo potrebno posebej razmišljati;

Zaradi telefona je izgubil nek vtis barv: to je bilo vizualno doživljanje, ki se mu je zdelo zelo naravno in nad njim ni imel nekega nadzora; ta vtis ni bil vizualen, niti kognitiven, ampak je bil nek občutek;

Če bi bila v tem občutku dovolj velika razlika, bi jo prepoznal, še preden bi ga verbaliziral;

Barv ni zares videl med pavzo;

Testni dražljaj: opazi razliko, ne ve pa točmno, kaj je razlika;

Nadzor nad mentalnimi procesi je neka gotovost; močen občutek v telesu, zavedanje da lahko po potrebi nekaj prikliče;

S06-01-C-02

Zdi se mu, da je boljši v verbalizaciji;

Občutek za barve, ki so se prikazale je ta globalni občutek *gista;*

V veliki meri se je zanašal na verbalizacijo;

Globalni občutek: podobno kot prej; gre za občutek za barve, kakor se kažejo; to ni mešanica barv, na eni strani telesa čuti te barve, na različnih predelih tega globalnega občutka;

Iz njega samega ne bi mogel izvleči posameznih barv; zaznava ga nekje na višini glave in izven nje; 15cm stran od glave;

Testni dražljaj: preprosto vedel je, kakšen je odgivor. Kadar nekaj ni ustrezali temu, je prejel nek signal v smislu, da nekaj ni kakor bi moralo biti (nekaj ga je zaščemelo v trebuhu). Ko nekaj ni enako, je občutek konsistentno tak; enakost čuti v glavi;

Ta občutek velja tako za globalni občutek, kakor ta verbalizacije;

Globalni občutek je nekaj, kar se mu dogaja, besede za barve pa so nekaj, kar on počne:

Verbalizacije so artikulirane in imajo trajanje, razvlečeno skozi čas;

Občutek, da si je dobro zapomnil spominski dražljaj: ve, če si ga dobro ali slabo zapomni. To ni verbaliziran proces, to je nek občutek, ki se ne dogaja na zavestni ravni; v trebuhu občuti neko tesnobo; ta vsako tako preverjanje ali test se poskuša maksimalno potruditi in ga moti, če si nečesa ne zapomni dobro;

30 S06-01-O-01

Presenetilo ga je, ker je to isto nalogo že opravljal. To je bila kratka misel, ki je bila jasna in neverbalizirana. Ta misel se je kar pojavila.

Umel je misel, da si mora te naloge zverbalizirati, ker si bo tako bolje zapomnil → če si nekaj zverbalizira, ima močnejši občutek, da nekaj dejansko ve, vendar si ni upal, ker časovno ne bi šlo skozi;

Ponovno se je zanašal na ta globalni občutek, kar je bilo bolj vizualno;

Poskus verbalizacije je bil zavesten;

Spominski dražljaj: eksperimentiral je z zapominjanjem slike in ugotovil, da ga verbaliziranje orientacij hitro zmede in več pozornosti je posvetil vizualnemu pomnjenju;

Verbaliziacije je odmislil: spremljal ga je občutek, da je prepočasen, kar je spremljala nekakšna tesnobnost, ker je vedel, da bo hitro izginilo;

Prisotna je bila časovna dimenzija;

Prejel je nek občutek, da to ne gre dalje in preprosto je prevzel vidno modaliteto;

Posamezne orientacije si je poskušal zapomniti vidni;

Malo je drugače od globalnega občutka: tukaj je usmerjenost dajala občutek, da so objekti narisani na listu papirju. To je bil nek občutek v ozadju: zdi se mu, da si je poskušal zapomniti tako, da si je to prikazal v mislih, vendar tega ni dejansko videl;

Pavza: ni bilo verbalizacij; počel ni nič posebnega, samo poskušal je vzdrževati občutek od prej; to je on doživljal zelo hitro; na pol je to počel, na pol pa se mu je to dogajalo;

Testni dražljaj: naloga se mu zdi težja kot prejšnja, ampak občutek je bil, podoben; ni imel toliko nadzora in ni bil preveč prepričan v svoje odločitve;

Če si ni uspel vsega zapomnit, potem je ravnal po občutku; ne more zares reči;

31 S06-01-O-02

Spominski dražljaj: ponovno si je poskušal malo verbalizirati stvari; zdi se mu, da malo drugače čuti objekte, kot prej barve; te so se mu jasne prikazovale v mislih, medtem ko se mu občutek za smer ne;

To je neka paslika: on ne vidi dejanske vizualne podobe;

Občutek je bolj telesen; kot da bi bil center v njegovi glavi, on pa bi vlekel iz tega;

Pavza: med pavzo ni nekega posebnega doživljanja; zelo hitro mine in samo poskuša zamrzniti;

Poskušal je ohraniti te telesne občutke;

Testni dražljaj: preletel je vsakega posebej in pridobil nek globalni občutek; pozoren je bil na to, da če bo kaj občutil v trebuhu;

Globalni občutek: nima veliko vpliva nad njim; predstavlja zmes lokalnih občutkov, to je nek vtis Gestalta; tega vtisa ne vidi, ko primerja, sta si pa občutka nekako vzporedna; lokalni občutki so zaporedni; ni prepričan, da je to zares v trebuhu, bolj je nekje okrog njegove glave;

Nekje na istem delu njegovega vidnega polja, kot dražljaj pod vprašajem; to je podobno prej omenjenemu prostorskemu *feelingu;*

32 S06-01-O-03

Dvakrat se mu je zgodilo, da je vedel, kaj je pravilen odgovor, vendar je napak odgovoril;

Še vedno sta prisotna tako strategija z verbalizacijo, kakor nek prostorski vtis;

Čuti kanček sramu in tesnobe ob banalnih napakah: v trebuhu čuti stiskanje, najprej pride krč, ki zbledi in ostane v neki šibki obliki;

Spominski dražljaj: vznik globalnega občutka;

S06-02-P-01

Naloga se mu je zdela bolj dinamična, lažja in bolj zabavna je od prejšnjih;

Ima nek globalni vtis nad katerim nima nekega nadzora; ima nek občutek za prostor; glede na to, kje so objektivni relativno do njega;

Globalni vtis je bil manj izrazit; to je potekalo vzporedno z nekim prostorskim občutkom, pir katerem je center v njegovi glavi, potem pa posamezne objekte fizično postavlja v prostor;

Spominski dražljaj: njegov pogled je bil tako na fiksacijskemu križu, kakor na pika;

Pavza: rekel bi, da je med pavzo zamrznil; prisotno je neko pričakovanje → težko dostopa do tega doživljanja;

Naloga mu je bila zabavna. Ker ni vedel, kje se bodo prikazali objekti, so ga malo presenetili; ni mogel imeniti nobenega vnaprejšnjega *feelinga.*

33 S06-02-P-02

Pavza: ponovi vzorec, premika oči, premakne se na prostor, kjer so se prej nahajali objekti. Ti za sabo pustijo prostorčke, ki jih doživlja zelo surovo. Zraven si nič ne reče, zdi pa se mu, da spustijo nek zvok, sliši nek ritem;

V teh prostorčkih je neka razlika od preostalega prostora: ob njih v trebuhu doživlja nekakšno težo;

Testni dražljaj: primerjava:: čuti, da je razlika v doživljanju → enostavno ve, kdaj je in kdaj ni; začuti, da ga v trebuhu nekaj stisne;

34 SO6-02-P-03

Testni dražljaj: zdelo se mu je, da je bil napačen; da ni bil enak spominskemu dražljaju; ni imel prave prostorske umestitve;

Pavza: med pavzo si je parkrat celo podal neko verbalizirano potrditev;

T mislimi in glavo je še enkrat šel od vzorca do vzorca;

Fokus v mislih: med pavzo je s fokusom sledil objektom: to lahko primerja s tem, ko pred samim intervjujem ustvarja vidno pozornost, ob tem pa se spusti nek zvok;

Ta pogled je zvezen: počasi pogleda od točke do točke: poti ne doživlja, to je nek prazen prostor (ta pot je manj jasna kot prostorčki, kjer se pojavljajo objekti);

Ta fokus ni lahkoten in ga je spremljal občutek agence;

35 S06-03-O-01

Zapomnil si je prek kombinacije verbaliziranja in vidne modalitete. Slednjo je doživljal kot nekakšen globalni občutek.

Med pavzo mu v mislih ostane vidna slika, ki sicer hitro bledi; nahaja se nekje med ekranom in njim;

Spominski dražljaj: zaporedno pogleda dva objekta, za druga dva pa mu zmanjka časa;

Prvima dvema pripiše verbalizacijo, za druga dva pa se nanaša na pasliko;

Pavza: obnovi spominski dražljaj. To sliko je kreiral on, čeprav ima občutek, da je prej preprosto bila tam. Ob tem se je zavestno premikal med objekti;

Testni dražljaj: zanašal se je na ta globalni občutek. Pozoren je bil na to, če kaj ni v redu, če kaj ne deluje;

Globalni občutek: temelji tako na paslikah, kakor na nekem občutku prostora. Šel je od objekta do objekta i vsakega primerjal z globalnim občutkom ter paslik. To premikanje je bilo aktivno, primerjava s pasliko pa je bila pasivna;

36 S06-03-O-02

V vsakem primeru si je poskušal verbalizirati, ker meni, da je to najbolj učinkoviti način zapominjanja. To je počel aktivno;

Istočasno pa ostajajo paslike, ki jih vidi pasivno. Med pavzo jih poskuša ohranjati na zaslonu;

Te paslike sicer vidi malo nad ekranom;

Glas, ki jih verbalizira, je njegov. Besede same poskuša izgovoriti čim bolj na kratko in ta govor je zelo zvezan z dražljajem samim. Govor doživlja kot aktiven;

Paslike so pasivne: meni, da prenašajo preveč informacij, da bi jih lahko aktivno ustvarjal ali si jih predstavljal;

Pavza: To doživljanje poskuša zaščitit pred drugimi mislimi; to se mu kaže kot močna koncentracija, obenem pa v tem verbalnem ohranjanju dražljaja iz paslik;

Jasno doživlja mejo svojega delovnega spomina, kar se mu kaže kot mešanica tesnobe, sramu in jeze. To so vse telesni občutki, ki jih doživlja kot zvijanje v želodcu, poleg tega pa čuti da mu zalije lica;

Pavza: ima občutek, da si je uspešno zapomnil dražljaj, kar je vedel, ker mu je med spominskim dražljajem uspelo priti skozi vse dražljaje, ob čemer čuti olajšanje in sproščenost;

37 S06-03-C-01

Zapomnil si je prvenstveno prek verbalizacij, ki so zavzemale obliko njegovega glasu. Ta je bil močan, prodoren in jasen;

Poleg tega se je zanašal na globalni vtis. Ni dejansko videl globalnega vtisa med pavzo, ampak je imel neko pričakovanje o tem, kako bo. To pričakovanje pa je imelo neko kvazi-vidno modaliteto;

Obenem se mu je kazalo pričakovanje, kdaj ga bom ustavil. To mu je sporočal nek tih, oddaljen glas ter stiskanje v trebuhu.

38 S06-03-C-02

Definitivno ne vidi paslik;

Verbalizacija;

Poleg tega ima globalni *feeling,* to je nek *gist,* ki se prelije iz pavze v testni dražljaj;

Testni dražljaj: *feeling,* da nekaj ni v redu;

Ob verbalizaciji vidi tudi barvo;

Testni dražljaj: primerja z globalnim občutkom, z barvami pa primerja samo, če je ta zbledel;

Vidi barve, ki vzniknejo ob verbalizaciji, obenem pa jih povleče iz globalnega občutka, kar pa terja določen napor.

39 S06-04-P-01

Lahko bi si izmišljeval, ker je opazil, da je našel v doživljanju nekaj, česar ni bilo.

Prostorsko se pomika po vsakem dražljaju, kot da bi premaknil pogled, ampak ga ne premakne. Premika se s pozornostjo.

Spominski dražljaj: pogleda vse objekte;

Pavza: ponovi, kar je videl.

Pri tem je nek zvočni element: »tp, tp, tp, tp.« torej jih pogleda diskretno. Kakor da se v mislih prestavi do objektov. Ob tem začuti občutek v predelu trebuhu, na katerega se zanaša, če je objekt na pravem mestu.

Pavza: Prostorsko pomikanje po pikah: to doživljanje sploh ni bilo vizualno. Samo v telesu, bolj pa v glavi pa je imel podoben občutek kot da bi se premikal po prostoru, le da je to počel v mislih. Medtem so njegove oči mirovale. Njegov fokus je bil globalno na območju, kjer se pojavljajo objekti. Njegova pozornost je zaobjela celoten ta prostor in osredotočal se je na Gestalt tega prostora. Obenem se niti ni osredotočal na to, kaj vidi pred sabo. Bil je totalno v svojih mislih.

Pavza: Spominski dražljaj: konkretno je videl izven fokusa, v smislu da je bilo neizostreno. Ob tem se je z neko notranjo, mentalno pozornostjo pomikal prek pik. Ob tem je imel nek prostorski *feeling,* pike same pa je doživljal diskretno. V resnici sploh ni imel vidne predstave o teh pikah. Ni utvarjal neke celosten reprezentacije. Premikal se je od pike do pike in vedno ko je videl novo, se je zavedal le te; ostale so izginile.

Pavza: Premik notranje pozornosti je nekaj, kar je zavestno počel;

Pavza:: akustična kvaliteta.

40 S06-04-P-02

Spominski dražljaj: Vsakega posebej je pogledal ločeno in nekako se mu je ustvaril občutek v glavi, da je na tem mestu pika. Ta občutek je bil to, da so se mu v področju glave prostorsko umestile pike. To se je dogajalo v nekem mentalnem prostoru, ki se nahaja nekako za očmi;

Te mentalne podobe je istočasno začutil v mentalnem prostoru; dodajale so se tja. To je bil precej mentalen občutek; zelo jasno lahko čuti lokacijo tega;

Te mentalne podobe so bile nek občutek, ki je bil kot rahel pritisk na določenem delu glave;

Ima občutek, da so se te podobe ustvarjale avtomatsko;

Spominski dražljaj: udeleženec je vedel, da si bo lahko zapomnil dražljaje. Te mentalni občutki prostora so bili dovolj močni, dovolj distinktivni, da je vedel, da si jih bo lahko zapomnil (prim. *vmes so mu enkrat odtavale misli in ni imel diskretnih, posameznih občutkov in te gotovosti ni bilo*);

Vedel je, da bodo ostali v spominu: iz tega, da so bili dovolj močni je dobil nek občutek sigurnosti, kar se mu je kazalo, tako da je občutil nekakšno pomirjenost (deloma telesno, v trebuhu, obenem pa se je zanašal na občutek v glavi, pri katerem razlikuje med neko prisotnostjo ali tem, da je vse pomešano; če se pojavi nek vzorec) in samozavest;

Spominski dražljaj: pomikal se je od dražljaja do dražljaja. Takrat se še nič ni konkretno ustvarilo, vse je bilo precej neopredeljivo, ne zna ga opredelit (ker je ta občutek zelo avtomatičen), ko pa enkrat poskuša ponovit, ga lahko obnovit. Sprva ni bilo neke reprezentacije, ki bi jo lahko zagrabil. Končni spomin je vsrkal pred pozornosti. Dokler ni ponovil tega, ni bilo ničesar tam;

Pavza: imel je nekaj v sebi, kar se mu ni na poseben način kazalo, ampak je bilo tam in potem se je lahko pomaknil prek teh občutkov prostora in tako obnovil vzorec. V mentalnem prostoru se je premaknil po prostorih, premikal se je z mislimi, ni bilo vidne komponente, ob tem pa je ob vsakem premiku zaslišal »tm.«

Vedel je, da so te prostorski občutki tam, še preden jih je dejansko poskušal razpreti. Ta občutek je bil prisoten in bil je različen od občutka, ko tega ni imel, vendar ne ve točno, kako se mu je to kazalo. Občutil je nekaj v trebuhu. Je nek občutek, ki ga zagrabi;

Prostorske občutke je enega za drugim dodajal v mentalni prostor, potem pa so istočasno ostale tam (iste kot v S06-04-P-01);

On se je zavestno premikal po prostorskih občutkov, ko pa je točno na tistem mestu nekaj nastalo, se mu je dogajalo, zgodilo se je samo od sebe. Za vse ostalo pa je imel močan občutek agence in avtonomije.

Akustična komponenta: »tm, tm, tm, tm.« to je bil njegov glas, zadušen in ni prepričan, ali se je pojavil sam od sebe, ali ga je on govoril. Bilo je zelo na meji. Ima zelo močan občutek, da celotno stvar on kontrolira (ne opazuje, ampak dela; ima občutek, da on manipulira s tem), vendar je ta zvok zraven pomožno sam od sebe.

Pavza: imel je nek občutek, da je ta podoba, ki jo je ustvarjal ustrezala spominskemu dražljaju, vendar ne ve, kako se mu je kazal. Te občutki so tako jasno, da ko jih ni, ve, da si ni zapomnil.

41 S06-04-P-03

Malce je bil utrujen in je čutil, da ga v ozadju pečejo oči in to je spremljalo njegovo doživljanje;

Spominski dražljaj: pomikal se je od ene do druge pike in ob tem si je vizualiziral nekakšno linijo.

Ob spominskem dražljaju se mu je v trebuhu pojavil kanček tesnobnosti zaradi utrujenosti, obenem pa mu je begala koncentracija;

Pavza: obnovil je spominski dražljaj, pomikal se je skozi prostorske občutke in poskušal ojačat te lokacije. Zdi se mu, da tisto, skozi kar se najprej pomika po objektu v spominskem dražljaju, ostane nek vtis, potem pa se on pomakne skozi njih in jih spremeni v nekaj zelo fizičnega v mentalnem prostoru. Vtis je bil čisto nekaj malo vezanega na pasliko na ekranu, ki je hitro zbledela, za njo pa je ostalo nekaj zelo medlega (to je bilo zelo pasivno), potem pa se je z mislijo pomaknil tja in ob tem samem gibu se je to fizično ustvarilo samo od sebe; te mentalni premiki so bili zavestni, občutek pa je bil pasiven; te občutki niso bile konkretne podobe, ampak je bilo samo, da je nekaj tam. Tega ni videl, bilo je prostorsko umeščeno in ima neko fizično težo, nek pritisk;

Testni dražljaj: še enkrat se je premaknil skozi te prostorske občutke in če se je moral premakniti v neko drugo smer zaradi objekte na ekranu, je vedel, da je testni dražljaj napačen;

Pavza: sploh ni bil pozoren na zunanje dražljaje ampak le na svoj notranji mentalni prostor;

Risanje črt med spominskim dražljajem: v mislih je od enega do drugega ustvaril črno linijo. Ta linija je bila črna, ni imela debeline, videl jo je na ekranu. Črta se ni kar pojavila, ampak jo je vlekel. Ta črta se je pojavila sama od sebe;

Testni dražljaj: ko se je pojavila posamezna pika, se je takoj za tem mentalno v notranjem prostoru premaknil v prvi del tega zapomnjenega zaporedja in če se je ta premik ujemal z dejanskim premikom od fiksacijskega križa do prvega objekta, je bilo okej, potem je nadaljeval, sicer pa je začutil, da se ne ujema in neujamenje je sporočilo, da ni v redu;

Ujemanje: (mogoče) je v mislih primerjal objekt v mislih in tega na ekranu. Najprej je pogledal objekt na ekranu, potem pa še v mislih in če sta se občutka ujemala;

Tesnoba: čutil jo je na začetku, potem pa ne ve. Meni, da se mu ni kazala. Občutek utrujenost pa je obstal. Najbolj je bil opazen na začetku, potem pa je to pomnjenje prevzelo večinski del njegovih kapacitet in pozornosti in utrujenost je malo izzvenel. Vsekakor pa je bil v manjši meri prisoten. Kazal se mu je pekoč občutek za očmi. To je bil zelo telesen občutek.

42 S07-02-P-01

V glavi si je govorila »in en in dva in tri in štri,« in (mogoče) si je med paslikami izrisala črte. Ko je prišlo do testnega dražljaja, je imela nek občutek, da je ali vse v redu sledila, ali pa da je nekaj odstopala;

Njene verbalizacije so bile vedno izgovorjene z isto intonacijo;

Risanje črt: niso bile zares črte, ampak se ji je zdelo, da se je bolj prosojna različica tega kroga potegnila v smer naslednjega;

Z očmi je sledila tej usmeritvi; zdi se ji, da ta *opacity* ni bila vizualna, ampak je bila bolj prostorska pot, ki jo je vlekla z očmi;

Ustrezanje med testnim dražljajem: če je bil testni dražljaj enak, ga je spremljal občutek prijetnosti. Več ko se pik ujema, boljša je kontinuiteta teh občutji;

Če pa nek dražljaj ni enak, potem se ta kontinuiteta prekine, poleg tega pa ima tudi nek bolj zavesten občutek, da nekaj ni na pravem mestu;

Prijetnost ob ujemanju: telesen občutek v grlu in prsih;

Odstopanje je bilo manj telesno, ampak je šlo bolj za prekinitev tega občutka, ki mu je sledilo ozaveščenje nekega *errorja;* zdi se ji, da je prisotna bolj intelektualna obdelava, kje bi objekt v resnici moral biti. En vidik tega je, da dokler sledi temu, kjer bi objekti morali biti in so, potem pa nekam pogleda, kjer bi naslednji objekt moral biti in ga ni tam. Soočenje s to belino podaja popolnoma vizualen signal, da je nekaj narobe, njena pozornost pa se preusmeri tja, kjer dražljaj dejansko je;

Pavza: med pavzo je imela zarisano neko pot, nek zemljevid. To se ji je kazalo kot neke pike, ki so ostale, potem pa jih je razmazala do naslednje pike. Ta vmesni del je bolj prostorski. (mogoče)

Pritisk na tipko: v veliki meri je izven njenega doživljanja. Dogaja se ji, da je prepričana v odgovor, če pa klikne narobe, jo nekaj zmoti. Velikokrat se ji to zgodi, ker pritisne napačno tipko. Prepričanost v odgovor se ji kaže kot občutek, da velika verjetnost, da si je zapomnila, oz. da ve, da je posvečala pozornost nalogi.

Pavza: občasno si je ponovila dražljaj. Takrat si je notranja izrekla »in en in dva in tri in štri,« s pogledom pa je po vrsti sledila, kako naj bi se prikazali te dražljaji. Pri ponavljanjih paslike ne izginejo, ampak ostanejo tam in dobi nek celosten vzorec;

Ta celostni vzorec je slika v njeni glavi, ki jo spremlja nek občutek, ki je zelo vezan na neko gibanje, ki se zelo jasno kaže v verbalizaciji »in en in dva in tri in štiri.« Čuti, da se ta številka pripiše enemu izmed teh objektov, s čemer ga bolje definira.

S07-02-P-02

Spominski dražljaj: vtisnila si je neko kinestetično stvar, kinestetično linijo, ki ni vidna. Zdelo se ji je, da so se njene oči premikale počasi in zarisale to vidno pot, kakršna je bila v prejšnjem primeru, poleg tega pa je bilo še nekaj nevidnega, ki se je ustvarilo hitreje;

Med pavzo je mentalno v glavi ponavljala te gibe (*občutek ima, da teh ponovitev ni delala med vsakim primerom*);

Te gibi so bili premiki pozornosti in zaradi tega se ji je zdelo, da je videla neko povezano stvar;

Kinestetične linije vleče od kroga do kroga in te gibi so včasih ravni, včasih pa jih povleče na tak način, da ne trčijo ob krog, ampak se malo podaljša onkraj njega;

Ponovno je bila prisotna verbalizacija »en in dva in tri in štir,« ki udeleženko spominja na plesni ritem; v temu zvoku je prisoten tako ritem čez vokalizacije, obenem pa v »x in« zasliši premik čez celotno oktavo;

Udeleženka je bila zelo prepričana v to, kakšna je pot. Prepričanost se ji je kazala kot to, da ni le vedela, ampak da bi lahko celo narisala vzorec, ne le da bi ga lahko prepoznala (*lahko bi ga rekonstruirala*);

Ta potencialna rekonstrukcija ni bila vizualno prisotna v njeni zavesti, ampak je imela občutek, da jo *lahko* nariše in da *ve,* kje so te pike;

Lokacije za katere je vedela, da so tam pike je včasih (mogoče) doživljala tako, da jih je malce celo videla, vendar udeleženka poroča, da mogoče to dodaja med intervjujem;

Ena oblika je bila podobna metulju, kar je doživljala tako, da je doživela vizualno asociacijo metulja, ki s sabo nosi neke reči, obenem pa je doživljala nekaj nežnega. Ko je videla podobo si je vzkliknila, da je to še ena stvar, prek katere si jo lahko zapomni. To pa je bil še en dodaten element gotovosti. V tem ni bilo nobene verbalizacije.

43 S07-02-P-03

Opomba: Udeleženko sem prekinil med pavzo;

Pričakovala je, da jo bom ustavil;

Ponovno je slišala »en in dva in tri in štiri;«

Če se je umirila, si je zapomnila kinestetične črte, ki jih je vlekla s pogledom, obenem pa je razvlekla pasliko, ki je ostala za vzorcem. To sliko je deloma videla na ekranu, vendar se je malo premikala;

To premikanje je bilo kakor da bi videla narisano sivo stvar, ki se potem malo omehča in zaplava; bilo je, kakor da bi slikala plavala na vodi, slika sama pa je bledela s časom, ob naslednjem primeru pa je popolnoma zbledela;

Med pavzo je pričakovala, kje se bo pojavila prva točka. Vsa njena pozornost je bila usmerjena na tisto mesto, kjer se je običajno pojavila in če se je, je doživela prijeten občutek potrditve;

Prijeten občutek potrditve: to je bil tako mentalen, kakor telesni občutek (najbolj izrazito v grlu in prsih);

Kar je ugotovila, je, da je ob pritisku, da je slika ista, ta slika, ki jo je ustvarjala, še vedno ostala na ekranu. Slika, ki je ostala, je bila nekako močnejša od tiste, ki jo je videla med pavzo;

Paslika: prva paslika (po spominskem dražljaju) je bila manj močna, tista, ki ostane po testnem dražljaju pa je obstala le, če sta bila dražljaja skladna, sama po sebi pa je bila močnejša;

Ko je povezovala sliko v vzorec, ni bilo nujno, da je bila ta slika zaključena;

Pike so se povezale v tisti smeri, kjer je naredila gib s pozornostjo. Ta gib sam je nekaj, kar ona počne, vendar ne zavestno. Počuti se, kakor da je avtorica tega giba. To ni nekaj, kar ona dovoli. Zdi se ji, da je kakor ko naredi gib z roko, vendar da je bila v igri neka druga okončina, niti ni to obraz, ampak je nekaj kar vodi ta gib, je kot neko orodje, ki ga vodi ona;

44 S07-03-C-01

Spominski dražljaj: na nek način vidi te štiri pike, ne vidi jih zares posamično. Zdi se ji, da je hotela, da jih vidi kot neke lastnosti (npr. bolj močne, pastelne, nežne). Če jih je grupirala po neki lastnosti, je močneje zaznala razliko, oziroma je bila ta zelo očitna;

Videla je vse barve;

Pavza: Zdi se ji, da vmes ni nič ponavljala, vendar je včasih imela občutek, da bi jih lahko poimenovala, tudi če si jih med spominskim dražljajem ne bi zares dobro ogledala;

Odstopanje: ne ve, ali je to miselni konstrukt ali je to nek občutek; zdi se ji, da je malce obojega → ni videla posameznih barv, ampak je videla nek skupek. To ni bil prostorski vzorec, ampak bolj nek vzorec barv. Iskala je neko nadpomenko za te štiri barve. To je bilo vizualno doživljanje v smislu, da

je prepoznala neke skupne lastnosti in te skupne lastnosti so ji ostale v spominu. To je doživljala kot neko esenco teh štirih barv, iz česar ni mogla izluščiti posameznih barv; tega občutka ni ustvarila zavestno, ampak gre bolj za nek način gledanja, ki ga ima;

Katerakoli esenca teh štirih barv je bila zadostni močan spomin, da ji je pozornost med testnim dražljajem avtomatično padla na tisto, ki ni ustrezala;

To je bilo razločljivo drugačno doživljanje od vizualne zapomnitve; vizualni del je doživljala ne tako, da jih je konkretno videla, ampak je šlo za vedenje o tem, katere barve so bile, doživljala je občutke barv, ki bi jim lahko pripisala občutke (za razliko od esence);

Esenca je bila vedno prisotna, včasih pa je nad to esenco doživljala tudi ta vizualno občutek barv;

Pozornost je bila lahkotna;

Včasih se spomnila, da bi na nekem mestu med testnim dražljajem morala biti neka druga barva: gledala je naravnost v ta objekt, ker jo je zmotil in zaradi tega je nekako vedela, da je pred njo napačna barva, da bi morala biti neka druga. (mogoče) je celo videla to barvo;

Meta-spomin: imela je občutek, da si je nalogo dobro zapomnila. To se ji je kazalo tako, da je včasih med pavzo a) lahko rekonstruirala dražljaj (ni bilo nujno, da jih je dejansko poimenovala, ampak je bilo dovolj, da je doživljala že sam potencial za poimenovanje). Tudi če se ne trudi in ni vse pod njenim nadzorom je dražljaje lahkotno zaobjemala. V tej lahkotnosti je bila neka pomirjenost, zadovoljstvo, kar se ji je kazalo kot nek telesni in mentalni občutek (prim. s prisiljeno pozornostjo, kjer je tako foksuirana, da včasih pozabi na širšo sliko);

Med reševanjem naloge se ne zaveda svoje utelešenosti;

Testni dražljaj: odločitev o odgovoru je doživljala kot izstopanje nečesar in da če ni izstopalo, je ob tem čutila zadovoljstvo. Če se nekaj ujema ima prvi *response,* če pa je nekaj drugače, mora tudi ona spremeniti svoje vedenje. S spremembo v telesu (prst za odgovor ima na gumbu za enakost) označuje spremembo v dražljajih;

Dejanje odgovora: to doživljanje je bilo zelo različno. Če je samo kliknila, se je ustrašila, da se bo morda pogosto zmotila, kakor se je v gornjih fazah naloge. zavestno je morala preveriti, kakšen bo njen odgovor. Včasih si to izgovori (»da, »ne«), da ve, kateri gumb mora pritisniti;

45 S07-03-C-02

Spominski dražljaj: med nalogo je ugotovila, da ji ni treba posvečati toliko pozornosti nalogi. Lahko si ne poskuša aktivno zapomniti naloge, ampak jo lahko samo sprejme in se barve vtisnejo vanjo;

Enkrat se ji je zgodilo, da če so spremembe bolj pretanjene, jih v tem slučaju ne opazi, saj ni občutljiva na neke barve, ki so si zelo podobne (npr. vijolična in roza);

Spet doživlja neko esenco barv, kar doživlja kot nabor štirih barv. Ne vidi jih dejansko vsake posamezne, ampak jih spremlja neko poznavanje teh barv. V tem gre za nekaj relacijskega, v smislu,

da so tam notri rumena in neke ostale nežne barve, ni pa nekih sivih ali bolj intenzivnih barv. Gre za neko osnovno oznako (svetle barve, kontrastne barve), to pa se dopolni s tem, katere barve so dejansko bile;

Če so vse barve res zelo neočitne, jih ta kategorija le približno ozavesti, če pa kategorija ni pomembna, si barve zapomni bolj izrecno (prepozna barvo, vendar je nikoli ne izgovori, ampak jo na pretanjeni ravni registrira kot to dotično barvo; to ni verbalizacija, ampak je neka vizualizacija barve);

To pripisovanje kategorij barvam je bolj široko doživljanje, medtem ko je konkretna barva ožje. Široko na način, da je bolj avtomatsko. Ne rabi te pozornostne usmeritve. Njena pozornost je tako razpršena, da lahko vse zaobjema. Če pa bi si izrecno zapomnila neke barve, se pozornost za trenutek bolj zašpiči vanje in to je nekaj, kar ona počne, vendar to spremlja občutek, da se ji to dogaja.

Meta-kognicija: imela je občutek, da je dobro reševala, ta občutek pa je usahnil, kadar je nalogi podajal manj pozornosti. Takrat je pomislila, da je zgrešila kako manjšo razliko. To je bila konkretna zverbalizirana misel. Do tega je tudi prišlo, kadar je razmišljala o doživljanju, kar se je potem prelevilo v razmišljanje o tem, da je mogoče zgrešila kakšno stvar. Obenem ima občutek, da je vizualno zaznala spremembo, ampak ji ni dala toliko pozornosti in je posledično prevladal občutek, da je vse približno isto. Torej je zaradi pomanjkanja pozornosti nek objekt zlit v esenco preostalih dražljajev. Odgovorila je, da sta dražljaja enaka, obenem pa je razmišljala o tem, da je zaznala neko razliko.

To razmišljanje je bilo na pol verbalizirano: v tem razmišljanju ni bilo telesnosti, ampak je bil poudarek na toku misli. Te misli si je udeleženka (recimo da) govorila, vendar v tem govoru ni bilo ust. Ne sliši fonemov, niti ni zgolj pomen. To niso zelo abstraktne misli. Zaveda se nekega govora, ki nima glasu. Če že, je to njen glas, vendar ne zelo izrecno. Ima le občutek, da so to njene misli. S tem tokom misli se poistoveti in doživlja, da ga aktivno proizvaja;

Del pozornosti je bil usmerjen na njeno opazovanje doživljanja: to je bilo malce povezano s tem miselnim tokom. Ni bilo prisotno ves čas, sploh kadar se je močno osredotočala na nalogo. Kadar pa je njena pozornost padla stran z naloge, je bilo več notranjega komentarja, katerega vsebina je bilo neko dojemanje sprotnega dogajanja (»Aha, zdaj imam pa manj pozornosti na nalogi.« »Zdaj pa lahko odgovorim z 'ja'.«). Ta komentar je bil oblikovan v tem istem pol-verbaliziranem miselnem toku.

46 S07-03-O-01

Dejansko je imela neko sliko v glavi;

Videla je dražljaj in potem ponovila te stvari in jih povezala. Dražljaj je izginil in med pavzo ni imela neke poti, ki bi bila temporalna, kakor pri poziciji, ampak si je bilo to pot težje zapomnit, ker je izginjala. Poskušala se je zapomnit, kje je bila prva smer, včasih si je celo rekla v glavi (»gor, dol«);

Videla je paslike, ampak je ugotovila, da skoraj pri nobeni nalogi ne pomagajo zares, ker so zelo *blurry,* razen morda pri poziciji. Pri tej nalogi ji je bilo težje iz njih razbrati obliko;

Testni dražljaj: ni imela res dobrega občutka, kaj je bilo prej. Sledila je nekemu občutku, ali se nekaj ujema, ali ne.

Med testnim dražljajem je podvomila, ali se je pravilno spomnila. Zdi se ji, da je videla spremembo, kjer je ni bilo.

To nalogo je doživljala kinestetično. Vmes je premikala glavo in zdi se ji, da bi jo lahko premikala več in si tako bolje zapomnila. To so zavestni premiki.

Verbalizacije: večinoma si to pove pri zadnjem, ker tako poskuša priklicat prvega iz te poti. Zdi se ji, da ji verbalizacije podajo še en način priklica. Ima občutek, da mora še enkrat ponoviti, da se zasigura.

Občutek ujemanja: bil je zelo pretanjen, ker je bila zelo neprepričana. Ker se ji zdi, da ne gre več samo za nek zavestni spomin, ampak pri težjih nalogah gre za nek občutek, ki ga nima zares ozaveščenega, ampak sumi, da je mogoče tam. Ta sum se ji kaže popolnoma zavestno. Prepriča se, da če je nekaj videla in če zraven nekaj naredila z glavo in ob tem dobila nek občutek, s tem da ne more v spominu preveriti, kakšen je ta občutek, še vedno lahko sledi temu občutku (ki je mentalen). Ob tej strategiji ji je malce neprijetno (ki jo čuti telesno). Kjer bi morala imeti jasen odgovor, ima nekaj neotipljivega. to da ne more seči v ta občutek in dobiti jasnih spominov je zvezano s tem telesnim občutkom, ni neka svoja stvar. Je neka praznina. Hkrati pa nekaj le je v zraku in se mora zanašati na to, ne pa na dejansko stvar, ki bi bila otipljiva.

Paslike: pri tej nalogi so ji bile malce moteče.

47 S07-03-O-02

Ni bilo dovolj, da premika samo oči. V glavi nima te kombinacije krogca in črte, ampak samo črto, samo usmerjenost. Njeni premiki so ponovno zaporedni, pogleda vsak objekt posebej;

Včasih se je namerno vračala nazaj k objektom, da jih je še enkrat preverila;

Poleg tega pa si zelo jasno poskuša zapomnit vsaj enega, da vsaj za enega z gotovostjo ve, da je točna, čeprav ji to mogoče jemlje spomin, ki bi ga lahko pridobila iz tega občutka celote;

Včasih ta pot ni samo premik oči, ampak doda neko verbalizacijo, ali pa si jo vizualno bolj vtisne, en samo kot pot, ampak kot sam objekt. Torej tej kinestetični poti za kak objekt doda še neko dodatno informacijo.

Ve, da ko bo napočil testni dražljaj, da bo videla krog-črto, ne le črte, ampak ji je dovolj, da se med spominskim dražljajem osredotoča le na črto. Tudi tako si lahko zapomni usmeritev.

Poleg tega še kdaj poskuša kaj utrditi, da bo ustvarila neko točko jasnosti v tej kinestetični poti, ker se ji včasih zdi, da če sledi tem črtam, sploh ne ve, kaj je videla. Ne vem, ali je prav, ali narobe, kar se ji kaže kot neka praznina.

Preveri dražljaj: to je zelo zavestno, ampak ne na ta način, da bi se vedno znova odločila za to. Na neki točki ji je to postalo del procesa in ni več to zelo izrecno zavestno dejanje. Je le neko iskanje sigurnosti. To je kvalitativno podobno (mogoče) podobno *flowu,* ki ga je čutila med barvami, samo da je tam čutila neko lagodnost, da nikoli ni bilo praznine, tu pa je bila ta praznina prisotna in *flow* je bil prisoten le kot nek avtomatiziran postopek, ni pa bilo občutka, da ve, kaj dela. Praznina je bila zelo povezana s tem, da ne ve.

48 S07-04-P-01

Spet je povezovala črte;

Ponovno je slišala nek zvok, ki je bil kvazi-verbaliziran: »in en in dva in tri in štri.« Na to je bil zvezan nek ritem;

Oči je prestavljala iz pike na piko. Ne ve, ali je šlo za gibe s pozornostjo, ali z očmi. (Mogoče) je šlo za premik pozornosti, zvezan s premikom oči;

Pozornost je doživljala prek tega, da je videla premik pike;

Premiki oči so se dogajali pol avtomatsko pol voljno, ker pa je imela v glavi vebralizacije, se jih je zavestno zavedala (pričakovala je nek premik, ki ga je uskladila z ritmom). To pričakovanje je doživljala kot da neka naloga je in da se bo zgodilo nekaj povezanega z njo; ni pričakovala, da se lahko na ekranu pojavi nekaj popolnoma drugačnega);

Vedenje, kar naloga je: gre za nekaj, kar lahko ubesedi, vendar v doživljanju ni prisotnega nič deklarativnega; je nekaj, kar bi lahko povezala in izgovorila, ampak ni ji treba tega storiti, da razume nalogo; ta *tam* se ne nanaša na neko lokacijo v njenem notranjem svetu, niti ne gre za neko konkretno predstavo. Bolj gre za to, da je kot neka tema, v kateri je luč, za katero približno ve, kaj vsebuje, oziroma kako se ji mora približat, da ve, kaj je v njej, vendar če ne pogleda tja, tega ne bo zaznavala. Terja neko aktivnost (pogled), da se je ove; na tak način se je zavedala zakonitosti te naloge;

Pričakovanje naslednje pike: ni vedela, kje se bodo pojavile, šele ko se je dejansko pojavila naslednja pika, se jo je zavedala; ni poskušala napovedati, kje bodo, vendar je bilo vmes vseeno dovolj časa, da se je izrisala ta pot. Ko se je pojavila druga pika, sta se te dve povezali. Obenem pa ji je bilo jasno, kje je bila prva pika. Tega ni doživljala deklarativno, ampak je videla te pasliko.

V tem kontekstu se paslika nanaša na to, da ko se je ta vzorec premikal, je bilo za njim najprej belo, potem pa je z očmi poskušala zarisati pot, ki se ji je malce pokazala. To je bilo nekaj med konkretno vizualno izkušnjo in mentalno predstavo. Obstajal je vsaj rahel mentalni občutek.

Pojavitev naslednje pike: fokus ni bil na sami piki, ampak na njeni poziciji v prostoru in takoj se je zarisala ta mentalna pot med prvo in naslednjo piko;

Mentalno pot težko opiše, ker jo povezuje s pasliko. Paslika se ji je zdela kot kaplja barve, ki jo razmažeš do naslednje, kakor da bi raztegnila center barve. To je bilo zelo rahlo in ni bilo vizualno izrecno. Tesno je bilo zvezano s premikom oči in relacijo dveh pik v prostoru;

Med pavzo je še enkrat opravilo pot med pikami. Ta pot med spominskim dražljajem ni bila vidna, med ponavljanjem pa je postalo močnejše (tako paslika pik kot poti). S tem da so bile pike bolj ven iz sivosti ekrana. Zelo jasno je doživljala pot med pikami, pike same pa so bile zvezen s tem vokaliziranim ritmom (»en in dva in tri in štiri.«);

Spominski dražljaj: »en« je povezan s tem, da je določena pika na nekem mestu. »in dva« je vpeta med dva ritma in dve vizualni podobi. Ne ve, če je to verbalizacijo ponovno slišala med pavzo.

Testni dražljaj: takoj je prepoznala, če je neka pika odstopala od te poti;

Odstopanje od poti: videla je piko, potem pa je z očmi skočila tja, kjer je pričakovala naslednjo piko in če se je ta pojavila drugje, kot kjer bi se mogla, je to zaznala kot neujemanje. To se ji je kazalo lokacijsko – pika ni bila na piki, ampak je bila nova točka, izven te poti, kjer bi morala biti (ta pot se ni na novo izrisala, ampak je približno vedela, kakšna je celotna pot). Meni, da ne bi vedela, če bi neka pika padla v pot, vendar v drugačnem časovnem zaporedju;

Med testnim dražljajem ni bilo zvočne komponente;

Spominski dražljaj: vidi te štiri pike, kar je spremljala verbalizacija, ki sicer niso bile zelo zavestne. Bilo je bolj, kot da pustijo okus na ustih. Ni zaznavala jezika, bilo je zgolj v glavi. Verbalizacije so se ji tako počele, kakor dogajale – odvisno od primera (čuti, da je ta del nje utečen in ni del, ki bi ga aktivno usmerjala, kljub temu da je vedela, da to počne ona);

Vedenje, da ima pravilen odgovor: nekajkrat ni bila popolnoma prepričana, ampak se ji je vseeno zdelo, da čim pika ni ustrezala, je prišlo do neke prekinitve. Bolj nesigurna je bila, če je bila pot ista, kakor si jo je zapomnila, ker je bilo to odstopanje bolj izrazit znak, da nekaj ni okej, kakor kot ko je nekaj isto. Ob istosti je večkrat imela dvom, da nečesa ni zaznala;

Prekinitev procesa ob neujemanju: to je obenem početje in dogajanje. Zgodilo se je samo od sebe, istočasno pa ji je bilo ugodno spustit. To ugodje se ji je kazalo kot neko zadovoljstvo, olajšanje, ki je bilo kot neko zadovoljstvo ob koheretnosti oz. videnju vzorca. To je bilo predvsem mentalno izkiustvo z blagim občutkom v zgornjem prsnem delu;

Tekom celotnega eksperimenta je udeleženka v prsih čutila manjšo tesnobnost, ki pa je le redko vstopila v njeno zavest. Vmes se ji doživljanje ni preusmerilo na to tesnobo.

Meta-kognicija: na začetku se ji je zdelo, da bo lahka naloga, vendar se je potem malce zmedla in ko je začela se ji je zdelo, da bi jo lahko bolje reševala. Sprva ni bila najbolj prepričana, to pa je do neke mere usahnilo tekom reševanja naloge.

49 S07-04-P-02

Malce je premišljevala o tem, kdaj jo bom ustavil. Pomislila je, da jo bom ustavil. To ni bilo verbalizirano, ampak je bila zgolj misel. Ta misel je vzniknila sama od sebe in je ni priklicala;

Zdi se ji, da zaradi tesnobnosti in utrujenosti nima tako jasnega videnja doživljanja, malce vatasto se počuti, kakor da bi bilo vse zavito v kosme vate. Ni tako ostrih misli, kar se ji kaže tako da se vsake toliko zave, da se dogajajo reči, ki jih ne sprocesira;

Njene verbalizacije ob objektih je voljno poskušala zamenjati z nekim *chantanjem:* »tam, ta tam …« kar se ji je zdelo težjo. Opazila je, da je neka diferenciacija v tem plesnem ritmu s številkami, zaradi česar ji je lažje reševati nalogo. Brez njih je težje vedeti, kje začeti;

Vmes se je spominjala reševanje prejšnje naloge, ki se je dogajalo, vendar je to imela pod nadzorom (to spominjanje bi do neke mere lahko umaknila proč). Te spomini so se ji kazali kot da je tekom reševanja lahko vzporedno nekaj počela. Še vedno je lahko izvajala nalogo, tudi če ni bila nanjo pozorna ves čas. Vseeno se ji zdi, da to ne bi bila dva enaka tokova. Imela je neke misli, na katere je bila pozorna nekaj časa, potem pa je pozornost preusmerila na nalogo, in potem spet nazaj na misli. Nihala je med tema tokovoma;

Videla je paslike in zdelo se ji je, da je to dovolj za reševanje naloge;

Ponovno si je verbalizirala, vendar le med spominskim dražljajem in pavzo, ne pa tudi med testnim dražljajem;

Pike so se povezale v nek skupni vzorec prek te kinestetične poti;

Med pavzo je ponovila to pot, s tem da je videla rahlo pasliko te pike;

Med testnim dražljajem je sledila ponovnemu ponavljanju in opazovala splošno ujemanje s to pasliko;

Splošno ujemanje: to ji je bilo pomembneje, kadar se je vse ujemalo. Kadar se nekaj ni ujemalo, ko pika ni sledila začrtani poti, ji splošno ujemanje ni bilo več tako zelo pomembno. Pri popolnem ujemanju na koncu še enkrat preveri, če je vse v redu, ob čemer dobi zelo hipen občutek, ali je to, kar vidi, ustrezno. Ni pogledala posameznih pik, ampak je preverjala celoto, če je znotraj teh kotov, ki so postavljeni v tem liku. To je zelo mentalen *feeling,* v njem ni bilo nekega zadovoljstva, oziroma je bilo to zadovoljstvo zelo blago (prim. z neujemanjem, kjer je bilo to zadovoljstvo bolj intenzivno);

50 S10-01-C-01

Imel je dve vzporedni strategiji pomnjenja: pri eni si je govoril imena barv: »črna, modra, roza, bela.« Govoril si jih je v različnih zaporedjih (vedno je začel zgoraj levo, potem pa se je premikal ali dol, ali pa desno). Prvo barvo je vedno izgovoril v nekem relativno normalnem notranjem glasu, proti zadnji pa je ta artikulacija postala bolj zamomljana;

Glas mu je zbledel: to je bilo tako, da si ni moral dovolj hitro govoriti besed in je tako govoril vedno hitreje;

Glas je bil njegov, kakor vedno ko si govori stavke v glavi;

Druga strategija: skakanje z očmi od ene do druge; ob tem si ni zares ustvarjal nekega vtisa, ampak imel je neko pričakovanje, da ko bo prišel drugi dražljaj, da bo avtomatično vedel, ali ustreza;

Skakanje z očmi: v ozadju je imel neko pričakovanje, da se bo podoba avtomatično vtisnila v njegov vtis. Skakanje z očmi je počel, ozadnji vtis pa se mu je dogajal. Obenem je bil avtor gibov, po drugi strani pa so njegove oči avtomatično skočile na vsak objekt;

Pričakovanje vtisa: zaupanje, da se je nekaj vtisnilo. To zaupanje je bilo mentalno. Verjel je, da se mu je v glavi oblikovalo nekaj, kar bo lahko kasneje primerjal;

Testni dražljaj: doživljal ga je celostno; kakor je prvič skakal od ene točke do druge, si je tu ponovno izgovarjal besede, vendar so bile bolj nek odmev in niso bile vezane na to, kar je videl na ekranu. Bolj so bile vezane na ta vtis v ozadju. Pri prepoznavi se je tako bolj zanašal na ta ozadnji vtis;

Vtis: to je bil nekaj, kar je preprosto prišlo. To je bil nek mentalni občutek.

51 S10-01-C-02

Zaradi tega ker sem ga prekinil, je čutil odsekan občutek;

Kljub temu je pred tem čutil pričakovanje, da ga bom prekinil;

Pričakovanju je sledila pavza, potem sem ga dejansko prekinil in potem je doživel odsekanost;

Pričakovanje: vzporedno z reševanjem naloge in prevzema znaten del pozornosti. Če bi temu pričakoval več pozornosti, bi bilo reševanje naloge težje. To pričakovanje sestoji iz dveh stvari: nejasnega glasu + občutek anticipacije.

Ob reševanju naloge se udeleženec zgolj daleč v ozadju zaveda moje prisotnosti. To je neko vedenje;

52 S10-01-C-03 (fragmentarno)

Doživljanje barv: Razmišljal je o tem, ali je mogoče, da se menjata dražljaja, ki sta enaka in različna. Ta misel je bila verbalizirana v njegovem glasu. Ta glas je izviral iz njegove glave iz spodnjega dela.

Barve: imel je neko doživljanje, ki je bilo zelo avtomatizirano. Kakor da se je že malo navadil na nalogo in se je moral manj truditi zanjo, kakor na začetku;

Sprva si je govoril barve, medtem ko je delal krog z očmi. Ta gib je krožen in zvezen. Je njegov avtor v smislu, da aktivira ta gib, ta pa potem sam steče naprej. Ta aktivacija je konkreten odziv na dražljaj;

53 S10-01-C-04

Spominski dražljaj: vidi kopijo barv, čeravno ne more razbrati njihovega zaporedja. To je dvodimenzionalna podoba štirih jasnih slik na belem ozadju. Ta stika je na ekranu;

To podobo lahko vidi, če zapre oči;

Podoba sama hitro bledi;

Pozornost: je lahkotna. Čuti jo nekje nad čelom;

54 S10-01-C-05

Spominski dražljaj je izgubil;

Razmišljal je o več stvareh hkrati; ti tokovi so se prelivali drug v drugega. Ena misel je vzniknila, potem druga, nekaj časa sta se prekrivali in potem je ena prevladala, kar je bilo vezano na to, kateri je posvečal pozornost. Vsebina teh misli je bila malce slišna, vendar ne povsem. Zraven je bilo več stvari;

Narobe je začel pritikat, kar ga je vrglo iz ritma, po čemer se je malo moral spraviti nazaj v element, spet se je moral uskladiti z nalogo;

Ugotovitev, da je narobe pritiskal: ko se je enkrat zmotil, je ostala stalna nevarnost, da lahko pritisne na napačno tipko; to je bil v glavnem občutek, ki pa ga je občasno spremljal njegov glas; občutek je bil paničen. To je bila neka vzdraženost;

Ritem reševanja: po tem, ko je rešil nekaj primerov, se je njegovo reševanje avtomatiziralo in terjalo je minimalno aktivnost, da je reševal nalogo. Premik prstov je bil minimalen, njegove oči so avtomatično risale krog;

Zelo hitro je pritisnil po tem, ko se je pojavil drug dražljaj.

Premik prstov: to je nekaj, kar je on inciiral, ampak z veliko manj truda, kot na začetku reševanja naloge;

Zavedanje prstov: zavedal se jih je; iz tega da se ni zavedal ničesar, je njegovo doživljanje prešlo v to, da se je nenadoma zavedal nečesa tam. Malce se je zavedal oblike prstov, ki pa niso bili pravilni. Bilo so veliko bolj jajčasti;

55 S10-01-C-06

Spominski dražljaj: doživljal je vtis slike; bilo je zelo celostno. Ni bilo več glasu ali ponavljanja besed;

Celostnost dražljaja: zdi se mu, da zdaj samo še išče razliko, ne pa več toliko tega, kaj je na ekranu. Samo poskuša zadržati to, kar je videl v spominu in takoj, ko vidi, da se s to sliko kaj ne ujame, to zadrževanje razpade;

To ni bilo vizualno, ampak je bil le občutek. Iz tega občutka ne bi mogel izvleči posameznih barv;

Pavza: Bil je le pripravljen na nadaljnjo nalogo;

Meta-kognicija: imel je nek občutek, neko utež, kako dobro si je zapomnil spominski dražljaj. To je doživljal kot zelo minimalen občutek sigurnosti, ki ga je doživljal nekje v glavi;

56 S10-01-O-01

Naloga mu je bila težja kot prej; na nalogo je moral usmerjati več fokusa. S tem stran potiska neke stvari, ki niso povezane z nalogo samo. Izražene so neke misli, ki se prikazujejo in nima občutka, da jih on tvori.

S pogledom je delal gib prek vseh objektov; skakal je od objekta do objekta.

Opazil je, da mu za zadnji objekt zmanjka časa.

Zadnji objekt med spominskim dražljajem mu izgine. Naredi sakado nanj, ampak ga ne ujame;

Premika oči in pozornost;

Premiki: on ga sproži, potem se premiki dogajajo avtomatsko, ampak gibe same pa spremlja;

Ko usmeri pozornost na posamezen objekt, si ga poskuša zapomniti tako, da hoče ustvari nek *inprint*. Ta vtis je manj vizualen, kakor pred barvami, ampak si ga še vedno lahko predstavlja kot vizualno podobo. Ta je manj jasna, obenem pa so relacije med objekti drugačne. Tu gre za neko usmeritev in za razliko od barv ni vseeno, kako so postavljeni;

Prostor je tu bolj pomemben, zaradi česar ga v glavi naredi bolj prožnega. Lahko ga manipulira, kar doživlja tako, da ima neko izbiro med formati slik. V vsakem primeru si izbere format, kjer je bolj poudarjena prostorska strategija;

Vmes je spremenil strategijo na iskanje neke celovitosti.

Odločitev o spremembi strategije: imel je občutek, da mu vedno nekaj zmanjka in da mora poskusiti nekaj novega. To je doživljal kot neko čustvo. To je bilo zelo bledo čustvo.

Celostni vtis: poskušal je videti vse naenkrat in gledal, če bo prišlo do spremembe. Meni, da so diskretne enote kodificirane v tej celoti, vendar je ne bi mogel rekonstruirati. Razliko lahko zazna le ob primerjavi;

57 S10-01-O-02

Reševanje naloge je bilo veliko bolj površno, kakor prej;

Zraven se je spraševal, ali se stvari sploh menjajo in koliko se spreminjajo; koliko se spremeni samo eden od štirih, ipd. To spraševanje je bilo verbalizirano. Ob teh mislih je še vedno doživljal dražljaje, vendar se je manj ukvarjal z njimi;

Pozornost: malo je bil naveličan in pozornost mu je začela prehajati. Naveličanost je doživljal dvodelno kot a) umik truda in b) tavanje pozornosti;

Trenutek odgovora: še vedno je informirano odgovarjal. Razlika je bila v tem, da je bil večji element ugibanja, ker se je manj trudi ohranjati reprezentacijo med pavzo.

58 S10-02-P-01

Spominski dražljaj: s premiki glave je skakal od objekta do objekta. Ob tem so bili občutki premikov večji kot so bili dejanski premiki glave → ti skoki so bili precej hitri. On jih je počel, vendar jih je spremljal nek občutek avtomatičnosti.

Občutek gibanja: kazal se mu je kot nek vestibularen občutek premika glave; → bilo je kakor če bi se vodil po cesti in ga njena oblika premika; doživljal je neke male telesne občutke premika, ki jih je čutil v glavi, obenem pa ga je malce simuliral z dejanskimi premiki glave;

Ob tem je ustvarjal zvok: »hm, hm, hm, hm,« občasno pa si je tudi štel ob dražljajih; → običajen notranji glas, njegov. Ta zvok je bil vedno isti. Niso bili zvezani z lokacijo, ampak je bilo vseeno tako, kot da bi se ustvarila neka štiristopenjska lestvica, ki je ostajala enaka ne glede na primer;

Govor številka: namesto zvoka si je z istim glasom kdaj pa kdaj govoril številke »ena, dva, tri, štiri.«

Pavza: še enkrat si je ponovil ta ritem, ob čemer je ponovil te skoke z očmi. Poskušal je ustvariti nekakšen odmev tako v zvočni kakor gibalni modalnosti. Ponovil je oboje, le da je bila jakost zmanjšana. Ob ponovitvijo očesnih skokov je prostorski občutek ostal enak kot prej;

Akustični element »hm« je bil nekako pridušen;

Testni dražljaj: v ozadju je imel ta *template* gibov in zvokov, sicer pa je pasivno spremljal dražljaj. Preprosto je opazoval, koliko se sklada in čim je prišlo do neskadja, je spustil reprezentacijo, ta notranji oblaček, ki se je prej ohranjal;

Prepustitev dražljaja: bilo je kot, da je bil nanj vezana neka sila, nek moment, ki je terjal nek prag akcije. On ga mora samo iniciirat, po čemer se ta reprezentacija zmanjša, ali izgine. Ni kot da bi presekal, ampak zbledi. To je doživljal kot razbremenitev, ki je mešanica telesnega in mentalnega občutka (napetost v glavi izgine v trenutku, okrog pritiska tipke);

Trenutek odgovora: doživel je mini nagraden občutek → to je isto kot zgoraj omenjena razbremenitev.

59 S10-02-P-02

Dražljaja sta bila enaka;

Med reševanjem je dokončeval misel, ki se mu je razvila tekom celotnega bloka in sicer, da je različen občutek med tem ali sta ista ali različna in potem je razmišljal, kaj je tista stvar, ki ju ločuje. To je zaobjelo dve tretjini njegovega doživljajskega polja;

Ta misel je bila nekakšen dialog, ki ga je imel sam s sabo; moral se je malce zadržat, da ni prevzel njegovega celotnega občutka. Tako je vzniknil občutek, da je uravnovešal reševanje naloge in soočanje s tem vprašanjem, kaj je razlika med enakimi in drugačnimi dražljaji;

Pozornost je bila lahkotna in avtomatična in brez truda je prehajal med tema doživljajskima tokovoma;

Istost: to je bila neka dopolnitev vzorca; ob istosti je doživljal nek prijazen občutek. Ob tem na površje pridejo neki prijetni osnovni občutki. Ob različnosti pa pride do nekega drugačnega občutka. Ti občutki so zelo globoki in težko dostopa do njih.

60 S10-02-P-03

Opazil je razliko v tem, kakšna je razdalja med objekti;

Osredotočal se je na to, kaj ustvarja to razliko v razdalji;

Osredotočal se je na prostor med pikami;

Prostorskost: povečana pozornost, oziroma zoženje pozornosti na prostor, kjer so se pike pojavljale;

Kadar je bil med pikami majhen prostor, se je osredotočal nanj, kadar pa je bil prostor velik, pa je ob njem doživljal akustično komponento, ki pa je bila nekako razpotegnjena (vendar tega ni doživljal ob vsakem primeru)

Pavza: onkraj njegovega doživljanja;

Testni dražljaj: onkraj njegovega doživljanja;

61 S10-02-P-04

Imel je občutek, da je bilo vse bolj ustavljeno (imel je tak *mindful* občutek) in ni si več ponavljal;

Vtis dražljaja, ki si ga mora zapomnit je postal veliko bolj avtomatičen, kar je izviralo iz pričakovanja, da ga bom ustavil;

Zadnji dražljaj je bil precej lahek;

Prepoznava istosti: imel je neko zaupanje v to, da je ta minimalni vtis, ki ga ima, dovolj, da bo lahko odgovoril. Ta vtis se mu je kazal kot nek občutek, ki je bil kakor da bi ga shranil v podzavest in da se je cel proces zgodil onkraj njegovega zavedanja. On je dal samo zeleno luč;

To, da je nekaj izven njegovega zavedanja je zaupanje v avtomatizem; enako je zaupanju, da obstaja svet za njegovim hrbtom, tudi če ne gleda vanj. To je zaupanje v zveznost, v to, da se svet ne spreminja kar tako. To zaupanje je zelo mentalno;

Meta-kognicija: na splošna ima malo občutka, da si je dobro zapomnil dražljaje. To je vezano na jakost tega vtisa. Sicer ta občutek prihaja iz drobovja, ampak je zelo blag občutek. Občutek dviganja iz drobovja navzgor.

62 S10-03-O-01

Povedal jih je v zaporedju v obliki pravokotnika. Uspešno je pogledal tri, četrtega pa ne; → ta gib je bil malce zavtomatiziran, ampak je še kar imel občutek, da je prisoten, kakor da bi vedno nihal med avtomatizmom, ki ga aktivno usmerja.

Pavza: ni imel česa zadrževati v glavi, bil je le nekakšen preskok, med katerim ni imel ničesar v glavi;

Izginotje dražljajev pred pavzo: na to je bila vezana neka anticipacija, onkraj tega pa je težko reči, kaj je doživljal;

Testni dražljaj:

Imel je precej dober vtis prvih treh, vendar ne četrtega: meta-kognicija: imel je samozavesten občutek; nekako si je ustvaril strategijo, da si zapomni samo tri; vedel je, da je vtis zelo boljši, če se osredotoča samo na tri. Četrtega je doživljal, kakor da je tam nekaj bilo in od njega je ostal nek *feeling,* ki se mogoče lahko vrne. Včasih je sproti pobral z očmi četrti dražljaj in takrat je vedel, da je nekaj tam, sicer pa tudi tega ni bilo;

Četrtega ni mogel rekonstruirati med pavzo, med testnim dražljajem pa je kdaj vedel, ali je enak ali drugačen;

Ustvarjanje strategije: ustvaril jo je skoraj takoj, po drugem ali tretjem primeru; ugotovil je, da se bolj splača, če si zapomni tri. Ta ugotovitev se mu je aktivno kazala. Ni zgolj spremenil svojega vedenja, ampak je bolj gotovo pristopil k nalogi, če si je zapomnil le tri. Pri tej strategiji je imel občutek, kakor da je vešč te naloge;

Samozavest: ni občutka zmedenosti ali lovljenja. Nima občutka, da ga nekaj pregana, ali da bi moral posvečati več pozornosti temu, da zadržuje to podobo v glavi. S tem je sicer zvezan nek telesni občutek, ampak težko reče, kaj je. Je zgolj neka pozornost. Malo je privzdignjen v telesni drži;

Preskok med pavzo: ni bil zares preskok, ampak je doživljal nek tok, ki je bil kakor, ko vržeš žogo po stopnicah in veš, da so neki nivoji, kjer se za hip ustavi, ampak veš, da ima svoj moment;

Pojavitev testnega dražljaja je doživljal zvezno. Zveznost je bila vizualna, pri čemer ni bil to zares občutek časa, ampak je bila neka zaporednost;

Prepoznava istosti: onkraj njegovega doživljanja;

63 S10-03-O-02

Primer je doživljal kakor da bi ga lahko premaknil;

Vsak objekt posebej je pogledal in je z očmi naredil gib v smeri posamezne orientacije;

Bilo je kakor da bi jih hotel s pogledom prestaviti v smer, kamor so kazali. To strategijo je ubral med testnim, med spominskim pa je ponovno pogleda samo tri, četrtega pa je pustil;

Zaradi tega giba je bil spomin bolj občuten. S premikom oči je naredil dejanski občutek, da je tam nekaj bilo in to nekaj je bledelo (prim. ko se dotakne roke, še kar čuti, kje se jo je dotaknil, ko umakne prst);

Na premike oči je *outsourcal* to, da si je nekaj zapomnil;

Od vizualnega je tam ostal nek vtis, ki je počasi bledel. To je bilo kakor paslika. Doživljal jo je na ekranu, pripisoval pa ji je sekundaren pomen;

Gib je čutil na očeh; imel je občutek, da je bilo nekaj tam;

Pavza: (mogoče) še enkrat je z očmi naredil te gibe in potem je vlagal nekaj truda v to, da jih zadrži. To je on počel. Priprl je veke. Aktivno se zaveda, da je to rekonstrukcija med intervjujem;

Pozornost: je bila nekaj srednjega med lahkotnim in trudnim;

Vedenje o istosti: pogledal je posamezne točke in primerjal je skladnost vtisa v očeh in tega, kar je bilo na ekranu. Ta skladnost je preprosto bila.

Trenutek odgovora: onkraj njegovega zavedanja. Meni pa, da je bilo precej avtomatsko. Da se mu je dogajalo.

Meta-kognicija: v splošnem ima ta občutek. Kaže se mu kot intenziteta vtisa. Pri pasliki gre za to, kako močna je, kako bleda je. Pri tem občutku premika pa je isto. Poleg tega pa je še pozornostni vidik in emocionalna komponenta. Emocionalna komponenta je v veliki meri vezana na nagrajajoč občutek.

64 S10-03-O-03

Ponovno je trikrat s pogledom šel čez dražljaje. Še vedno je delal ta premik z očmi, s tem da je imel manjši fokus na tem; kombiniral je strategijo s pogledi in izločanjem zadnjega dražljaja;

Ugotovil je, da se ta premik z očmi bolje vtisne, če gre na ven, pri čemer ga tudi hitreje izvede;

Če je šlo na noter ponavadi ni mogel dovolj hitro tega naredit;

Pavza: v zadnjem primeru ga je nekaj zmedlo in ni vedel, ali si je prav zapomnil ali ne;

Testni dražljaj: malo mu je bilo odveč. Čutil je željo po tem, da bi bilo nekaj drugače. Igral se je z mislijo, da bi spremenil strategijo, ali morda poskušal potrpeti, da bo konec vsega;

Vznik odločitve, da bo kombiniral strategije: tekom prejšnjega intervjuja si je rekel, da bo poskusil nekaj drugega in sicer da se bo fokusiral na te premike z očmi; v ozadju je imel nek plan, da bo poskusil novo strategijo. Ni preprosto spremenil vedenja. Moral je podati zeleno luč.

Oči: doživljal jih je podobno kot prej, le da z manjšim fokusom;

Fokus: več je razmišljal o tem, kam bo pogledal, ne pa toliko o tem, kaj bo naredil. to je bilo neko ozadnjo premlevanje. Tega premlevanja ne bi mogel ubesediti;

Iz središča ven se bolje vtisne: središče je doživljal drugače kot preostanek vidnega polja. Nikdar ga ni pogledal, ampak je bil vedno tam. Ni imel kakega občutka teže.

Meta-kognicija: več je bilo emocionalne komponente in manj zavedanja, kako dobro se mu je dražljaj vtisnil. Bilo je bolj mentalno.

Testni dražljaj: vse skupaj je poteklo zelo avtomatično. Aktivno se je zavedal le, če je kdaj narobe pritisnil tipko, ob čemer je malo podvomil v to, kar počne. Bilo je, kakor da bi prsti sami od sebe poskušali neke stvari in pošiljali signale v glavo. Imel je bolj občutek, da se mu to dogaja; zavedal se je občutka položaja prstov in malce tudi oblike, kjer sta se prsta dotikala tipk (minimalna oblika);

65 S10-03-C-01

Precej slabo se mu je vtisnilo;

Ne ve zares, kaj je hotel narediti med samim primerom. Ponovno je kombiniral različne strategije: poskušal je zajeti vse naenkrat, ali pa je skakal od enega do drugega objekta ter si jih verbaliziral;

Pri zadnjem primeru je naredil mešanico vseh strategij in na koncu je med testnim dražljajem imel zelo osnoven občutek tega, ali je bil dražljaj isti ali ne;

Spominski dražljaj: barv ni zares doživljal. Tekom celotnega primera jih je preprosto videl kot iste ali ne/iste;

Meta-kognicija: vedel je, da je imel slab vtis teh barv. Ni imel ničesar, česar bi se lahko oprijel. Po spominskem dražljaju ni od njih ostal nikakršen odmev. Ni jih videl ali slišal kake zvoke in tudi če bi hotel jih ne bi mogel rekonstruirati. Videl je precej jasno sliko teh barv, vendar jih ni mogel poimenovati, ko so enkrat izginile;

Jasnost med spominskim dražljajem: med posameznimi objekti so jasne prostorske relacije. Zaznava jih jasno v prostoru. Intelektualno ve, da so štirje krogci, vendar pa je zaznaval neko celoto.

Preskakovanje med strategijami: imel je cilj, ki je bil, da mora najti neko strategijo, ki bo efektivna. Specifične strategije pa so potem prihajale naključno same od sebe in jih ni on zares oblikoval. Imel je občutek, kakor da ima pripravljeno naslednjo.

Testni dražljaj: osnovni občutek istosti ali neistosti → (mogoče) ta vtis se je malo pomešal s testnim dražljajem, kar je vodilo do neke spremembe, v čigar primeru je to razumel kot neisto (torej je bila ta primerjava aktivna); tega vedenje, od kod je prišla ta razlika ne more ubesediti. To je nekaj, kar je preprosto vedel, da je res; sam občutek primerjave je bil vsebovan v dražljaju; ta primerjava je torej aktivna;

66 S10-03-C-02

Imel je nek občutek za barvo, ki si ga je zapomnil tako, da je kombiniral dvojice, ki so pasale skupaj. Iz tega grupiranja po ustreznih barvah je celotna slika izgledala bolj ali manj grdo. Če je bili testni dražljaj lepši ali grši, je vedel, da je drugačen;

Grdost, denimo, se je kazala kot občutek na očeh; dražljaju je pripisal zelo majhno valenco. To je bil telesen občutek, ki pa je bil tako majhen in hipen, da ga je težko kamorkoli umestil;

Velikokrat je kombinacije z zeleno doživljal kot grde: ko je opazil, da obstaja razlika med lepim in grdim (element geste refleksije), ga je začelo zanimati, kaj dela slike lepe ali grde in opazil je, da je faktor, ki nekaj naredi grdo pogosto bila zelena;

Zelena ima nek gnusen pridih; to je obenem mentalno, telesno in vezano na nek spomin; zgodi se hkrati z vizualno podobo. To se sicer dogaja, vendar ga je opazil šele, ko je enkrat začel posvečati pozornost temu;

Istočasno s tem je opazil, da najprej izginejo barve in potem oblika in sicer tako, da se skrči vase; to krčenje je videl konkretno na ekranu;

Testni dražljaj: pri ugotovitvi istosti ni bilo nobenega vmesnega koraka primerjava, kakor v prejšnjem primeru. Bilo je samo ali ustreza ali ne;

Testni dražljaj: pozoren je na spremembo v tem estetskem občutku, po čemer ve, ali je prišlo do spremembe v dražljaju;

Pritisk na gumb: je precej avtomatičen in ne razmišlja o tem, kaj mora storiti; pripravljeno ima, kaj se bo zgodilo in če ne pride do spremembe pusti, da teče in da gre po svoje;

Prisotnega je več njega, kadar pride do spremembe; bolj aktivno pritisne, kot kadar je ni;

67 S10-03-C-03

Na začetku, ko je še malo v glavi imel prejšnji pogovor občutka za estetiko, je poskušal vse skupaj početi zelo racionalno. Stopnjo estetike dražljaja je ocenil na lestvici od 1 do 10 z namenom, da ugotovi, kakšna je razlika;

Hitro je ugotovil, da je to nesmiselna strategija;

Iz tega pogovora je izvirala aktivacija racionalnega dela njega, ki je hotel vse skupaj analizirat, zaradi česar se je odmaknil od samega reševanja naloge;

Ocenjevanje: kar je govoril prej, je bilo malo vezano na nek občutek, tukaj pa je dražljaje racionalno analiziral in lahko jim je pripisal neko številsko vrednost. Te vrednosti niso bile jasno artikulirane, ampak bi jih lahko ubesedil številsko;

Racionalna modalnost: (lahko jo opiše le karikirano): bilo je, kakor da bi zraven prišlo nekaj, kar je preveč pripravljeno;

Vedel je, kakšen je estetski občutek še preden ga je racionalno zanalizirat; nesmiselnost tega pa se mu ni pokazala takoj, ampak šele kasneje;

Spoznanje o nesmiselnosti strategije: ni bil prepričan v svoje odgovore, kakor pri drugih strategijah; prepričanost se mu je sicer kazala kot primaren občutek tega, kako dobro si zapomni stvar.

68 S10-04-P-01

Reševanje primera je bilo zelo lahkotno;

Objekti sami so doneli, spremljal jih je nek glas, nek zvok. Ko ga je opazil, se je vprašal, zakaj ga sliši, ob čemer se je malce zmedel in kasneje je bila njegova pozornost malce okrnjena;

V glavi je ob pojavnosti dražljaja zaslišal zvok; ta zvok je bil diskreten in zvezan s posameznim dražljajem;

Istočasno je s pogledom naredil premik čez štiri objekte;

Zvok je bil relativno glasen in je za sabo pustil odmev; ko mu je začel posvečati pozornost pa je njegova jakost upadla in za njim ni bilo več odmeva;

S pogledom je malce zanihal čez vse objekte;

Spominski dražljaj: s pogledom je sledil objektom, ob tem je zadonel nek zvok. To sta bili dve vzporedni stvari;

Pavza: ni bilo ničesar. Zdelo se mu je, da je bil to nek prazen prostor. Primera ni doživljal kot dveh dražljajev, ločenih s pavzo, ampak kot dve polovici istega dražljaja;

Testni dražljaj: premik + zvok;

Zvok je bil kot nek tlesk, ali pa momljal. Spremljal ga je nek premik jezika. To je bilo z nekim notranjim glasom. Bilo je avtomatično, ampak v njegovem glasu.

69 S10-04-P-02

Spominski dražljaj: pri zadnjem primeru je bil malce zmeden. Ne ve, točno zakaj.

Spraševal se je, kako primerjati ne le okvirno lokacijo, ampak bolj prefinjene točke v prostoru. Zdi se mu, da so premiki oči nekoliko grobi;

Kako ve, da se lokacija ne vtisne točno? V enem prejšnjih primerov je opazil, da je opravil povsem enake premike, točna lokacija pa ni ustrezala;

Akustična komponenta: je bila prisotna, vendar je bila zelo drobna;

Objekti sami so bledeli: za njimi je ostal občutek, da je tam nekaj bilo; kakor da je za njimi ostal zelo, zelo bled odmev; to je opazil predvsem med testnim dražljajem;

Narisal je sliko o tem, kako je bilo to videti:

Tega odmeva ni videl ves čas, ampak je vzniknil med testnim. In ima občutek, da se je to dogajalo;

Pavza: med pavzo je ponovil gib;

Rekonstrukcija primera: SD: vidi objekte in jim sledi s pogledom + manjša akustična komponenta; P: ponovi gib, brez akustične komponente; TD: sledi s pogledom, obenem pa vidi vizualne odmeve teh odmevov;

70 S10-04-P-03

Hotel se je osredotočati na to, da bi videl odmeve, ampak so s tem, ko je bil pozoren nanje, odmevi izginili; tega ni več našel v svojem doživljanju;

Reševanje naloge: premiki oči + ostrejši fokus, brez nekih avditornih komponent in z manj poudarka na gibu;

V ospredju njegove pozornosti je, ali bo ujel te odmeve. To se mu je kazalo kot usmerjanje pozornosti na točko, kjer so se prikazovali objekte, ob čemer je čutil željo, da bi tam nekaj ostalo;

Želja: malce je bila vezana na pričakovanje, bila je neko verjetje, da tam nekaj bo; v to je celo vlagal nekaj truda, kakor da bi poskušal v video posnetku opaziti za en *frame* dolgo subliminalno sliko;

Dražljaj je tako nekako postal odmaknjen od njegovega doživljanju, kljub temu da mu je posvečal ogromno pozornost;

Nalogo je sicer še kar reševal, pri čemer je bila njegova strategija reševanja v veliki meri vezana na premike z očmi;

Trenutek odgovora: preveril je, če je naredil iste poglede z očmi, potem pa je precej avtomatično odgovoril. Ta vtis je bil kvalitativno drugačen od estetskega občutka iz S10-03.

71 S14-01-O-01

Spominski dražljaj: s pogledom ga preišče, začenši z dražljajem desno zgoraj, po čemer naredi krožni gib z očmi in poskuša 4x zabeležiti splošno smer;

Premik z očmi zahteva veliko mero pozornosti, predvsem ker ji naloga ni zanimiva in čuti težnjo, da bi prenehala;

Pozornost: prisoten je mali *drift* oči, ki ne gre v smer, kamor bi moral iti – mora ga popraviti, kar je močno telesno doživljanje;

Naloga je dolgočasna: tiho zavedanje, da bo gledala veliko med sabo nerazločljivih vzorcev in ve, da bo zadeva trajala dlje časa in mora varčevati z energijo;

Tega se zaveda le tiho: to ni glas, ampak občutek, ki je podoben utrujenosti po nekem akademskem delu in da mora varčevati z energijo, obenem pa je zahteva naloge takšna, da ji mora posvečati pozornost;

Med nalogo ni imela časa za neko verbalizirano spremljanje, od tega je imela le občutek: to ni bilo telesno; spomni se zavedanja, da bo vse okej, tudi če se zmoti pri nalogi;

Beleženje usmeritve: oči za nekaj trenutkov sledijo črti in zabeležijo smer;

Ko preverja testni dražljaj, preverja, če je *feeling* ob tem, kako oči zarišejoo krog enak kot med prvim;

72 S14-01-O-02

Vzorec je uničen zaradi iskanja tipke za pavzo.

73 S14-01-O-03

Ko vidi spominski dražljaj, če med pregledom vmes pomežikne, si ustvari pasliko, nek *afterimage,* in tako se cel prvi dražljaj še malo razvleče v pavzo;

Paslika: je bela na zelenem, vidi samo črte brez krogcev, če sta si diagonalno in kažeta drug proti drugemu, se združit a v eno večjo črto; vidijo jo zadaj za ekranom; začne se kot manjša, potem pa zapre oči in ko jih spet odpre, se poveča, kakor da padeš skoznjo; to je vizualna izkušnja, ampak postavljena v prostor;

Pavza: zapre oči, v tistem času, ko spet odpre oči, vzdržuje pasliko, ki ni več to. Ko ima zaprte oči je bela slika na zelenem, potem pa je bela na malo manj belem; druga paslika je bolj bleda; od nje ostane nek *gist*, ki ni več vizualen, ampak gre bolj za občutek, da mora biti, kakor je bilo (torej ima neko vizualno komponento);

Vzdrževanje paslike: stresno doživljanje. Četrti objekt je zelo hitro bledel in če se ni trudila, ga je pozabila; videla je utripanje na tistem mestu, ta katerega ni prepričana, kaj je; kakor da se menjajo možnosti in potem se pojavi testni dražljaj; poskuša se spomniti vseh možnosti in če nekaj klikne, če nekaj ustreza, se odloči skladno s tem;

74 S14-01-O-04

Lažje si zapomni dražljaje, ki so diagonalno usmerjeni in se povežejo v enega; poveže jih v X ali pol-X, medtem ko so ravne usmeritve težje za zapomnit;

Testni dražljaj: v veliki meri je izven njenega doživljanja: odloči se na podlagi tega, kako podoben je ta dražljaj spominskemu;

O dražljajih, ki si jih zelo dobro zapomni, sploh ne razmišlja – ali se skladajo, ali pač ne;

Dobro zapomni: dražljaj ostane tudi po tem, ko spominski dražljaj izgine in to he ta paslika, o kateri je bilo govora višje;

Ugotavljala je, kakšna je verjetnost, da si je pravilno zapomnila: desno zgoraj si vedno zelo dobro zapomni, za leva dva je pa ta verjetnost odvisna od primera do primera: zmanjka ji časa, da bi s pogledala zaobjela vse štiri;

Ustvarila je vzorec in sicer tako, da ene stvari skupaj zgledajo lepše kot druge;

Sestavi novo obliko, da si mora zapomniti manj objektov;

To da nekatere lepše pašejo skupaj: paslike se same od sebe povežejo skupaj;

Nima dovolj časa: ima občutek, da čas mineva na račun tega, da je spominski dražljaj krajši;

Trenutek odgovora: malo pomisli, da je leva tipka za enake, desna pa za drugačne; tega ne verbalizira, ampak konkretno premakne svoj prst;

Prst pa dejansko občuti šele ko odgovori;

75 S14-01-O-05

Spominski dražljaj: ustvaril se je zelo lep vzorec; ugotovila je, da ne bo tako težek, zaradi česar ji je padla pozornost;

Oblika je bila tako logična, da jo je kljub manjši pozornosti prepoznala;

Lep vzorec: tista dva, ki sta zgoraj ali spodaj, se nista razlomila; šla sta v isto smer;

Te vzorce vidi kot diskretne enote, ali pa kot en lik, vendar nikoli kot štiri objekte; vedno zmanjša njihovo število;

Skupaj z upadom pozornosti se je razveselila, ker se ji je vzorec zdel lahek. Kot da so njene oči bile malo bolj lene, kot da je pozornost postala malo bolj lahkotna;

Pomislila je, da se je premalo potrudila. Ta misel je vzniknila med pavzo. Skupaj z njo je čutila manjšo paniko, in malce obžalovanja. Ta misel je bila čustvo; ni bila verbalitirana, ampak je zaslišala nek medmet, slišala ga je za glavo na levo:

To je spremljalo spoznanje, da je vse skupaj *irreversible*, to je bila neka želja, da bi ponovno videla spominski dražljaj;

Domnevam, da se vzorci povezujejo skladno z načeli Gestalt psihologije.

76 S14-01-C-01

Naloga ji je bila veliko lažja;

Spet je grupirala barve v pare glede na nek *tag* (npr. osnovne barve), ali pa v kaki relaciji sta;

Grupiranje: verbalizira si, vendar ne v obliki celega stavka. To je samo nek vzklik, nekakšen medmet, ima ton. Tista dva, ki tvorita enoto, imata podoben ton. Ta je pri drugih dveh drugačen;

Ta ton je do neke mere bezan na pogled;

Pavza: vzdržuje ta spomin → ostajajo neke paslike, ki so kvalitativno drugačne od prej; rumena ostane kot neko sonce, rdeča kot jabolko; ba te asociacije ne misli, ampak se osredotoča na tiste, za katere nima asociacij;

77 S14-01-C-02

Vse je bilo tako različno, da ni znala ničesar zares povedati.

78 S14-01-C-03

Spominski dražljaj: ne spomni se zares, kako ga je doživljala;

Eno barvo vedno doživlja, kot da je malo manj barva; je nek *outlier*, ki si ga malo bolje zapomni. Ve, kje je tisti, ki je bilo drugačno;

Preostali trije so ustvarili nek set barv;

Ta set si dejansko zapomni: kako si jih zapomni, je odvisno od kombinacije; povežejo se, denimo, v barve neke zastave:

Zapomni si posamezne barve: zapomni si, a,i tako, da jih poveže v en vzorec, na primer zastavo, ali pa jih grupira po lastnostih;

Pavza: kot da bi si hotela zamisliti te barve, kot da bi jih hotela pričarati;

Poskušala je obuditi, kar je videla in to je bilo zelo zahtevno, kar je doživljala kot da se mršči (telesno doživljanje na površju čela);

79 S14-01-C-04

Spominski dražljaj: videla je kombinacijo rdeče-belo-zeleno + outlier; za vsako si je morala zapomniti le položaj, najprej si zapomni *outlier* in potem, kaj je okoli njega;

Outlierja doživlja kot da ga na nek način ni;

med pavzo je samo vzdrževala spodnje dva;

čez čas je prišel nek dvom glede svojega spominja, kar se ji je kazalo kot stiskanje v želodcu;

testni dražljaj: videla je tri, ki so se ujemali iz prve, ob čemer je občutila olajšanje – nenadoma je lahko normalno vdihnila;

Trenutek odgovora: spet je preverila, kateri gumb je kateri;

Ujemanje je spremljal nek medmet; ta zvok je njen;

Bolj prijetno ji je, če ve, kakšen je odgovor;

80 S14-01-C-05

Dražljaja se nista ujemala in je izgubila vse doživljanje.

81 S14-01-C-06

Pavza: osredotočila se je na *outlierja,* vzdrževala je zaporedje barv prek verbalizacije »rumena, levo zgoraj …« in vidne predstave. Poskušala si je zapomniti vsaj eno izmed teh verbalizacij;

Prostor, v katerem so barve ni nekaj fiksnega: ni vedela, kakšne so dejanske razdalje; prostor je razširila in povečala (med pavzo);

Ta delovni prostor je pod njo, pod očmi;

Med pavzo nadaljuje z verbaliziacjami;

82 S14-02-P-01

Zapomnila si je, kakšno pot mora orisati slika;

Pavza: vzdrževala je to sliko poti;

Testni dražljaj: slika ni bila enaka, ob čemer je bila kar malo užaljena;

Pot: pika izgine in se pojavi nekje druge, v tem trenutku pa se izriše neka črta, jer so bistvene orientacije; malo je bilo vezano na gibanje oči; oči gredo naprej in nazaj; pri črti je v resnici šlo bolj za nek prostorski občutek;

Občutek ima, da ona ustvarja to prostorsko črto;

Pavza: vzdrževala je ta prostorski *feeling,* spominjala se je, kako so v vrstnem redu potekale orientacije teh štirih črt;

Poskušala se je spomniti nanje: poskušala si je zapomniti razdaljo same pike od fiksacijskega križa;

Prostorski *feeling* je bil v resnici relacija od pike do fiksacijskega križa;

Skrbelo jo je, da si sta dva dražljaja podobna: do spominskega dražljaja je začela čutiti nekakšno racionalno navezanost, češ da preprosto mora obstajati v nadaljevanju; to je hipen, mentalen občutek;

Pikam sledi z očmi, riše pot, močno zvezano z samimi gibi, zraven pa se je navezala na vzorec; ta navezanost se je začela z drugim, ko se je začela vzpostavljati oblika;

Vzorec doživlja kot eno samo piko;

Pri poti je pomembna orientacija: ni popolnoma simetrično samo vase, ampak je nek trapezoid, kot da bi daljše črte gledala od zgoraj;

Če bi spremljala pike na daljši razdalji, je bulo, kakor da se je pika malce dvignila;

Pavza: Še enkrat je izrisala pot: našla je lokacijo, pomežiknila, povezala: ko tretje pike ni vedela, se je poskušala postaviti nazaj na to, kakšna je bila cela slika med spominskim dražljajem;

Pot tedaj ni bila zares vidna, bolj je bila neka slutnja, ki povezuje prostorski *feeling* pik;

Pavza: med pavzo ni čutila nobenega afekta;

Testni dražljaj: čutila je užaljenost, ker ni bil isti. To je bila neka ranljivost, otroška užaljenost, ki se je kazala kot mentalni občutek → ta občutek se kaže kot nagnjenost k minimalni dejavnosti, k lenobi, kot da zdaj noče zapravljati energije za nekatere stvari.

Ob tem pa čuti, da jo nekdo obsoja, kar čuti telesno, v grlu;

Tega si ni konkretno govorila, bolj je bilo nekakšno tiho razočaranje;

Primerjava: dražljaj je bil nekaj popolnoma drugačnega in opustila je vse skupaj: ko se je pojavila druga pika je v mislih zverbalizirala »to ni to!« → s tem glasom se poistoveti in bil je voljan;

Torej sta bila pri prepoznavi dva koraka: dražljaj se ji je kazal kot različen, po tem pa je bila užaljena;

83 S15-02-P-02

Spominski dražljaj: z očmi je sledila pika in med njimi ustvarjala prostorsko črto;;

Pavza: ohranjala je splošno pozicijo prvega in drugega objekta; to je bilo neko polje, kjer se je nahajal oblak → neuniformna siva, ki jo je kvazi-vizualno projicirala, oblak pa je orisovala s tem, da je risala malo spiralo;

Spominski dražljaj: tokrat ni bilo neke čustvene komponente, ampak je obliko posvojila za svojo, kar je hipno doživljanje v trenutku, ko dobi svojo obliko;

Pavza: ustvari si enko idejo tega: to je vezano na to, da oblika vzame za svojo; s tem je zvezana želja, da testni dražljaj ustreza spominskemu: to se je kazalo kot nestrpnost, pričakovanje, da se bo vsaj prva pika pojavila na praven mestu, to pričakovanje je bilo malo fizično, kot neko motrenje v čelu (pričakovanje vznikne med pavzo);

Testni dražljaj: ni bil enak in za trenutek je podvomila vase, češ da ne ve;

Strateško sklepanje: ker je podvomila v to, da si je zapomnila prvo piko, je začela razmišljati, kako bo iz poti ugotovila, ali je vzorec enak; tega ni izrecno pomislila. Tokrat ni bila užaljena, ker je bil testni dražljaj vseeno bolj podoben spominskemu;

84 S14-03-O-01

Poskušala je ustvariti nek lik, ki bi zavzemal te dražljaje;

Pri testnem so se vsi lepo poklopili, razen četrtega, ki si ga ni zapomnila;

Iskala je nek potek, kjer bi se vse skupaj lepo zaključile; da bi vsaj dva elementa lika bila povezana; povezala jih je s črtami → v smeri, v katero kažejo, je ustvarila eno neskončno dolgo črto in bistveno je bilo to, da ima neko pomembnost;

Črte: debele so približno 2-3 mm, vledeče, zeleno-bele; črte vidi malenkost za ekranom in ima občutek, da jih ustvarja (če želi, da so tam);

Med temi črtami ustvari lik: med črto je najprej nek prostor beline, lih sam pa je črnosiv; ta belina ni belina ekrana;

Lik vidi malenkost bližje kot črte; še vedno je za ekranom, samo da je malenkost izbočen;

Pavza: med pavzo se poskuša osredotočati, da ne misli na druge stvari, da je pripravljena na to, da se bo nekaj pokazalo. To je doživljala kot napetost oči. Ta tenzija je zanjo v veliki meri pozornost;

Med pavzo lika ne vidi več;

Med pavzo ima neko stopnjo prepričanja, da bo uspela rešiti nalogo → to je prepričanost, da se bosta pozornosti primerljivi, da ne pričakuje sprememb;

Med pavzo so ji prihajale neke misli: kar se ji je kazalo kot občutek, ki ji hoče zmanjšati pozornost → to je telesni občutek v očeh, mišice v očeh niso pripravljene na izvajanje sakad, ampak bolj tavajo. To se ji dogaja;

Testni dražljaj: tisti trije objekti, ki jih je uspela zapomniti so ustrezali, za zadnjega pa ni bila prepričana; ta element jo je vodil v paniko;

Četrti dražljaj je obnovila iz ikoničnega spomina; zdelo se ji je, da je identična slika;

Imela je neko stopnjo verjetja v enakost, vendar to ni bilo 100%;

Obnavljala si je pasliko: zaprla je oči in potem kot da bi naredil nek *scan,* ki je dejansko črta; oči je premikala in ustvarjala ta *scan:* kvalitativno je bil podoben zeleno-vijolični črti od prej;

Četrti objekt je bil v relaciji do lika: ko je poskušala priklicati sliko od prej, ni bilo možno priklicati samo četrtega objekta, ampak ta doživljal sam zase ni obstajal; obstajal je samo skupaj z ostalimi in ne bi ga mogla izluščiti iz preostale množice;

Panika: kot da bi jo nekaj stisnilo v sapniku + malo ožanje vidnega polja;

Testni dražljaj: pogledala je, če se je lik, ki ga je priklicala ujemal s tem, kar je videla;

Prekrivanje: ob tem je čutila neko čustveno olajšanje. Ob prekrivanju je prišlo do nekega zavedanja, da se ji ne več potrebno ukvarjati s tem in da lahko malo popusti, da ima čas vdihniti; s tem je povezano izkustvo olajšanja;

Ob koncu zahteva, da rešuje nalogo popusti: to je avtomatsko: oči še kar naprej preiskujejo sliki: ona je v nadzoru nad svojimi očmi do te mere, da je njena volja, da rešuje nalogo. Sami gibi pa niso pod njenim nadzorom;

Stres: misli, da ne bo mogla vzdrževati tega in da ji je malo odveč reševati to nalogo + občutek nesposobnosti ob reševanju Prologa [ostanek izpitnega obdobja];

85 S14-03-O-02

Najprej je pogledala samo dva: zares je videla samo dva: druga dva sta bila bolj *gist*; za leva dva ve, kako sta bila obrnjena, za desna dva pa ostane bolj *gist* teh črt;

Pavza: vzdrževala je splošni *feeling* levih dveh dražljajev;

Poskušala si je zapomnit, kako bi te črte potekale;

Če je rešila prav, je bil v testnem dražljaju *gist* drugačen; ni ustrezal temu, kar je ona imela v *feelingu;* to velja le za en objekt;

Imela je občutek, kje bi se nahajala, če bi bila v tisti točki; kam bi jo vodila ta črta; vedela je tudi, kam bi jo vodil spominski dražljaj. Potem pa je primerjala, če bi hodit v tej ravnini, ali bi hodila v isto smer;

Dva elementa, ta katere je vedela, kako sta obrnjena: obrnjena sta bila v tako smer, da se ji je zdelo, da zaključujeta nekaj, kot da sta skupaj rob vogala, prek tega občutka je dostopala do znanja o njuni obliki; Vogal je bil popolnoma vizualna izkušnja, kakor da se tam vse, kar je relevantno, konča; kot da onkraj tega sploh ne gre več za doživljanje, ampak je meja vsega, kar je znano;

Ni se zavedala ekrana onkraj te meje, onkraj je videla le črno-vijolično;

To je ista črno-vijolična, ki jo vidi, ko se zazre v svoj notranji mentalni prostor;

Druga dva pa je videla kot *gist* → vsak od njiju ima svoj *gist:* kot da če bi se znašla v desnem zgornjem, vi videla kam kaže levi spodnji in kot če bi se znašla v desnem spodnjem in gledala proti zgornjemu, vi vedela kam kaže zgornji; eden je bil zelo zvezan z drugim; informacija o enem se je nahajala znotraj tega, da se je postavila v drugega;

Med pavzo je ohranjala splošni občutek teh dveh *gistov,* tega feelinga ne bi mogla razpreti in ven dobiti posameznih usmeritev, lahko je dobila le njihovo presečišče. Da vi vedela, da sta te dve črti, bi morali biti na dveh točkah hkrati;

Pavza: zavedala se je, kje bi to presečišče bilo, če bi stala v obeh hkrati in gledala proti drugemu; to zavedanje se je kazalo, da se je zdelo, da stoji v desne spodnjem in dojema razdaljo, kjer se nahaja presečišče teh dveh premic; bilo je kot da bi fizično stala v tej točki;

Vedenje o tem presečišču je bilo prostorsko, kar je doživljala prostorsko, kot da je tam en ogromen objekt;

Ta objekt je bil vizualen: to je bil ogromen valj: vidi ga na robu pogleda in ni preverjala če ima vrh ali če je zaključen;

To je konkretno videla: črno-vijoličen valj v belo-vijoličnem prostoru; levo od tega valjam je belo-vijolična, desno od njega pa ni nič;

To se dogaja v dejanskem prostoru: valj je širok, da bi lahko osem udeleženk sklenilo roke okrog njega;

Prostor: vidi ga povsod, kjer ni valja in nahaja se tudi onkraj njene pozornosti; to je njen mentalni prostor, ki se začne tam, kjer se meja njene pozornosti začne prelivati v resnični svet;

V oziru do valja, je levi vogal malenkost bližje njej kot ta stolp; vzpostavlja neko relacijo in vse to vidi v 3D prostoru;

Vse to ona ustvarja in to od nje terja veliko mentalne energije; ko je ta prostor enkrat vzpostavljen, pa ni bilo več tako naporno;

Ob prehodu v testni dražljaj vztraja, da tam obstaja levi vogal in da tudi valj na nek način je; zapomniti si poskuša, kako daleč se čuti ta valj, poleg tega pa pazi na to, da je testni dražljaj ne pretenta, da se nahaja na drugi relaciji:

Da je ohranjala to relacijo, je bila pozorna na to, kolikokrat bi se morala stegniti od enega do drugega, da bi prišla do tam; to je bilo vizualno, ampak za ohranjanje tega je izvajala mentalne premike; kolikor energije bi terjalo do tja (motorično);

Onkraj tega belo-vijoličnega prostora ni videla računalnika; to je popolnoma prevzelo njeno vizualno zavest;

Testni dražljaj: ugotovitev: ob strani je pustila relacijo spominskega dražljaja, vendar le za malo. Čim prej je poskušala oceniti, koliko bi trajalo, da pride do testnega in če gre za približno enako energetsko porabni premik;

Testni dražljaj: doživljala je oba dražljaja hkrati: spominski dražljaj je imel nek *afterglow*, kot da je zavedanje o zahtevnosti premika na začetku bilo močno, s časom pa preden ta občutek zbledi do te mere, da mu ne bi več mogla zaupati, pa se pojavi testni dražljaj in ustvari novo relacijo; hitro bledi in terja napor, da ga ohranja, na to pa dodajo razliko med njima → zdela sta se ji enaka; imela sta isto pozicijo v prostoru;

86 S14-03-O-03

Vsakič ko dva objekta kažeta na 45° običajno ustvari X; s svojim glasom si reče »iks« in tega ne reče jezik, ampak samo grlo. Ve da je X, ker samo zanj grlo naredi nekaj takega (prim. s /h/), kar naredijo usta pa sploh nima veze. Reče si, da bosta desna zdaj X;

Za leva dva si je naknadno rekla, da bosta kot T, »t-desno«. Ni si govorila vsega;

Znaki so se kar naenkrat zlili skupaj; to povezavo je naredila ona; dokler je bil celi dražljaj tam, pa je drugim prilepila še eno ime;

Pavza: poskušala si je spomniti, kakšen je bil X na desni: ohranjala ne nek prostorski *feeling:* če repki kažejo na ven, se običajno prostor razširi, če pa kažejo na noter, se je občutek prostora dosti manjši; zdelo se ji je, da je celoten dražljaj manjši in med pavzo je ohranjala to razdaljo;

Testni dražljaj: iskala je, če je testni dražljaj enako velik: bolj pričakuje, da bodo kake razlike v izbočenosti; te razlike ni videla:

Enakost: tako kot prej jo je spremljala neka pomirjenost; je razlika, ki je ne bi mogli pripisati bledenju;

87 S14-04-P-01

Poskušala je orisati vzorec črt;

Ni dejansko risala vseh povezav, ampak samo dve diagonali;

Nariše jih že med spominskim dražljajem, potem pa med pavzo samo sledi temu vzorcu;

Testni dražljaj: vse, kar si je morala zapomniti, je, če se je začelo na eni od končnih diagonal;

Diagonale: ozadje je bilo belo, točka črna, na njenem mestu pa ostane črnina, kasneje pa se tam pojavi bela črta, kjer pa prehaja v črno, njeni robovi so belo-zeleni; vidijo jo malo onkraj računalnika;

Črto vidi, ko povezuje pike, ni ves čas tam, ampak samo ko sledi objektom;

Meni, da jo določa dolžina očesnega premika;

Pavza: sledi točkam, pri čemer so jo vodila diagonale;

Pozorna je bila tudi na relacijo do fiksacijskega križa, ker se ji zdi, da ji pomaga pri sestavljanju;

Vse bledi skladno s pozornostjo, ki jo posveča drugam;

Imela je občutek, da si je uspešno zapomnila ta občutek → za nobenega od korakov ni potrebovala premišljevanja ali si ustvarjala alternativ. Med pavzo je točno vedela, kam bi moral pogledat in kar se je na koncu premika oči izkazalo, da je mislila, da si je zapomnila, ni bilo podvrženo dvomu (prim. Kadar je dvom, je prisotna tudi tesnoba);

Točno je vedela, kam mora pogledat: oči, ko poskušajo sledit temu gibu, ki je ta lik, med pavzo so bolj usmerjene, bolj avtomatsko se premaknjeno;

Ko ni te prepričanosti, pa ji oči preiskujejo prostor, kar se kaže kot da ta gib ni samoumeven in avtomatiziran, ni celota, ampak je neka medla ideja, kje bi pika lahko bila;

Diagonal ni pred gibom; malenkost ga prehitevata, vendar ga ne potrjujeta → nakazujeta zgolj splošno smer, gib in diagonala sta vezana eden na drugega;

Testni dražljaj: nima velike površine in je simetrične, točke so bile v linije in je ugoden. Bilo je kot da si je bilo treba zapomniti manj podatkov:

Istost: kar je imela kot ideja, ki jo je dopolnila, je lepo sledila prikazanemu; to je isto sledenje kot med spominskim dražljajem in pavzo;

Ko v testnem dražljaju potrdi napako, noč poslej ni relevantno.

88 S04-04-P-02

Sledi v smeri urinega kazalca;

Ni premišljevala o tem, da čas mineva in bo mogoče pozabila;

Na prvo pojavitev ni bila zares pozorna in rekla si je, da se bo malo sprostila;

Imela je občutek, da je bila še vedno tam; četrta pika ga je naredila simetrična in vzorec se ji je zdel naraven in jasen;

Ugotovila je, da ima podobo zmaja → vzdrževala je podobo zmaja, ki leti;

Testni dražljaj: če bi nanj projicirala sliko zmaja, bi ustrezalo;

Spominski dražljaj: ni bila pozorna na prvi objekt, ampak ga je vseeno lahko priklicala: gledala je v zaslon in načeloma bi ga morala videti in na nek način ga je, vendar ni bila pozorna; ko se je prikazal drugi objekt, je bilo kot da bi iz belo-vijoličnega prostora vzniknila pika od prejšnjega, ampak te barve niso bile homogene → vijolično-bel je bil le zunanji obris pike, ki je bil kakor da je zvezana s to mislijo, kako se rešuje ta naloga;

Pike ni zares videla, ampak ko je prišlo do testnega dražljaja, si je lahko rekla, da je to piko že enkrat videla;

Obris pike je bil kvalitativno podoben črtam v prejšnji nalogi, kljub temu da ni iste velikosti;

Zmaj: če bi zelo na kratko ošvrknila obseg, je to vodilo do asociacijo na oranžno rumenega zmaja na modrem nebu iz neke otroške slikanice;

Ti ni specifična podoba in med pavzo je videla cel ta prostor;

Zmaj je v njej vzbudil neka čustva, ki so bila pomirjujoča, vendar ne preveč intenzivna. Brez tega čustva se ne bi mogla spomnit te asociacije:

Modro nebo in oranžna: oranžno je videla, modra pa je med prikazovanjem testnega dražljaja bila v okolici tega dražljaja;

Zmaja je videla na zaslonu, zaslon onkraj je imel globino: zmaj je bil v nekem prostoru, ampak ta globina ni dodala nobenega pomena, noč ne pomaga, niti ni konstantno to v doživljanju; če jo vprašam po globini, potem jo ima; *(za Urbana: je to gist globine?)*

Vzklik, da je kot nek zmajček: njen glas, glasne kot ko govori, zamomljan, bilo je avtomatsko, ampak je bila ona avtorica tega glasu;

Testni dražljaj: pike so se prikazovale; imela je idejo zmaja in skladno s pikami je zmaj vztrajal; pike so idejo zmaja orisale;

Potrditev, da so pike na pravem mestu: črte zmaja se staknejo in tam, kjer so pike, se sestavi v to, kar je → iz tega ve, da ni neenako.

89 S14-04-C-01

Ni vedela, ali je desno zgoraj rožnato, ali zeleno. Ko ga je prvič pogledala, se ji je zdelo, da je objekt zelen in si je rekla, da si ga bo zapomnila, potem pa se je osredotočila na ostale tri;

Med pavzo ni več vedela, ali je zelena ali roza, kazala se ji je roza, ampak zdelo se ji je, da je zelena;

Ostale tri so bile en element (asociacija na slovanske narode), zadnja je bila pa *outlier;* vedela je, da če bi bil roza, bi ga grupirala s trojico, ker je bila tam rdeča;

Sklepanje: potekalo je v besedah, bilo je hitro z manj veznikov. Sama s sabo ima lahko ful krajši dialog, kakor z drugimi. Iz tega toka je sklepala na različnost, saj ve, da si ne bi postavila takšne miselne sheme, če bi bil testni dražljaj enak spominskemu;

To vedenje: kot da to ni najbolj optimalen način ta zapomnit + če bi bila rožnata, bi drugače postavila skupaj: njen *glow* je bil drugačen od spominskega dražljaja;

Način reševanja ji je povedal, da to ni isto;

Ko se je pokazal spominski dražljaj, je vedela, da si tega ne bi zapomnila, kakor si je; rumena in rdeča sta namreč skupaj tvorili nek element, ki ga prej ni bilo.

90 S14-04-C-02

Tri od štirih so bili enaki, na četrtem pa je bilo drugače:

Trojico je lahko potrdila, ker si je zapomnila, kje sta modra in rumena, zelena je bila pa njuna kombinacija; torej je sklepala na podlagi trikotnika, ki ga je registrirala kot barvo;

Testni dražljaj: rožnati objekt je porušil celotno dinamiko: pride z drugimi konotacijami kakor črna, ima drugačen *vibe;*

Če grupira na podlagi kromatike, je črna *outlier;*

Spominski dražljaj: levo zgoraj je črna, ki razen z belo ne kombinira z nobeno in je potemtakem *de facto outlier.*

Ugotovitev neenakosti: spominski dražljaj je imel nek obtežen občutek, kar je vzdrževala je imelo nekaj, ki je počasi bledel, ob testnem dražljaju pa se je pokazal nov naboj, ki se je prekrival z bledečim nabojem iz spominskega dražljaja; ta prehod ni bil lep;

Naboj: zeleno-črna kombinacija terja pozornost: občutek je, kot da je an avtocesti in mora biti pozorna na znake. Zeleno-roza kombinacija pa vodi v popolnoma drugačno dispozicijsko držo. Bolj je *chill.*

Pavza: ohranjevala je barve , z očmi je skakala čez položaje;

91 S16-03-O-01

Ugotovila je, da so objekti videti kot policijske lizike;

Med zadnjim primerom je ugotovila, da njena strategija ni efektivna, kar jo je malce zmedlo;

Asociacija na lizike: videla je sliko policija, to je bila jasna podoba, ki je zavzela medlo miselno sliko. Ni bila na ekranu. Bila je nekje za vidnim poljem in pred sabo je še vedno videla računalnik. Ni bilo zelo jasno vizualno. Sliko je nekako potegnila. Bila je tam, ampak naenkrat se je iz ozadja premaknila v ospredje, s čemer se je slika razjasnila. To se ji je dogajalo;

Ob tem je zaslišala glas: »O, policijska lizika!«, kar se je zgodilo istočasno kot podoba. To je bil energijsko nabit glas, imel je emocionalno noto (da je zanimivo) in to se ji je dogajalo;

Meta-kognicija: ugotovila je, da to ni učinkovita strategija.

Med reševanjem je slišala glas ki je govoril, da so različne smeri, ob čemer pa ni vedela, ali so med testnim enake kot med spominskim. Glas sam je bil drugačen od vzklika, da je kot policijska lizika. To so bila nadzorovana navodila sami sebi. Ni si jih konstantno ponavljala, vendar je imela občutek, da mora biti pozorna na različnost smeri;

Ob tem je doživljala napet, pozoren fokus. Ta *feeling* je izginil, ko se je pojavil testni dražljaj. Kazal se ji je kot nek pritisk v glavi, v čelnem delu ob spremljanju naloge. asociacija se je zgodila malo po tem;

Trenutek ko napoči pavza: njene misli so bile prazne in pojavil se je brezciljen občutek, kakor da si mora zapomniti te različne smeri, ko pa je spominski dražljaj enkrat izginil, ga je zamenjal tak izgubljen *feeling,* kot da ne ve kam točno naj gleda in da ne ve, kaj naj si zapomni;

Poskušanje, da si zapomni je imelo dve komponenti: 1) rekla si je, naj si zapomni; in 2) poskušala je ohraniti neko miselno sliko tega, samo da je bila tko zelo, zelo slaba in nejasna. Bilo je kot da gre k okulisti in sploh ne vidi. Kakor da vidi nekaj, kar nima zares jasne podobe. Ker je bila ta podoba tako nejasna, je to vodilo v občutek izgubljenosti; to podobo je videla spredaj, pred ekranom v njenem vidnem polju, kakor da jo je projicirala;

Objekti so bili videti, kakor da bi jih dal za neko zelo umazano steklo. Izgubili so svojo barvo in postali bolj svetli, kakor da bi bili prekriti z meglo; bili so razbarvano črni;

Osnovna forma se ohrani, je razpotegnjena. To je bilo enako kot pri okulistu

Sliko je projicirala; imela je občutek, da jo je potegnila iz čelnega režnja; ki se je potem prestavila ven;

Pavza: projcira to packasto sliko, obenem pa si ponovi njeno vsebino (enkrat ali dvakrat); ob projiciranju čuti nek pritisk, da bi si to morala zapomniti in da je narobe, če jih ne vidi. Bilo je kakor sram (prim. z okulistom, kjer jo je sram, da ne vidi nečesa, zdi pa se ji, da bi morala);

Asociacije ni bilo več, misel na to pa je prešla v ozadje;

Menjala je pozornost med vsemi temi miselnimi tokovi. Dogajali so se hitri preskoki med njim, doživljala jih je kot tokove (asociacija, govor, packasta slika);

Sram: čuden občutek. Bil je mentalen. *Feeling* je bil res, kakor da sedi pri okulistu in je bil zvezan s packastimi slikami;

Testni dražljaj: rekla si je, »so različne, ampak ne vem, če so prave.«. ta monolog je bil oblikovan kot besede, ki so bile kvalitativno podobne opombi, »različne so,« le da jih je prej spremljala samozavestna nota, zdaj so bile pa tišje, bile so poražene. Glas je imel intonacijo, z upadanjem samozavesti je upadala tudi intonacija. Čutila je jezo ob spoznanju, da to ni bila dobra strategija;

Spremljal jo je občutek poraza;

To si je rekla s svojim glasom, čuti se, da je ona njegova avtorica, čeprav je govorila z intonacijo, ki pripada načinu govora njene matere;

Prehod iz samozavestnega govora na spoznanje, da v resnici ne ve. To je obenem občutila malo fizično, kakor da jo je nekaj potisnilo navzdol v predelu ramen in glave. Kar naenkrat se je njen samozavestni glas spremenil v ta mami-glas. Ob tem ji je bilo neprijetno in ta prehod je bil negativen. To je bil še vedno njen glas, ki pa je postal pasivno-agresiven in se noče poistovesti z njim.

Čutila je ziritiranost sama nad sabo;

Čustveni tok: iz samozavesti je prešla v samokritičnost in ta je bila ločena od čustev povezanih z ohranjevanjem podobe;

92 S16-03-O-02

Osredotočala se je na spodnje dva;

Zadaj v glavi je čutila pritisk, precej močno tenzijo;

Rekla si je, naj bo pozorna na spodnja dva; te besede so bile ponovno oblikovano kot neko sprotno spremljanje;

Zgornjih objektov sploh ni videla;

Istočasno je čutila tok, ki je podajal navodilo ter tenzijo, ki je bila povezana s pozornostjo;

Imela je še nek dodaten občutek, ki je dvomil v to, če je to dobra strategija;

V resnici ji je bila stresna cela naloga;

Pavza: ohranjala je miselno sliko, ki jo je videla spredaj na ekranu. Objekta sta bila bolj jasna. Bila sta sivkatsta, kakor senca, v kateri je bilo razvidno, kaj predstavljata;

Čutila se je kot avtorica obeh teh tokov;

Med pavzo ni bilo monologa;

Ostala je tesnobnost od prej, poleg tega pa je čutila pritisk, da si mora zapomniti te dražljaje. Obenem je čutila dvom, povezan z njeno strategijo;

Misel, da ne ve, kakšna sta druge dva objekta, ki se ji je kazalo v pasivno-agresivnem sprotnem spremljanju ter občutku, da kar naredi, ni dovolj dobro. Glas je bil drugačen od tistega, ki je podajal navodila;

Testni dražljaj: videla je, da sta spodnje dva ista. Ob tem je imela stalen občutek, da za zgornje dva ne ve, kako sta videti.

Trenutek odgovora: odgovorila je, da sta enaka. Ob odgovoru je čutila nekakšen ponos, istočasno kot dvomeč notranji monolog in pritisk v glavi. Slednjega je sicer malce izgubila, ko je videla, da sta spodnje dva enaka, se pa je kmalu vrnil.

Odgovor je besedno zracionalizirala.

93 S16-03-C-01

Imela je razbremenjen, lahkoten občutek, njeno delovanje je bilo pa precej avtomatizirano.

Ni se čutila, da si mora kaj zapomniti, ampak je bilo vse zelo lahkotno.

Videla je vse štiri barve;

V glavi je na hitro šla čeznje in jih verbalizirala v zamrmranem tihem glasu, ki je bil nekako momljajoč. To je spremljal samozavesten občutek, češ da ve, kaj dela. S tem občutkom je tenzija od prej začela bledeti, ton glasu pa se je umiril;

Koščki vizualnega so se sami od sebe sestavili skupaj;

Meta-govor ni več podajal ukazov, ampak je samo govoril, kaj se dogaja.

Na neki točki je tenzija popolnoma izginila.

Pavza: ima neko bolj nejasno sliko in ni vedela, katere barve so. Kljub temu pa je imela neko podobo, nek občutek zadaj v glavi, da barve so, niso pa bile projicirane. Imela je podobo kvadrata, v katerem se pojavljajo krogi (za očmi je čutila svetlo sive kroge);

Vedenje da barve so: ni ohranjala tega, obenem pa je čutila pomirjenost, da ve, kako naprej. To je bilo estetskemu občutku, ki ga je čutila med nalogo z orientacijo; te barve do neke mere pašejo skupaj, oz. pašejo na neko mesto. Med testnim dražljajem tako preveri, ali je nek dražljaj enako estetski kot spominskim, ali pa ne.

Med pavzo ni bilo ponavljanja;

Čakanje na testni dražljaj: to je bilo umirjeno, ni čutila pritiska, da bo mogoče pozabila, vedela je, da bo dražljaj kmalu napočil in da bo vedela, kaj odgovoriti;

Testni dražljaj: takoj je videla ujemanje; za pozitivne občutke misli, da ona ne govori tega, kljub temu da je glas njen;

Ob ujemanju je to z umirjenim glasom verbalizirala: »okej, prav imaš, to je enako.«

Glas je bil akustično njen, ampak nenavadno samozavesten, kar ji je dajalo malce občutka distance;

94 S16-03-C-02

Spominski dražljaj: ko se je pojavil dražljaj jo je rdeča zelo zbodla, kar je čutila kot da ji je mušica priletela v oko, ob čemer je vzkliknila »fak, ta rdeča mi gre res na kurac!«

Potlej je ta rdeča ostala zelo močna; imela je malce bojevite občutke, ki so presegli verbalizacijo; ko si je enkrat ogledala še druge barve, je ta jeza zbledela. Ob tem si je rekla »okej, vse barve, v redu.«

Pavza: jeze ni bilo več. Rdeča je ostala v njenem levem očesu in videla je zamegljeno podobo, ki se ji je dogajala. Prej je ohranjala vizulani *feeling*, kakor da bi nekaj konkretno videla. Ostala pa je neka prisotnost;

Rdeča je prevzela njen fokus;

Ni bilo verbalizacij med pavzo;

Pomirjenost se je prelivala v ta občutek živčnosti ob rdeči;

Sprva je za ostale barve vedela, kakšne so, ampak jih ni ohranjala;

Testni dražljaj: nekaj jo je zmedlo in je narobe kliknila.

95 S16-04-P-01

Spominski dražljaj: poglede je sliko in ko so se pojavile kroglice, jih je začela povezovati med sabo s črto, ki ni bila vidna; bilja je bolj imaginarna. Ni imela barve in preprosto se je pojavila na ekranu;

Spominski dražljaj: zdi se ji, da je s pogledom nekako naredila gazo v prostoru;

Ta črta je sicer nastajala sama od sebe, ampak je na neki točki ugotovila, da je uporabna;

Črto je zarisala po tem, ko je en krogec izginil, drugi se je pa pojavil. To povezavo je ustvarjala, kakor da bi počasi vlekla linijo, dokler se pač ena ni odstranila;

Pavza: ohranjala je podobo teh črt na ekranu;

Ta pot je bila zelo očitna, saj so bile povezave precej svetle;

Ni bilo občutkov, kakor da se je ta črta pojavila izven ekrana, ampak je bilo vse skupaj na ekranu;

Testni dražljaj: potrjevala je, ali so se objekti pojavljali na črtah; kar se ji je kazalo prek tihih potrditev. Tiho si je govorila »ja.«

Meta-kognicija: medtem si je ves čas govorila, kaj počne. Ta glas je bil umirjen in top. Ta pritrditveni »ja« je bil temu glasu kvalitativno podoben. Na to podajanje navodil ni bil vezan noben afekt. Je le jasno nizanje verbaliziranih misli.

Ob tem ni bilo nobene estetske komponente, na katero bi bil vezan afekt ali preferenca.

Pavza: med pavzo je ohranjala črto;

Videla je sliko, vendar brez krogcev; prisotne so bile le črte, oziroma kjer bi črte morale biti;

Črta je bila fiksna. Ko jo je enkrat ustvarila, ni bledela s časom ali zmanjšano pozornostjo, ni pa bila zelo močna;

Testni dražljaj: Ko pika pade v črto, je to spremljal nek vesel občutek ob skladanju. Vsakič ko se pojavi en objekt, je čutila olajšanje, češ da je tam, kjer mora biti. V ospredju njenega doživljanja je bilo to malo veselje, ki je bilo zelo kratkotrajno. To je prešlo v navodila sami sebi, da mora biti osredotočena in med tem dvojim so se dogajali hitri preskoki;

Veselje: čutila ga je v prsnem košu, kot malo hitrejši pulz (vendar ne zares), občasno pa tudi v gornjem delu telesa, kjer jo je iz sredine prsnega koša malo povleklo navzgor. Del tega je bil tudi miselni: udeleženka je bila tekom celotnega intervjuja prekrokana in je zaradi tega čutila tenzijo v glavi, zaradi katere ne more točno opisati mentalne komponente tega veselja. Obenem se je zavedala, da je malce privihala ustnice;

Olajšanje: čutila ga je v prsnem košu, kot da je neka teža šla stran;

Ob ohranjanju pozornosti čuti nek pritisk, neko zakrčenost, ki se ob sovpadanju objektov v testnem dražljaju s črto sprosti;

Torej je obenem občutila, da jo nekaj dviguje in spušča. Sočasno je bil *uplifting* občutek in pa *feeling,* ki jo spušča dol, vendar ne na tak negativen način. Dvigovala se je, vendar ne v tenzijski drži. Bila je sproščena;

Meta-kognicija: Imela je jasno prisotnost teh navodil. Imela je občutek, da ji naloga gre dobro od rok. Imela je nek ponosen *feeling,* češ da ve, kaj dela in zato so bila ta navodila tudi tako zelo pomirjena.

Pavza: med pavzo je z očmi sledila smeri črt. Ta slika je bila sama pa sebi precej statična. Med spominskim dražljajem jo je zarisala precej počasi, med pavzo pa zelo hitro in potem je samo še držala pogled na to celotno kompozicijo. Ni da bi premikala pogled oziroma usmerjala pozornost na kako specifično črto, ampak je samo gledala v te črte kot celoto. *Torej je črte še enkrat narisala med pavzo.*

96 S16-04-P-02

Doživljanje je bilo zelo podobno kakor v S16-04-P-01, le da je ugotovila, da bolj premika oči v kateri se pike pojavljajo. Te gibi se ji zdijo zelo intenzivni. Jasno je premaknila pogled, da je dobro zaznala točko, kjer se je pojavila pika. Ni premikala cele glave, ampak je bil občutek, kakor da je premaknila svojo glavo v tisto smer, kjer se je pojavila pika. Medtem ko se ji je v S16-04-P-01 zdelo, da jih samo bežno spremlja. Zdaj pa je čutila, kakor da se ji celotno doživljanje prestavi tja, kamor je fiksirala pogled;

Te gibi so bili malo avtomatično, malo voljni. Njeno spremljanje je podalo ukaz, gib sam pa je bil avtomatski;

Imela je občutek, da sledi neki drugi zenici, da sledi pogledu nekoga drugega;

Kroge je doživljala kot štiri ločene objekte;

Pavza: ponovno nariše te črte;

V njen samozavestni občutek, pa se je med pavzo priplazil dvom. To je bil trenuten občutek, ki ga je doživljala kot negativen glas, ki ji je govoril, da mogoče pa to ni tako zelo učinkovita strategija. S tem glasom je bila tesno zvezana tesnobnost. To je doživljala kot zakrčenost v prsih ter ramenih in tenzijo v glavi;

Glas, ki jo spremlja: negativni glas je avtomatiziran, nevtralen in pozitiven pa je zavestno artikuliran;

Testni dražljaj: objektom sledi z očmi. Ugotovila je, da tretji objekt ni bil enak, ob čemer je doživela manjši šok ob neskladju ter razočaranje. Do tega spoznanja je prišla tako, da je avtomatizirano premikala pogled, ta pa ni našel pike, kjer se je fiksiral.

To je torej trostadijski proces: 1) s pogledom sledi črti; 2) doživi presenečenje, ker pika ni bila v tej trasi; 3) ob tem doživi razočaranje in trzne s telesom;

Razočaranje je dlje časa lebdelo v njeni zavesti in se je razlilo v naslednje primere. Definitivno je trajalo več časa;

Drugo: doživlja minevanje stvari, nima pa občutka koliko časa konkretno mineva;

Men neustreznim objektom si je govorila: »ja, ja, joj, zakaj pa ni!«

97 S17-01-O-01

Udeleženka si je zapomnila le leva dražljaja;

Vedno si je verbalizirala: npr. »cik, cak«

Med pavzo si je ponavljala te besede;

Med testnim dražljajem si je rekla »ja« ali »ne,« glede na to, ali je ustrezal;

V vidnem polju pasivno spremlja tudi desna dva; doživlja ju kot neka vsiljivca in ju med testnim prepozna le, če sta zelo drugačna;

Primerjava med testnim dražljajem: išče verifikacijo, ob čemer doživi nek prijeten telesni občutek;

98 S17-01-O-02

Doživljanje je zelo podobno kakor prej: osredotočena je na leva dražljaja, ob čemer verbalizira »noter, gor, noter, gore.« in ta »gor« je skodiran zelo grobo, saj predstavlja tako gor, kakor gor-desno in gor-levo;

Desna dražljaja doživlja kot vsiljivca in ju vidi kot nekakšno celoto in če zazna spremembo, ne ve, točno kaj se spremeni;

Med pavzo si je na glas ponavljala verbalizacije;

Testni dražljaj: doživljala ga je lahkotno, ker je vedela, kaj bo (to nalogo je pri Gregi reševala že prej);

Pri neujemanju jo nekaj zmoti;

Verifikacija: ob istosti čuti nekakšno olajšanje;

Trenutek odgovora: če za oba dva prek besed potrdi, da sta ista, je pritisk tipke avtomatičen;

Pred pritiskom tipke si je povedala, kateri gumn je kateri, ob čemer je malce premaknila prste;

99 S17-01-O-03

Na koncu ni bila prepričana v to, kaj je pritisnila;

V ospredju je imela doživljanje prstov;

Med testnim dražljajem je doživljala dvom. Ni bila prepričana in to je doživljala kot nek fizični občutek, ki ga je čutila v prstih;

V ozadju je doživljala desna dražljaja, ki sta bila kakor zadimljena;

100 S17-01-C-01

Pri barvah ni bilo nobenega govora;

Spominski dražljaj: objekte je doživljala kot celoto, barv ni povezovala, razen če so izstopale;

Z očmi je preiskovala dražljaj: pogledala je naokoli po celotnem dražljaju;

Pavza: v primerjavi s prejšnjo nalogo (orientacije) je bila pavza manj skoncentrirana;

Zaslišala je ptice: ob tem je začutila neko prijetnost (nek pritisk se je zmanjšal; ta pritisk je bil v osnovi fizičen);

Med pavzo ni več doživljala celote barv;

Med testnim dražljajem sta se pojavljali dve opciji:

Če je barva izstopala, se je orientirala po njej;

Drugače je bila pozorna na celoto.

101 S17-01-C-02

Med spominskim dražljajem je iskala izstopajočo barvo: pregledala je vse objekte in v kolikor te barve ni, se je osredotočila na celoto;

Fiksacijski križ je nase prikoval njeno pozornost;

Pavza: ni se trudila, da bi karkoli ohranjala;

Pri izstopajočih barvah se ves čas zaveda, da zelo dobro ve, kaj je odgovor;

Pravilnost: gleda fiksacijski križ + če se ji zdi, da so vse enake, če pa kaka barva izstopa se ji pogled usmerja tja;

Večino časa pogled ostane na fiksacijskem križu.

102 S17-01-C-03

Pavza: slišala je neko glasbo; poleg tega pa ponovno ni bilo nobene posebne aktivnosti;

Ni bilo govora;

Zapomnila si je neko sliko, celoto; včasih pa so iz nje padale določene izstopajoče barve;

Celota: to niso barve, ampak slika, iz katere posameznih barv ne bi mogla izvleči, same barve pa so bolj intenzivne kot so v resnici. Slike ne vidi med pavzo in bolj gre za neko vedenje;

Vedenje, da je isto ali ne, se spet zgodi prek neke verifikacije;

103 S17-01-C-04

Spominski dražljaj: ne vidi zares slike, ima občutek, da se nekaj zgodi v čeli. Pogleda sliko in vidi celoto;

Pri odgovoru nekega vedenja ne ozavesti zares, prsti jo preprosto prehitijo;

Celota: ne vidi je kot 4 kroge, samo zazna, da ima štiri lokacije;

Če preveč posveča pozornost tej celoti se bo zmotila;

Ob tem ni posebej močnega doživljajskega prostora, z izjemo fiksacijskega križa na sredini;

Še preden je pomislila, ali je dražljaj enak ali drugačen, je že pritisnila na tipko;

104 S17-01-C-05

Spominski dražljaj: vidi vse štiri barve;

Testni dražljaj: barve so se ji zdele iste;

Spominski dražljaj: barve doživlja kot celoto, kot neko sliko in to je bilo popolnoma vidno doživljanje;

Testni dražljaj: ni vedela, zakaj je odgovorila, kakor je;

Bila je precej, vendar ne 100% prepričana v pravilnost svojega odgovora. Ve, kako se zave, da se je zmotila: telesen občutek, ki je kot da jo nekaj malega na hitro strese;

Med pavzo ni bilo posebne aktivnosti; zgolj čakanje.

Pomislek: V enem primeru so bile barve nemške zastave; ni si rekla »Nemčija« ampak je vedela, da je to to.

105 S17-02-P-01

Zdelo se ji je, da se pike pojavljajo skladno s srčnim utripom;

Spremljala jih je akustična komponneta, nekakšen »hm,« ki je bil kakor momljaj. Ne ve, če si ga je govorila. Je pa definitivno slišala to.

Slišala je tudi intonacijo. Ritem je bil vezan na zaporedje pojavnosti;

Ni gledala celote, ampak je sledila piki;

Zaporedne je gledala v pike in ni zrla zgolj v eno točko;

Pavza: preprosto je čakala, da pike pridejo nazaj;

Testni dražljaj: v ritmu je sledila pikam. Pravilnost je doživljala kot ravnovesje, da se nič ni spremenilo. Ravnovesje se kaže kot mentalna umirjenost;

Pritisk na gumb: ponovno ga je spremljala verifikacija, ampak drugače kot v prvem srečanju;

106 S17-02-P-02

Spominski dražljaj: vidi posamezno piko in vsaki sledi z očmi , vendar ne gre strogo do nje. Tiste, ki so bile bliže druga drugi je pogledala s pozornostjo, tiste, ki so bile daleč pa z očmi;

Telo šibko se je zavedala kinestetike oči, se je pa zavedala razdalje, kadar je bila ta velika;

Gibi so bili do neke mere avtomatski;

Pavza: v nekaterih trenutkih je v glavi imela ta ritem in momljanje;

Testni dražljaj: pogledala je vsako piko posebej in preverila, ali je ista;

Če so bile vse iste, je doživljala neko ravnovesje, kot da se ni nič spremenilo; med ne-istimi je bilo doživljanje drugačno, vendar se ne spomni, kako;

Meta-kognicija: pri tej nalogi ni imela nekega občutka, da je naloga zahtevan in njena pozornost je bila bolj lahkotna;

107 S17-03-O-01

Gledala je leva dva; pozorna je bila na repke in je verbalizirala njihove smeri;

Med pavzo si je ponavljala usmeritve;

Pozornost je bila osredotočena na leva dva, pri desnih pa je zaznavala le velike spremembe;

Pozornost je doživljala kot nek zakrčen pritisk v glavi;

Ker so krogi vedno isti, je bila le redkokdaj pozorna nanje;

Usmeritve si je ponavljala z besedami v svojem glasu, ki je bul malo tišji. Besede same so bile jasno slišane (»gor, dol, cik, cak, ipd.«). Počutila se je kot avtorica teh besed;

Usmerjenost je doživljala vizualno, bilo je kot slika in ni bilo neke prostorske komponente;

Glas je iz spominske dražljaja prešel v testnega;

Desna dva je odmislila in zavestno je izbrala leva dva, kadar pa je slučajno zaznala desna dva, je bilo to zelo izven njenega nadzora;

Testni dražljaj: istost je zaznala, kadar so se repki ujemali z govorom. Govorila si je usmeritve iz spominskega dražljaja in to so bile vedno iste besede;

Vedela je, da bi »gor« vedno ustrezala določeni obliki. To je bilo zelo semantično. Videla je neko usmeritev, za katero je vedelo, kako bi jo opisala, vendar tega ni zverbalizirala;

108 S17-03-O-02

Doživljanje je bilo zelo podobno kot pri S17-03-0-01.

Malo bolj je spremljala desna dražljaja;

Pri testnem dražljaju je začela preverjati dražljaje s tisto besedo, ki jo je ravnokar artikulirala, ni pa nujno, da se je to dogajalo v nekem prostorsko določenem zaporedju;

Zaslišala je tiktakanje ure, kar jo je najprej zmotilo, potem pa si je začela pomagati s tem, da je besede izgovarjala v ritmu ure. Začetni zvok pa je zmotil ritem izvajanja naloge;

Tiktakanje zasliši kot dva akustično razločljiva zvoka. Da bi ga odmislila, bi se morala močno potruditi;

Desna stran: v otadju je videle neke sledi; pozornost na teh dveh objektih je bila manjša, saj je vedela, da ne more obravnavati vseh štirih. To vedenje se ji je jazalo kot neprijetno. Raje ima pozornost na nečem, kjer bo vedela, da si lahko zapomni. To »navodilo« se ji je vzpostavilo kot neka norma reševanja in samo po sebi ni bilo eksplicitno.

Leva dražljaja sta ji bila preprosto domača, kar se ji kaže tako, da avtomatično pogleda leva dva;

Občutek, da bo znala odgovoriti: imela je neko sigurnost v svoj odgovor. To se ji je kazalo kot pomirjenost za razliko od neprepričanosti.

Če ve, zelo prepričano pritisne na gumb, kar pa samo po sebi ni preveč močno občutje.

109 S17-03-O-03

Gleda leva dva, ju verbalizira in potem prepozna istost;

Ob neujemanju je zadnje klikanje bolj nesigurno; vedenje se ji je zdelo bolj medlo, ker ni vedela, če se je desni kaj spremenil; ni čutila podkrepitve, ki jo čuti ko zagotovo ve, da je ena od levih nepravilna;

Ima boljši občutek, kadar ve, da je ena od levih zagotovo napačna;

Pri samem prepoznanju imamo torej tri opcije:

Leva je napačna (doživlja sigurnost);

V desnem zazna veliko spremembo (sigurnost);

Leva ista, za desna pa ne ve (nesigurnost);

Ko je nekaj narobe, je njen gib bolj avtomatiziran.

110 S17-03-C-01

Osredotočala se je na fiksacijski križ, ker se ji je tako zdelo, da vidi vse štiri kroge;

Ni bilo ponavljanja med pavzo, preprosto čakala je na testni dražljaj;

Pri testnem dražljaju je preprosto vedela, ali je isto, ali je drugače;

Fiksacijski križ se ji kaže kot neka točka, v kateri je fino gledat; avtomatsko, iz prve centrira svoj pogled tja;

Ko je gledala kroge, je imela občutek, da se ji to dogaja;

Pozornost: z lebdečo pozornostjo je zajela vse štiri objekte; krogce je (mogoče) videla ostro;

Pavza: nek prazen vmesni čas; vmes je imela neke misli, ki so prišle same od sebe. Razmišljala je o ptičjem petju in o uri, obenem pa je imela tudi vsebinske misli, ki jih je zlahka odgnala stran;

Testni dražljaj: vedela je, ali je enako ali drugače. Ne ve, kako. Točno je občutila to istost. Vedela je takoj, ko je videla dražljaj. Zdelo se ji je, da je vse skupaj šlo počasneje kot prej;

Občutek gledanja sam je bil podoben kot v prejšnji nalogi;

111 S17-03-C-02

Fiksacijski križ je videla ostro, krogi okrog njega pa so bledeli;

Med pavzo je doživljala nek prazen čas;

Testni dražljaj: takojšnje zavedanje istosti; Mogoče se je tega občutka zavedala v prsih, vendar se je večinoma nahajal v glavi;

Imela je občutek, da bo znala odgovoriti: imela ga je, glede na prejšnje izkušnje in je vedela, da se bo celota shranila in da bo znala prepoznati enakost testnega dražljaja;

Ta občutek je bil drugačen od občutka v prsih. Bil je kvalitativno drugačen od vedenja. Ta občutek je za trenutek prevladal; gre za občutek, da se nekaj še ni zgodilo to, da bo vedela, je manj konkretno od tega, da ve.

112 S17-03-C-03

Zavedala se je fiksacijskega križa in celote objektov;

Na začetku je začela razmišljati o občutkih, o sami gesti refleksije, ker je menila, da tudi brez tega gre. Pustila si je razmišljati. Kazalo se ji je kot nejasni, odsekani fragmenti govora v njenem glasu; so malce tišji, to ni bil nek kompleksen monolog. Ta samogovor se ji je do neke mere dogajal;

Zadnji primer je bil napačen in preprosto vedela je, da testni dražljaj ni enak;

Vedenje je avtomatsko vodilo v klik;

Vedenje neenakosti ni vizualno; zadnji gib je bil popolnoma avtomatiziran;

113 S17-04-O-01

Govorila si je kakšni so objekti (»cikcakasto«), vse od spominskega do testnega dražljaja je ponavljala te besede, na koncu pa je prišlo do spoznanja enakosti;

Te besede so bile voljno artikulirane v njeni glavi;

Enakost: doživljala je nekakšno ravnovesje, če je enakom če pa ni, pa se nekaj podre. Če ni skladno s tem, kar si govori, jo nekaj zmoti;

Če se ne podre, se take ravnovesja ne zaveda aktivno; preprosto je v nekem *flowu*, kjer se poslužuje strategije, ki se ji *de facto* daje;

Spremembo zazna še preden jo artikulira, to je še posebej res za desna dva, ki ju zavestno prezre med spominskim daržljajem in jo ob večji spremembi preprosto nekaj dregne;

Zmeda: kot ko se ove neenakosti, malo manj konkretno pri desnih dveh, neujemanje ustavi tempo reševanje; ta tempo si ustvarja ona sama prek govora. Če pa resničnost ne ustreza temu, kar si govori, se vse skupaj poruši;

Desna dražljaja nista del tega tempa; razen ko je razlika dovolj očitna, da jo zmotu;

Pozornost: zaobjema oba leva objekta, v ozadju pa popolnoma nezavestno spremlja desna dva;

114 S17-04-O-02

Govorila si je »gor, desno,« to pa je ponavljala dokler ne pride do drugih dveh objektov v testnem dražljaju;

Vzpostavi se ritem, na koncu katerega popolnoma avtomatično odgovori;

Enakost prepozna iz tega, da je prvo in drugo preprosto enako;

Za desna: med pavzo iz njiju ne bi mogla izluščiti posameznih enot; doživlja ju kot celoto, skupno sliko, ki pa ni vizualna (med pavzo je ne vidi);

Pavza: ponavlja verbalizacije;

115 S17-04-P-01

Pogleda vse štiri; njihova pojavnost ima ritem;

Med pavzo gleda fiksacijski križ in čaka, da se pojavi prvi objekt;

Med pojavljanjem objektov med spominskim dražljajem sliši akustično momljanje (ki je bilo sicer manj jasno kot nazadnje, ko je reševala to nalogo). Imeli so neko intonacijo, ki ni bila vezana na njihovo razdaljo od fiksacijskega križa, ampak na to, kdaj se je objekt pojavil;

Ob tem ni videla paslik;

Prostorsko se je ovedla pike, le če se je pojavila zelo blizu fiksacijskemu križu;

Pavza: še vedno je slišala te zvoke in to je bil en neprekinjen tok;

Enakost: z vsakim zvokom je bila v njenem spominu vtisnjena neka lokacijo (in to je bilo sočasno);

Lokacija o spominu je bila zvezana z občutkom prostora;

Prepričana je, da ve, kje je bila prej pika in to je zelo lahkotno reševanje naloge;

Testni dražljaj: če ni bilo ujemanja, se je zvok ustavil in to je bilo zelo avtomatsko;

116 S17-04-P-02

Ko se odloči, da testni dražljaj ni napačen, spusti pozornost in sploh ne registrira tega, kar sledi na ekranu;

Spominski dražljaj: pogleda vse, kar spremlja akustični momljal (ta glas je fiziološko neponovljiv);

Ostale so neke paslike, ki se jih ne bi zavedala, če prej ne bi govorila o nji;

Med pavzo je ostajal zvok;

Ni bila zares pozorna na njihovo lokacijo, ampak je imela občutek, da ve, kje so;

Zvok: zdi se ji, da je to njeno momljanje, vendar se pojavi samo od sebe ob pojavnosti kroga;

117 S17-04-C-01

Pojavijo se štirje krogi, ona pa gleda v fiksacijski križ in jih doživlja kot eno sliko;

Vmes ni bilo ponavljanja, le čakala je;

Avtomatično prepozna ujemanje;

Opazila je, da ima nek prostor, da razmišlja: lahko si je pustila, da nekam odtava; misli je ni napolnjevalo ponavljanje ali zvok od prej, lahko je razmišljala, a se je vmes zavestno ustavila;

Te misli je lahko prekinila, ne ve pa, če jih je zavestno priklicala;

Imela je en tok misli, ki ga je do neke mere nadzirala;

Misli so bile verbalizirane, v njenem glasu, ki je bil tišji;

Ujemanje: prijetno je, če se ujema. Nek občutek ravnovesja, če pa ne, pa motnja tudi ni tako velika. Pri ujemanju je to neka pozitivna nevtralnost, ki teče. Ko ni ujemanja, se to malo ustavi, ampak ni velikega preskoka;

Celota: krogi so bili ena slika, ne pa štirje diskretni objekti;

Med pavzo se te slike ni zavedala, niti ni mogla izluščiti posameznih barv, če ena izmed njih ni močno izstopala;

Pomisleki: Free Energy Principle;

118 S17-04-C-02

Med spominskim dražljajem se je osredotočala na celoto;

Med pavzo je čakala;

Na koncu je prepoznala ujemanje;

Vmes med pavzo barv ne more izluščiti, razen če so neke zelo izstopajoče barve;

Bi vedela, če so iste barve na drugih mestih: vedela je, kateri del je napačen, ni pa nujno vedela, zakaj;

Neujemanje: doživlja, da ena četrtina ni pravilna, da je tam nek manko, neko ravnovesje se tam poruši, vendar to ni neka močna motnja;

Rekla je, da ima sliko v spominu: ne ve točno, kaj ima v spominu; v grobem ve, kaj je bilo, vendar tega ne bi mogla analizirati;

Ve, da si je zapomnila in ni imela občutka, da ni vedela;

Pomisleki: Ali ta strategija sploh zadošča definiciji VPDS, če z »reprezentacijami« ne more manipulirati?

119 S19-01-O-01

Ob tem, ko je videla posamezne objekte, si je govorila »gor, levo, desno.« To si je govorila, vendar nemo. Ob tem ni čutila nekega telesnega občutka na jeziku ali v grlu. Imela je le nek občutek artikulacije, ki ga ni spremljala nobena zvočna komponenta. Ona je bila avtorica teh besed. Čuti, da je to počela in da se ji to ni dogajalo. Zaveda se namena (hitro poglej in si zapomni);

Že pred začetkom primera je čutila pričakovanje, ki se ji je kazal kot rahel pritisk v glavi in fokus na ekran. Telesni občutek tega pritiska je bil intimno zvezan s tem fokusom. To se je dogaja pred in med spominskim dražljajem;

Spominski dražljaj: gib z očmi je bil nekako onkraj njenega doživljanja. To je bil avtomatičen gib. Ni se zavedala svojih oči.

Meta-kognicija: čutila je rahlo tesnobo, ker si ni uspela zapomniti teh nemih verbalizacij. Tesnobo je doživljala kot medel cmok v grlu. Sama tesnoba ni bila tako močna. To, da si mogoče ni uspela zapomniti, je vedela, ker ni prišla do konca objektov. Dražljaj si je poskušala vtisniti v spomin in ko je napočila pavza, ni več vedela, kako je bilo.

Poskušanje vtisniti v spomin: nema verbalizacija + vizualno si je poskušala vžgati v spomin → to je doživljala kot zelo osredotočeno pozornost, oziroma poskus osredotočene pozornosti, ki jo je spremljala tesnoba, da si vsega skupaj ne bo zapomnila;

Ta pozornost je bila taka, da je samo gledala v objekte.

Bolščanje in nemi govor sta se dogajala istočasno;

Zadnjega lika med spominskim dražljajem si ni uspela zapomnit, videla pa ga je.

Pavza: pavza je bila napolnjena s pričakovanjem, kdaj se bo pojavil testni dražljaj. Skoncentrirano je gledala v belino ekrana, ob tem pa je čutila rahlo tesnobo in cmok v grlu. Ta tesnoba je ista stvar kot na začetku, ki je izginila ko si je začela zapominjevati in se spet pojavila med pavzo. Sicer je pa to majhna tesnoba. To ni nekaj močnega.

Pri spominskem dražljaju sta tesnoba in pozornost tekmovali za to, katera bo v ospredju;

Med pavzo ni bilo tekmovanja med tesnobo in pozornostjo, le obe sta bili prisotni. Kvalitativno sta bili drugačni med pavzo kakor med spominskim dražljajem. Pozornost je bila bolj usmerjena (to je bilo to pričakovanje: gledala je v križec in ni gledala ne levo ne desno, samo čakala je), tesnoba pa je bila malo manjša.

Fiksacijski križ je bil onkraj njenega doživljanja;

Med pavzo ni bilo ponavljanja besed;

Testni dražljaj: Bolj kot na besede se je naslonila na občutek ali je prav ali ni prav. Zanašala je na to, da bo istost razbrala iz občutka. Ta načrt, to računanje da bo vedela iz občutka se ji je kazal kot da bi moralo vzniknit nekaj iz neke globine, iz prsnega koša. Zanašala se je na nek telesni občutek;

Odločitev o odgovoru: odločila se je na podlagi tega občutka. To je bil preplet neke pozornosti in občutka v prsnem košu, na podlagi česar se odloči, ali je prav, ali narobe. Ne ve sicer ali je prav ali narobe (pritisnila je, da je enako). Telesno je preprosto vedela.

Pritisk na tipko: rahlo ji je bilo neprijetno, ker ni vedela, ali je prav (zanašala se je na občutek, da je dražljaj mogoče isti, ampak ni pa vedela, ali je isti). Upala je, da se bo vse to zlilo v neko gmoto in da bo tako dobila odgovor.

120 S19-01-O-02

V mislih si je govorila »gor, dol, levo, desno,« vendar ne s konkretnimi besedami, ampak v neki spakedravščini, kakor da je okrajšala besede, ker ni bilo časa za njihovo polno artikulacijo;

Obenem si je spominski dražljaj poskušala vžgati v spomin;

Pavza: Poskušala je držati sliko v mislih in si hkrati govoriti okrajšave teh besed;

Spakedravščina: ni bilo neke akustične komponente; imela je občutek, da sliši, vendar ni bilo kaj za slišat. To je bil mentalen občutek. Proizvajala jo je voljno in čutila se je kot avtorica teh besed;

Vžiganje slike v spomin: spet je preletela dražljaj z očmi in si poskušala zapomnit sliko, jo držat v spominu, brez da bi jo analizirala. Kot tako, kot celoto jo je hotela zadržat v spominu;

Celota: doživljala jo je razgibano;

Fokus je bil bolj prisoten kot v prejšnjem primeru, ker ni bilo tesnobe;

Meta-kognicija: imela je občutek, da si je like zapomnila bolje kot prej. To se ji je kazalo tako, da se je telesno sprostila, ramena je znižala. Ob tem je čutila veselje, ki sicer ni bilo močno, ampak je bil prisoten nek rahel občutek. To je bil le en moment, ko je pritisnila na tipko po testnem dražljaju.

Pavza: sliko si je držala v glavi. Čim večkrat je poskušala it čez njo. Sliko je videla v glavi, ni bila čista. Spodnji levi konec je migetal. Zraven je slišala spakedravščino. Sliko je videla pred ekranom in (mogoče) je videla ekran onkraj nje. Objekte je videla črne. To sliko je bilo malenkost naporno ohranjati, predvsem zato, ker ni bila čista. V resnici je pavza minila prehitro, da bi vsa zadeva postala naporna;

Sproščenost, ko ve, da si je dobro zapomnila: prišla je po testnem dražljaju, ko je vedela, da je dobro odgovorila;

121 S19-01-C-01

Najprej je poskušala pogleda celotno sliko, potem pa posamezne krogce. Ob posameznih pogledih je v svoji spakedravščini povedala imena barv.

Pavza: v mislih je obdržala sliko;

Spominski dražljaj: poskušala je vizualno zaobjet celotno sliko, si jo vtisnit v spomin in jo začutit. Hotela je dobiti občutek celote: če so bile svetle barve, je bil občutek zračen, lažji; če so bile temne, je bil občutek bolj gost.

Spominski dražljaj: 1. zajeti celoto + občutek; 2. ena po ena + spakedravščina;

Premiki z očmi so bili zavestni, odločila se je, kako jih bo premikala, to pa je bilo zelo zvezano z njeno pozornostjo;

Spakedravščina: voljna, nema;

Poskušanje zadržati sliko v mislih: poskušala je razširit pogled na vse in nekako res vžgat sliko. Tega ni počela namenoma, samo zaobjela je sliko. Pogledala je celoto in se oprijela te podobe; to vžgano celoto je videla pred ekranom. Ta celota je sestala iz štirih konkretnih krogov, ki jih je videla nad ekranom. Obenem je bila bolj prepričana kot je bila prej (prepričanost: telesna umirjenost, sproščenost, odsotnost beganja);

Občutki za barvo: to je nekaj, kar ustvarja ona. To je nek precej telesen občutek. Začuti gmoto, ki glede na barve prejme neko obteženost (kot neka toplina, kot nekaj žametnega), neko lahkost (lahkost, denimo, je začutila kot neke vrste gmoto, ki je lahka v telesu, kot sproščenost);

Pavza: v spakedranščini si je ponavljala imena barv, enkrat do dvakrat, obenem pa je ohranjala sliko nad ekranom ter telesne občutke;

Testni dražljaj: z očmi se je premikala ena po ena in je pogosto že na tej točki vedela, kakšen bo njen odgovor. Iskala je neko napako, potem pa je vse ostalo spustila. Spustila je pozornost in sliko ter spakedranščino, ki jih je prej zadrževala. To izgine vse naenkrat.

122 S19-01-C-02

Spominski dražljaj: najprej je pogledala enega po enega, potem je pogledala vse. Ko je pogledala enega po enega je v spakedranščini povedala imena barv.

Pavza: umirjeno je čakal na testni dražljaj;

Tokrat ni bilo teh občutkov, ampak v manjši meri kot prej. Ponovno jih je ona ustvarila.

Sliko si je še vedno poskušala vžgati v misli, ampak do manjše mere. Bolj lahkotno je odgovarjala. Pozornost ni bila tako močna;

Ni bilo tistega fokusa, ko greš v ekran; ta pozornost je bila zelo *flowy* in je kar padla vanjo. Doživljala jo je manj telesno kot prej;

Testni dražljaj: objekte je pogledala enega po enega in zagledala je eno napako, na kateri točki je spustila vse;

Napačnost: občutek zadovoljstva, ker je vedela, da je napačno; kot neka notranja umirjenost, zadovoljstvo (čutila ga je na obrazu pri ustnicah, kot nasmešek, vendar se ni zares nasmehnila):

Vedenje, da je napačno: to je gotovost, ki se je kazala z umirjenostjo, da je lahko spustila vse; dovolila si je spustiti vse; pred tem je doživljala ekstremno pozornost, ki ni bila zares zahteva, ampak kot nek trud obdržati to v mislih;

Spustiti vse: to je nekaj, kar ona naredi;

Pavza: ponavljala si je spakedranščini in poskušala videti sliko. Videla je kroge na beli podlagi. Ni imela občutka, da se oži prostor, ampak kot da je *copy-paste* slike z ekrana. Misli, da ni videla resničnega ekrana onkraj tega;

123 S19-02-P-01

Spominski dražljaj: lovila je točke, ki so se prižigale;

Pavza: ponavljala je v ritmu; predstavljala si je prižigajoče se črte;

Vizualno je sledila oblikam; to je počela med pavzo;

Pavza: Videla je črne pike na beli podlagi, sivo-črni obrisi so ostali na ekranu. Te podobe je ustvarjala prek pozornosti.

Pozornost je doživljala tako, kot da je iz spomina v istem ritmu vlekla pike. Ta pozornost je bila precej nežna, ni bila to močna koncentracija. Zdi se ji, da je preprosto padla vanjo;

Ritem: ritem se je vzpostavil že med spominskim dražljajem in je bil kakor da je štela »1, 2, 3, 4.« Te številke niso bile dejansko verbalizirane. Le telesno je čutila ta ritem v tročetrtinskem taktu, v kakršnem je recitirala. V tem pa ni bilo nobene akustične komponente;

Objekt je doživljala kot štiri različne pike;

Med pavzo je sledila pikam: z očmi je šla po isti poki kot so šle pike. Šla je po isti sledi; kjer pa so bile pike, so ostale bolj blede podobe; povezave med njimi niso bile izrazite (ni vlekla črte) in doživljanje je bilo zelo diskretno;

Meta-kognicija: imela je občutek, da si bo lahko zapomnila nalogo; čutila je nekakšno umirjenost in odsotnost tesnobe, bila je sproščena. To je bila obenem telesna in mentalna sproščenost.

Pavza: ona je bila avtorica gibov; vendar ni doživljala neke namere. Ona je bila le iniciatorka tega giba, preprosto je naredila še en krog;

Testni dražljaj: gledala je, če bodo pike na enakih mestih. Poustvarila je svoj vzorec in gledala, če se je to skladalo s testnim dražljajem. Iskala je predvsem kdaj ne bo enako;

Skladnost: odsotnost je doživljala pozitivno, ker je vedela, da ima odgovor. To je bila neka notranja pomirjenost;

Vedenje, da ima odgovor: to je šlo ven iz njenega ritma reševanja naloge, ritem je bil enak, vendar ni imel njene smeri. Ob tem pa je čutila prijeten občutek. Pika, ki bi po njenem pričakovanju morala biti tam, je bila nekje drugje. Občutek je imela, da je skočila iz njenega *flowa*.

124 S19-02-P-02

Spominski dražljaj: sledila je pikam;

Pavza: videla je pike, sivočrne, ne popolnoma črne, videla jih je pred sabo;

Vse je bilo povezano z ritmom, ki je vzpostavljal *flow*. Ta ritem je bil tih, odsekan, *stacatto*;

Testni dražljaj: enakost: oči je premikala skladno s tem, kjer je pričakovala, da se bo pika pojavila. Tik preden bi se po ritmu morala pojaviti, je na njeno mesto usmerila svoje oči. Ko se ni pojavila, je dobila občutek, da to ni to, da se ne sklada, da gre ven iz ritma. To je zelo mentalno doživljanje, to pa ni bilo razumno sklepanje, (češ »aha, šla je ven iz ritma, torej neustreza«), ampak je bil nek bolj pretanjen občutek. Kazal se ji je kot nek aha moment. Pike ni bilo tam, torej je moralo biti nekaj tam. Ko ni videla pike tam, kjer jo je pričakovala je spustila ritem in sliko, ki jo je imela v glavi;

Trenutek spuščanja: to je padec pozornosti, ki ga je ona storila;

Meta-kognicija: občutek, ali si je uspešno zapomnila dražljaj: ni imela posebnega občutka;

Prim. S19-01: Ustvarjanje občutka: to v tej nalogi ni bilo prisotno;

Ritem: čutila je utripajoč občutek v ozadju doživljanja;

Trenutek odgovora: vedenje, da ve: njeno vedenje je bilo v veliki meri vezano na pričakovanje, ob čemer je čutila rahlo zadovoljstvo in spusti pozornosti;

125 S19-02-P-03

Zelo se je osredotočala na vprašanje, *kako je vedela, ali je testni dražljaj enak* in ni bila tako zelo pozorna na preostale dele naloge;

Testni dražljaj: z očmi je sledila objektom in ni bila tako zelo pozorna na to, kako so skakale pike, ampak se je osredotočala na to, kdaj se bo pri njej pojavil ta občutek vedenja. Zdi se ji, da ji je to malo uničilo vse izkušnjo;

Vse kar je bilo, je bilo pričakovanje tega trenutka, ko bo vedela ali je prav, ali narobe;

Preveč se je osredotočala na iskanje tega občutka in ga potem ni bilo, ker je bila druga;

Gesta refleksije: nekje vmes je spoznala, da če bo pozorna na to, kdaj bo ta občutek prišel, ne bo tako pristen, ali pa ga sploh ne bo;

Pristen občutek bi bil nemoten, ob njegovi pojavnosti ni prisotna analiza. Če pa nanj prilepi neko mentalno občuteno vedenje, pa se ji zdi, da je ta občutek nekako spremenjen;

126 S19-03-O-01

Spominski dražljaj: zapomnila si je samo prve tri; v obliki črke *Z* je preletela objekte, ker drugače bi bilo preveč;

Pavza: videla je te črtice, obenem si je pa govorila in sicer okrajšane besede (njena spakedravščina);

Med pavzo je videla medle črte, sivočrne;

Testni dražljaj: preverila je prve tri in se odločila, ali je prav, ali ni prav;

Spominski dražljaj: ubrala je drugo taktiko in to ni bila zavestna odločitev, preprosto začela je začeti nekaj novega in naknadno (med reševanjem naloge) je ozavestila to (med gesto refleksije je zaznala, da si v spomin ne vtisne cele slike);

Premikanje pogleda v obliki *7:* ta gib ni bil načrtovan, preprosto naredila ga je. Bil je pod njenim nadzorom v smislu, da bi ga lahko prekinila. Malce se je zavedala premikov, ne pa oblike oči.

Izginotje spominskega dražljaja: bilo je popolnoma vizualno izkustvo. Ni izginil, ampak je zbledel, kakor da so ostale sledi, kakor da ga je povleklo v ekran;

Pavza: videla je črte, krogi so zbledeli, to pa je spremljala verbalizacija. Tudi črte so bile blede, v smislu da so bile sivočrne, kroga pa niti ni videla celega. Videla je malce kroga takoj ob črti, vse onkraj pa je preprosto izginilo. Ko je videla v mislih te podobe je ponavljala gib sedmice (dvakrat ali trikrat) pri čemer je premikala oči. O tej bledi podobi ima občutek, da jo je doživljala pasivno. Odmislila bi jih lahko le, če bi odvrnila oči;

Verbalizacije: slišala je neko gmoto povezanih črk, ki nimajo pomena, ampak ona je vedela, da je v njih pomen *gor, dol, levo.* To je bila neka prepletena čačka in gmota črk. To je doživljala vizualno. Kakor če bi v mislih zarisala čačke, mogoče pa je bila vmes kaka čačka. To vidi pri vsaki črti. Zdi se ji, da te podobe prihajajo iz nje, ne spomni pa se akta ustvarjanja. Te čačke so črne na beli podlagi (vidi tudi belo podlago). Ta podlaga je nekaj drugega, kakor ta ekran. Podlaga se konča, kjer se končajo znaki in ne gre čez. Uokvirja te podobe. Čačke – te gmote – pa zavzemajo približno polovico tega. Sveta onkraj tega ne vidi. Pri teh besedah je tudi neka akustična komponenta: to je ta nem glas, ki je njen. Ta spakedravščina je močno zvezana s to vizualno podobo;

Odločila se je ali je testni dražljaj pravilen ali napačen: to je bil nek *gut feeling*. Nahajal se je nekje v njenem trebuhu, to je bil umirjen občutek, kakor da bi se nekaj usedlo, položilo.

Trenutek odgovora: nima ozaveščeno, da bi ga kaj posebej doživljala;

127 S19-03-O-02

Doživljanje je bilo zdaj telo drugačno;

Spominski dražljaj: v obliki *7* je pogledala samo prve tri dražljaje (četrtega zavestno izpušča) in jih je spet verbalizirala, ampak ne v svoji spakedravščini. Slišala je neko intonacijo, neko zvezno melodijo in ti toni so ji nakazovali vsebino objektov; slišala je posamezne tone, ki so se pa zlile skupaj. Vsak objekt je nekako bil svoj ton; imeli so višino in barvo; toni so se pa prelivali eden v drugega; ob tem, da je slišala nežno melodijo, je doživljala tudi zeleno barvo;

Barva: poleg melodije, ki je nadomeščala govor, se ji je zarisala zelena in črta. Te črte so bile nežne, mehke in okrogle; črte so bile črne, nad njo pa je dvorazsežno videlo zeleno in rumeno barvo; to je bila neka avra. Te črte so bile kvalitativno drugačne od prejšnjih čačk. Videla jih je na belem ozadju, ki pa je bilo podobno tistemu prejšnjemu (ni videla sveta onkraj njega). Zdi se ji, da je melodijo ustvarjala, barve pa so preprosto bile tam;

Pavza: Med pavzo je nadaljnjo vlekla to melodijo in videla je črte z avro. Ta črna črta je bila bolj zvezana s toni, kakor z objekti samimi. Vse to se nahaja na tej beli ploskvi;

Če si med intervjujem udeleženka predstavlja belo ploskev s temi črtami in barvami, je ne more rotirati;

Repki so bili bolj bledi, kakor med spominskem dražljajem, vendar niso bledeli s časom. Ostajali so enako bledi;

Meta-kognicija: med pavzo ni imela meta-kognitivnega spremljanja;

Testni dražljaj: slišala je melodijo, spet je lahko vzpostavila povezavo med njo in objekti, barva in črta so bile sicer še vedno prisotne, vendar manj intenzivne;

Enakost: preverjala je repke, končno odločitev pa ji je podal *gut feeling,* ki se ji je kazal telesno, kot nekaj prijetnega in umirjenega. To je bil zelo blag občutek;

Trenutek odgovora: ve, da je pritisnila na tipko, vendar je nima posebej ozaveščene. To je bil nek podaljšek *gut feelinga.* Spremljala jo je meta-kognitivna zavest, da je pravilno odgovorila. To je bil telesen občutek. To je bil nek rahel vznik iz trebuha;

Udeleženko sem vprašal, če ima sinestezijo in misli da ne.

128 S19-03-C-01

Spominski dražljaj: njen pogled je bil razširjen na celotno sliko.

Pavza: fiksira oči na eno točko, običajno na zgornji desni krog in poskuša zadržati to sliko v mislih. Ob tem barve bledijo in ozaveščen ima fiksacijski križ;

Testni dražljaj: primerja celotno sliko, ki jo drži v mislih s tem na ekranu in išče skladanje.,

Med spominskim dražljajem in pavzo ustvarja v glavi nek takt: »ta, ta, ta, tam.« Med pavzo ji pomaga vzdržati sliko.

Med pavzo vidi kroge, ki izginjajo in migetajo.

Ko si med pavzo poskuša vtisnit dražljaj v spomin: njena pozornost je usmerjena in ostra, v glavi ima občutek ostrine, zapomniti pa si jo poskuša čisto vizualna. Poskuša nadaljevati videnje slike v pavzo. Vidi bel prostor, na katerem so te barve in onkraj tega prostora vidi križec.

Doživljanje križca: vidi ga precej jasno

Razširjen pogled: pozornosti nima na eni točki, ampak na širšem območju;

Fiksacija pogleda med pavzo: avtomatično dejanje, ki ga je ozavestila;

Zadrževanje podobe: medtem ko fiskira pogled zgoraj desno, vizualno vidi sliko, ki migeta. Barve niso več v krogih. Sicer ve, da so krogi, vendar ima ozaveščene le tiste polovice, ki so bližje križu; krogi ne izginejo dokončno, ampak zbledijo v smer stran od križa;

Takt v glavi: ustvarja ga ona, nima melodije, nima tona. Ob tem vidi sivo barvo, ki ima mehko obliko. Grafitno sivo. To vidi pod krogi. Med krogi in barvami je nek prosojen *flow* v obliki *7.*

Primerjava: je vizualno doživljanje. Pozornost spet razširi na celotno sliko, s tem da desni krog doživlja malo bolj intenzivno; ritma med testnim dražljajem ni več. Primerja le vizualno. O enakosti se odloči na podlagi *gut feelinga.* Ta pozornost je ista kot med spominskim dražljajem, le da je temu dodan ta element primerjave. Vizualno pogleda celotno sliko in ne odloči se racionalno. Prej gre za to, da ji nekaj da vedet, ali je enako, ali ni. Ni nobenega vznikanja. (Mogoče) občuti nek telesni občutek nagibanja, ki ji daje vedet, ali testni dražljaj ustreza.

Trenutek odgovora: to je nadaljevanje tega *gut feelinga,* češ da je delo narejeno, samo še pritisniti mora; po odgovoru vizualna podoba ni popolnoma odpadla stran;

129 S19-03-C-02

Spominski dražljaj: s pogledom zaobjame celotno sliko, znotraj tega pa naredi to črko *7.* ni bilo prisotnega nekega ritma. Velik poudarek je bil na gesti refleksije, zaradi česar si je vse skupaj slabše zapomnila.

Pavza: pozornost na gornjem desnem krogu; vse skupaj je bledelo, gornji desni pa je dlje časa ostal intenziven. Ti krogi so migetali in aktivno bledeli ter celo zbledeli do testnega dražljaja.

Testni dražljaj: pozornost ponovno razširi na celotno sliko;

Gesta refleksije: doživljala jo je moteče. Kot da je stopila ven iz odnosa med računalnikom in njo. Bilo je kakor da je bila zraven ena Petra, ki je opazovala doživljanje druge Petre. Ne izraža se metaforično, ampak se res počuti razcepljeno. Del, ki je opazoval, se je kazal v neki čisti ostri pozornosti, ki je bil usmerjen med računalnik in njo. Obenem pa je bila pozornost druge nje na njej sami. Lahko je prehajala med tema pozornostma, vendar je bilo vedno prisotno oboje. Manj pozornosti je bilo na reševanju naloge in gesta refleksije je prevzela večino doživljanja. Ni se mogla osredotočit samo na nalogo in odrinit sam opazovanje;

Poskušanje zmanjšanja opazovanja: poskušala je povečati fokus na ekran. Poskušala se je povezat z ekranom. Ta fokus je bil še vedno relativno nenaporen. Večji napor je bil zmanjšati fokus, ki je bil na doživljanju. Ta napor se ji je kazal kot neka neprijetnost;

Dražljaj si je zapomnila slabše, to pa je vedela tako, da je bil ta akt primerjave (ta občutek) precej bolj nejasen. Slika spominskega dražljaja je bila razsuta; pol jo je manjkalo. Od nje so ostali le fragmenti. Ta nejasnost jo je spremljala čez celo nalogo. V tem ni bilo nobene dodatne razsežnosti, le njeno osnovno doživljanje je bilo okrnjeno;

Pritisk tipke: doživljala je nelagodje ob neprepričanosti in v resnici je pri odgovoru ugibala. Ni bilo *gut feelinga.* Nelagodje se ji je kazalo kot neka praznina.

Ugibanje: bilo je kot nek nežen telesni metronom med levo in desno tipko; to nihanje je bilo kvalitativno drugačno nihanju v prejšnjem primeru, ker je bilo več praznine (manj je bilo opore, ni bilo nekega *feelinga,* na katerega bi se lahko zanašala).

130 S19-04-O-01

Poskušala si je zapomnit od leve proti desni samo prve tri; v črki 7;

Medtem si je govorila neko spakedravščino; ni imelo neke forme, ampak je bila neka gmota glasov, ki naj bi imele svoj pomen; je bila neka opora na katero se je naslonila, da si je lažje zapomni. Spakedravščino so spremljale neke vizualne podobe. Videla je belina, nad njo pa trorazsežne širše črte. Bile so rahlo bež barve na robovih in je bilo kakor da so se dvignile iz površja. Vsebuje možnost da se spremeni in ni se premikalo (potencial premikanja). To se je nahajalo nekje v miselnem prostoru;

Pavza: videla je vizualne podobe, skupaj s spakedravščino; ponavljala si je, poskušala pa je zadržati sliko prvih treh objektov → videla je repke z delom kroga pri repku; ostalo je bilo zabrisano. Pri četrtem je bila nakazana črnina, ki je dajala vedet, da tam nekaj je; to je bilo vizualno izkustvo; to je ona ustvarjala; niso bledele; malenkost naporno jih je bilo ustvarjat;

Testni dražljaj: ponovno je s pogledom šla v obliki 7, ampak zelo nahitro. Osredotočala se je najbolj na gornje dva. Po nekem občutku se je odločila ali je ali ni;

Aktivno je primerjala s sliko, ki jo je držala v mislih; predvsem gornje dva. V tem ni bilo prepričanja, ali je prav ali ni;

Pritisk tipke: bilo je po nekem občutku; kakor da se je naslonila na neko notranje vedenje, ki ni razumno (vedenja ni bilo, bila je bolj neka slutnja);

Meta-kognicija:: večinoma je imela občutek, da so njeni odgovori pravilni; bilo je kot neko nihalo, ki gre levo ali desno, da zanihaš na eno ali drugo stran. Nikoli ni bilo dokončno tam, ampak se je le nagnila tja. (mogoče) je čutila to nekje v prsnem košu;

Zahteve naloge: na začetku je bila rahlo prisotna tesnoba. To ni bil ravno strah. Bala se je, da ne bo mogla izklopit vsega. Strah se ji je kazal tako, da so se ji ojačali zvoki iz okolice. Ker je vedela, da jih mora utišat,, jih je bolj zaznala. Vendar je to že po prvem primeru stopilo v ozadje. Ta tesnoba, strah in zvoki so izginili.

Zahteve naloge: bilo je le vedenje, da mora povečati pozornost. Ni bila prisotna neka odklonilnost do naloge, ali ukaza samega.

131 S19-04-O-02

Spet je z očmi šla v obliki črke 7; govorila si je, kam grejo črtice. Tokrat ne v spakedranščini, ampak v jasno izraženi: »dol, tja, tja.« s tem da je zadržala sliko prvih treh; zadnja pa je bila le nakazana kot neka črna gmota;

Pavza: zadržala je to sliko;

Slika se ji je zdela bolj jasna, tudi besede so bile bolj izrazite; → bolj so obstale, manj jih je lovila; manj truda je vložila v to, da so se obdržale. Kar pustila jih je, da so bile tam;

Fokus: ni ne lahkoten niti truden; je neobremenjujoče poglobljena;

Zdaj pri besedah ni bilo ničesar vizualnega;

Testni dražljaj: ponovila si je »dol, tja, tja« z besedami; spet je šla v obliki črke 7 čez objekte, predvsem čez gornje dva.

Pritisk tipke: prisotna je bila večja mirnost, z malo večjo prepričanostjo v primerjavi s prejšnjim primerom; → ni bilo nekih motečih faktorjev iz okolja, ni bilo nekega strahu ali tesnobe; v prsih je čutila neko mirnost (to je bil nek telesen občutek);

Ni bilo strahu;

Mar ji je bilo, da pravilno odgovarja, vendar je ni bilo tako strah; želela si je pravilno odgovorit, ampak je bila v tej svoji želji bolj mirna v primerjavi s prvim blokom;

132 S19-04-P-01

S pogledom je sledila pikam in slišala je nem zvok, ki ga sicer ni bilo;

Občutila je ritem; to je bila slušna izkušnja, ki jo je čutila v telesu;

Slišala je pike;

Zvok je bil lahkoten, nežen, top in brez dejanske akustike;

Pavza: ni počela nič, le čakala je na tesni dražljaj. Ni zadrževala slike. Samo čakala je na to, kaj bo. Zrla je v ekran, splošno v sredino, kjer so se pojavljale. V fiksacijski križ.

Testni dražljaj: s pogledom je sledila pikam; če so zarisale isto shemo, kakor prej, če so šle v isti linije (bila je povezana linija, vendar to ni bila vizualna izkušnja. Bila je nevidna; kot neka pot → prisotna je bila že med spominskim dražljajem: med spominskim se je zarisala in v njej je bilo prisotno večje pričakovanje; ostala je med pavzo), je spustila pozornost. Ni več sledila, ker je vedela, da ni prav.

Spustiti pot: to se ji je zgodilo;

Trenutek odgovora: rahel občutek zadovoljstva; telesen, občuten v prsnem košu; top občutek, nežen.

Meta-kognicija: bil je prisoten nek občutek, da je uspešno reševala nalogo; to je neko vedenje, miselno, da je prav; spremljala ga je neka mirnost, neko prepričanje, dober občutek v telesu. To je prej odsotnost dvoma, kot neka dodatna stvar. Mogoče je bilo malo dodanega prijetnega občutka, neke pozitive.

Zahteve naloge: ni bilo prisotno;

Bilo ji je pomembno, da pravilno rešuje, vendar ne zelo. Ne bi dojemala kot nekaj hudega, če ne bi pravilno reševala. Naloga ji je bila rahlo igriva in prisoten je bil nek užitek → ritem in pot sta bila lahkotna kot, da ji ni bilo težko; ni bilo tesnobe, strahu. Bolj z zanimanjem je spremljala, kaj bo. Ni bilo pritiska v pozornosti, da se mogoče ne bo spomnila.

133 S19-04-P-02

S pogledom je sledila objektom;

Z njenim glasom jim je dajala »tam, tam, tam, tam.« pojavljala se je intonacija; ona je voljno to počela;

Pavza: ponavljala je vzorec, po katerem so šle pike. To ni bila povezana linija, ampak so bile le točke. Te točke so bile vizualna izkušnja. Bile so bele. Ona jih je ustvarjala. To je bilo lahkotno;

Pavza: imela je čas, da je razmišljala o čem drugem;

Testnim dražljaj: čakala je, če se bo prva pika pojavila pri spominskem. Sledila je, če gre po istem vzorcu; to pričakovanje ni bilo prisotno med pavzo; ampak je vzniknilo, ko se pojavi fiksacijski križ → ki je tam ves čas, torej ga udeleženka ni registrirala;

Zraven je slišala svoj glas »tam, tam, tam, tam.«

Trenutek odgovora: prisotno je bilo močno prepričanje v pravilnost odgovora, to je bilo razumsko vedenje, da je prav + mirnost v telesu, kakor da se ti nekaj usede;

134 S22-01-O-01

Nekaj, kar se dogaja je, da zelo čuti nogo in napetost v telesu; čutila je neko napetost, ki je ne bi bolje znala opisati, kakor da se nahaja v nogi in da se razrašča; v prsnem košu se je čutila zelo odprto, zračno;

Osredotočala se je na zaslon, na kar jo je pozval fiksacijski križ, ko se je pojavil. Imela je odprte oči in gledala je na zaslon;

Poskušala je ohranit ta občutek, s katerim bi si potem lahko pomagala vedet, ali je ta dražljaj testni, ali ne. Ta občutek pa ni bil neka detajlna slika. Ni si zamislila objektov do potankosti, ampak je skušala zadržat ta občutek. Bil je vizualen, prostorski, ampak ne tako artikuliran vizualno, niti prostorsko; skušala se je zavedat, ali je nekaj diagonalno. Tega ni verbalizirala. Iskala je te osnovne dimenzije;

Par primerov prej jo je začelo frustrirat, kako je zadržala v nekem spominu to, kar je videla od spominskega do testnega dražljaja;

Artikulirala se ji je misel, kako *annoying* je to, da si lahko zapomni le to, ali je diagonalno, ne pa tudi na kateri strani repek izhaja iz paglavca; obenem je vedela, da hoče pokomentirat to in mi poročati o tem;

Pri odgovarjanju si je skušala zapomnit te stvari, hkrati pa ni imela nobenega zaupanja v to; zavedala se je, da je bila v tem občutku, ki si ga je poskušala zapomnit, bila prisotna neka splošna nesigurnost; ne da je bila nesigurna, ker ni bilo časa za to, ampak bilo se je vdala v to, da sploh nima pojma, kako si zapomni;

Vedela je, da si lahko zapomni eno diagonalo, ne pa kje ima glavo;

Trenutek odgovora: preprosto pritiskala je;

V njenih odgovorih je bila vdanost v to, da itak ne more;

Vse je šlo močno hitreje, kakor je mislila, da bo;

Pavza: ne spomni se, ali bi med pavzo lahko izluščila posamezni objekt iz tega občutka, ker so se ji primeri pomešali med sabo; težko ji je odgovorit na vprašanje za ta specifični primer;

Če bi jo ustavil med pavzo in če bi si lahko vzela čas, bi lahko izluščila vsaj nekaj objektov; lažje bi jih rekonstruirala;

Osredotočenost na ekran:

Imela je neko zavedanje, da je *too much,* in ni bila osredotočena na nalogo, ampak vdana v dejstvo, da jo mora reševat. Način gledanja na ekran je bil tak, da je vedela, da ni šans, da si zapomni vsako stvar posebej, zato si je zapomnila nek *gist* nečesa; to pa ji je že med samim opravljanjem naloge bilo zelo skrivnostno:

Čutila je na pol vdanost, na pol paniko; vdana je bila v panično stanje in stanje potlej ni bilo panično; prim. z občutkom, ko je prvem letniku reševala Gregino nalogo (nek laboratorij, strah, šopanje dražljajev, ki jih je popolnoma preveč); → to ni občutek, ki bi ji sicer bil znan iz življenja;

Vedenje, da opravlja neko nalogo: med samim reševanjem naloge je bilo ogromno stvari, o katerih bi želela komentirat, oziroma o njih razmišljat. Tako ni bilo časa sledit katerikoli misli. Vedenje, da opravlja nalogo, je povezano s tem kontekstom;

Občutek totalnega pomanjkanja nadzora: ve, da ne more odgovorit na vprašanja, ampak mora odgovarjat: naključno je klikala; ob tej vdanosti ni nobene misli, vendar pa prevzame večji del njenega doživljanaj;

Pri prvem trialu se ni mogla spomnit, ali mora pritisniti levo, ali desno; ob testnem dražljaju ni mogla vedet, a je isti, a ni isti, niti, kaj mora pritisniti. Tako je pritisniti, kar nekaj naključno; po tem ji je postalo jasno, da je bil verjetno isti, ampak je pritisnila drugo tipko (kljub temu da je hotela pritisniti tipko za istost);

Tekom naloge se ji je začel razvijati nekaj misel, če ima občutek, da ugiba; začela je dobivat to neko vprašanje, če se bo izkazalo, da sledenje nekemu temu medlemu feelingu, da zazna neko razliko;

Misel, da mi je hotela povedat, da je ful jebeno, da se orientacija spreminja tudi glede na to, kjer je glava;

Pavza: poskušala si je zapomnit nek kvadrat, nek lik, ki ga te črte zarišejo; ni si poskušala zapomnit vsakega posebej, ampak jih je grupirala v neke like. To je bil *gist,* s katerim si je pomagala; to je bil ta v paniko vdan *feeling;*

To da je povezala dražljaje, se ji je dogajalo; prvi primer jo je res šokiral; ni imela neke strategije, preprosto sledila je nekemu temu osuplemu občutku; vtis ni bil iz nje ven na dražljaj, ampak je je bila ta stvar tam zunaj in ona je bila dosti pasivna; ni poskušala nečesa zadržat; zadeva se je samo vtisnila vanjo;

Občutek strmenja; imela je občutek, da kar dela, dela s čelnim delom glave in da se širi ven;

Sproti je nekaj na glas govorila;

Vedenje, da si ne more zapomnit;

Splošna nesigurnost: samo njeno doživljanje ni bilo nesigurno, ravno zaradi tega, ker se je vdala tej nalogi; sprva je to bilo prisotno, potem pa je nekako sprejela ta *ne morem* in je samo začela pritiskat stvari;

Kvadrat: meta-misli o tem, kaj naj dela; ali naj sledi temu *feelingu,* ali je to totalno zgrešeno; kar je skušala zadržat je bil občutek vseh teh štirih elementov, kako so orientirani; te misli so bile

artikulirane; tisto, kar je rekla, so bile neposredno izrečene misli; ni bila predhodno artikulirana misel, ki bi jo potem oblikovala z besedami, ampak je neposredno izrekla; veliko teh komentarjev je bilo namenjenih meni, ne toliko njej sami; pri prvih nekaterih se je spraševala, ali si ljudje res zapomnijo to; prisotno je neko spraševanje o tem, ali je normalno, da si ne more zapomniti dražljajev; te misli sicer niso bile tako formirane, ampak je bilo le zavedanje, da ne hendla tega; pod tem pa je bilo neko vprašanje, ali se bo to naučila hendlat tekom naloge in ali folk hedla;

Nesigurnost = sprejemanje, da ne more;

Pri nobene odgovoru ni imela občutka, da je isto ali drugačno;

Na koncu se je ustalila na tem, da si bo zapomnila ali dva po dva ustvarita del nekega osnovnega lika;

Panično je reflektirala o sami nalogi: ali morda ostajajo dražljaji isti in se sploh ne spreminjajo + želja, da si želi te stvari razjasnit z mano;

Vseeno pa je vedela, da bo 100 ponovitev in bo na neki točki razvila strategijo;

Ni pa bilo blaznega poskušanja pri strategijah, samo delala je stvari;

Neko spoznanje, da spet opravlja nek psihološki eksperiment; znano ji je bilo, kako se dela te stvari; občutek vdaje in sprememba odnosa pa je bil pospremljen s tem zavedanjem in prepoznavo, da je to take vrste eksperiment; najprej je poskušala nekaj zagrabit, pri ostalih pa je pustila, da se stvari dogajajo;

Sprotne misli: ona jih ustvarja; za razliko od reševanja naloge je pri tem veliko več občutka neke aktivnosti;

Na glas je izrekla neke besede, preklinjanje: to je bilo ločeno od samega reševanja naloge, ampak je bilo nekako pomirjujoče, ko je izrekla to;

Njene misli in komentarji so bile bolj namenjene meni, ki jih ne bi bilo, če me ne bi bilo tam; mene se je aktivno zavedala do te mere, da je vedela, da sem tam;

Ko je to rekla, je to ustvarilo neko ločitev med tem, kar dela in kar se ji dogaja;

135 S22-01-O-02

Niti si ne more zapomnit, kakšna je bila orientacija; ne mre izluščit zadnjega primera;

136 S22-01-O-03

Za zadnji primer ima občutek, da je odgovorila narobe; dražljaja sta bila različna, odgovorila je pa, da je isto, čemur je sledil takojšen občutek, ne da ni bilo isto, ampak, da ne ve, če je bilo isto;

Ta občutek je bil vezan na zgornji desni kot;

Poskušanje, da si zapomni: tako je *too much* vsega, da je razmišljala, kako bi lahko razvila nekaj, kar ne bi imelo *feelinga,* da totalno, ne ve; ni imela časa razviti nekaj, da bi se odločila, kaj bo gledala: vsega je bilo samo preveč;

Na pol odločeno se je poskušala zapomniti dve po dve (ali vodoravno, ali diagonalno);

Znotraj tega strmenja, ki je poskušalo zaobjet štiri, je malo bolj imela občutek za dve in dve;

Te dve in dve bi morala še vedno zložit skupaj in to ji še vedno ni uspelo;

Pri zadnjem je skušala (pasivno) zapomniti dve po dve, s tem da so se te podobe same pogrupirala;

Feeling, da v resnici ne ve, je bil vezan na desni gornji kot;

Imela je občutek, da je bila desna orientirana diagonalno glede na levo, ampak v drugo smer;

Občutek, da ne ve, je zelo izviral iz ekrana, kar velja celostno za doživljanje te naloge;

Opazila je, da sta dva zaporedna primera bila ista, ob čemer je imela neko prepoznavo, da je to isto, kar se je kazalo kot medel občutek; pri nobene svojem odgovoru nima nekega občutka, da je tko. Ko je poskušala med tem najti nek ta občutek ja ali ne, *tako da se je osredotočila na posamezen objekt, vendar si ni upala spustit celote. Občutek je bil, da če se bo osredotočila na posamezno stvar, bo izgubila preostale. Nikdar med reševanjem te naloge, ni imela občutka istosti. Tudi, ko je opazial, da sta primera ista, ni bilo občutka istosti, samo opazila je to, ob čemer se je vprašala, kako lahko vem, da sta ista, če nimam občutka, da sta ista; ta prepoznava ni prišla iz nje, ampak je bilo kot da je bil na zaslonu isti print; kot da bi od prejšnjega primera ostalo nekaj, na kar bi se drugi preprosto prilepil; spraševala se je a) kako da zdaj ve, da sta ista; b) ker je bil trikrat isti dražljaj, c) če se trikrat ponovi je drugače, kot če se dvakrat, d) ali bom zdaj vedela, kaj bo → ko je prišel testni, ni bilo občutka prepoznave, da je isti ali ne. Odgovorila je, da je isti; in e) ali so mogoče vsi triali isti. Te misli so bile zaporedne in neartikulirano; to zavedanje je bilo zelo, zelo na zaslonu;*

Ves čas med nalogo ima *background* zavedanje nevednosti;

Zavedanje, da je isto, je bilo na zaslonu, hkrati pa je bilo povezano z *backrgound* vdanostjo;

Tej prepoznavi, oziroma temu sumu, da je isto, je sledilo presenečenje ob tem, da prepozna, da je isto, kljub temu da prej ni prepoznala;

Zavedala se je, da ne ve in je rekla »ja,« ker ni bilo nekega znaka, da ne;

Zavedala se je, da je dvakrat zaporedno odgovorila »da.« imela je neko zavedanje sivih tipk in nekega dvakratnega pritiska; sive tipke, ki so imele črne tanke znake, ki so pa druge, kakor jih v trenutku intervjuja vidi na ekranu; zavedanje da je dvakrat rekla »da« je bilo na tej tipki, ne pa v njenem prstu;

Grupiranje dva po dva: dvojico je ona prisila skupaj; več je bilo iskanja in poskušanja. To je bil manj vtis in bolj to, da je nekaj delala; skušala je nekaj naredit iz teh štirih stvari;

Zavedala se je, da njena investiranost … telesno se je nagnila v ekran; kar se je dogajalo se je čutilo zelo aktivno, kar se ni skladalo z njenim občutkom, ki je v resnici dosti vezan na to, da dela to in da mora za to rekrutirati neko enegrijo;

Obenem je slišala zvok motorke zunaj: nenadoma se je zavedala tišine, ki se jo je zavedala tišine, ob čemer je njeno doživljanje postalo veliko bolj sveže. Obenem je čutila nek strah, da se bo hrup vrnil;

Tišina je bila nek občutek zračnosti in črne barve;

Celoto je očitno držala, če si je ne upa spustit; ima občutek držanja in sploh ni občutka, da ji polzijo, ker v danem trenutku ni prepričana v nič;

Imela je strategijo, ki je ni zavestno razvila, da je hotela najti doživljajski korelat istosti; hotela je dobiti *feeling,* da najde napako;

Dalj časa, ko je to trajalo, bolj je razvila občutek, kaj dela. Pri tem je nekako bolj videla, kaj je levo spodaj in desno zgoraj.

Vendar pa tudi če je imela občutek spominskega dražljaja in bi ga med pavzo lahko rekonstruirala, ko je prišel testni dražljaj, je izgubila *feeling* od prej;

Njen poskus prepoznat sploh ni bil vezan na to, da lahko *overlapa* testni dražljaj na kar je ostalo od spominskega;

Un *feeling,* ko ga je imela, da sta si bila primera ista, tudi za spominskega drugega, ni vedela, da je enak kot testni prvega, ampak spominski prvega;

Pričakovanje: poskuša si zapomnit prejšnjo četverico in ima občutek, da lahko to stori (vsaj v par primerih), pričakovanje pa je vezano, da se ji bo nekaj bliskalo v glavo. S pričakovanjem se ni zares ukvarjala, saj je bila osupla nad tem, da je bilo preveč vsega;

Pričakovanje je bilo precej nespecifično. Vedela je, da ji bo nekaj zabliskalo;

137 S24-01-P-01

Med spominskim dražljajem je sledil pikam z glavo;

Zapomnil si je kam padeta prva in zadnja pika, drugo si je zapomnil približno;

Recimo, če so se pojavljale v krožnici, si je zapomnil in po gibu, obenem si ga je pa predstavljal;

Med pavzo bi lahko narisal, kje so te pike;

Zavedanje okolice: je oddaljena, ampak se je zaveda. Fokus je pa ne piki. Njegova pozornost je razširjen krog, kjer se lahko pojavljajo pike;

Med pavzo si je pike predstavljal na ekranu; črne kakor ko jih je dejansko videl;

Testni dražljaj: primerjal je le prvo in zadnjo;

Napačni odgovori so mu bili bolj prijetni, ker je zagotovo vedel, da ni pravilno. Hitreje je ugotovil.

138 S24-01-P-02

Lažje;

Stoodstotno gotov je, da je vse rešil pravilno;

Manj je videl okolico;

Fokus je bil popolnoma na pikah;

Dajal si je ritem, ki je bil enakomeren in brez intonacije;

Pavza: samo čakal je, kam bodo padle nove pike in ni imel nobenih misli. Pavza se mu je zdela kratka (vse skupaj se dogaja kar hitro);

Med pavzo si je na hitro ponovil tako, da si si jih je predstavljal;

Zdaj je bil že bolj prepričan v svoje odgovore: bolj fino se mu je zdelo, ker si je zapomnil vse štiri odgovore;

Reševanje naloge še vedno ni gladko, še vedno je prisoten dvom.

139 S24-01-P-03

Med pavzo si je ponavljal pike, videl jih je v vrstnem redu. Ponovil je celo kombinacijo. Te slike so bile manj črne, niso več zares barvne pike, ampak njihova pozicija;

Reševanje naloge mu je prijetno;

Meta-kognicija: v ozadju glave čuti prijetne občutke, da rešuje prav;

Ugotovil je, da je pogosto isti nabor pik, samo da je ena zrcalna;

Občasno si je vzklikal »gor, dol, levo, na križku, daleč stran, …«

140 S24-01-C-01

Bolj intenzivna naloga, ker nima ritma, ampak mora fokus dati na štiri naenkrat. Ne more premikati z glavo. Z očmi mora sliko videti hkrati.

S pogledom se je premikal od kroga do kroga ob tem pa je verbaliziral imena barv (počutil se je kot avtor besed, skoraj jih je izgovoril naglas);

Zdelo se mu je, da je manj pavze. Vmes se je osredotočal na to, katera tipka je pravilna in se fokusiral na ekran;

Moral je biti bolj pozoren;

Testni dražljaj: primerjal je z verbalizacijami;

Okolice se ni mogel zavedati;

Meta-kognicija: spet meni, da mu gre dobro. Ta občutek je kategorično podoben tistim mislim, ki so neverbalizirane in jih doživlja zadaj v glavi;

Tekmuje sam s sabo ali pa z računalnikom;

141 S24-01-C-02

Meni, da v reševanju naloge ne napreduje, da je vedno ista težavnost, oziroma da jo rešuje vedno težje;

Pride do tretje barve, zadnje pa se le bežno zaveda;

Pogleda jih kakor se jih bere in jih verbalizira;

Pavze so tako kratke, da se uspe le pripraviti na naslednji dražljaj, če ima čas ponovi barve z verbalizacijami;

Testni dražljaj: barve si ponovi;

Četrto barvo med spominskim dražljajem vidi malce zamegljeno, med testnim dražljajem pa odgovori, da je ta enak, tudi če vidi samo prve tri;

Zaradi tega četrtega dražljaja doživlja nekaj dvoma, kar doživlja tako, da bi rad videl rezultate. Pričakuje, da jih bo videl;

142 S24-01-C-03

Udeleženec si je med reševanjem nekaj šepetal;

Ko je videl, da mu uhajajo objekti, si je poskusil zapomniti lik v barvah; Pogledal je celega;

Pavzo: si je ponovil z besedami te barve, izluščil jih je s celote;

Udeleženec vztraja, da je pri tej nalogi manj časa;

Testni dražljaj: ni pogledal celotnega lika, ampak je po vrsti verbaliziral barve;

Meni, da rešuje dovolj pravilno, ampak mu je naloga zelo hitra in ne ve, ali si zapomni pravilno, ali ne;

143 S24-02-O-01

Pogleda celota in si reče »gor, gor, dol, desno.«

Je njegov glas, tišji;

Ne more si zapomnit samo vizualno;

Konkretno ustvarja besede;

Meta-kognicija: meni, da kar dobro rešuje nalogo;

Med pavzo ne počne ničesar, ker je premalo časa. Osredotočen je na to, kaj se bo zgodilo po tem:

Primerjava: malo je vizualna, malo si pa ponovi besede; te besede se zgodijo same od sebe;

144 S24-02-O-02:

Zapomnil se je, kako so padali repki;

Spet si je govoril;

Med pavzo ponovno nima časa in samo pričakuje, da bo prišel naslednji dražljaj;

Naloga je težja od vseh do zdaj;

Ni več prepričan v uspešnost: to doživlja tako, da bi rad še poskušal in se še navadil;

Pogleda vse štiri like naenkrat; premalo časa je za en sam gib;

145 S24-02-O-03

Pogledal je, kako so bili usmerjeni;

Vsi repki so bili usmerjeni na dol: to si je zapomnil tako, da si je rekel »vsi so na dol,«

Med pavzo se samo koncentrira: fokus je naporen;

Primerjava: dražljaj se mu je preprosto dajal tak, da je videl, da je eden usmerjen navzgor.

Zdi se mu, da se ne izboljšuje pri reševanju naloge;

Zdi se mu da je pavza enako dolgo, spominski dražljaj pa se mu zdi krajši.

146 S24-02-O-04

»gor, gor, dol, dol.«

Fokus je na vseh štirih naenkrat, ob tem si je pa hitro ponovil besede zanje;

Med pavzo je bila samo koncentracija, ni bilo nobenih misli;

Sobe ni slišal;

Zaznaval je samo nalogo;

Prepoznava je bil vizualna, tudi med drugim si je zraven govoril, pri čemer si je ponavljal usmeritve objektov.

147 S24-02-C-01

Zelena, rdeča, črna, rumena

Poskušal je ven potegnit: potegne jih nekje iz zadnjega dela glave; to je bilo lahkotno;

Poskuša se fokusirat samo na določen lik: zapomni si ga in pozabi v trenutku, ko odgovori na nalogo, ob čemer njegovo doživljanje prevzame pričakovanje na naslednjega. To pričakovanje je isto, kot med pavzo;

Pogleda cel dražljaj;

Verbalizira si imena za barv, to ponovi še enkrat, med testnim;

Meta-kognicija: zdi se mu, da uspešno rešuje nalogo;

Verbalizacije so v njegovem glasu, s svojim glasom;

148 S24-02-C-02

Vizualno si je zapomnil vse štiri;

Pogledal je cel kvadrat;

Zraven ni bilo verbalizacij, samo gleda;

Med pavzo jih poskuša ponovit; med testnim pa še enkrat poskuša v glavi ponoviti barve; ponovi jih prek verbalizacij;

Meta-kognicija: občutek, da dobro rešuje nalogo. Občutek je prijeten, konkretno si reče »ta je pa pravi.« ta glas je kvalitativno enak glasu, ko si verbalizira barve.

149 S24-02-C-03

Pomislil je, da je verjetno zadnji in si ga moram zapomnit; ta misel je prišla sama od sebe;

Videl je vse štiri, obenem pa si jih je ponovil;

Gledal je bolj šablonsko: premalo je bilo časa, da bi si vse ponovil in v resnici si je ponavljal med pavzo in testnim dražljajem;

Meta-kognicija: občutek, da dobro rešuje in ponovno si je to rekel: »kar dobro sem rešil; sem se že malo navadil:« bolj je podobna misli, da je to zadnji primer, ker verbaliacije barv so postale zelo avtomatizirane.

150 S24-03-P-01

Vsaki posebej sledi z očmi;

Naloga je ravno toliko počasnejša, da ji lahko sledi. Lažje ugotovi, kje so; ni mu treba videti vsega naenkrat;

Smer si ponavlja: »levo, desno, gor, dol, zraven, zraven, stran, stran.« med spominskim dražljajem in še enkrat si ponovi med pavzo;

Ko ponovi, le skoncentrirano čaka;

Čaka, da križ postane črn (pred spominskim dražljajem), ker ve, da bo takrat spet začelo padat;

Naloga mu ni naporna;

Meta-kognicija: to ni dodaten občutek, ampak ve, da če bo zbran, da jo bo rešil pravilno;

Zbranost je lahkotna;

Meni, da se sobe ne zaveda, razen mogoče v ozadju ve, da sem tukaj jaz in soba;

Verbalizacije: zavestno ustvarja, se počuti, da je on avtor teh besed.

151 S24-03-P-02

Vzorce si zapomni tudi onkraj reševanja;

Verbalizirano si je ponavljal + celotnega je videl;

Vidno: ostane neka sled; vse skupaj je bledo; projicira iz misli na ekran, ima občutek, da nekaj aktivno počne; to projiciranje terja malce več pozornosti;

Projekcije nastajajo ena po ena;

Med testnim dražljajem se ta smer ponovi in če ne, potem je odgovor negativen;

Ugotovitev enakosti: pogleda ali se pojavitve objektov ujemajo s projekcijami + ponavlja si z glasom;

Testni dražljaj: včasih ve, da je nepravilno, še preden ga vidi do konca; → išče napako;

Celotna naloga je lahkotna, pozitiven občutek, ki ni nek ekstra občutek, ampak je preprosto lahkotno opravljanje naloge.

152 S24-03-P-03

Zapomni si »graf,« to so projekcije; → osredotočal se je na križ in potem je pozornost preusmerjal stran od njega proti objektom;

Ko se je enkrat spominski dražljaj končal, je ta graf ohranjal v glavi;

Z besedami si je zapomnil položaj: »gor od pike, dol od pike …«

Graf: nahajal se je na ekranu: projekcija je bila meglena, ni imel barve, v megli je videl potek tega vzorca; zaznaval je neko smer;

Pavza: le čakal je, gledal je v križ; ni imel časa za razmišljanje;

Testni dražljaj: pozoren je bil, če bo kak objekt padel v ta graf;

Ko vidi, da je ena padla ven iz grafa, ve, da je odgovor negativen in takoj pritisne na tipko;

Meta-kognicija: še vedno ima občutek, da dobro rešuje;

Projekcije on aktivno počne in se mu ne dogajajo;

Zdaj ko ustvarja projekcije, je vse skupaj bolj naporno.

153 S24-03-O-01

Vzorca sta bila oba »dol, dol, gor, gor.« vendar si bolj kot verbalizacija poskuša zapomni cel graf;

Meni, da je slabši → če ni videl vseh je vedel, da ali bo blefiral, ali bo odgovoril, da je narobe; to si je rekel v glavi, ko je odgovarjal na nalogo;

Spodnja dva vidi v megli: ne more videti vseh štirih hkrati; pogled mora malo osredotočiti na dvojico. Če mu uspe pogledati obe dvojici, potem si zapomni;

Obenem si malo govori z besedami;

Če si ponavlja besede za vse štiri objekte, mu zmanjka časa;

Premalo časa je, da bi ustvaril neko sled; zapomnil si samo nek celosten vtis;

Med pavzo ga ne bi mogel rekonstruirati; šele ko ga vidi med testnim dražljajem, razbere, ali ustreza;

Spominski dražljaj: objekti se mu že dajejo grupirani v pare; vedno vidi skupaj zgornje dva in spodnje dva;

Če je hotel videti cel spominski dražljaj se je moral zavestno premakniti iz zgornjega para na spodnjega in ta premik je bil naporen;

Verbalizacije so bile njegove, ampak ni bilo časa, da bi si jih vse ponovil;

Pavza: ni bilo dogajanja, le koncentracija. samo pričakoval je, da se bo nekaj zgodilo; ni bilo nekih dodatnih misli, le uperjenost naprej;

Ugotovitev enakosti: glede na vtis + besede; istoćasno, ko je videl testni dražljaj je kdaj pa kdaj že takoj vedel, ali je vzorec enak ali ne (tak se mu je dajal), ampak si je vseeno ponovil še besede;

154 S24-03-O-02

Zgornja dva sta bila obrnjena v isto smer in spodnje dva sta bila obrnjena v isto smer;

Spremenil je taktiko, da si hitro ponovi »desno, desno, gor, dol.« še vedno se mu gornje dva in spodnje dva dajeta grupirana;

Zdi se mu, da je ta taktika boljša, ker vidi vtis (ki je sestavljen iz dveh delov) nanj pa poda verbalizacije;

Boljšost taktike: fin občutek ob napredku: to je mentalen občutek ob napredku, po katerem je bolj gotov v to, da jih je več rešil pravilno;

Pavza: koncentracija, ob čemer si je v mislih govoril, da mora čim hitreje verbalizirati usmeritve;

Testni dražljaj: vtis + ponavljanje; istost je zaznal še preden si je zverbaliziral besede; repki so se mu že dajali kot pravilni ali napačni;

Ni mogel videti vseh štirih naenkrat, ampak je lahko pogledal le po dva naenkrat;

Premik iz zgornjih dveh na spodnje je dva je moral biti hiter, da ni zamudil spominskega dražljaj; ta hiter premik je naredil zelo zavestno, ob čemer je premaknil cele oči.

155 S24-03-O-03

Za zadnji primer ni popolnoma prepričan;

Hotel je odgovoriti, da je pravilno, ampak je bil skeptičen za spodnje dva. Zaradi vtisa se mu je zdelo, da je drugače;

Testni dražljaj: »gor, desno, dol, dol.« »dol, dol« je bilo tako v megli med spominskim dražljajem, da je imel med testnim vtis, da je bilo malo drugače; ta drugačnost se mu je kazala vizualno. En repek je bil po njegovem obrnjen drugam. Ta drugačnost ni bila popolna.

Po besedah bi bilo enako;

Med spominskim dražljajem sta bila spodnje dva v megli; ni ju imel v fokusu; videl ju sicer je, vendar ne dovolj, da bi si lahko zapomnil repke;

Meta-kognicija: še vedno je imel občutek, da dobro rešuje nalogo, le v zadnjih dveh primerih mu je padla koncentracija.

156 S24-04-O-01

Bolj slabo je ujel zadnji primer: odgovoril je, da je pravilen, vendar ga je videl malo v senci;

V senci: delček sekunde si je preveč dal dela z gornjima dvema, tako da je spodnje dva samo pogledal, ni si ju pa zapomnil;

Poskuša jih pogledat vsakega posebej, hkrati pa se zaveda celote. Če poskuša videti samo celoto, mu ne uspe → če pogleda samo celoto, potem ni siguren (če pogleda vse štiri hkrati, med testnim dražljajem spomin zbledi in pri odgovoru blefira, če gre pa od enega do drugega, pa si spomin bolj zapomni);

Zraven si ves čas govori usmeritve: »dol, levo.« S svojim glasom. To je bežno. On je njihov avtor;

Sigurnost ni nekaj, česar se aktivno zaveda, ampak je vezano na to, ali ve odgovor pri testnem dražljaju;

Premiki oči: preveč misli na to, kam so usmerjeni repki in tako so premiki popolnoma avtomatski. Zmeraj začne na leve in gre proti desnemu, in potem spodaj levo, desno;

Koncentrira se na nalogo in kdaj pa kdaj med primeroma pomisli, če bi spremenil taktiko (»mater, kaj če bi probal spet celoto!«

Pavza: samo koncentrira se in si poskuša zapomnit; ta koncentracija je precej naporna.

Testni dražljaj: še enkrat ponovi besede in preverja, če podobe na ekranu ustrezajo; kdaj pa kdaj pa vidi že samo po grafu (to je sploh kadar je graf precej enostaven);

Meta-kognicija: občutek, da dobro rešuje, je vezan na to, da se trudi pri reševanju. Poskuša jo rešiti čim bolje;

157 S24-04-O-02

100% je, da je pravilno rešil;

Bil je popolnoma skoncentriran in bil je tako postavljen, da si jih je lahko zapomnil;

Pogleda ga je enega za drugim, ga potrdil (lahko je pogledal vsakega posebej in ker so bili enostavni, jih je lahko pogledal vse še enkrat) in prepoznal, da je isti;

Zraven si je povedal besede za vsako usmeritev posebej;

Med pavzo se je ponovno samo koncentriral;

Testni dražljaj: ponovil si je besede in pogledal, če njihova vsebina ustreza temu, kar je na ekranu;

Trenutek odgovora: zavestno pritisne; ne zaveda se oblike prstov;

Doživljanje tega, da je to naloga: v sebi je nek pozitiven občutek, ker rešuje neko nalogo in se trudi in napreduje; ta občutek je zadovoljstvo; to je mentalen občutek in zaradi tega lažje dela nalogo (ker meni, da jo pravilno rešuje); v kolikor pa bi nalogo slabo reševal, bi bila težka;

Meta-kognicija: samo nek mali pozitivni občutek;

158 S24-04-P-01

Ker ima čas, ker so en po en, vsako piko pogleda in ustvarja graf; med pavzo ga poustvari, med testnim ga pa preveri;

Primerjava grafov: ena pika je padla izven grafa na fiksacijski križ in vedel je, da je napačna;

Spominski: pogleda vsako piko posebej in si med njimi ustvari graf; vmes med objektih si predstavlja črto; črta je zabrisana in namišljena; to ni konkretno vizualna izkušnja, ampak je le medla povezava; ta graf ni naporno ustvarjat;

Pavzo: še enkrat ustvari graf, s tem da je ob njem še enkrat premaknil oči;

Testni dražljaj: še tretjič je premaknil oči in si predstavljal ta graf;

Prepoznava istosti: aktivna primerjava, čakanje da pade iz grafa;

Meta-kognicija: pozitiven občutek + ker je vaja lažja, ni tako naporno (in vmes ima ves časa, kljub temu pa ne bi mogel zraven razmišljati, saj še vedno mora posvečati pozornost nalogi), kdaj pa kdaj si konkretno reče, da ima pravo taktiko, pozitivni občutek je mentalen, je zadovoljstvo;

159 S24-04-P-02

Spet ni ustrezal: vse pike so bile blizu skupaj in bil je lep vrstni red, v testnem pa je en objekt padel daleč stran;

Lep vrstni red: zapomnil si ga je in vsi objekti so bili okrog sredine;

Spominski: pogleda vsako posebej in si med njimi predstavlja graf;

Pavza: zraven si je govoril »zraven iksa, desno, gor, dol.« + še enkrat je naredil gib z očmi in si predstavljal graf;

Testni: čim je ena napačna, ne razmišlja več dalje in samo prekine in odgovori;

Prekine: če je pravilen graf, mora čakati do konca, v nasprotnem primeru pa ne misli več na ta poskus in se začne koncentrirati na naslednjega;

Ob pikah je spet slišal nek ritem, malo si je govoril »hmpf, hmpf.« Ampak meni, da je to posledica tega, da pojavljanje pik ustvarja nek ritem; on to počne;

160 S24-04-C-01

Na splošno gre drugače;

Meni, da si lažje zapomni barve kot črne objekte; sploh ne rabi ponavljat;

Ne gre od ene do druge in samo pogleda celoto; od leve proti desni + celota; tudi če ne pride do konca si barve vseeno zapomni;

Nima časa, da bi si dvakrat vse ponovil; → ob barvah si govori besede; tretje in četrte si ne reče zmeraj;

Med testnim si še enkrat ponovi z besedami in barve se pokažejo v možgani; dražljaj se mu daje isti ali drugačen;

Tudi če ne ponovi barve, med testnim ve, ali je isto ali drugače;

Meta-kognicija: ima občutek, da dobro rešuje. Vaja je sicer težja od pozicije. Terja veliko koncentracije zaradi časovne omejenosti, da ve, ali je prav, ali ni;

161 S24-04-C-02

Dražljaja sta bila enako in to je videl hitro;

Če so barve nežne, se mu jih je lažje zapomnit kot bolj agresivne; zadnji sklop je bil prijazen;

Pogledal je vsako posebej in si zraven govoril njihova imena; kdaj pride do dveh, kdaj do treh, kdaj tudi do štirih;

Med testnim se spet pojavijo in še enkrat poskuša ponovit; kdaj ne ve takoj, ampak ko se pojavijo se spomni, da je tako ali drugače;

Terja koncentracijo, ki sicer ni energijsko potratna, ampak se pa mentalno utrudi;

Pavza: ne počne veliko, ker ni časa; samo koncentrira se, kdaj pa kdaj preveri, ali drži na pravi tipki;

Pavza: med pavzo ne bi mogel rekonstruirati spominskega dražljaja (vsaj ne vedno);

Meta-kognicija: mentalno zadovoljstvo;

162 S25-01-O-01

Ostalo od prejšnjega primera: Bila je zelo zmedena (nemir v prstih, kot da ni čutila neke gotovosti, ali povezave med tem, da je desno prav; ne le, da ni vedela, ali je prav, ampak tudi ni vedela, kako odgovoriti). Bila je pod stresom (neko vrvenje, povišan srčni utrip, zelo telesno; zmedenost in stres sta bila intimno združena, čeravno je stres prišel prej), ker se ji je zdelo, da je prejšnjega odgovorila narobe (ni bila več osredotočena na sliko) in se ni mogla ustavit, da ne bi razmišljala o tem;

Spominski: Osredotočala se je na levo spodaj; ni se nalašč osredotočala tja; ostale je ošvrknila s pozornostjo; v resnici jim je posvečala precej malo pozornosti, vendar se jih je vizualno zavedala v relaciji z levim spodaj (če ne bi vedela, kaj je levo spodaj, mogoče ne bi imela vizualne predstave o ostalih);

Pavza: počela ni ničesar, bila je zelo mirna (prijeten občutek; ni bila ekstra prisotnost, ampak je bila samo neka obarvanost doživljanja, nek lahkoten flow). Vmes se ji je utrnila misel (misel se ji je dogajala) »o, fak, kaj če je ta občutek, da mi gre dobro napačen« (verbalizirano, glas brez zvoka, ona je avtorica teh besed in se z njimi poistoveti) v katerem je bil kanček dvoma; vendar je bila ta misle relativno mala in ni prevzela njenega doživljanja; osredotočala se je na fiksacijski križ in čakala (ona je to aktivno počela, čeravno tega ni izrecno nameravala početi).

Testni: Čutila je neko paniko ob tem, ali je bila res tako poševna, ali je bila bolj vodoravna. In imela je občutek, da na zadnjega ne bo pravilno odgovorila (bilo ji je neprijetno + ni vedela, kateri pritisk je

bil za kateri odgovor; dvom se je razširil iz misli na telo); ta dvom je bil bolj aktiven kot misel med pavzo;

Testni: ugotovitev enakosti: fokus na levo spodaj in na podlagi tega je ugotovila; ni razmišljala o levem in desnem, ni imela pojmov, ni jih ubesedila; samo vidno; ta prepoznava je bila na ekranu;

Dvom: ni bil verbaliziran, bila je neka konkretna miselna prisotnost;

Trenutek odgovora: je onkraj njenega zavedanja; ne spomni se ga;

Celostno doživljanje: na začetku ji je bilo zelo udobno, dobro se je počutila, ko se je usedla. Ni bila napeta, niti telesno niti psihično. Na koncu pa si je že zelo želela pavze. Telo se ji je zakrčilo, ni ji bilo udobno. Ni se več počutila povezano s tipkami in dražljaji, d alahko nekaj opravlja (ni imela več občutka, da so njena dejanja vzročnopolseidčno povezana z nalogo). Izgubila je zaznavanje delov telesa. Prej je bilo zelo očitno, kako ima roke, da ima prekrižane noge, potem pa je izgubila to zavedanje. Njena pozornost se je spremenila; ne bi več mogla razdelit na levo-spodaj, levo-zgoraj. Slike ni več mogla razdeliti na neke odnose.

163   S25-01-O-02

Fokus ji je pošel; ampak ne zaradi neuspeha prejšnjih, ampak ker je imela občutek, da ne razume, kdaj se ji prikazuje kaj in da ne ve točno, kaj dela;

Neprijetno ji je bilo ob tem; skorajda je čutila paniko, ki je bila hujša ob neuspehu; → malo ji je šlo na jok; občutek nemoči; čutila je napetost, ki se je dvigala navzgor iz prsnega koša; dvigalo se je med ključnici, višje pa ni prišlo;

Čutila je, da ima povišan srčni utrip;

Nemoč: ni vedela, kaj naj naredi; kot neka zmeda, tema v glavi. Ni vedela, na kaj naj se osredotoči. Ogromno je premlevala, vendar brez misli. Nič si ni govorila. Doživljanje je bilo celostno telesno. In ni mogla ustaviti tega, niti se odločiti, kaj naj naredi;

Fokus: ni bila osredotočena ne na prvo, niti na drugo sliko;

Doživljala je le paniko, vendar ni poskušala reševati naloge. nekaj je kliknila, ker je vedela, da mora → doživljala ga je kot pritisk, ki je bil tudi fizičen. Kot neka sila, da ne sme spustiti (da se ne sme ne odzvati in da ne sme samo gledat), da mora. To silo je čutila v rokah in v desni polovici prsnega. Ta zahteva je izvirala iz računalnika in se mene ni zavedala, kot faktor tega.

Predstavljala si je neko beleženje, koliko jih gre mimo, koliko jih ne poklika. To ni bilo vizualno, ampak je imela idejo. Od tod je oizhajala ta panika.

Premlevanje: tega ni nadzirala. Niti ne ve, kaj je premlevala. To se ji je dogajalo. Dogajalo se ji je v glavi.

Zdaj ne bi vedela niti, katero tipko je pritisnila, kaj šele, da bi vedela odgovor;

Na začetku se je enkrat zasmejala, ko se je prvič zmotila, ker se ni zmotila, češ da ne bi vedela, ampak je pritisnila po nesreči. To je bilo močno zavedanje, tako napaka, akkor smeh. Vendar pa to ni vplivalo na nadaljnjo reševanje, kakor dvom od prej. Se je pa zmedla kasneje.

164 S25-01-C-01

Odzvala se je zelo počasneje. Povezava med pritiskom in odgovorom (da bo poslala signal) ni bila samoumevna. Kakor da ne bi bilo njeno telo tisto, ki je pritisnilo. → bilo jo je strah, da bo tako ostalo v prihodnjih primerih. Imela je močnozavedanje tega kazalca, ker se je odgovor dogajal na desni tipki z desnim kazalcem. Zdelo se ji je, d aje njen prst ful večji; na njem je bil nek občutek težje; ta teža je ostajala po odgovoru;

Spominski dražljaj: brez posebnosti. Krajši čas se je prikazovalo. Zdi se ji, da je vse skupaj hitreje izginilo in težje se je osredotočala na levo spodaj;

Pozornost: centralna. Bolj je šlo proti levi zgoraj. Njena pozornost je bila bolj razširjena (prim. z S25-01-O-01, kjer je bila točkasta) pri preverjanju pa je gledala bolj točkasta. Pozornost se ji je zdela trudna. Ona je ustvarjala pozornost;

Pavzo: ne bi mogla rekonstruirat vseh barv; mogoče eno ali dve, ali tri;

Pavza: nič ni doživljala; neka uperjenost, pričakovanje naslednjega dražljaja. Ni se jih sploh zavedala; v prvi je imela neko pričakovanje, nek čas za zavedanje tega, kar prihaja. Zdaj tega ni bilo. Je pa čutila potrebo po fokusu in zavedanje katere barve so;

Zavedanje barv: to se je kdaj zgodilo samo od sebe;

Barv ni verbalizirala;

Testni dražljaj: ni bil mentalen, ampak zelo telesen;

Prepoznava enakosti: občutek zavedanja; neka gotovost, če je bila rumena, ji je bilo bolj udobno. Vendar, kadar se je zavedala le ene, ni bila več tako zelo pozorna na ostale;

Za en ali dva primera, se ji je to spoznanje zgodilo v glavi, drugače pa je bilo vse na računalniku;

Sprememba celotnega doživljanja: je nima;

165 S25-02-P-01

Ravno si je začela v glavi povezovati črte med pikami; črtkane črte;

Pred tem ni nič razmišljala o tem, da bi jih lahko povezala. Mogoče je pred zadnjim prišla misel, da bi jih lahko. Pri zadnjem pa jih je delno povezala;

Črte: definitivno ni bila cela črta, ne bi je mogla prerisati; ni bila zares črta, ampak sled v vodoravni oblik; izginjala je; ni imela barve, niti ni bla stalna, izginila je, ko je njena pika izginila;

Nastala je tako, da se je pika sočasno s črto prelila v drugo piko; videla je eno črto naenkrat;

Te črte so se ji dogajale;

Ugotovitev, da bi lahko vlekla črte se je kar naenkrat pojavila in jo je zaznala; ni se spraševala, zakaj bi to bilo uporabno, ampak je protu koncu kar naenkrat bila ta misel v glavi;

Misel je bila neverbalizirana;

Pavza: na začetku se je pavze še zavedala in vmes med eno je pomislila, da ne gredo v določeno obliko (pred tem se je zalotila, da je pomislila, da se pike vedno prikazujejo v istem liku, samo da se prikazujejo na drugem mestu); neverbalizirane misli;

Te misli so najbolj pogoste take neverbalizirane misli;

Črt ni namenoma narisala med pavzo;

Zaznala je nek ritem: sprva ji je bilo malo neprijetno, ker se ji je zdelo, da nima dobre prostorske predstave (če bi ji dal navodilo, naj pokaže, kje je bila ta pika, je ne bi znala pokazat: dražljaja ne bi mogla rekonstruirat) + zmotno prepričanje, da so znotraj enega lika, potem pa je pretvorila prostor v ritem → fokus je iz prostora prestavila na ritem;

Ritem: ni bilo nekega zvoka; tempo je bil vedno isti, kljub temu pa je bilo nekaj drugače; ritem je čutila glavi kot neko prisotnost; ritem je (mogoče) ustvarjala ona;

Testni dražljaj: poskušala se je spomnit, ali je isti ritem ali ni bil; ritem se ji je že sam dajal kot isti ali drugačen, nikoli ga ni aktivno primerjala; to je res dobila iz ritma, ne iz nečesa vizualnega, kljub temu da je obenem zavestno videla kroge;

Meta-kognicija: ne, ampak ji je bil občutek ritma v glavi pomirjujoč iz vidika, da ji je lažje slediti ritmu kot nekim prostorskim stvarem; → prijetnost je zelo majhen občutek; ni je zelo okupiral; neka mlačnost v telesu in mentalno (v prsnem košu, mentalno pa nekje v glavi); to je hipen občutek, ki pa je potem ostal: po tem, ko se ji je utrnila ta misel, se ni več obremenjevala s tem, ali ji je prijetno ali ne.

Zahteve naloge: razmišljala je o tem, da teh rezultatov sploh nihče ne gleda in iz tega vidika je bila precej bolj sproščena. Ni razmišljala o tem, ali je to prav ali narobe. Prisotna je že od začetka, je nekaj prijetnega, vendar je ni zares čutila v telesu.

166 S25-02-P-02

Zavestno je hotela razmišljat o teh črtah, v smislu če jih vidi tudi med testnim dražljajem, ker je v dveh prejšnjih razmišljala, da jih vidi le v spominskem. Zavestno je hotela biti pozorna na to → usmerila je svojo misel, da mora biti pozorna na to, vendar to ni bilo artikulirano, bilo je neko notranje dejanje. Bilo je popolnoma njeno. Čutila se je kot avtorica tega navodila;

Gesta refleksije: veliko se je ukvarjala s črtami, ampak ne iz vidika, da bi razmišljala o meni, ali nalogi, ampak iz vidika tega, kaj je prej opazila. Ukvarjala se je sama s sabo v kontekstu tega o čemer sva prej govorila;

Prva misel: »O, shit, hitreje je, ritem se je spremenil! (to je bila vsebina misli)« to je bilo bolj konkretno izrečeno kot ostale misli, ampak še vedno ni bilo verbalizirano; bila je neka vsebinska in opazna. Ta misel izstopa, je bolj izrazita. Sicer pa nima glasu. Izstopila je iz podlage – če imam podlago, kjer imam misli, je izstopala, lahko jo je tipala, medtem ko ostalih ne more – ona je bila njena avtorica; misel se je zgodila sama od sebe;

Spominski dražljaj: tej prvi misli je sledil občutek pomirjenosti, da je isto kot prej, ni pa bila to aktivna misel prvi. Ni se mirila. To ni bil nek dialog.

Spominski dražljaj: bolj je razmišljala o doživljanju, kot pa doživljala; spraševala se je o naravi povezovanja s črtami; premlevala je te misli;

Imela je neko vidno pozornost na dražljajih;

Premlevanja o GOBI ne bi mogla odriniti stran; zelo prisotno je bilo, hkrati pa ni bilo neprijento; zdelo se ji je, da zmore to razmišljati, da ima priložnost, da ima čas za to: → da ima čas je bil spet nek občutek med prsnim košem in glavo; to je bila neka jasnina; jasno je, da ne skačejo druge stvari noter (v smislu drugih misli ali občutkov) → tako si je dovolila, da začne premlevat. Ritem je bil del tega (ta neobremenjenost z nalogo); lahko je premlevala stvari tekom cele nalogo, ker si niti enkrat ni mislila, da ima v glavi ritem;

Meta-kognicija: imela ga je enkrat ali dvakrat: kot da bi se zarisane črte malo zabliskane (mogoče ima malo občutka, da je to nastalo med intervjujem) bilo je nekaj s svetlobo, nek kontrast. To je bilo bolj vizualno kot kaj drugega, ne bi pa rekla, da je bilo zelo. Pomen je bil, da je dobro reševala nalogo. Če se ji je to res zgodilo, je bilo to enkrat ali dvakrat zavestno z zadnjim krogom. To je bil nek aha moment;

Trenutek odločitve: onkraj njenega doživljanja.

167 S25-02-P-03

Od enega trenutka naprej so se ji misli vedno vračale na nek dogodek vmes; →

Dogodek: poskušala je povezati s črtami pike, ki so bile zdaj precej manj ritmične; črte so bile bolj izrazite in močne (prim. bolj kot prvič ko so se zgodile); med testnim je šlo vse čist drugam in spet so se ji črte povezale; zdelo se ji je, da jo spominjajo na klopčič volne, nek vozel (vizualno se ji je zdelo zmedeno, videla je prekrižan vozel iz teh črt); ob zavozljanosti je občutila neko paniko (telesna ob tem, da je popolnoma drugače; ne bi je mogla locirat, je pa ostala in vedno se je vračala na to);

Vračanje: ni več mogla jasno videti teh črt, ker je ostala panika, obenem pa je še vedno videla te črte;

Rekon: črte so se zgodile, bolj izrazite, črne, v njih je prepoznala vozel, ob čemer je začutila paniko, ki je ostala;

Teh črnih črt se ni mogla znebit in so vztrajale v naslednje primere;

Misli so se ji vedno vračale, vrinjale na ta klopčič;

Misli (preprosto so bile, niso bile izrečene) niso bile neprijetne, je bil pa neprijeten občutek, da jih ne more spustit;

Vedela je, da jo to ovira pri zdajšnjem reševanju; → nikoli ga ni ubesedila, niti ni razmišljala v smeri, da bi ga verbalizirala; imela je zavedanje, da jo to ovira; ne more locirati niti misli, ne fizičnega občutka o njem.

Ritem: ne ve, če je bil še prisoten;

Osrednje v njenem doživljanju so bile te črte;

Zahteve naloge: ni tako izrazito čutila, kako sedi; ni čutila določenih točk v svojem telesu, zdelo se ji je, da je zavzela več prostora; ne fizično, ampak kot da jo je več. Bilo je kot neka razlitost, kot da je povsod; ni neke točke v doživljanju, ki bi jo čutila;

Klopčič ji je bil nekaj zelo groznega. Prej je imela ata ritem, zdaj pa ga je to zelo močno presekalo. Ne iz vidika, da ji gre bolje ali slabše, ampak kakor da se je zgodila neka nesreča.

168 S25-02-P-04

Ponovno je bil občuten konflikt med spominskim in testnim dražljajem; močno je občutila razliko, ampak brez katastrofalne nesreče klopčiča; bilo je le močno narobe;

Močno narobe: neprijeten občutek, hkrati pa nič ne more narediti, da bi bilo drugače in se zaradi tega ona sama ne počuti slabo. Samo drugače je. Doživlja ga zgolj v prsnem košu;

Ta občutek ni vedenje, je nekaj fizičnega. Malo zaboli, ampak ne čuti bolečine. Se ji pa dogaja;

Spet je bil v ospredju bolj ritem; → ni nič bolj jasen kot prvič. Spet je bil v glavi; drugačnost dražljaja je bila pa v telesu, v prsih;

Že prej je pomislila, da bolj zaznava ritem in da je manj črt; → ta misel se ji je zgodila;

Mogoče je bila neka vidna komponenta v vidu; ker je videla, da se objekti ritmično prikazujejo; manj pa je bilo črt; bolj so bile blede;

Spominski dražljaj: spominskega in testnega je isto doživljala. Pri obeh je videla črne kroge in čutila ritem, ki ga je prepoznala kot drugačnega. S tem da se ni spraševala ali je isti ali drugačen; preprosto vedela je, da je drugačen; ni ga aktivno primerjala, ampak se ji je dajal kot drugačen;

Pavze se ne spomni;

Trenutek odgovora: onkraj njenega doživljanja, niti se ne spomni pritiska; zelo jo je zabolelo, da ni isto;

Bolečina ob drugačnosti: ni bila čustveno prizadeta, v prsnem košu; bila je točkasta in ne ploščata; usmerjena je bila točkasto vanjo; topa; bila je bolečina, ki ni bolela → ni ji pripisovala čustvene vrednosti, niti je ni prenesla v misli; ni razmišljala o tem, zakaj ali kaj bo zdaj; bilo je hipno;

Meta-kognicija: se ne spomni;

Odnos do naloge: vesela je bila, da se je vrnil ritem, čeprav si tega ni izrekla. Čutila je, da ima spet toplino, za razliko od izrazitih črt. Zaveda se, da ji je to ljubše (med intervjujem); na začetku se je bala, da bodo ponovno izrazite črte, da se bo klopčič spet tako izrazito zavil, ampak ker črte niso bile tako izrazite, to sploh ni bilo mogoče. Ni razmišljala o tem, da bi kdo gledal odgovore. Iz vidika naloge ji je bilo lahkotno reševati nalogo, ne pa iz vidika bojazni pred črtami. Bilo jo je strah teh črt, ampak to se je zelo hitro razblinilo;

169 S25-02-P-05

Sploh ga ni videla; odklopila se je in ni bila prisotna;

Ni videla krogov, še vedno je čutila zametke nekega ritma; to pa ni bilo dovolj, da bi zmogla prepoznat, kakšen je bil testni;

Ni videla črnih krogov in ritem je bil nek minimalen;

Tako po spominskem, kakor po testnem, je na isti način trznila s prstom, česar se ni zavedala; niti se ni zavedala prstov;

Poleg minimalnega ritma je imela misli in neko zmedo, kakor da je preveč misli in da se ne more odločiti, kaj bo mislila; ni se mogla odločiti, kateri misli bo verjela in sledila;

Ni imela zavedanja o telesu;

Nič takega se ni zgodilo niti v telesu niti v okolju, da bi se zavedala tega;

Imela je ogromno nekih misli; mentalno zavedanje; ni čutila neke teže, ali da bi bila njena glava. Totalno nasprotje jasnine od prej; te misli so se dogajale same od sebe in iste misli so se dogajale znova in znova na isti način; ponavljalo se je in ni šlo naprej; ne more poimenovati njene vsebine (prim. »o shit, …«); vsebina misli ji ni na voljo, ve pa da je bilo veliko različnih vsebin; vsebine so se pa ponavljale; oblika misli je bila nedoločljiva;

V trentuku intervjuja ji je frustrirajoče in too much, da ne more zagrabiti teh misli;

Naloga je bila popolnoma onkraj njenega doživljanja;

Vmes se spomni, da je videla križec, kako je potemnil, kar je za trenutek prevzelo njeno pozornost;

Ni se obremenjevala s tem, kateri dražljaj je spominski, kateri pa testni. Ni ji bilo važno; ni se spraševala o tem; tudi če bi se, ne ve, če bi se lahko prisilila, da bi ji bilo mar;

170 S25-03-O-01

Zelo podobno kot zadnjič, v smislu, da nima pojma, kaj dela.

Bolj ko ne je reševala na blef;

Ob tem ni bilo nekih neprijetnih občutkov, zaradi tega, ker ji ne bi šlo. Precej jebivetersko je bilo vse skupaj (→ bila je neobremenjena s to nalogo in ni se čutila pod kakršnimkoli pritiskom);

S tem je povezano prepričanje, da se rezultatov ne beleži → naknadno je razmišljala o tem in to ji je bilo v veliko olajšanje. To ji je omogočalo, da se bolj posveča sebi kot pa nalogi; to je prijeten občutek, ki pa ga ne more locirati. Celotno njeno doživljanje je obarvano s tem, ni pa to neka konkretna svoja stvar;

Vmes jo je prešinila neka misel, »sploh se ne osredotoča na levo spodaj …« kljub temu da je zavestno želela to narediti; → ta misel ni bila konkretno verbalizirana in jo je ubesedila šele med intervjujem. Bil pa je nek občutek, ki je imel to vsebino; ta misel se je zgodila sama od sebe, čeprav jo je na nek način zavestno producirala s tem, ko je hotela usmeriti pozornost; ta misel je bila neločljivo povezana s silo, ki jo je ustvarjala (beri *infra*);

Bilo ji je, kot da z neko nevidno silo, ki je imela neko smer, hoče fokus premaknit v levo spodaj (to zavestno ustvarja ona sama) To je bil zelo bežen trenutek. Sredinska utripajoča točka je bila tako močna, da je njen pogled preusmerila nase;

Meta-kognicija: enkrat samkrat je imela občutek, da pravilno rešuje, vendar ne ve, zakaj. To je bilo le zavedanje neke neizrečene misli. To ni nekaj, kar bi lahko ful razdelala. V tem jebiveterskem odnosu je bilo implicirano tudi to, da ji je vseeno, kako rešuje.

Zahteve naloge: že ko sem začel govorit navodila, se je zavedala materiala svojih hlač. Njena pozornost je padla na to, obenem pa se je ovedla svojega zavedanja. Med samim reševanjem je bil prisoten ta občutek zavedanja materiala hlač;

171 S25-03-O-02

Začela je razmišljat o tem, da ji ni prijetna vaja → konkretno jo je ubesedila in zelo se je začela zavedati svojega mežikanja;

Zmotijo jo repki; povzročajo ji neke neprijetne občutke. To so bolj telesni, kakor mentalni občutki, vendar ne tako, da bi jo karkoli bolelo, ali da bi ji bilo stresno. Gre bolj za preferenco, ki se kaže, kot da bi jo malo zabolelo;

Mežikanje: veliko je začela mežikanja; v glavi je mentalno spremljala to mežikanje. Sprva so bili mežiki avtomatični, potem pa jih je začela nadzirati; to je zavzelo precejšen del njenega mežikanja; mežikala je v ritmu prikazovanja dražljajev;

Njeno reševanje je bilo ponovno zelo neosredotočeno;

Iritacija: kot da bi hotela zaviti z očmi, češ da ji te črte niso všeč;

Parkrat je še poskušala reševat nalogo;

Pozornost je skušala usmeriti v objekt desno zgoraj, ker se ji je zdelo, da je bolj prisoten, pa ni šlo. Vendar to usmerjanje pozornosti ni bila tako izrazita stvar, kot prej. Zdaj se ji je definitivno dogajalo. To ni bilo aktivno usmerjanje pozornosti;

Spominski dražljaj: videla je, kaj je bilo na ekranu, vendar tega ni registrirala;

Pavza: z nekim občutkom pričakovanja je čakala na neprijetnost teh črt. Zelo se je zavedala teh pavz → pričakovanje je ustvarjala ona;

Testni dražljaj: ni imela dovolj energije, da bi se aktivno trudila. Na neki točki je imel občutek, da je ena mogoče obrnjena v pravo smer;

Trenutek odgovora: odgovarjala je z nekim občutkom. Večinoma so bili to odgovori na blef. Na neki točki je registrirala, da večinoma odgovarja z levo tipko.

172 S25-03-O-03

Občutek ima, da kinka, pri čemur se je malo predramila;

Ritem je bil podoben temu: ko je zakinkala in predramila, se je hitro zbudila in ima povezavo s pojavnostjo dražljajev. To je bila hitra reakcija, vendar ni registrirala dražljajev samih;

Zmedla se je glede tega, kaj je spominski in kaj testni dražljaj. Ob tem je avtomatično kliknila in leva stran telesa ji je trznila ob tem;

Imela je težke oči: zelo telesno doživljanje;

Kinkanje: ni bilo zares kinkanje. Začutila ga je v celem telesu in v glavi. Ta občutek je doživljala sprejemajoče. Ni se posebej želela prebuditi, ali nasprotovati temu;

Pozornost: osredotočala se je na svojo utelešenost, na očeh, ki so se ji zdele težke;

Dražljajev se ne spomni;

Večino časa je še vedno pritisnila na tipko; na podlagi ene črtice, ki jo je samo prepoznala. Enkrat je vedela, da je nepravilna, ker je bila popolnoma nasprotna;

Predramitev: malce neprijeten občutek, kakor da ve, da ne sme kinkat, pa vseeno kinka. Nek imperativ je v tem, češ da mora reševati nalogo, čeprav je ni zares reševala in se je zavedala tega, ampak je vseeno doživljala nek »morati.«

Morati: prej se ga ni zavedala, nastal je šele med intervjujem. To je samo neka prisotnost, ki pa je ni ustvarjala ona; čeprav jo je na neki točki ustvarjala tudi ona. Najprej je prišla nekje od zunaj, potem pa jo je ona sama nadaljevala.

Meta-kognicija: sploh nima ozaveščeno, kako pravilno je odgovarjala;

173 S25-03-C-01

Zadnji primer je bil neprijeten v smislu, da je pritisnila na napačno tipko;

Udeleženka je ob tem odkimala, vendar se tega ne zaveda;

Spominski dražljaj: ni bilo nič posebnega; prepoznala je vse štiri barve in ni se jih trudila zapomnit. Ni si ji ponavljala, ali preizkušala kake druge tehnike.

Pozornost: lebdeča pozornost na vseh štirih, čeravno je znotraj tega najbolj videla dve v diagonali. Dajala sta se ji kot bolj očitna. Občutek ima, da tega ne bi mogla spremenit;

Pavza: razmišljala je o tem, da so ji ene barvne kombinacije ljubše kot druge. To se je razpotegnilo čez več primerov. Kot da se je odprl en tok in ta misel je začela teči. Ni je mogla takoj ustavit. Vsakič znova, ko so se pojavile te barve, se je vrnila. Sprva so bile te misli verbalizirane, kasneje pa ne more več reči, ali so še bile besede, ali kaj drugega. Ima občutek tekočnosti. Prevzele so večino njenega doživljanja. Na začetku je ona priklicala to misel, potem pa ni bila popolnoma v nadzoru nad njimi. Te misli niso bile hotene, čeprav se poistoveti z njimi;

Pavza: za dve barvi bi lahko rekla, kateri sta; z izjemo enega primerja, ko bi lahko vse štiri;

Testni dražljaj: fokus je imela na diagonali; zagledala ju je in ju prepoznala na podlagi celotnega občutka; to je bilo na ekranu in je bilo drugačne oblike, kot kar je ona imela v glavi. Pri slednjem je ona dodala nekaj svojega.

Kar je imela v glavi: zelo vizualna prisotnost, ki je bila zelo vidna. Nahajala se je desno zgoraj. Težko bi jo opisala. Nima nekih mej. Na začetku bi iz tega agregata lahko izluščila posamezne barve, kasneje pa ne več. V resnici ni procesa od prej-potem. Zdi se ji, da vsaj diagonalni barvi, potem pa ne več; prisotno je v testnem dražljaju; tega ne počne aktivno, to se ji dogaja;

Prepoznava enakosti: nek občutek skladnosti s tem, kar ima v glavi in tem, kar je na ekranu. Tega ne primerja aktivno, ampak se samo zgodi;

Meta-kognicija: nima občutka, edino enkrat je bilo drugače. Takoj na začetku je čutila olajšanje nad tem, da so barve, obenem pa je čutila večji pritisk.

Olajšanje: telesen občutek, zelo majhen, zamenjal pa ga je občutek, da mora dobro rešiti nalogo. Zamenjal ga je strah pred neuspehom;

Zahteve naloge: za razliko od prej se ni več ukvarjala s tem, da so ji črte neprijetne. Zdaj je bil prisoten nek njen pritisk, da mora boljše reševati. Strah jo je bilo neuspeha → zelo majhen ampak

močan občutek. Ta strah je bil vodoraven gib vanjo. Ni je zbolelo. Niti ne bi rekla, da je bilo v njej, ampak se ga je s telesom zavedala. Gib je prišel od spredaj vanjo.

174 S25-03-C-02

Ko je zagledala barve, jih je začela povezovati z asociacajami; tekom celega bloka je nanje pripenjala neke asociativne misli;

Asociacije so se zgodile samo pri spominskem dražljaju;

Ne ve, ali jih je ustvarjala, ali so se ji dogajale;

Asociacije: to je bila ena beseda, ki se prikaže v istem ritem in ostane desno zgoraj; napisana beseda + njena vsebina. Jasno vidi obliko črk, kakor da bi jih lahko začutila kot nek relief. Ni pa to vizualno izkustvo;

Pavza: enkrat se je vrnila k asociaciji in je preverjala asociacija → pomislila je na zastavo ene države; → te misli o pravilnosti asociacije niso bile izgovorjene. Bila je le neka prisotnost; le neka bežna misel;

Testni: prepoznala je posamezne štiri, s tem da si jih ni poskušala zavestno zapomnit; pogledala je vsako posebej. Aktivno jih je primerjala s spominskim dražljajem na podlagi nekega občutka. To ni bil ta desni zgorajšnji agregat kot prej. Bilo je bolj izrazito;

Testni: med spominskim si jih je zapomnila in potem je vedela, da je prav ali ni;

Meta-kognicija: najbolj prisoten doslej. Kazal se ji je kot večja osredotočenost. Kljub asociacijam, ki so bile včasih smešne ali prijetne, je lahko držala fokus na štirih barvah.

175 S25-04-O-01

Osredotočala se je na desno polovico, v zadnjem primeru pa ni bilo nič kaj posebnega razen tega, da se ji je zdelo, da dražljaja na podlagi desne polovice nista enaka;

Ugotovila je na podlagi tega, v katero smer so bile obrnjene;

Ni aktivno ohranjala reprezentacije med pavzo;

Jih je pa včasih poskušala verbalizirano usmeriti, ampak je s tem imela probleme;

Smeri ni zares verbalizirala;

Za testnega je takoj vedela, da je napačen, ko je bil napačen, ko je bilo pa isto, pa je aktivno primerjala in to ji je vzelo več časa;

Leve strani sploh ni zaznavala;

Meta-kognicija: zelo majhen mentalen občutek, da uspešno rešuje + dvom, ker se zaveda, da opazuje samo desno stran → dvom se ji je kazal kot neka kratka misel, ali zavedanje, da nima zares aktivne strategije;

Ni nekega posabenga ozadnjega občutka; na koncu jo je malo začelo nervirati, ker je uporabljala slabo strategijo, ni se pa zavestno odločila, da bi mogoče spremljala tudi levo stran.

Nič ni razmišljala o tem, da ji je bilo lahkotno reševati nalogo;

Pri pritisku tipke si ni ponovila, kaj je bilo kaj, avtomatizirano je pritisnila na tipko in ni imela ozaveščeno, da je desna pravilna, leva pa napačna.

176 S25-04-O-02

Bolj je bil prisoten občutek, ali ji gre dobro, ker se ni držala le desne strani, ampak je bolj poskušala zajeti celotno sliko. Bil je močnejši občutek, da ji dobro gre. Enkrat je trznila z nogo. Čutila je neko energijo v nogi, ker sedi prekrižano;

Celotna izkušnaj je bila malo bolj telesna kot prej; to je bilo po občutku, da dobro rešuje;

Pogledala je vse štiri objekte;

Pogledala je samo repke in si poskušala ustvariti sliko smeri v katero so obrnjene;

Videla je le palčke, ne krogov;

Reševala je po občutku;

Slika, ki jo je ustvarjala je bila 2D, na ekranu; videla je tudi med pavzo in občutek ima, da jo je ona ustvarjala;

Ob ustvarjanju te slike je zelo hitro izgubljala fokus; npr. ko je začutila nogo, ji je zmanjkalo fokusa;

Testni dražljaj se je na nek način usedel na reprezentacijo in če je bilo isto ali ne, je odgovorila. To so bile zelo medle, nejasne slike;

177 S25-04-O-03

Zdelo se ji je, da je bilo pravilno; razlikovalo se je od prejšnjih, kjer je imela občutek, da ji ni šlo dobro;

Meta: uspelo se ji je osredotočit na celotno sliko; avtomatično se je poskušala osredotočit na desna dva, ampak ji to ni uspevalo;

Bolj ji je bilo pomembno, da se trudi glede strategije, kakor da je uspešno reševala nalogo;

Skušala je ohranjat boljšo strategijo, vendar ji ni upsevalo, bilo je kakor da je bila prepočasna;

Enkrat je paličice poskušala ubesedit in je uspela zajeti samo levo polovico, niti se ne spomni več, kaj si je rekla;

Ugotovila je, da ji ne bo uspelo, da je prepočasna;

Testni dražljaj je spremljal občutek, ali je isto ali ni, vendar ni bilo tega usedanja. Vse je bilo bolj medlo in ni bilo neke gotovosti, sploh ko je bilo narobe;

Zahteve naloge: manj ji je bilo prijetno samo reševanje te naloge;

178 S25-04-P-01

Reševanje naloge je bilo prijetno → utelešen občutek; ob tem ni imela nobenih misli, razen da je pomislila, da ji je ljubša pozicija kot orientacija;

Zdi se ji, da je pravilno ugotovila → ne bi ga mogla posebej opisat;

Od polovice naprej je začutila eno živčnost; češ da gre nekaj prepočasi; želela si je, da bi šlo naprej. Pavze so bile predolge → zaradi tega je bila na koncu malo nemirna ali živčna;

Strategija se ji je dala sama od sebe, vendar manj aktivno kakor v prejšnji. Samo sledila je tem krogom s pogledom. Ni se zares ubadala s strategijo;

Ni ponavljala pik med pavzo;

Testni dražljaj: preprosto je sledila z očmi;

Včasih je pomislila, da je kaka pika bolj na levo ali na desno in si je zaželela, da bi bile bolj očitne razlike med njimi;

179 S25-04-P-02

Postala je zelo zaspana;

Na koncu je težje sledila dražljajem → včasih kar ni vedela, ali so se pojavile prej ali ne; pozabila je, kateri dražljaj je pred njo; struktura naloge je razpadla zanjo;

Zelo se je zavedala svojega telesa; močno je čutila utrujenost, ki se je iz glave razširila na celo telo, kakor da je postala zelo težka, sploh v nogi, ker ima prekrižane, je vse postalo zelo težko;

Poleg tega, da se ji je zdelo težje zaradi urtuejnosti, ji je še pozornost šla na njeno utelešenost;

S pogledom je sledila pikam; bilo ji je pa težje, ker ni bila zbrana;

Med pavzo ni ponavljala, se je pa spraševala o tem, ali je zdaj ponovitev ali spominski dražljaj;

Spet je sledila s pogledom in je vedela, če so bili drastično drugje te krogi;

Meta: ima občutek, da ji je nekajkrat uspelo odgovoriti na nalogo. ni toliko, da je pravilno odgovarjala, ampak da je včasih odgovarjala narobe;

S25-04-P-03

Zelo ji je v spominu pavza ostala, ker je bila zelo osredotočena na križec;

Vidi ga nekje v ozadju, med zadnjim primerom je bil pa zelo jasem;

Zdi se ji, da je rešila prav;

Sledi z očmi, med pavzo ji je pozornost padla na križec, potem je spet sledila z očmi in preprosto vedela, ali je isto, ali ne.

Zaspanost se ohranja, vendar tega ni občutila telesno, se ji pa zdi, da je bolj počasna v smislu, da tem krogom ne sledi z njihovo hitrostjo, ampak da jih ne dohaja. Zdi se ji da so veliko hitrejši od njenega pogleda.

180 S25-04-C-01

Zdi se ji, da so bile barve iste;

Razmišljala je, če bi jih znala med intervjujem rekonstruirat, vendar jih najbrž ne bi;

Spominski dražljaj: bilo ji je prijetno, všeč ji je bila ta naloga in barvna kombinacija;

Ko ji barvna kombinacija ni všeč, se je težje vrnila k njej v spominu;

Pogledala je celoto;

Pavza: razmišljala je o hrani;

Ni bilo verbalizacij;

Med pavzo ne bi mogla rekonstruirat teh štirih barv;

Drugi ji ni bil prijeten in je bil ta kontrast dal vedet; sploh gornji dve;

Testni: takoj ve, da ni enako, oz. zdi se ji, ni pa neke aktivne primerjave;

Ozadnji občutek: ni več tako zaspana, prisotna pa je neka utrujenost, le da ni aktivno zaspana in ne čuti teže telesa.

181 S25-04-C-02

Med pavzo si je ponavljala barve; vendar niso bile izgoverjeno, v glavi je le imela neko jezikovno vsebino;

Pri zadnji se je lomila, ker sta bila dva različna odtenka modre;

Med spominskim si je že govorila barve;

Včasih ji je bilo to zelo težko. Sploh ni mogla poimenovat ene ali več barv. Kot da ne bi vedela neke barve, čeprav je vedela, da ve;

Meta: ima občutek, da je uspešno reševala nalogo. to je bil mentalen občutek; to je bil nek močan občutek gotovosti, iz česar izhaja ta samozavest.

182 S25-04-C-01

Zdi se ji, da so bile barve iste;

Razmišljala je, če bi jih znala med intervjujem rekonstruirat, vendar jih najbrž ne bi;

Spominski dražljaj: bilo ji je prijetno, všeč ji je bila ta naloga in barvna kombinacija;

Ko ji barvna kombinacija ni všeč, se je težje vrnila k njej v spominu;

Pogledala je celoto;

Pavza: razmišljala je o hrani;

Ni bilo verbalizacij;

Med pavzo ne bi mogla rekonstruirat teh štirih barv;

Drugi ji ni bil prijeten in je bil ta kontrast dal vedet; sploh gornji dve;

Testni: takoj ve, da ni enako, oz. zdi se ji, ni pa neke aktivne primerjave;

Ozadnji občutek: ni več tako zaspana, prisotna pa je neka utrujenost, le da ni aktivno zaspana in ne čuti teže telesa.

183 S25-04-C-02

Med pavzo si je ponavljala barve; vendar niso bile izgoverjeno, v glavi je le imela neko jezikovno vsebino;

Pri zadnji se je lomila, ker sta bila dva različna odtenka modre;

Med spominskim si je že govorila barve;

Včasih ji je bilo to zelo težko. Sploh ni mogla poimenovat ene ali več barv. Kot da ne bi vedela neke barve, čeprav je vedela, da ve;

Meta: ima občutek, da je uspešno reševala nalogo. to je bil mentalen občutek; to je bil nek močan občutek gotovosti, iz česar izhaja ta samozavest.
