# Supplementary material for: What Individuals Experience During Visuo-Spatial Working Memory Task Performance: An Exploratory Phenomenological Study
Source: Front Psychol. 2022 May 18;13:811712. doi: 10.3389/fpsyg.2022.811712 (PMC9159378; doi:10.3389/fpsyg.2022.811712)
Supplement: Supplementary file 2 [file Data_Sheet_2.PDF]

Phenomenology of Visuo-spatial Working Memory Task Performance

Supplementary Materials B.2: Data Notes

1 Introduction

The present document is a description of the quantitative data in the study *Phenomenology of Visuo-spatial Working Memory Task Performance.* The quantitative data is made available in the file *WM-Phenomenology-SM-B.1-Data.xlsx.*

2 Data notes

The spreadsheet only includes valid samples (i.e., samples in which participants were able to provide a subjective report on their experience of performing the most recent trial of the visuo-spatial change detection task).

An individual sample can include multiple rows, in the cases where participants reported on multiple aspects of their experience.

2.1 Variables

The spreadsheet includes the following variables:

- Sample_ID: Identification number of the sample;
- Participant: Identification number of the participant;
- Task: Task condition (C – color; O – orientation; P – position);
- Acc: Performance accuracy (C – correct response; W – wrong response; N – no response);
- Rt: Reaction time (measured in milliseconds);
- Level5: Coding at V-level;
- Level4: Coding at IV-level;
- Level3: Coding at III-level;
- Level2: Coding at II-level;
- Level1: Coding at I-level.
