# Supplementary material for: What Individuals Experience During Visuo-Spatial Working Memory Task Performance: An Exploratory Phenomenological Study
Source: Front Psychol. 2022 May 18;13:811712. doi: 10.3389/fpsyg.2022.811712 (PMC9159378; doi:10.3389/fpsyg.2022.811712)
Supplement: Supplementary file 3 [file Data_Sheet_3.pdf]

Phenomenology of Visuo-spatial Working Memory Task Performance

Supplementary Materials C: The Annotated Codebook

Table of Contents

1 Introduction 4

2 Saturation Grid 4

3 The Codebook 6

3.1 Phenomena at the front of consciousness (level V category) 7

3.1.1 Encoding (level IV category) 7

3.1.1.1 Active encoding (level III category) 8

3.1.1.1.1 Staring (level II and I category) 8

3.1.1.1.2 Reducing complexity (level II category) 9

3.1.1.1.3 Motor planning (level II strategy) 9

3.1.1.1.4 Searching (level II and I category) 10

3.1.1.1.5 Describing (level II and I category) 11

3.1.1.1.6 Imagining (level II and I category) 11

3.1.1.2 Passive encoding (level III category) 12

3.1.1.2.1 Visual feeling (level II and I category) 12

3.1.1.2.2 Recognizing a pattern (level II category) 13

3.1.1.2.3 Acoustic (level II and I category) 14

3.1.2 Maintaining (level IV category) 15

3.1.2.1 Active maintaining (level III category) 15

3.1.2.1.1 Rehearsing (level II category) 16

3.1.2.1.2 Imagining (level II category) 16

3.1.2.1.3 Waiting (level II category) 16

3.1.2.2 Passive maintaining (level III category) 17

3.1.2.2.1 Impression (level II and I category) 18

3.1.2.2.2 Afterimage (level II and I category) 19

3.1.3 Recalling (level IV category) 19

3.1.3.1 Active recalling (level III category) 19

3.1.3.1.1 Comparing (level II category) 20

3.1.3.1.2 Comparison with a mental image (level I category) 20

3.1.3.1.3 Applying eye-movement (level I category) 21

3.1.3.1.4 Applying verbal descriptions (level I category) 21

3.1.3.1.5 Guessing (level II and I category) 21

3.1.3.1.6 Mixed recalling (level II and I category) 22

3.1.3.2 Passive recalling (level III category) 22
3.1.3.2.1 Appearing (level II and I category) 23
3.1.3.2.2 Hunch (level II and I category) 24
3.1.4 Mind-wandering (level IV - I category) 24
3.1.5 Meta-cognition (level IV category) 25
3.1.5.1 Explicit (meta-cognition) (level III category) 25
3.1.5.1.1 Commenting (level II and I category) 25
3.1.5.1.2 Attention (level II and I category) 26
3.1.5.1.3 Monitoring (level II and I category) 26
3.1.5.2 Implicit (meta-cognition) (level III category) 27
3.1.5.2.1 Meta-cognitive feeling (level II and I category) 27
3.2 Background feelings (level V category) 27
3.2.1 Overarching states of mind (level IV category) 28
3.2.1.1 Attentional dispositions (level III category) 28
3.2.1.1.1 You towards the task (level II and I category) 28
3.2.1.1.2 The task to you (level II and I category) 29
3.2.1.1.3 Coupling with the task (level II and I category) 29
3.2.1.1.4 Separation from the task (level II and I category) 30
3.2.1.1.5 Off-task (level II and I category) 30
3.2.2 Attitude towards the task (level IV category) 30
3.2.2.1 Difficulty (level III category) 31
3.2.2.2 Attitude towards task-performance (level III category) 31
3.2.2.3 Engagement with the task (level III category) 32
3.2.3 The atmosphere of experience (level IV and III category) 34
3.2.3.1.1 Positive neutrality (level II category) 34
3.2.3.1.2 Smoothness of performance (level I category) 35
3.2.3.1.3 Calmness (level I category) 35
3.2.3.1.4 Rhythm (level I category) 36
3.2.3.1.5 Fatigue (level II and I category) 37
3.2.3.1.6 Informational chaos (level II and I category) 37
3.2.3.1.7 Faith in recognition (level II and I category) 38
4 References 38

1 Introduction

This document contains the *annotated codebook* for the study *visual representations in working memory, study 1* (abbreviated as *VR.WM.1.*). The document amounts to supplementary materials aiming at establishing the rigor of the processes of data acquisition and analysis according to the standards of qualitative research. In this document, we have outlined the *annotated codebook*, an instrument in qualitative research with which we can ascertain the validity of a given research (Nelson, 2017). The annotated codebook consists of two parts: a) the saturation grid; and b) the codebook.

2 Saturation Grid

The saturation grid is a tabulation representing participants with whom we have induced a new experiential category. Columns represent participants, and rows represent codes (prior to being subjected to relational coding). The symbol "X" denotes the first occurrence of a given code. We can consider the data in a given qualitative study broad (i.e., enough data has been gathered so as to make further recruitment of participants unnecessary) when no new codes occur. According to the saturation grid, we achieved a sufficient breadth of data with participant WM.VR.1.14.

**Code/Participant**	**1**	**2**	**3**	**4**	**5**	**6**	**7**	**8**	**9**	**10**	**11**	**12**	**13**	**14**	**15 - 31**
Abstract image														X	
Acoustic component				X											
Afterimage		X													
Applying eye-movements				X											
Applying verbal description	X														
Boredom	X														
Calmness	X														
Concrete image							X								
Comparing with a mental image	X														
Competitive attitude				X											
Coupling with the task	X														
Difficult task		X													

Faith in recognition				X											
Grouping		X													
Guessing										X					
Hunch	X														
Impression	X														
Informational chaos	X														
Knowledge about paying attention	X														
Leaning forward	X														
Meta-cognitive feeling	X														
Motor strategy		X													
Mixed recalling							X								
Obligation	X														
Observing experience	X														
Off-task										X					
Omitting					X										
Other	X														
Playful						X									
Rehearsing eye-movements				X											
Rehearsing verbal descriptions	X														
Rhythm				X											
Searching	X														

Separation from the task	X														
Simple task					X										
Smoothness of performance				X											
Staring				X											
Stimulus appears identical/different	X														
Symbol					X										
Symbolized oversight	X														
The task towards you	X														
Towards the task	X														
Unburdedness					X										
Vain attitude	X														
Void					X										
**New codes discovered**	22	4	0	8	5	1	2	0	0	2	0	0	0	1	0

3 The Codebook

The purpose of a codebook is threefold. Firstly, the aim of the codebook is to elucidate the process of the coding of raw data. In doing so, the codebook represents an important instrument within qualitative research as it enables an independent researcher to familiarize themselves with the coding process in a reasonable amount of time. Secondly, the codebook represents a criterion of validity of the gathered data. Valid coding framework is considered to be the one that yields a fully specified codebook without any major gaps in the coding. Thirdly, the purpose of the codebook is to organize the gathered qualitative data.

The codebook outlines the induced experiential categories by organizing them into *levels of coding.* The lowest order of coding represents the smallest degree of abstraction from the raw data, whereas higher orders represent classes into which many lower-order categories are combined; i.e., they represent a higher degree of abstraction from the raw data. Crucially, categories on the lower orders of coding were entirely induced from the data. Conversely, categories on the higher orders of coding were grouped together based on insights from the extant psychological (e.g., dividing working

memory into *encoding, maintenance,* and *recalling*), phenomenological (e.g., the category *atmosphere of experience*), and neuroscientific (e.g., the category *overarching states of mind*) theory.

The present codebook has been constructed in line with earlier empirical phenomenological studies (Hurlburt & Heavey, 2006; Kordeš et al., 2019; Oblak, 2020). Based on these examples, entries in the codebook are specified according to five elements:

- Name of the category;
- Description of the category;
- Subcategories/values;
- Examples;
- Considerations.

Name of the category refers to an easy-to-remember name that was assigned to a specific experiential category during the coding process. The names were selected by the coders. Description of the category provides a definition of a given experiential category based on its primary features as observable in the raw data. Subcategories/values denote how a given experiential category can be further subdivided. On the lowest level of coding, the categories contain values rather than subcategories. Examples contain direct quotes from the raw data. Finally, considerations include any notes and comments by the coders that are relevant to understanding of the coding process. Primarily, this element points out specific differences between similar experiential categories.

3.1 Phenomena at the front of consciousness (level V category)

Description: This category refers to the aspects of experience that the participants are aware of in a given moment as the thing that is most apparently happening to them or that they are doing. It is an experience that is easily accessible in reflection, and roughly corresponds to the theoretical idea of the *shallow conception of consciousness;* i.e., conscious phenomena are those that we are directly aware of when we live the experience and not those that we become aware of during the gestures of awareness (cf. Froese, Gould & Seth, 2011; Froese, Gould & Barrett, 2011)

Subcategories:

Encoding;

Maintaining;

Recalling;

Mind-wandering;

Meta-cognition.

3.1.1 Encoding (level IV category)

Description: *Encoding* refers to the experience of memorizing a stimulus during a visual-spatial working memory task. *Encoding* as a category is defined by the temporally delineated phase of the visual-spatial working memory task; i.e., by the duration of the memory stimulus.

Subcategories:

Active encoding;

Passive encoding.

3.1.1.1 Active encoding (level III category)

Description: *Active encoding* is an experiential category that describes a strategic attitude towards the task (i.e., the participants actively go about solving the task) in which they attempt to memorize the memory stimulus. By the term *active,* we mean that participant experience the memorization of the initial stimulus as something that they do, rather than something happening to them.

Subcategories:

Staring;

Reducing complexity;

Motor planning;

Imagining;

Searching;

Describing.

3.1.1.1.1 Staring (level II and I category)

Description: Staring is an experience in which the participants simply look at the stimulus hoping to remember it. They experience a sort of tendency towards remembering, however, in doing so they have no real strategic attitude towards the task. The only thing they are mentally doing is by effortfully looking at the stimulus, hoping that they will somehow remember it. By extension, staring is defined by a sort of hopelessness and resignation in the form of the awareness that this strategy will (in all likelihood) be unsuccessful.

Values: None.

Examples:

> "I am simply looking and I get the feeling in which direction they are turned, but not also from which direction they are originating. And then, I have a feeling that I am holding something, that I am keeping something. And I am really trying to hold on to it. And during the delay, there's really this strong sense of my holding something, as if I actually have it. And then the test stimulus appears and I see that in reality, there was nothing there. And that I actually don't know." (VR.WM.1.22-03-O-01)

> "I tried to visually burn it into my memory. It was this very focused attention. Like an attempt at focusing my attention. It was accompanied by this anxiety that I won't be able to remember it all." (VR.WM.1.19-03-C-02)

Considerations: Staring is different from simply looking at the stimulus and consequently knowing whether the test stimulus is identical to or different from the memory stimulus. While staring, the participants more or less know that this strategy will be unsuccessful. It is a last ditch effort at solving the task. On the other hand, when simply looking and subsequently knowing, the experience is light and easygoing.

3.1.1.1.2 Reducing complexity (level II category)

Description: Reducing complexity refers to different strategies the participants used to make the memory stimulus easier to remember. This mainly happened in two ways: grouping and omitting objects. Grouping refers to a varied experience whereby the participants create a subset of objects that somehow belongs together (e.g., the subset forms a logical pattern). Omitting objects refers to a radical version of reducing complexity in which the participants decide or - due to the complexity of the task - have to opt to not try to memorize all the objects of the memory stimulus.

Values:

Grouping;

Omitting objects.

Examples:

Grouping: "I had some sort of a sense of color. I memorized it by forming couples that belonged together. And this grouping led to the image as a whole looking more or less ugly. If the test stimulus was prettier or uglier, I knew that it was also different." (VR.WM.1.10-03-C-02)

Grouping: "I was once again creating couples out of colors according to some tag. For example, if they form basic colors, or what is the spatial relationship between them. Like diagonal or something. I verbalized them. But not in the form of a whole sentence. It's more of an exclamation. Like an interjection. It has tone. The one that form a whole have a similar tone to them. The tone is different from the other two." (VR.WM.1.14-01-C-01)

Omitting objects: "I focused on the right half. In the last example, there was nothing special, except that I had a hunch that the stimulus seemed different based on the objects to the right." (VR.WM.1.25-04-0-01)

Omitting objects: "In the memory stimulus, I saw the combination: red, white, green plus an outlier. And so for each one, all I had to do was remember the position. First, I remember the outlier, and then the stuff around it. In my experience, the outlier seemed to be missing. In a way, it was something that wasn't there." (VR.WM.1.14-01-C-04)

Considerations: Within reducing complexity, the primary strategy can be any way of solving the task. Commonly, the participants use one strategy on one subset of objects, and a different strategy on the other subset of objects.

3.1.1.1.3 Motor planning (level II strategy)

Description: Motor planning describes the experiential category in which the participants become aware of their own eye-movements. They thematize these eye-movements - i.e., bring them to the front of their awareness - in different ways. Among others, they can mobilize the eye-movements strategically to solve the visual-spatial working memory task. Eye-movements therefore form a scale of awareness, where on one end of the spectrum, they constitute a mechanical activity that serves no further purpose than traverse the stimulus. On the other end of the spectrum, the participants may

mindfully memorize the feeling of their eye muscles that accompanies them observing the stimulus. According to the attention that is paid to the eye-movements, they can be experienced in three different ways, and by extension, mobilized as performing different functions.

Subcategories:

Pushing away;

Moving the eyes;

Path.

Examples:

Pushing away: "It seems to me that the eye-movements and attention are just one part of that. In a way this movement is almost visual. Yes. it's as if it is forced upon the screen [...] When comparing, I get a strong sense of the eye movements. It is not visual. It is in my body. It feels harmonic or dissonant. As if the two patterns resonate with each other." (VR.WM.1.29-01-O-01)

Moving the eyes: "I memorized it through the eye-movement. This eye-movement was continuous. I slowly look from point to point. I don't experience the path itself. It is an empty space. This path is less clear than the pockets of emptiness that appear when the objects disappear." (VR.WM.1.19-02-P-02)

Path: "I followed the same trajectory with my eyes as the dots did. I went along with their traces. Where there were dots, these pale images remained. The connections between them were paler still. It was a very subtle experience." (VR.WM.1.19-02-P-02).

3.1.1.1.4 Searching (level II and I category)

Description: Searching is a memorizing strategy in which the participants attempt to find a pattern in the stimulus. Essentially, it is not necessary that this strategy yields a pattern. It is more that the participant take an analytical attitude whereby they attend to the stimulus in such a way that they attempt to explore it. For most participants, this inquisitorial attitude is enough to memorize the stimulus.

Values: None.

Examples:

"I tried to form a geometrical pattern that would encompass all things on the screen. I was looking for a path according to which the objects would nicely conclude. So, each element had to connect with at least one other element." (VR.WM.1.14.03-O-01)

"I realized that it is easier to remember if I find patterns in the images. S, I look whether the lines are arranged according to some visual rules. I glanced at it and immediately tried to find a new rule. These rules are intuitively clear to me. They weren't verbalized. I wanted to verbalize them. But I didn't have to." (VR.WM.1.05-02-O-02)

Considerations: Describing is different from the experiential category recognizing a pattern in that the latter is automatic. It amounts to a sudden association, related to the stimulus which appears in participant's awareness as soon as they look at it. In other words, there is no inquisitorial attitude to recognizing a pattern.

3.1.1.1.5 Describing (level II and I category)

Description: The experiential category describing refers to situations in which the participants experience inner speech. Inner speech can be multifarious in their experience. It can be articulated and heard, articulated and unheard, or unarticulated and unheard. Alternatively, it can be simply the awareness of some sort of linguistic content. Further, within the experience of inner speech, we can observe a difference between active and passive inner speech (i.e, speech that the participant do and speech that is happening to them). At the level of the strategic use of describing to solve a visual-spatial working memory task these differences - although phenomenologically relevant - are not of interest to us. Therefore, we understand describing in a general sense as verbalized thoughts.

Describing therefore encompasses strategic use of inner speech where the speech is willfully articulated and on the level of content refers to describing objects, stimuli, ways of solving the task, etc. In short, describing refers to any description rendered in inner speech used by the participants for successfully performing the visual-spatial working memory task.

Values: None.

Examples:

> "My reasoning took the form of words. It was something quick without any linking words. I can have a much shorter dialogue with myself than with other people. Out if this flow of words, I could assume about the difference of the test stimulus. I was basically comparing the mental scheme that I put up. I knew that if the test stimulus was equal to the memory stimulus, I knew that I wouldn't reason up such a mental scheme." (VR.WM.1.14-01-C-03)

> "I described the stimulus with words. I feel authorship over these words, but I don't think that I chose them. Or that I produced them with a specific purpose." (VR.WM.1.04-01-C-03)

3.1.1.1.6 Imagining (level II and I category)

Description: When we are talking about imagining, we are talking about an aspect of experience in which the participants either willfully or automatically imagine a visual image of the memory stimulus. This visual image can be located on-screen or within the participant's mental space. It is an experience that is comparable with producing a mental image during the interview (which we tested by asking them to imagine an apple and compare it to the strategy of memorizing).

Participants may detect these images in a mental space. This is often described as a parallel world, overlaying the world perceived via vision. The participants can willfully shift their attention between these two "worlds." In this case, the mental image is in the minds of the participants.

When this experience is seen outside of the mental space of the participants, it is typically imagined on-screen. It amounts to a kind of projection onto the screen. This aspect of experience is typically very clear, visual, and related to the participant's attention. If they pay attention to something else, the mental image on the screen begins to fade.

It is not necessary that participants imagine the memory stimulus such as it was, but may form a variety of imagined constructs around it, which make the memorizing process easier. They can, for instance, imagine lines connecting individual objects, aggregates of colors, etc. Commonly, imagining can be related to automated associations, appearing as momentary visual images.

Examples:

> "I was creating a line connecting one with another in my mind. This line was black. It had no thickness. I saw it on the screen. It did not just appear all of a sudden. I had to draw it." (VR.WM.1.05-05-C-02)

> "I held on to a sort of a mental image that I saw in front of me on the screen. The objects were more gray. Grayish. Like a shadow. But a shadow in which it was apparent what they are supposed to represent." (VR.WM.1.16-04-P-01)

> "It was a kind of a mental image that I saw. It was as if I was projecting it in front of me. But it wasn't a part of this world." (VR.WM.1.05-05-C-02)

> "This was some sort of imagination. It was passive. It was definitely something that was happening to me. It was located inside of this mental space in my gaze. It is different than, for instance, looking at a red dot and then looking away and seeing a green afterimage. It wasn't on the screen. It really was something visual and it was located in my mental space." (VR.WM.1.05-C-01)

3.1.1.2 Passive encoding (level III category)

Description: *Passive encoding* is an experiential category that describes a serendipitous attitude towards the task (i.e., task-performance is something that they are passively involved with) in which they attempt to memorize the memory stimulus. By the term *passive,* we mean that participant experience the memorization of the initial stimulus as something that happens to them, rather than something they do.

Subcategories:

Visual feeling;

Recognizing a pattern;

Acoustic.

3.1.1.2.1 Visual feeling (level II and I category)

Description: This experiential category refers to situations in which participants implicitly ascribe certain visual properties to stimuli, such as density, weight, lightness, etc. Typically, this is something that happens to them automatically. Considering that this experiential category refers to visual properties, it can happen with any stimulus modality. Individual colors or color combination may evoke different feelings in participants, such as heaviness, airness, joy, etc. These feelings are not verbalized but are typically tied to emotions or bodily sensations. Colors or shapes can be associated with feelings

(of, for example, weight, density, or direction). While visual feelings refer primarily to the visual modality, they may be accompanied by a bodily sensation as well.

Values: On the lowest level of coding, the category *visual feeling* can take any value. As the value on the lowest level of coding essentially amounts to the content of *visual feelings* (e.g., the overall sense of the color arrangement), these do not constitute phenomenal data.

Examples:

> "I noticed that green has a disgusting sense to it. It's a very complex feeling. It is simultaneously mental, bodily and tied to memories. It happens simultaneously with the visual image. As soon as I look at the stimulus [pause] This is something that happens to me, but I only noticed it after starting to pay attention to it." (VR.WM.1.10-03-C-02)

> "I could remember it because the pattern had this nice feeling to it. The bottom ones did not break apart the direction created by the top ones. And so the entire direction had a sensible feeling to it." (VR.WM.1.13-01-O-05)

Considerations: Visual feeling is a different category from recognizing a pattern. While visual feeling describes a more subtle, wholesome sense of the stimulus, recognizing a pattern is a more conceptual experience. Recognizing a pattern is pregnant with a potentiality for verbal description, while with visual feeling, verbalizations are typically inadequate to fully describe them.

3.1.1.2.2 Recognizing a pattern (level II category)

Description: Recognizing a pattern is an experiential category that refers to situations in which participants look at a given stimulus, which immediately presents itself to them in the form of some sort of association: it appears tied to a content, which is not exclusively related to the visual nature of the stimulus. This link between what the stimulus is an additional content is something that happens to the participants (i.e., it is a passive process). Considering the content, recognizing a pattern can be further divided into three subcategories: recognizing a symbol, known abstract image, and concrete image.

Recognizing a symbol refers to whether the stimulus is similar to some well-known visual sign. Often, this experience is related to straightforward recognition of basic geometric shapes, symmetry, etc. Known abstract image refers to participants recognizing an abstract, higher-order content in the stimulus. This content does not present itself to participants in the form of mental imagery, but as feelings that they can then further unfold into concrete contents. Most commonly, we are talking about nation state flags, sports team colors or shapes that are implicitly reminiscent of known images (e.g., faces). Concrete image refers to visual imagery, elicited by the stimulus in the participants. Unlike imagining - which is an active gesture - concrete image appears automatically and typically uncontrollably in the visual awareness of the participants.

Subcategories:

> Recognizing a symbol;

> Known abstract image;

Concrete image.

Examples:

Recognizing a symbol: “The task was very easy. All the shapes formed a spiral, which is a familiar shape. And it was therefore easy to remember.” (VR.WM.1.05-02-P-01)

Recognizing a symbol: “I recognized a shape in it. It was not perfectly symmetrical. It was a trapezoid. Like looking at longer lines from above.” (VR.WM.1.02-P-01)

Known abstract image: “This was not something visual. It was not as if I said to myself [brand name] ice cream in order to see this. It was more of a memory [pause] on this level of [pause] immaterialness. It was intangible. It is more of a stereotypical feeling. It elicits certain feelings. If it is like a German flag, it elicits some feelings related to World War II. But it is not as if I was actually imagining this. These are just feelings. If I imagine an ice cream, there are these intangible feelings of calmness.” (VR.WM.1.15.04.C.01)

Known abstract image: “As soon as I looked at the coors, I started to connect them with associations. I thought of a flag of a country. And this association was not verbalized. It was a fleeting thought.” (VR.WM.1.25-03-C-02)

Concrete image: “I realized that the shapes are like police lollies. I saw an image of a cop. It was a very clear image. It wasn’t on the screen. It was somewhere behind my field of vision and I could still see the screen in front of me. It was very clearly visual. It took the form of a mental image first. And then I kind of extended the image. It was there all the time, but then it moved from the back of my awareness to the front and this made the image clearer. This was happening to me.” (VR.WM.1.03-0-01)

Concrete image: “I realized that the image had the shape of a kite. And this kite awoken certain feelings in me. But these feelings weren’t too intense. Without these feelings I could not remember this association. I saw a blue sky and an orange kite. The orange was very concrete and the blue was more in the background. The kite was on the screen. The screen beyond the kite had depth. So, the kite was located in space, but this depth of space added no extra meaning. It did not help me in any way. Nor was it constant in my experience [...] Now, when you ask me about the depth, it suddenly attains it, whereas before, the depth was not there.” (VR.WM.1.14-04-P-02)

Considerations: Recognizing a pattern is different from the experiential category searching in that the former is a passive process (i.e., it happens to the participants): the stimulus is tied to a pattern that may elicit a concrete mental image. Conversely, searching is a process of active examination of the stimulus which does not necessarily lead to a recognition of a pattern.

3.1.1.2.3 Acoustic (level II and I category)

Description: Acoustic is an aspect of experience whereby the appearance of a stimulus or an object in a stimulus is accompanied by a mental sound, intimately tied with the moment of appearance itself.

This sound is not necessarily clearly articulated. It can be a kind of mumbling, counting, construction of a dancing rhythm, interjections or even physiologically impossible sounds.

Commonly, these sounds construct rhythm, which subsequently colors the entire atmosphere of experience. In some cases, the acoustic is so strong and clearly tied to the appearance of the objects that it is sufficient for discovering whether the test stimulus is identical to or different from the memory stimulus.

Values: None.

Examples:

> "While I was looking at the screen I was creating a sound: 'hmph, hmph, hmph, hmph.' And I was counting whenever the stimuli appeared as well. This counting was done in my regular inner voice. This sound though wasn't always the same. And they were not necessarily tied to the location of the objects. It was as if this four-level scale emerged and it remained the same, regardless of what was happening in the example." (VR.WM.1.10-02-P-01)

Considerations: Acoustics are different from rhythm even though these categories are clearly related. It is possible that the two appear in the participants' experience independently of each other, even though the most common structure of experience is the initial appearance of the acoustic, followed by the construction of the rhythm.

Rhythm itself is not tied merely to the acoustic, auditory experience, but can constitute itself through bodily feelings as well.

3.1.2 Maintaining (level IV category)

Description: *Maintaining* refers to the experience of holding on to a memory of a stimulus during a visual-spatial working memory task. *Maintaining* as a category is defined by the temporally delineated phase of the visual-spatial working memory task; i.e., by the duration of the delay phase.

Subcategories:

Active maintaining;

Passive maintaining.

3.1.2.1 Active maintaining (level III category)

Description: *Active maintaining* is an experiential category that describes a strategic attitude towards the task (i.e., the participants actively go about solving the task) in which they attempt to hold on to the memory stimulus during the delay period. By the term *active*, we mean that participant experience the maintenance of the initial stimulus as something that they do, rather than something happening to them.

Subcategories:

Rehearsing;

Imagining;

Waiting.

3.1.2.1.1 Rehearsing (level II category)

Description: *Rehearsing* is an experiential category that comprises two different attitudes towards memorizing of the memory stimulus. If the participant memorized the memory stimulus via describing, repeating refers to articulating the description of the memory stimulus during the delay period. If the participant memorized the memory stimulus with a motor strategy, then repeating refers to the constant repetition of the pattern of eye-movements during the delay period.

Values:

Rehearsing verbal description;

Rehearsing eye-movements.

Examples:

Rehearsing verbal description**:** "During the delay period, I once again said the names for the two colors that I set out to memorize. So, I was repeating 'blue, black, blue, black.'" (VR.WM.1.04-01-C-02)

Rehearsing verbal description: "I was focusing on the object that was a kind of an outlier. I couldn't memorize it neatly. So I was repeating the description for the outlier. I was saying to myself,: 'Yellow, upper right.'" (VR.WM.1.05-02-O-03)

Rehearsing eye-movements: "During the delay period, I was reiterating the connections between the objects. I had a feeling that the delay was very brief. That I don't have a lot of time. It felt as if the test stimulus was coming at me really quickly. This made me kind of anxious about the possibility that I forgot the memory stimulus, so I was wildly moving my eyes around the lines that connected the objects." (VR.WM.1.05-02-O-03)

Rehearsing eye-movements: "I repeated the pattern. I moved my eyes. I moved them to the point where the objects were previously located. The objects leave behind these little space. The experience was very raw. I didn't say anything. I do feel though that they leave a sound. It's as if a hear a rhythm of a sort. I shift my gaze to these little spaces." (VR.WM.1.06-02-P-02)

3.1.2.1.2 Imagining (level II category)

Considerations: Category imagining as a subcategory of maintaining is equal to the category imagining as a subcategory of memorizing described above.

3.1.2.1.3 Waiting (level II category)

Description: Waiting refers to the experience of the delay period that is not occupied with strategic maintenance of memory stimulus, nor is it filled with mind-wandering. Waiting refers to two types of

experiences during the delay period: void and leaning forward. Void refers to the situations where it seems as if nothing was going on during the delay period; i.e., the participants did not experience any mental content. In most of these cases, the participants are not even aware of the delay period as such entering their awareness. Leaning forward refers to waiting that is filled with expectation. Importantly, leaning forward is not a passive experience, but is accompanied by a sense of action (i.e., the participants experience that in their expectation, they are performing a mental gesture towards the future moment). For the most part, leaning forward refers to the expectation of the test stimulus but it may also refer to the moment when the participants will be prompted to report on their experience.

Values:

Void;

Leaning forward.

Examples:

Void: "By and large the pause is beyond my experience. It is as if I was experiencing a kind of leap. There was simply nothing going on." (VR.WM.1.10-03-O-01)

Void: "Nothing much is going on during the break. I just freeze. It passes by very quickly and I try to not do anything so that I don't lose the memory stimulus." (VR.WM.1.06-01-O-02)

Leaning forward: "The delay was not just waiting. I experienced a kind of flow. Tied to it was a sort of anticipation. It was like when you throw a ball down the stairs, and you know there are levels where it stops for a moment, but it will go on because the ball has a momentum of its own.2 (VR.WM.1.14-03-O-01)

Leaning forward: "During the pause, I was focusing on not thinking about other things. I was just ready for something to appear. I experienced it as a kind of focus in my eyes. This tension. I recognize this tension as attention." (VR.WM.1.14-03-O-01)

3.1.2.2 Passive maintaining (level III category)

Description: *Passive maintaining* is an experiential category that describes a serendipitous attitude towards the task (i.e., task-performance is something that they are passively involved with) in which the participants attempt to hold on to the initial stimulus. By the term *passive*, we mean that participant experience the holding on of the initial stimulus as something that happens to them, rather than something they do.

Subcategories:

Impression;

Afterimage.

3.1.2.2.1 Impression (level II and I category)

Description: Impression is a subtle experience of an echo that remains after the stimulus disappears. Importantly, this aspect of experience is not visual; it is almost-visual. Many participants find the following metaphor useful as a description of this experience: impression is qualitatively similar to touching one's own hand, and after the touch, still being aware of the point of contact. It is a very raw experience in the sense that it fades very quickly and carrying a very limited amount of information (specifically, it entails the datum *something was there*). Sometimes, this feeling is so subtle as to be strategically useless. At different times, it may be so clear that it can be unfolded and based on it, it becomes possible to figure out the identity of the memory stimulus.

Values: None.

Examples:

> "It is a very light feeling of knowing. Like a small touch that makes me know where something was." (VR.WM.1.29-01-O-01)
>
> "This was not the echo of the object or an image, but rather of movement. It feels as if the movement continues without moving your eyes. The movement itself; however, is tied to a single spot. It feels like movement that stays on the screen." (VR.WM.1.29-03-O-02)
>
> "During the delay period I have this vague image. I didn't know what the colors were. But I did have an image of the color, like a feeling at the back of my mind about how the colors are. But they were not projected. I held on to the image of the square in which the colors appear, and then behind the eyes, I could sense these colorless circles." (VR.WM.1.16-03-C-01)
>
> "The memory stimulus had a temporal component in terms of how it was presented. So the dots left an echo behind them. This echo slowly faded away. I could then connect these echoes with each other. I created a path. But not from the objects. Based off of this feeling I gained from my memory." (VR.WM.1.05-02-P-02)
>
> During the delay, I spatially moved through the dots. This experience was not at all visual [...] Actually, I didn't even have any representation about these dots. I was not creating a kind of wholesome representation. I separately looked at each dot, which somehow created this feeling in my mind that there is a dot on that spot. This feeling was just that spatially, the dost were placed there. They got added to my mental space. What it was was that I felt their location. (VR.WM.06-04-P-02)

Considerations: Impression is not a form of imagining. While imagining is a concretely visual experience, impression does not have a visual component. It is almost-visual. Most commonly, this feeling is experienced when participants are moving their eyes from one object to the other, after the stimulus has disappeared. Consider the following example:

> "I moved my eyes, first, along the upper two symbols and then I repeated the same motion during the delay period. At this point, I was surprised at how the third symbol suddenly became apparent to me, and I could finish it by moving my eyes. The third symbol had this sense of impression. Not the first two. The third one did." (VR.WM.1.03-O-03).

Because of this experiential connection between motor behavior and impression, we suspect that the two constitute a related phenomenon.

3.1.2.2.2 Afterimage (level II and I category)

Description: Afterimage refers to the experiential correlate of a physiological (i.e., entoptic) process whereby participants experience a trace of the object that was previously held in the front of awareness (typically, in the form of an echo in the complementary color scheme). These afterimages cannot be dismissed by simply moving the attention away from that. Consequently, many participants find the afterimages disrupting their task-performance. On the other hand, some of the participants may use the afterimages strategically to help them with successful task-performance.

Values: None.

Examples:

> "Afterimages are something strictly visual. They appear when the stimulus is gone and afterwards their color fades away quickly. I got confused because they appeared after the test stimulus and persisted until the memory stimulus of the next example. [...] The afterimages are something that happens to me. If they weren't, I would use them to my advantage." (VR.WM.1.04-01-O-01)

> "When I see the memory stimulus, if I blink while scanning it, I can create an afterimage. And so I can extend the whole first stimulus into the delay period." (VR.WM.1.14-01-O-03)

Considerations: The experiential category afterimage must be strictly differentiated from impression. Specifically, the former describes a visual experience, whereas the latter does not describe a visual experience, but rather a raw experience of the location of the previous stimulus that is experienced *as if seen.*

3.1.3 Recalling (level IV category)

Description: *Recalling* refers to the experience of memorizing a stimulus during a visual-spatial working memory task. *Recalling* as a category is defined by the temporally delineated phase of the visual-spatial working memory task; i.e., by the duration of the test stimulus.

Subcategories:

Active recalling;

Passive recalling.

3.1.3.1 Active recalling (level III category)

Description: *Active recalling* is an experiential category that describes a strategic attitude towards the task (i.e., the participants actively go about solving the task) in which they attempt to recognize the test stimulus as equal or different to the memory stimulus. By the term *active*, we mean that participant experience the memorization of the initial stimulus as something that they do, rather than something happening to them.

Subcategories:

Comparing;

Guessing;

Mixed recalling.

3.1.3.1.1 Comparing (level II category)

Description: *Comparing* is an experiential category that describes a mental action whereby the test stimulus is actively compared to some form of representation of the memory stimulus with the goal of identifying as equal or different. By the term *active*, we mean that participant experience the memorization of the initial stimulus as something that they do, rather than something happening to them.

Subcategories:

Comparing with a mental image;

Applying eye-movements;

Applying verbal description.

3.1.3.1.2 Comparison with a mental image (level I category)

Description: The participants maintain some form of visual representation of the memory stimulus, which they then juxtapose with the test stimulus. As long as the representation and the test stimulus match, the participants know that the two stimuli are the same. Conversely, if the two do not match, then they know that the stimuli are different. Comparison with an object in mental space is an active experience, accompanied by reasoning about the stimuli. Primarily, the participants are looking for a divergence rather than compatibility between the stimuli.

The participants frequently do not compare the objects themselves but the constructs created to memorize the memory stimulus (e.g., path, line, afterimage, visual feeling). A participant, for example, reports that if the sequence of objects in a stimulus were to be replaced and not their location, she would not be able to recognize the difference.

Examples:

> “This new thing filled in the thing that happened before. I was not aware of both of them at the same time, but I recognized the second one as different in relation to what was in its place before. [...] It felt as if there was the same object there, but it was colored differently. I recognized the second one from its relationship to the first one.” (VR.WM.1.22-04-C-04)

> “I experienced a kind of overlap. While comparing them, I took the memory stimulus in my mind and there, I placed one image on top of the other. One image was like the thing I was seeing on screen and the other one was like the memory of the first stimulus and so it was more condensed. (VR.WM.1.05-01-C-01)

3.1.3.1.3 Applying eye-movement (level I category)

Description: During the test stimulus, the participants shift their eyes to the location where they expect the appearance of the next object. If the object is not there and they have to shift their eyes someplace else, they know that the test stimulus is different from the memory stimulus. With each subsequent matching object, there is a sense of rising continuity, which forms a kind of affirmatory affect. If an object does not match, the eye-movement is typically accompanied by a somewhat more intellectual examination: a re-examination of where the next object should be located.

Values: None.

Examples:

> "I was looking to see whether I make the same movement during the test stimulus as I did during the memory stimulus. Unlike before, I felt a kind of pleasure at the fact that they matched. It was not just something that exists, but it was something that was good." (VR.WM.1.15-04-O-02)

> "During the test stimulus, I was again moved from these little spatial feelings left behind by the memory stimulus. If I had to move into a different direction because of the object on the screen, I knew that the test stimulus was wrong." (VR.WM.1.06-04-P-03)

3.1.3.1.4 Applying verbal descriptions (level I category)

Description: When applying verbal description, participants engage in an active form of recalling, whereby they examine the identity of the test stimulus by naming it according to how they named the memory stimuli. If the descriptions match the test stimulus, they know it to be identical to the memory stimulus.

Values: None.

Examples:

> "I detected the sameness when the tails of the objects matched what I was saying in my head. I was naming the descriptions of orientations from the memory stimulus. And they were always the same words: 'up, down, zig zag.'" (VR.WM.1.17-03-O-01)

> "I repeated the words and looked whether their content matches the things I see on the screen." (VR.WM.1.24-04-O-02)

3.1.3.1.5 Guessing (level II and I category)

Description: Participant decide about their answer regarding the identity of the test stimulus in a completely uninformed manner. While doing so, they may experience anything from carelessness, because they simply do not care about the task, to unease, because they are performing poorly at the task. The guessing itself is typically experienced as oscillating between the two answers without finding any support for either of them in their experience.

Guessing also encompasses the task-performance in which the participants are performing at solving the task. In some situations, the task structure completely falls apart, and the participants are no longer aware whether they are observing memory stimulus or test stimulus. In consequence, they engage with the task simply because they have to.

Values: None.

Examples:

> "When I pressed the key to respond, I experienced an unease. It was related to me not being sure. Actually, I was guessing when I answered. There was no gut feeling. I just experienced a void. [...] This guessing was something like a gentle leaning from left to right key. [...] There was a lot of emptiness in my experience. There was no feeling that I could use to get the right answer." (VR.WM.1.19-03-C-02)

> "I mean, there was some reason why I answered the way I answered, but mostly, it was guessing. I realized at some point that I mostly just press the left key and I don't even decide about my answers." (VR.WM.1.25-03-O-02)

3.1.3.1.6 Mixed recalling (level II and I category)

Description: Mixed recalling refers to the situations in which the participants use two or more strategies to decide whether the test stimulus is identical to or different from the memory stimulus. While there is a certain level of variability to mixed recognition, it mostly describes the situations where participants first passively recognize the identity of the test stimulus, and then - because they do not trust this way of task-performance due to a lack of explicit mental gestures - employ a concrete gesture to make sure of their answer.

Values: None.

Examples:

> "During the test stimulus, I was still seeing the tails of the objects, but they were paler than during the memory stimulus. [...] I could again establish the connection between myself and the objects. Colors and lines were still present but they were less intense. I was checking the tails. In what direction they were pointing. But finally, I decided based on my gut feeling. This was something bodily. A pleasant, calm feeling. It was very mild." (VR.WM.1.19-03-O-02)

> "My comparison was a little bit verbal and a little bit visual. First, there was a feeling that the image is the same. And then the words happened and I checked again whether that was true." (VR.WM.1.25-02-O-01)

3.1.3.2 Passive recalling (level III category)

Description: *Passive recalling* is an experiential category that describes a serendipitious relaization about wether the test stimulus is equal or different to the memory stimulus. The participants. By the

term *passive* , we mean that participant experience of recognizing a stimulus is something that happens to the participants, rather than something that they do.

Subcategories:

Appearing;

Hunch.

3.1.3.2.1 Appearing (level II and I category)

Description: This is an exceedingly passive experience whereby the test stimulus simply appears as being identical to or different from the memory stimulus. Participants simply know whether the stimulus is the same or not. Something in participants' experience informs them about the nature of the stimulus. The participants typically do not attend to individual objects within the stimulus, but the stimulus appears to them as identical to or different from the memory stimulus. The feelings of similarity and difference accompanying this experience are fairly subtle and participants typically have a hard time accessing them. Participants are able to detect the difference between the stimuli even before they look at individual objects, or before they actively compare the stimulus to an explicit mnemonic representation. Even when a difference is detected, in most cases, participants are unable to pinpoint what exact object is different.

Accompanying the experience of stimulus appearing as identical/different can be a mild, almost imperceptible sense of pleasure. Insofar as the participants are not focusing on the objects of their visual perception, but, for instance, the rhythm of solving the task, it is the rhythm itself that can be experienced as equal to or different from the memory stimulus. Cooccurring with equality, participants can experience the experiential category of positive neutrality, a sense of some sort of balance being maintained.

When participants employ grouping as a strategy of reducing complexity in order to differentiated between the in-group and out-group objects, they commonly rely on passive perception of difference. They are usually convinced that providing the difference between the memory stimulus and the test stimulus being large enough, the unattended group of objects (the out-group) will appear to them as different.

Values: None.

Examples:

> "I didn't remember them, but I did recognize them. It was as if when you are drawing and everything seems clear to you, and then somebody new looks at the painting, and they immediately see what's wrong. Well, in a similar way, I recognized the difference here. [...] It was so very obvious whether it's same or different that I didn't have to go looking into my memory for the answer.2 (VR.WM.1.15-04-C-01)

> "This second thing [the test stimulus] is a different process. There's less work to it. The first one you have to *memorize*. In the second one, you're just checking the fit of the model. It's a completely different process. You don't have to look for relations, except for the relation between the first and the second stimulus. And this relation is basically just some feeling. [...] It is in large part tied to whether I recognize the Gestalt. I look whether the second stimulus

> corresponds to this sense of whole that I got before. [...] If it's wrong, it's usually wrong right away. If it's correct, then I have to compare it. Look for a flaw. If I detect that there is a flow, I immediately know that it's not correct. And I immediately respond that it isn't." (VR.WM.1.07-01-O-03)

> "When it was wrong, I knew right away that it is wrong and I had no doubt about it. When it was correct, I had to reassure myself that I read it correctly." (VR.WM.1.13-02-P-01)

Considerations: The central difference between the appeaing and hunch is that the former is accompanied by a relatively high level of certainty in the answer. While both categories share the lack of a sense that the participants performed a mental gesture of comparison, there is a difference in how stable the answer appears to be in their consciousness.

3.1.3.2.2 Hunch (level II and I category)

Description: Hunch is an experiential category that describes passive recognition of the test stimulus (i.e., it is an experience that happens to the participants, rather than performed by them). Hunch refers to a difficult to describe and difficult to access feeling that is commonly related to a change in bodily awareness. This feeling (often described as *gut feeling*), is fairly uncertain and commonly leads to a re-checking of the test stimulus (mixed recognition).

Subcategories: None.

Examples:

> "During the test stimulus I simply knew what the answer was. When something did not match this [global feeling] I received a signal in the sense that something did not seem as it should be. Something tingled in my belly. And when something was different, the feelings was consistently like that." (VR.WM.1.06-C-02)

> "I decided about the answer based on this feeling. This was an intertwining of some sort of attention and a feeling in my chest, based on which I make a decision about whether it is right or wrong. However, I don't know whether it is right or wrong. I don't know *rationally.* I simply know *bodily."* (VR.WM.1.19-01-0-01)

Considerations: Unlike the stimulus appearing identical/different, hunch is accompanied by a large degree of uncertainty and doubt. Participants do not trust this feeling. The difference is illustrated by the following example:

> "I don't go looking around my consciousness, but I just make a rapid decision. I wouldn't stand behind this feeling. I don't know for sure." (VR.WM.1.07-01-C-02)

3.1.4 Mind-wandering (level IV - I category)

Description: Mind wandering describes situations in which participants are not focusing on the task at hand but the content of their thoughts. It commonly amounts to thinking about things that are not explicitly related to the task at hand. This is either thinking about completely unrelated matters, or

about how the participants will be reporting on their experience. The latter therefore describes the awareness of and dealing with demand characteristics; i.e., in what way do the properties of the research situation enter participants' awareness.

Examples:

> "I wasn't even focused on the things I should be seeing in front of me. I was totally absorbed in my thoughts." (VR.WN.1.06-04-P-01)

> "The task seems somewhat more difficult. Mostly because I have to constantly push away some things. Some thoughts. These thoughts are not related to the task itself. Rather, they come into being on their own. I don't have a feeling that I am the one shaping them or constructing them. It takes a lot of energy to push them away." (VR.WM.10-01-O-01)

3.1.5 Meta-cognition (level IV category)

Description: *Meta-cognition* describes cognition about cognition. In these experiences, the participants became aware of their own task-performance.

Subcategories:

Explicit;

Implicit.

3.1.5.1 Explicit (meta-cognition) (level III category)

Description: *Explicit (meta-cognition)* is an experiential category wherein aprticipants actively engaged in monitoring of their task perfromance.

Subcategories:

Commenting;

Attention;

Monitoring.

3.1.5.1.1 Commenting (level II and I category)

Description: Commenting is a form of metacognitive oversight whereby the participants make use of an ongoing commentary to monitor have well they are performing the task.

Examples:

> "While I was solving the task, I experience a kind of dialogue. At some point this thinking happened, which was in large part a commentary on how I was performing the task. It was a

kind of development of strategy. Because of that I could not focus on the task and I had to do away with these thoughts by starting to talk about something else." (VR.WM.1.01-0-02)

"For the whole time, I could hear this voice, this emotionally charged voice that accompanied my task-performance. In actuality, I was constantly talking to myself about what I am doing or what I should be doing [...] During the test stimulus, I said to myself, `It is different, but I'm not sure whether it's right." This monologue took the form of words. And they were similar to the comments I otherwise make on how I am solving the task or what is going on. Except that before they were accompanied by this sense of confidence, whereas now they came with a sense of defeat." (VR.WM.1.16-03-O-01)

3.1.5.1.2 Attention (level II and I category)

Description: Attention is a kind of metacognitive oversight in which the participants feel that they are performing well, because they know that they are paying attention to the task without allowing any disturbances (in the form of wandering/thinking to enter their experience). Further, this category refers to the awareness about performing the task poorly by not being focused on the task and dealing with other aspects of their experience.

Values: None.

Examples:

"I know I am doing well because I am paying attention to the exercise. Even if the task is simple, I know I have to pay attention to it and so I know I am doing it well." (VR.WM.1.04-P-01)

"I think I did worse than in previous examples. I was trying to focus on the right half of the stimulus, but I sort of failed to do so. My mind was wandering and it became harder to know what is going on. I think I did worse. I am not sure in my responses." (VR.WM.1.25-04-O-03)

3.1.5.1.3 Monitoring (level II and I category)

Description: Monitoring is the experience whereby participants are actively paying attention to their experience both in order to perform the change-detection task better, and to be able to provide a better subjective report.

Monitoring: "I think that the main difference was that I was aware of the more subtle things in my experience and so I was able to look at the task differently. Yeah. It was a particular kind of attending. And the strategies remained the same." (VR.WM.1.23-02-C-02)

Monitoring: "I wanted to give you the best answers I can. And so during the delay, I was planning out the things I was gonna tell you. I wanted to create good data for you." (VR.WM.1.15-03-C-01)

3.1.5.2 Implicit (meta-cognition) (level III category)

Description: *Implicit (meta-cognition)* is an experiential category wherein participants experience a vague bodily awareness that they could – if attended to – appraise as an awareness of the quality of their own perfromance.

Subcategories:

Meta-cognitive feeling

3.1.5.2.1 Meta-cognitive feeling (level II and I category)

Description: Metacognitive feeling is an experiential category that refers to participants being implicitly aware that they are successful at performing the task and memorizing the stimuli. This feeling is by and large related to the atmosphere of positive neutrality: i.e., it amounts to task-performance characterized by an absence of disturbances. Unless disrupted, this feeling is by and large not brought to the front of awareness.

Values: None.

Examples:

> "I had a feeling that I successfully memorized the memory stimulus. This feeling was about me not needing any contemplation or searching for alternatives for any of the steps of the task. During the delay, I knew exactly where I have to look, and what happened after my gaze had been done was not subjected to any sort of doubt." (VR.WM.1.14-04-P-01)

> "I was not sure. I was creating the same path, but one of the objects may have been one centimeter more to the right. This didn't spoil the overall shape. I detected this only through the absence of completely being sure about it. I detected in the doubt. This doubt stopped me for a little bit. I disrupted my rhythm." (VR.WM.1.04-02-P-02)

Considerations: Metacognitive feeling is an experiential category that is similar to the background feeling of the smoothness of performance. We believe that it is in fact a very similar kind of experiential landscape: it is both the experience of positive neutrality; however, thematized in a different way. Metacognitive feeling is thematized as awareness of the quality of one's own actions rather than the awareness of the stability of the task itself.

3.2 Background feelings (level V category)

Description: Background feelings refer to those aspects of experience that are not in the forefront of one's awareness but rather constitute a kind of an atmosphere or a frame of experience. In other words, it refers to those aspects of experience that can be different while the object in the front of awareness remains the same. It is only the coloration, the sense of the experience that changes. Roughly-speaking, we can equate this experiential category with the theoretical construct of *existential feelings* (cf. Ratcliff, 2008).

Subcategories:

Overarching states of mind;

Atmosphere of experience;

Attitude towards the task;

3.2.1 Overarching states of mind (level IV category)

Description: *Overarching states of mind* refer to the experiential category whereby an overall attentional attitude organizes the specific cognitive functions involved.

Subcategories:

Attentional dispositions.

3.2.1.1 Attentional dispositions (level III category)

Description: Attentional dispositions are a background feeling that refers to the way in which we turn our attention towards an object in the world. How we pay attention to it, in turn, determines in what way we experience the object itself. In regard to the visual-spatial working memory task, we constructed five ways in which our participants attended towards the stimuli.

Subcategories:

Separation from the task;

Coupling with the task;

The task to you;

You towards the task;

Not on the task.

Considerations: The expression attentional dispositions itself is derived from the literature (Petitmengin et al., 2009: Kordeš et al., 2019). In this research, the experiential category was established before we were familiar with this concept. In particular, it was coded as two separate categories: connection with the task and direction of attention. When we became aware of the concept of attentional dispositions, we collated the two categories into a single experiential category.

3.2.1.1.1 You towards the task (level II and I category)

Description: You towards the task is an experiential category describing an attentional dispositions whereby the participants have the feeling that they are performing mental gestures on the task.

Sometimes, this feeling can be soc lear, that it is accompanied by a spatial feeling of one's experience being directed towards the task.

Values: None.

Examples:

> "The way I solve the task seems more aggressive. I don't let it come to me. I am the one who reaches out and I force myself onto the task." (VR.WM.1.29-02-P-03)

> "The task seemed more difficult than before. Before, it was enough for me to just have the impression of the image, whereas now I had to keep more of my focus on the task. I had to push away the things unrelated to the task. I performed movements with my gate from object to object. I was jumping across them." (VR.WM.1.10-01-C-06)

3.2.1.1.2 The task to you (level II and I category)

Description: The task to you is an experiential category whereby the participants let the task come to them in their attention; i.e., they are receptive to the stimulus. They allow it to appear to them such as it is without performing any explicit mental gestures on them.

Values: None.

Examples:

> "I just let the dots happen [...] I trusted myself that I will be able to know whether the next stimulus is correct or not. [...] It was as if I shut the task down. I was looking at the [fixation cross] and it was a little bit like meditating. I didn't have to think of the dots or anything." (VR.WM.1.15-02-P-04)

> "It was not as if I performed an action into the world. The world imprinted into me. I held it with my attention and then something came from the outside in. It lodged inside of me. Into my chest. As if there was something gently percolating there." (VR.WM.1.19-05-C-02)

3.2.1.1.3 Coupling with the task (level II and I category)

Description: Coupling with the tas is an attentional disposition whereby the participants have a feeling that there is a connection established between them and the stimulus. They feel as if nothing besides them and the task exists. Often, the sense of the environment (including the interviewer) disappears, as well as the feeling of their own embodiment.

Values: None.

Examples:

> "It is a kind of bubble. My attention is concentrated here at the front, at the task. This bubble is essentially a very pleasant kind of attention. I really like this focus. And my surroundings disappear. And I'm not aware of you either. There wasn't even an awareness of where I'm

> sitting. All that existed was this task. and I felt warm about that. This warmth was for me and for the way I do the task. It wasn't warm like the sun, but like a warmth in my thoughts." (VR.WM.1.21-01-P-02)

> "I was no longer even paying attention to my surroundings. I was completely focused on the task and all that was present was the rhythm of solving the task. I had no thoughts. All there was was the solving of the task." (VR.WM.1.24-01-P-02)

3.2.1.1.4 Separation from the task (level II and I category)

Description: Separation from the task is an attentional disposition in which the participants are experiencing a certain measure of distance from the task. They lose the sense that they can exert causal influence over the task, it seems to them as if they are watching a video recording.

Values: None.

Examples:

> "I don't know. Something about these dots confused me and I stopped paying attention to the task. It was as if in that moment, I memorized the stimulus and then immediately forgot it. I clicked already during the delay as if it were wrong." (VR.WM.1.02-01-O-03)

> "I wasn't really with the task. I was solving it and it was important to me to solve it well, but I was also really aware of the room and you sitting behind me [...] What happened was that there was this sense of whole that was leftover from the previous example, and then I didn't know whether I'm comparing it to this example or the one that came before. I was thinking about that." (VR.WM.1.27-04-O-01)

3.2.1.1.5 Off-task (level II and I category)

Description: *Off-task* is an attentional disposition that refers to situations in which the participants stop performing the task and start to pay attention to task-unrelated things.

Values: None.

Example:

> "I was kind of bad at solving the task. I was wondering whether the stimuli are even different and how much they can change and if there's maybe only one in four that changes. I was still aware of the stimuli. I experienced them. But I didn't deal with them." (VR.WM.1.10-01-O-02)

3.2.2 Attitude towards the task (level IV category)

Description: When talking about the attitude towards the task, we are talking about a background feeling within which the participants implicitly interpret the experimental paradigm in one way or another. In this context, we use the term "interpretation" to refer to what the participants make of the

whole research setting. This interpretation is implicit, and unless reflected on, the participants are not aware of it.

Subcategories:

Difficulty;

Attitude towards the task-performance;

Engagement with the task.

3.2.2.1 Difficulty (level III category)

Description: Difficulty is an attitude towards the task in which the participants interpret the task either as easy or difficult. Importantly, it is apparent that the difficulty of the task is not a property of an observer-independent, objective world, but it amounts to an attitude that participants lean into.

Values:

Difficult task;

Simple task.

Examples:

<u>Difficult task:</u> "During the test stimulus, I said to myself, `It is different, but I'm not sure whether it's right." This monologue took the form of words. And they were similar to the comments I otherwise make on how I am solving the task or what is going on. Except that before they were accompanied by this sense of confidence, whereas now they came with a sense of defeat." (VR.WM.1.16-03-O-01)

<u>Difficult task:</u> "I was uncomfortable. It almost felt like panic. And it got worse whenever I failed. I wanted to cry. I had a feeling of helplessness. I felt anxious. It was this tightness rising upwards from my chest. It was rising all the way to my collarbones, but no higher than that. I felt how my heartbeat increased."

<u>Simple task:</u> "I still tried to burn the image into my mind, but to a lesser extent. I was more lighthearted in my answers. My attention wasn't as strong. There wasn't that focus where you go into the screen. It was a very *flowy* attention. I just sort of stumbled into it." (VR.WM.1.19-01-C-02)

3.2.2.2 Attitude towards task-performance (level III category)

Description: Attitude towards the task-performance is an experiential category within which the participants may interpret their own task-performance either as something fun and playful (or maybe as a challenge if the task is difficult), or, on the other hand, as a test or an exam.

Values:

Playful;

Task-like.

Examples:

Playful: “The task seems more dynamic somehow. It was easier and more fun. I didn’t know where the objects will appear and so there was always a little surprise when they showed up. I didn’t have an anticipatory feeling about where they will appear. It was fun.” (VR.WM.1.06-02-P-01)

Playful: “It was important to me that I solve the task well, but not to a large extent. I didn’t perceive it to be really bad if I didn’t solve it in the right. The task was playful. There was a pleasure to it. Rhythm and path were light. It wasn’t difficult. There was no fear or anxiety. It was more that I was interested in what will happen. There was no pressure that I might forget.” (VR.WM.1.19-04-P-01)

Playful: “I was really tired and my attention dropped a bit. And then I saw that the pattern was actually really easy and I became happy about that. It was as if my eyes became more lazy. As if my attention became lighter.” (VR.WM.1.14-01-O-05)

Task-like: “I began thinking about how I don’t like the exercise. The tails of the objects bothered me. They caused these bad feelings inside of me. These were more mental than bodily. It wasn’t as if I was in pain or under stress. It was more of a preference. As if I didn't have these tails on the objects.” (VR.WM.1.25-03-O-02)

Task-like: “At the beginning, I hated the task. I experienced this small memory from an event at the uni. It let me know that I need to be more humble in the way I solve the task, otherwise, a defeat will follow.” (VR.WM.1.04-02-P-02)

3.2.2.3 Engagement with the task (level III category)

Description: Engagement with the task is an experiential category that describes the level of enthusiasm and zeal with which the participants approach. In other words, it is a question of whether the participants approach the task as if it is important and something they have to do well, or whether they approach it as something irrelevant, something that is simply there.

Values:

Engaged:

Competitive attitude;

Vain attitude

Obligation;

Disengaged:

Disregard,

Unburdedness;

Boredom.

Examples:

Competitive attitude: "I feel a sort of competitiveness. It's something in my attention and my body. As if I am ready to perform. I can feel it in my chest and my heart." (VR.WM.1.07-01-C-02)

Competitive attitude: "I am doing well. It is important to me that I am doing well. I don't know. It feels like I am competing with somebody. Maybe I am competing with myself or maybe I am competing with the computer. In any case, it's important that I'm doing well." (VR.WM.1.24-01-C-01)

Vain attitude: "I have to do this. It's not as if there are some positive or negative consequences if I succeed or fail. It's a challenge. I accepted the challenge. So, now it becomes the question of whether my capabilities - mental capabilities are up to par. Whether I can do it. I'm curious about that. I'm curious about whether I can do it." (VR.WM.1.27-04-O-01)

Vain attitude: "While I was solving it, I had this thought, '[expletive] what if this feeling that I am doing well is wrong?' I felt panicky about that. About whether the image was really as diagonal. It was a relatively small thought. It didn't overtake my experience. I still just glanced at the stimulus and I didn't really pay much attention to it." (VR.WM.1.25-01-0-01)

Obligation: "It seems to me that I feel that this is a task that I have to perform. I feel some sort of drive to perform it. It's a kind of tension. I am doing my best to memorize it. That I solve it well. So, I put a lot of time into getting it right. This tension is present throughout. In my body. In my shoulders. I can feel that I am constantly ready for something. For performing the task. For answering." (VR.WM.1.01-01-O-02)

Obligation: "I woke up from snoozing. It felt uncomfortable. As if I knew I can't fall asleep, and yet, i am still sleeping. There was an imperative to me solving the task. Even though I wasn't actually solving it, I experienced some sort of *I must.* It's a kind of presence. I didn't create it. Well, maybe at some point, I maintained it. First it came from the outside and then I continued it. (VR.WM.1.25-03-O-03)

Disregard: "It seemed to me like it should be fun, but it isn't. Now, it's stressful. I am using only about 20% to pay attention to it and I kind of don't care about it. I started to think about the task and now it's no longer as fun. And everything began going by so quickly. And so, I started thinking, why should I even bother if everything is going by so quickly. And then I ask myself, what's wrong with me? Am I dumb? And so I just refuse to answer." (VR.WM.1.27-04-O-02)

Disregard: "I couldn't even see the screen. I turned off. I wasn't present. I couldn't see the objects. I could still feel some sort of rhythm, but this wasn't enough for me to be able to solve the task [...] The task is completely beyond my experience. I remember noticing that at some point the [fixation cross] turns dark, and that was the only time I was paying attention to the task for even a little bit." (VR.WM.1.25-02-P-05)

Unburdedness: “The task was boring and difficult. I knew I have to pay attention to it, otherwise I won’t be able to solve it correctly, but at the same time, I was also aware that everything’s alright even if I make a mistake.” (VR.WM.1.14-01-O-01)

Unburdedness: “I was thinking about how nobody’s even going to look at these results. And that made me much more relaxed. I was no longer thinking about whether I am solving it right or wrong. This feeling was there from the very beginning. It wasn’t really bodily. It was just a thought.” (VR.WM.1.25-02-O-01)

Boredom: “I was confident but in a cool sort of way. As if I am no longer really tired. I got used to the whole process. I know what to expect and then I was no longer working at the task. It was really clear during the test stimulus, because I no longer tried to find this sense of validation. I no longer care whether I’m wrong.” (VR.WM.1.05-01-C-04)

Boredom: “I kind of got sick of it. My attention started to wane. I experienced this tiredness in two parts. One was this end to trying my best. The other was wandering of my attention. I still gave informed answers, only now, there was a greater sense of guessing because I no longer tried to maintain representations during the pause.” (VR.WM.1.10-01-O-02)

3.2.3 The atmosphere of experience (level IV and III category)

Description: The atmosphere of experience is a background feeling that describes the frame of participant’s experience. It describes those aspects of experience that do not carry with them a specific content, but rather give color to the phenomena at the front of participant’s consciousness.

Subcategories:

Positive neutrality;

Fatigue;

Informational chaos;

Faith in recognition.

Considerations: Atmosphere of experience is different from the attitude towards the task in that the latter clearly takes the task as its object. Atmosphere of experience in and of itself has no object. It amounts to a deeper and more essential feeling, intimately related to the theoretical and philosophical concept of existential feelings (cf. Ratcliff, 2008).

3.2.3.1.1 Positive neutrality (level II category)

Description: Positive neutrality is an atmosphere of experience in which the participants are aware of the absence of any kind of disturbance. It is the sense of the smooth running of events. This smoothness; however, is not experienced as positive in and of itself: it is a noticeably neutral aspect of experience. Because the participants are aware of nothing being wrong, this neutrality is experienced as something good, as something mildly positive.

Values:

Smoothness of performance;

Calmness;

Rhythm;

Metacognitive feeling.

3.2.3.1.2 Smoothness of performance (level I category)

Description: Smoothness of performance is an atmosphere of experience in which the participants are aware of positive neutrality at their own task performance. The task - and its performance - run smoothly, without any interruptions. This smoothness in and of itself is not pleasant. The absence of disturbances is the aspect of experience that is perceived as positive.

Values: None.

Examples:

> "Everything was related to the rhythm. It created this flow. This rhythm was silent, discrete, a staccato. I moved my eyes according to where I was expecting the dot to appear. When it didn't appear, I got a sense of dissonance. It went outside of the rhythm. It's a very mental experience, yes, but it is not reasoning. It wasn't as if I said 'Aha, it went out of rhythm, therefore, it does not fit.' It was a more subtle feeling. Like an *aha moment.* The dot was not here, so it must be there. When the dot was in an unexpected place, the rhythm broke down. And so did any image that I had in my mind." (VR.WM.1.19-02-P-02)

> "A rhythm of performing the task established itself. At the end, I answer completely automatically. If there's a mismatch, this way of solving the task stops." (VR.WM.1.17-04-O-02)

3.2.3.1.3 Calmness (level I category)

Description: Calmness is an atmosphere of experience whereby the participants feel pleasant in the absence of disturbances. Calmness itself has no object. It describes the general way of existing in a particular moment.

Values: None.

Examples:

> "[The association of a kite] has awoken certain feelings in me. But these feelings weren't too intense. Without these feelings I could not remember this association. Because of it, I could relax and I wasn't fully aware of the presentation of the task." (VR.WM.1.14-04-P-02)

> "The whole task felt light. I can feel these positive feelings. It's not something extra. Performing the task just feels nice." (VR.WM.1.24-03-P-02)

Considerations: Calmness is a different experiential category from unburdedness in that the former has no object. It is an undirected sense of peace. Conversely, the latter has an object in the form of task-performance. Specifically, it amounts to an awareness that even if the participants does poorly at the task, this will have no negative impact neither on them nor on the research.

3.2.3.1.4 Rhythm (level I category)

Description: The experience of rhythm is by and large tied to the appearance of stimuli themselves. This experiential category refers to an embodied (and sometimes even auditory; when accompanied by the category acoustic) element of experience. Participants report that rhythm structures their time and that task-performance in consequence becomes more relaxed and predictable. Rhythm can be embodied and is commonly tied to the feeling of heart rate.

Similarly to the smoothness of performance, rhythm can break down in the case of a mistake or a disturbance in experiential field. This leads to the tempo stopping. Insofar as the disturbance dos not occur, the rhythm encompasses the final key press as well.

Examples:

> "I said to myself, 'one, two, three, four.' This happened in the same rhythm as the dots appeared in. these verbalizations were sounds that happened in my own voice. The rhythm helped me concentrate. It gave structure to my sense of time. It wasn't intended to be a dancing rhythm but it reminded me a lot about dance where you have to count [...] I'm the author of these words. I willfully produce them. When they first appeared, I didn't willfully create them. It wasn't my strategy at first. But then I noticed that I'm doing it and that it feels good. What felt good was the structure. I latched on to that and started actively doing it." (VR.WM.1.04-02-P-01)

> "There was no sound to it. The tempo was constantly the same even though some things may have changed. I felt the rhythm in my mind as a kind of presence. I might have even been creating it but it is hard to say." (VR.WM.1.25-02-P-01)

Considerations: We have to be careful in distinguishing rhythm from two related experiential categories: the acoustic and smoothness of performance.

The acoustic refers to an auditory experience that is tied to the appearance of the stimuli or the objects making up the stimulus. These objects are related to an experienced sense of sound. Rhythm is not necessarily an auditory experience. It can range from being a bodily feeling to merely being an awareness of a sequential, repetitive activity. This can be discerned in the reports themselves in the cases when rhythm co-occurs with the acoustic. One commonly *creates* the other. Whether it is the rhythm the one that induces sound or the sound structuring time so that it forms rhythm. Then again, it is also possible that rhythm and acoustic do not appear simultaneously at all. This is especially apparent in cases where there is a discernible intonation present in the acoustic.

Smoothness of performance is an experience that is structurally similar to rhythm from the point of view that once it is present, it appears as the default state of the experiential landscape. Both aspects of experience may be cut short by a disturbance,. The difference is that smoothness of performance is by and large unconscious. Rhythm, on the other hand, clearly constitutes a presence.

3.2.3.1.5 Fatigue (level II and I category)

Description: Fatigue is an atmosphere of experience that is related to participants becoming sleepy. It refers to an entirely bodily feeling of being tired and having to fall asleep. Most commonly, it appears as a burning, heavy sensation in the eyes, bodily weight, and slowing down of mental gestures.

Values: None.

Examples:

> "I suddenly became aware of the light. Of how strong it is. I am actually still aware of that now. It was as if the light burnt me a little bit. This is why the task seemed more difficult. I think I had to pay much more attention to it. The task was suddenly very far away. It was as if I did not have enough focus." (VR.WM.1.31-02-P-02)

> "Sleepiness lingered on. But I no longer feel it in my body. it 's mental now. Or maybe a little bit bodily as well. I think I am slower in the sense that I can follow the dots with the same speed as they are appearing. I can't keep up. They are much faster from my eyes." (VR.WM.1.25-04-P-03)

3.2.3.1.6 Informational chaos (level II and I category)

Description: Informational chaos describes the experience where the visual-spatial working memory task seems so complex as to be impossible to solve. It seems impossible to memorize the memory stimuli on account of how complex they seem. Participants commonly experience this complexity as the absence of any stable point at which to direct their attention in order to start memorizing the stimulus. It is an exceedingly unpleasant experience and it is accompanied by feelings of anxiety and fear.

Values: None.

Example:

> "I ran out of focus, but not because I was wrong with the previous one. It was simply no longer clear what I have to do. I don't understand what is happening on the screen. I don't know what to do. It was really, really uncomfortable. This panic was much worse than if I was just wrong. I almost started crying. I felt totally powerless. I felt this tension that was rising from my chest to my clavicles. I felt that I have an increased heart rate. I didn't know what to do. There was a confusion in my mind. I didn't know what to focus on. There was a lot of pondering, but actually, I didn't really have any thoughts. It was all mostly bodily experience. I couldn't stop it. I couldn't make a decision about what to do. This panic was the only thing I was really experiencing. I wasn't solving the task. I was just randomly clicking. Because I knew I had to. And there was this idea of a record of everything that I don't click on it." (VR.WM.1.25-01-O-02)

3.2.3.1.7 Faith in recognition (level II and I category)

Description: Faith in recognition describes an atmosphere of experience whereby the participants have faith that in the moment of the test stimulus, the answer will be available to them. This does not describe an explicit awareness of a representation, but rather a subtle belief that they somehow memorized the memory stimulus. It is a very relaxed, light experience. At its most apparent form, this category refers to simply knowing that they will be able to solve the task with minimal possible amount of conscious mental activity. In the weakest sense, this is the belief that they will be able to discern any changes in the test stimulus.

Values: None.

Examples:

> "I know I can do it. It's just that I don't know how exactly I know. When I look at the memory stimulus, it just seems like I will be able to remember it." (VR.WM.1.07-01-C-02)

> "This is not some surface experience. In the sense that I could name it. It's much more raw. It's not as if I concretely saw the colors. It's just a faith that I can. Everything was out there. I didn't have to add anything extra to it." (VR.WM.1.23-02-C-02)

4 References

Froese, T., Gould, C., & Seth, A. K. (2011). Validating and Calibrating First- and Second-person Methods in the Science of Consciousness. *Journal of Consciousness Studies, 18*(2), 38–64.

Froese, T., Gould, C., & Barrett, A. (2011). Re-viewing from within: A commentary on first- and second-person methods in the science of consciousness. *Constructivist Foundations,* 6(2), 254–269.

Hurlburt, R. T., & Heavey, C. L. (2006). *Exploring inner experience.* . Amsterdam: Johns Benjamins.

Kordes, U., Oblak, A., Smrdu, M., & Demšar, E. (2019). Ethnography of meditation: An account of pursuing meditative practice as a tool for researching consciousness. *Journal of Consciousness Studies, 26*(7-8), 184–237.

Nelson, J. (2017). Using conceptual depth criteria: addressing the challenge of reaching saturation in qualitative research. *Qualitative research, 17*(5), 554–570. doi:doi.org/10.1177/1468794116679873
